# Synovial cell cross-talk with cartilage plays a major role in the pathogenesis of osteoarthritis

**DOI:** 10.1038/s41598-020-67730-y

**Published:** 2020-07-02

**Authors:** Ching-Heng Chou, Vaibhav Jain, Jason Gibson, David E. Attarian, Collin A. Haraden, Christopher B. Yohn, Remi-Martin Laberge, Simon Gregory, Virginia B. Kraus

**Affiliations:** 1 Duke Molecular Physiology Institute, Duke University School of Medicine, Durham, NC 27701 USA; 2 Division of Rheumatology, Department of Medicine, Duke University School of Medicine, Durham, NC 27701 USA; 3 Department of Orthopaedic Surgery, Duke University School of Medicine, Durham, NC 27701 USA; 4 Unity Biotechnology, South San Francisco, CA USA

**Keywords:** Molecular biology, Rheumatic diseases, RNA sequencing

## Abstract

We elucidated the molecular cross-talk between cartilage and synovium in osteoarthritis, the most widespread arthritis in the world, using the powerful tool of single-cell RNA-sequencing. Multiple cell types were identified based on profiling of 10,640 synoviocytes and 26,192 chondrocytes: 12 distinct synovial cell types and 7 distinct articular chondrocyte phenotypes from matched tissues. Intact cartilage was enriched for homeostatic and hypertrophic chondrocytes, while damaged cartilage was enriched for prefibro- and fibro-, regulatory, reparative and prehypertrophic chondrocytes. A total of 61 cytokines and growth factors were predicted to regulate the 7 chondrocyte cell phenotypes. Based on production by > 1% of cells, 55% of the cytokines were produced by synovial cells (39% exclusive to synoviocytes and not expressed by chondrocytes) and their presence in osteoarthritic synovial fluid confirmed. The synoviocytes producing IL-1beta (a classic pathogenic cytokine in osteoarthritis), mainly inflammatory macrophages and dendritic cells, were characterized by co-expression of surface proteins corresponding to *HLA-DQA1*, *HLA-DQA2*, *OLR1* or *TLR2*. Strategies to deplete these pathogenic intra-articular cell subpopulations could be a therapeutic option for human osteoarthritis.

## Introduction

Osteoarthritis (OA) is a leading cause of joint pain and disability and health-care costs worldwide^1^. Although OA is now considered a complex whole joint disease involving cartilage degeneration, osteophyte formation, subchondral sclerosis and inflammation of the synovial membrane^2^, little is known about the disruption of the physiological relationship and cross-talk between these tissues that contributes to the pathogenesis of OA. Thus, systematic molecular changes in the whole joint organ in human OA are still largely unknown and direct evidence of phenotypic and functional heterogeneity of both synoviocytes and chondrocytes has been lacking. The goal of this study was to elucidate the molecular cross-talk between cartilage and synovium and the contribution of the synovium to disease and cartilage degradation in OA using single-cell RNA-sequencing (scRNA-seq).

scRNA-seq allows assessment of fundamental biological properties of cell populations and biological systems at an unprecedented resolution^3^. To date, most prior gene expression analyses of OA joint tissues, including our own, have performed “bulk” tissue analyses^4–6^. This is problematic for a tissue such as synovium, whose cellular constituents are known to be highly heterogeneous. Evidence suggests that many cell populations, including synovial fibroblasts^7–10^ and immune cells^11–14^, play a role in OA pathogenesis, although it is not clear which cell type may be most important at the various stages of OA development and severity. Bulk tissue analysis is also problematic for cartilage. Although cartilage has long been believed to consist of only one dominant cell type, recent evidence derived from other scRNA-seq studies suggests that cartilage contains cells of many different phenotypes^15^. The potential upstream regulators of these different chondrocyte phenotypes and the tissue(s) of origin of the regulators remain largely unknown. Such information could provide insights into the development and progression of OA from which new therapies might arise.

We performed single-cell transcriptomic analysis on OA knee joint tissues to systematically identify cell types and states within human osteoarthritic (OA) synovium and matched cartilage as well as determined regulators of these articular chondrocyte phenotypes. We used immunofluorescence analysis of synovial tissue to validate our scRNA-seq findings at a protein level and to spatially integrate discovered cell types and states into the anatomy of the OA synovium. Our results elucidate the contribution of synovium to disease and cartilage degradation and the cross-talk between OA synovium and cartilage, including the pathways and cell origin of mediators involved in the pathophysiology of OA. Surface markers, HLA-DQA1, HLA-DQA2, OLR1 or TLR2, identified synoviocytes that co-expressed IL-1beta protein and other pro-inflammatory mediators, providing potential identifiers to aid targeting of these pathogenic intra-articular cell subpopulations for depletion or reprogramming.

## Results

### scRNA-seq reveals twelve distinct cell types in the synovium

**Figure 1:**
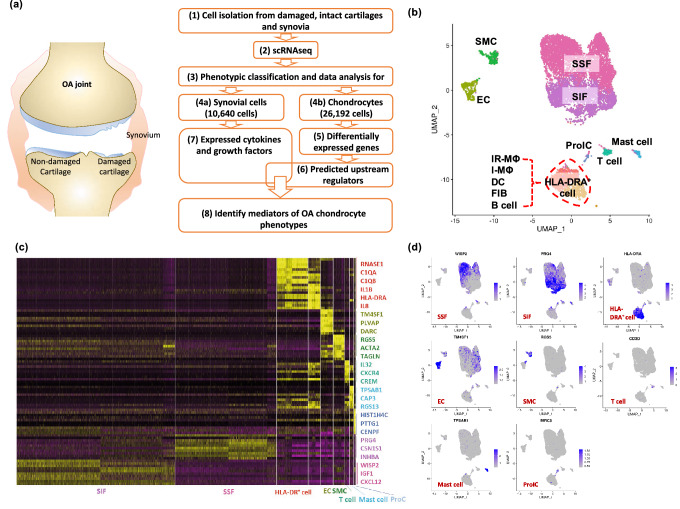
Single-cell RNA-Seq of human OA synoviocytes. (**a**) Flowchart shows the experimental strategy for systematically identifying cell diversity of synovium and cartilage in the pathogenesis of knee OA. (**b**) uniform manifold approximation and projection (UMAP) plot of scRNA-seq show unsupervised clusters colored according to putative cell types among a total of 10,640 cells in OA synovia. 44.1%, 33.2%, 12.82%, 3.63%, 3.28%, 1.35%, 1.13%, 0.49% of total acquired cells were synovial subintimal fibroblasts (SSF), synovial intimal fibroblasts (SIF), *HLA-DRA*^+^ cells (including immune regulatory [IR-MΦ] and inflammatory macrophages [I-MΦ], dendritic cells [DC], activated pro-inflammatory (*HLA-DRA*^+^) fibroblasts [iFIB] and B cells), endothelial cells (EC), smooth muscle cells (SMC), T cells, mast cells and proliferating immune cells (ProIC), respectively. (**c**) Heatmap of unsupervised clustering analysis shows the top ten highly expressed genes per cell type as determined by Seurat analysis with the top three genes per cluster highlighted on the right. Expression level is scaled based on z-score distribution. (**d**) Expression of the selected top marker genes for each cell type is shown in UMAP plots.

Profiling of 10,640 synoviocytes from a total of three individuals with knee OA (Fig. 1a) yielded a total of 21,253 identified genes. A total of 3,138 genes selected by unsupervised clustering revealed great heterogeneity of cell types, namely twelve distinct cell populations (Fig. 1b) including (from most to least abundant), synovial subintimal fibroblasts (SSF), synovial intimal fibroblasts (SIF), *HLA-DRA*^+^ cells (immune regulatory (IR-MΦ) and inflammatory macrophages (I-MΦ), dendritic cell (DC), activated pro-inflammatory (*HLA-DRA*^+^) fibroblasts (iFIB) and B cell clusters), smooth muscle cells (SMC), endothelial cells (EC), T cells, mast cells, and proliferating immune cells (ProIC). The identity of the 12 distinct cell subtypes could be confidently defined on the basis of the differentially expressed genes within each cell cluster (Fig. 1c, Supplementary Tables S1A,B). A representative gene for each cluster, mapped onto the UMAP plots, demonstrates the distinctly different nature of each major cell type (Fig. 1d). The majority of acquired synoviocytes were SSF and SIF (77.26%). These two cell types were readily distinguished in that SSF expresses collagen genes and stromal cell-derived factor 1 (*CXCL12*) for the synthesis of extracellular matrix components (Supplementary Fig. S1e–h); and SIF expresses genes producing essential constituents of synovial fluid including lubricin (*PRG4*) and hyaluronan (*HAS1*), and the synovial fibroblast biomarker, *HTRA1*, a secreted enzyme that regulates the availability of insulin-like growth factors (IGFs) by cleaving IGF-binding proteins (Supplementary Fig. S1i–k). Canonical gene markers for natural killer (NK) and NKT cells, *NCAM1* (CD56)^16,17^ and *ZBTB16* (PLZF)^18,19^, respectively, were not detected in the cell expression profiles of our OA synoviocytes.

### Identification of cell types in the *HLA-DRA*^+^ population

**Figure 2:**
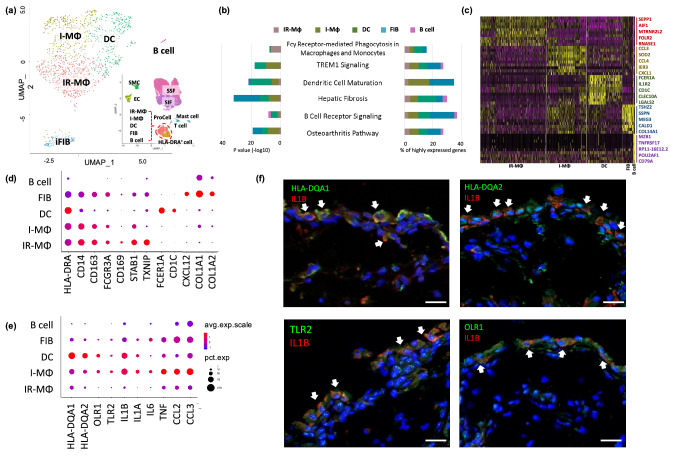
Identification of specific *HLA-DRA*^+^ cell subtypes in OA synovia. (**a**) The *HLA-DRA*^+^ cell populations consisting of five distinct cell subtypes are shown in a UMAP plot. (**b**) Bar graphs demonstrate the top ranked canonical pathways associated with each cell subtype based on their percentage of highly expressed genes (right) and their significance (left). Pathways related to these cells include: Fcγ receptor-mediated phagocytosis; TREM1 signaling; dendritic cell maturation; hepatic fibrosis; B cell receptor signaling; and multiple cell populations associated with OA. (**c**) Heatmap shows the top ten highly expressed genes for each cluster with the top five highly expressed genes per cluster highlighted on the right. The top highly expressed genes in IR-MΦ were *SEPP1* and *FLOR2* that are known to play a role in macrophage polarization and specifically expressed in regulatory macrophages, respectively. The top highly expressed genes in I-MΦ were inflammatory mediators, including *CCL3* and *CCL4*. The top highly expressed genes in iFIB were collagen genes including *COL14A1* and *COL1A1*. Consistent with identification as B cells were cells with high expression of *MZB1*, *TNFRSF17* and *CD79A*. (**d**, **e**) The dot plots depict the average expression level (color scale) and percentage of cells expressing the selected marker genes (dot size) for each cluster. (**d**) The dot plot shows expression of *HLA-DRA* in all five cell subtypes and co-expression with an additional 11 markers (d) and additional immune markers and cytokines (**e**). (**d**) Classic macrophage marker genes (*CD14*, *CD163* and *FCGR3A*) were highly expressed in IR-MΦ, I-MΦ and iFIB. Immune regulatory genes, *CD169*, *STAB1* and *TXNIP*, were highly expressed in IR-MΦ. DC marker genes, *FCER1A* and *CD1C* were exclusively expressed in DC. Fibrous matrix genes (*COL1A1* and *COL1A2*) and stromal cell-derived factor 1 (*CXCL12*) were highly expressed in iFIB. (**e**) Surface maker genes (*HLA-DQA1*, *HLA-DQA2*, *OLR1* and *TLR2*) and cytokines were highly expressed in I-MΦ and DC. (**f**) Representative immunofluorescence staining showing co-expression of IL1B and surface biomarkers of I-MΦ and DC, such as HLA-DQA1, HLA-DQA2, OLR1 and TLR2 in human OA synovium. Scale bar = 20 μm.

The HLA-DRA^+^ cell population comprised 12.8% of the total acquired cells in OA synovia and were enriched for cells with high expression of MHC class II genes, such as *HLA-DRA*, *DRB1*, *DPA1* and *DQA1*. These cells comprised five subclusters or cell types consistent with heterogeneous groups of macrophages (IR-MΦ and I-MΦ), DC, iFIB and B cells (Fig. 2a). Pathways related to these cells included: Fcγ receptor-mediated phagocytosis; TREM1 signaling, which plays important roles in innate immune responses, such as activating inflammatory responses^20^; dendritic cell maturation; hepatic fibrosis associated with an accumulation of extracellular matrix (ECM) proteins^21^; the pathway related to B cell receptor signaling; and multiple cell populations associated with OA including I-MΦ, DC and iFIB (Fig. 2b). A heat map of the differentially expressed genes (*p* value threshold < 0.05 and log fold change (FC) > 0.25 compared to other clusters) for each HLA-DRA^+^ cell type demonstrates their distinctly different transcriptomes (Fig. 2c). Interestingly, the HLA-DRA^+^ iFIB cells, like IR-MΦ, I-MΦ and DC, all expressed *CD14* (Fig. 2d). The *CD14*^+^ iFIB were definitively identified as fibroblasts due to high expression of *CXCL12* and collagen (*COL1A1*, *COL1A2*, *COL3A1* and *COL14A1*) genes (Fig. 2d). Cell surface CD14 on blood-borne fibrocytes is a biomarker of cells that can rapidly enter sites of tissue injury and be involved in chemokine/chemokine receptor interactions^22^, suggesting a critical role for these iFIB cells in wound repair.

IR-MΦ differentially expressed genes for signaling pathways related to immune regulation such as Stabilin 1 (*STAB1*), a homeostatic receptor linking signals from extracellular to intracellular vesicular processes^23^, Thioredoxin Interacting Protein (*TXNIP*), an inhibitor of NF-κB activity^24^ and *CD169*, a macrophage specific marker of immunoregulation and inflammation^25^ (Fig. 2d and supplementary Fig. S2). We therefore confidently defined this macrophage cell type as an immune regulatory macrophage (IR-MΦ). I-MΦ like IR-MΦ expressed classic macrophage markers (such as CD163) but were distinguished from IR-MΦ by high expression of proinflammatory cytokine genes including *IL1B*, *IL1A*, *IL6*, *TNF*, *CCL2*, and *CCL3* (Fig. 2e and supplementary Fig. S2a). DC also highly expressed these pro-inflammatory cytokine genes (Fig. 2e) and *FCER1A*, a high-affinity Fc-gamma receptor, and *CD1C*, biomarkers for classic dendritic cells^26^ (Fig. 2d and supplementary Fig. S2a). Interestingly, cell surface proteins encoded by *HLA-DQA1*, *HLA-DQA2*, *OLR1* and *TLR2* were more highly expressed in I-MΦ and DC than IR-MΦ suggesting they might be used to target pro-inflammatory cytokine producing cells (Fig. 2e). We evaluated expression levels of these genes in publicly available bulk RNA gene expression profiling data (GSE1919, GSE41038, GSE55457, GSE55235 and Lambert et al.’s study)^27–30^ from non-disease and OA synovial tissue (Supplementary Table S2). With the exception of TLR2, one or more of the datasets with publicly available data demonstrated an upregulation of each of these mediators in OA relative to control; in no case was there a down-regulation of any of these markers in OA relative to control. We also confirmed co-expression of these cell surface markers with IL-1beta protein (Fig. 2f). As expected, cytokines such as CCL3 protein were not expressed by IR-MΦ that were distinguished by their expression of CD169 and STAB1 proteins (Supplementary Fig. S2b,c.

### Identification of chondrocyte phenotypes in OA

**Figure 3:**
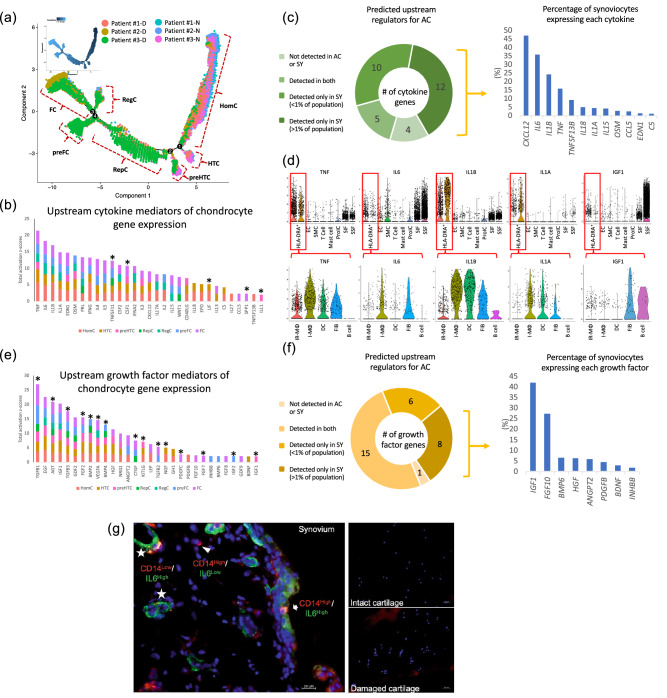
Identification of chondrocyte cell types in OA and the potential upstream regulators of these phenotypes. **a** shows a similarity across patients and a predominance of preFibrochondrocytes (preFC), Fibrochondrocytes (FC), Regulatory chondrocytes (RegC), Reparative chondrocytes (RepC) and some preHypertrophic chondrocytes (preHTC) in diseased areas designated ‘**D**’ corresponding to the medial tibial plateau (MT); **a** also shows a predominance of Homeostatic chondrocytes (HomC), some PreHTC and Hypertrophic chondrocytes (HTC) in non-diseased areas designated ‘**N**’ corresponding to OLT or the lateral tibial plateau. Potential upstream cytokines (**b**) and growth factors (**e**) were identified based on all highly expressed genes from each cluster and scaled by activation z-score; asterisks indicate the upstream mediators that were expressed by > 1% of acquired chondrocytes. Nearly all cytokines (**c**) and growth factors (**f**) were expressed by synoviocytes: 22 of 31 cytokines and 14 of 30 growth factors were only expressed by synoviocytes and not by chondrocytes (the proportion of acquired synoviocytes expressing each cytokine and growth factor are shown in the pie charts). (**d**) Violin plots show expression levels of representative cytokines (*TNF*, *IL6*, *IL1B and IL1A*) and a growth factor (*IGF1*) across all cell types in OA synovia and the *HLA-DRA*^+^ cell subtypes. (**g**) Representative immunofluorescence staining of synovium and cartilage (intact and damaged regions of a single patient specimen) for IL6 and CD14. As expected, the damaged cartilage is hypercellular compared to intact cartilage due to the development of multicellular chondrons in damaged cartilage with osteoarthritis. IL6 was highly expressed by CD14^+^ synovial fibroblasts (SSF, SSF and iFIB), I- MΦ, and SMC, but not chondrocytes from either intact or damaged regions of cartilage.

We isolated chondrocytes from articular cartilages collected from joint replacement surgery of patients with OA. We profiled a total of 26,192 cells, 14,613 cells from intact cartilage sites of the outer lateral tibiae (OLT), and 11,579 cells from damaged cartilage sites of the medial tibiae (MT) (Fig. 1a) yielding a total of 21,866 identified genes (20,770 from OLT and 21,034 from MT, 19,918 in common between them). Gene expression of chondrocytes from the more degenerated medial tibial plateau compared to the macroscopically normal outer lateral tibial plateau revealed marked gene expression differences in the two compartments (Supplementary Table S3). Chondrocytes from MT regions highly expressed numerous OA progression related genes, such as *S100A4*, *COL1A1*, *COL1A2*^5^, COL5A1^15^, *TNFAIP6*^31^ and *ADAMTS5*^32^. To gain insight into the transcriptional changes of chondrocytes in OA, we performed an unsupervised pseudotime trajectory analysis of the top 1,000 variably expressed genes yielding a total of seven distinct states/cell types of chondrocytes (Fig. 3a and Supplementary Table S4). Similar to a previous study^15^, based on GO pathways of the differentially expressed genes, we identified homeostatic chondrocytes (HomC), hypertrophic chondrocytes (HTC), prehypertrophic chondrocytes (preHTC), regulatory chondrocytes (RegC) and fibrochondrocytes (FC) (Table 1). In addition, we also discovered two unique subtypes of chondrocytes, which we named based on their gene expression profiles, reparative chondrocytes (RepC), and prefibrochondrocytes (preFC) (Table 1). The preHTC and HTC cell types were similar (shared 57% of their differentially expressed genes); preFC and FC were also similar (shared 18% of their differentially expressed genes). HomC cells were distributed at the root of the trajectory, followed by a branch for HTC, preHTC, and RepC, followed by another three branches consisting of RegC, preFC and FC. Intact (lateral tibial) cartilage was enriched for HomC or HTC chondrocyte cell types, while damaged (medial tibial) cartilage was enriched for FC, preFC, RegC, RepC and preHTC (Fig. 3a). Key pathways in which these cell types are involved include modulation of cellular homeostasis in response to external stimuli (HomC), skeletal development (HTC and preHTC); ECM components (preHTC), extracellular matrix signaling and collagen fibril organization (RepC), ECM organization and disassembly (preFC and FC), signaling pathways related to response to endogenous stimuli and inhibition of biological processes (RegC) (Table 1). Interestingly, with respect to differential expression, the FC chondrocytes, enriched in damaged regions of cartilage, were the primary source of a host of OA related proteases (ADAMTS1, ADAMTS5, ADAMTS6 (also from preFC), MMP2, MMP14, HTRA1 (also from preFC)). The HomC and HTC chondrocytes, enriched in non-damaged cartilage, were primary sources of MMP3 and the anti-protease SERPINA1 (Alpha-1 antitrypsin), respectively.

**Table 1:** A summary of chondrocyte subtypes with key expressed genes and involved pathways.

Cell type	Key genes	Key involved pathways	False discovery rate
Homeostatic chondrocyte (HomC)	MMP3, FOSB, JUN	Regulation of gene expression	1.35E−09
		Response to external stimulus	1.06E−09
Hypertrophic chondrocyte (HTC)	COL10A1, IBSP, JUN	System development	9.90E−09
		Multicellular organismal development	3.89E−08
Pre-hypertrophic chondrocyte (preHTC)	COL10A1, IBSP, COL2A1	System development	5.50E−09
		Skeletal system development	6.63E−08
Reparative chondrocyte (RepC)	COL2A1, CILP, COL3A1, COMP	Extracellular matrix organization	3.98E−10
		Collagen fibril organization	1.41E−06
Pre-Fibrochondrocyte (preFC)	IL11, COL2A1, CILP, OGN	Extracellular matrix organization	5.28E−22
		Extracellular matrix disassembly	1.34E−08
Fibrochondrocyte (FC)	COL1A1, COL1A2, S100A4, PRG4	Extracellular matrix organization	1.27E−33
		Movement of cell or subcellular component	7.94E−17
Regulatory chondrocyte (RegC)	CHI3L1, CHI3L2	Negative regulation of biological process	1.12E−04
		Cellular response to metal ion	1.32E−06

### Identification of potential upstream mediators regulating chondrocyte phenotypes in OA

Secreted inflammatory cytokines and growth factors are widely accepted as critical mediators of the disturbed homeostasis implicated in the pathophysiology of OA^33^. Therefore, potential upstream cytokine and growth factor regulators of the chondrocyte genes distinguishing each chondrocyte cell type were identified based on prior knowledge stored in the IPA database of expected effects between transcriptional regulators and their target genes. A total of 31 upstream cytokines with significant activation z-scores (z-scores ≥ 2, *p* values < 0.01) were identified (Fig. 3b and Supplementary Table S5A). We hypothesized that the main cytokine regulators were secreted by cells from synovial tissues. Based on a threshold of > 1% of cells expressing the gene, 17 of the 31 cytokines were expressed by synoviocytes. Twelve of the 31 cytokines (*TNF*, *IL6*, *IL1B*, *IL1A*, *EDN1*, *OSM*, *CXCL12*, *IL15*, *IL18*, *C5*, *CCL5*, *TNFSF13B*) were exclusively expressed by synoviocytes and not chondrocytes (in > 1% of synoviocytes but < 1% of chondrocytes); *TNF*, *IL6*, *IL1B*, and *IL1A*, were expressed by 16.16%, 36.17%, 24.44%, and 4.74% of acquired synoviocytes, respectively (Fig. 3c, Supplementary Fig. S3 and S4, Supplementary Table S6). Except for B cells that lacked *TNF* expression, *TNF* was expressed by most *HLA-DRA*^+^ cells and most highly expressed by I-MΦ. *IL1B* and *IL1A* were most highly expressed by I-MΦ, DC, and iFIB. *IL6* was expressed by I-MΦ, iFIB, SSF, SIF and SMC (Fig. 3d). For the OA-related cytokines, *IL1A*, *IL1B*, *IL6* and *TNF*, whose average expression in synovium exceeded that of damaged cartilage by at least 25 fold, we were unable to detect any gene expression by chondrocytes from non-damaged and damaged full thickness regions of knee articular cartilage by qPCR in another independent OA cohort (n = 10) (Supplementary Fig. S5). Interestingly, based on the threshold of > 1% cells expressing the gene, only five of the total 31 predicted upstream cytokines were detected in chondrocytes, including *TNFSF11*, *CSF1*, *LIF*, *SPP1* and *IL11* (Fig. 3b and Supplementary Table S5A). By scRNA data, among the 31 upstream cytokines, 3 cytokines (*IFNA2*, *IL3*, *IL17A*) were not detected in synovial or cartilage tissues. The remaining five cytokines (*TNFSF11*, *CSF1*, *LIF*, *SPP1*, *IL11* and) were detected in both synoviocytes and chondrocytes; among these the expression of only one cytokine, SPP1, was far greater in chondrocytes than synoviocytes (Supplementary Table S5A).

A total of 30 upstream growth factors with significant activation z-scores were identified. Based on a threshold of > 1% of cells expressing the gene, 8 growth factors (*IGF1*, *HGF*, *ANGP2*, *PDGFB*, *FGF10*, *INHBB*, *BMP6*, *BDNF*) were exclusively expressed by synoviocytes, no growth factors were exclusively expressed by chondrocytes, while 15 (50%) of the 30 growth factors (*TGFB1*, *TGFB2*, *TGFB3*, *AGT*, *FGF2*, *FGF7*, *BMP2*, *BMP4*, *VEGFA*, *CTGF*, *KITLG*, *NGF*, *PDGFC*, *IGF2*, *FGF1*) were expressed by both synoviocytes and chondrocytes (Supplementary Table S5B and Fig. 3e, f). We confirmed (by ELISA, human protein cytokine array and in a few cases, by published literature) the protein expression in OA synovial fluid of the majority (19 of 20) of the upstream inflammatory cytokines and growth factors whose gene expression was in > 1% of acquired cells and predominant in synoviocytes (Table 2 and Supplementary Figs. S6, S7 and S8); only BMP6 was not detected in OA synovial fluid. By scRNA-seq data, one of the predicted growth factors, GDF2, was not detected.

**Table 2:** Potential upstream regulators for OA pathogenesis from synoviocytes in OA synovial cells.

Predicted upstream regulators for OA chondrocytes	Total activation z-scores	Proportion of expressing synoviocytes (%)	Proportion of expressing HLA-DR^+^ cells (%)	Highly expressing cell types	Concentration of detected mediator in OA SF	Method of detection
*Cytokines*						
TNF	21.32	16.16	60.26	I-Mφ	2.34 ± 0.74 pg/mL	Array* and Chou et al. (PMID: 29129649)
IL6	18.27	36.17	25.44	I-Mφ, SSF, iFIB SMC	105.76 ± 193.61 pg/mL	Array* and Chou et al. (PMID: 29129649)
IL1B	17.01	24.44	61.88	I-Mφ, DC	1.02 ± 0.15 pg/mL	Array* and Chou et al. (PMID: 29129649)
IL1A	14.82	4.74	29.91	I-Mφ, DC	2.90 ± 2.64 pg/mL	Array* and Chou CH et al. (PMID: 29129649)
EDN1	14.73	1.93	1.98	EC	3.0 ± 0.5 pg/mL	Nahir et al. (PMID: 1865412)
OSM	13.87	3.17	17.38	HLA-DR^+^ cell Mast cell	39.7 ± 108.0 pg/mL	Beekhuizen et al. (PMID: 24027021)
CXCL12	8.99	47.13	14.74	SSF iFIB	++	Array*
IL15	7.84	4.72	8.43	Many synovial cell types	282.07 ± 203.47 pg/mL	Array* and Chou et al. (PMID: 29129649)
IL18	5.48	5.34	35.56	HLA-DR^+^ cell, Mast cell	623.81 ± 266.43 pg/mL	ELISA^§^
C5	4.16	1.45	1.17	Many synovial cell types	+++	Array* and Wang et al. (PMID: 22057346)
CCL5	2.24	2.84	4.91	T cell	305.52 ± 275.61 pg/mL	Array* and Chou et al. (PMID: 29129649)
TNFSF13B	2.22	9.66	31.89	HLA-DR^+^ cell	+++	Array*
*Growth factors*						
IGF1	20.32	42.34	18.77	SSF, iFIB, B cell	9.4 ± 4 nM/L	Denko et al. PMID: 11048621
HGF	11.38	6.54	5.13	SMC	338.2 ± 966.9 pg/mL	Array* and Mabey et al. (PMID: 24966080)
ANGPT2	9.32	6.20	1.17	EC, SMC	0.54 ± 1.41 ng/mL	Array* and Mabey et al. (PMID: 24966080)
PDGFB	2.60	4.81	27.27	I-Mφ	+	Human XL cytokine array
FGF10	2.43	27.71	3.74	iFIB	3.01 ± 4.40 ng/mL	ELISA^¶^
INHBB	2.21	2.06	0.51	Many synovial cell types	Activin, 311 ± 80.2 pg/mL Inhibin, 2.15 ± 1.18 μg/mL	El-Gendi et al. PMID: 20704626
BDNF	2.10	3.32	0.29	Many synovial cell types	224.76 ± 162.39 pg/mL	Chou et al. (PMID: 29129649)

*Human XL cytokine array from R&D systems.

^§^IL-18 ELISA assay from Meso Scale Discovery.

^¶^FGF ELISA assay from LSBio.

Based on our data, four of the major OA-related cytokines (*TNF*, *IL1B*, *IL1A* and *IL6*) and a major OA-related growth factor (*IGF1*) were predominantly expressed by synoviocytes. The specific synovial cell types producing each of these upstream mediators are shown in violin plots (Fig. 3d). *IL1A* appeared to be exclusive to immune (*HLA-DRA*^+^) cells. *TNF* and *IL1B* were also primarily expressed by immune cells (*HLA-DRA*^+^). *IL6* was primarily expressed by CD14^+^ synovial fibroblasts (SSF, SSF and iFIB), I-MΦ, and SMC (Fig. 3g). Interestingly, the proliferating immune cells (ProIC) of the synovium expressed low but detectable levels of *TNF*, *IL1B* and *IL6*.

**Figure 4:**
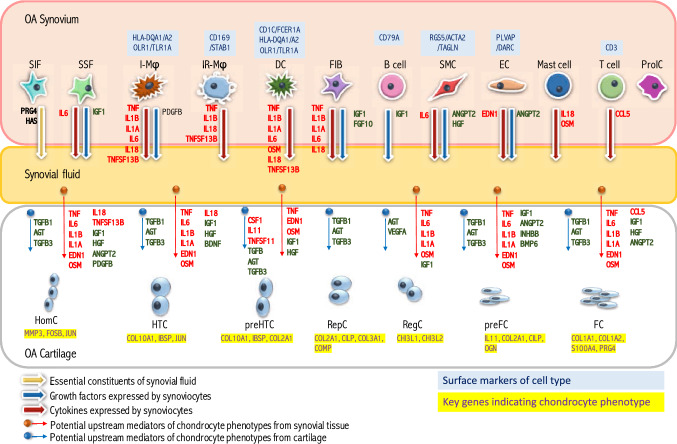
The cross-talk model of osteoarthritis. We identified 12 subpopulations from OA synovia and 7 distinct chondrocyte subpopulations from OA articular cartilage. We predicted potential upstream regulators of chondrocyte gene expression during OA progression to infer molecular cross-talk networks between cartilage and synovium. Genes expressed by OA chondrocytes and identified as potential mediators of chondrocyte phenotypes in OA are indicated by blue solid dots with arrows; the preponderance of growth factors, such as TGFB among them, is consistent with upregulation of anabolic processes to maintain cartilage homeostasis in OA. However, genes identified as potential mediators of chondrocyte phenotypes in OA that were exclusively expressed (in > 1%) by synoviocytes but not by any of the chondrocyte subtypes are indicated by red solid dots with arrows; among these were genes for several key pro-inflammatory cytokines implicated in the pathogenesis of OA, including IL1A, IL1B, IL6 and TNF that were specifically expressed by inflammatory macrophages (I-Mϕ), dendritic cells (DC) or other synoviocytes. I-Mϕ and DC expressed HLA-DQA1, HLA-DQA2, OLR1 or TLR2; these cells appeared to be the primary cytokine producing cells.

Collectively, our high-resolution single cell expression data provided insight into the spectrum of cellular heterogeneity within human OA cartilage and synovium. Twelve synovial cell types and 7 distinct chondrocyte cell types were identified in articular cartilage. Many of the signals regulating chondrocyte transcription in OA progression originate in the synovium, particularly from the I-MΦ and DC cell populations, not in the cartilage (Fig. 4).

## Discussion

Although substantial efforts have focused on molecular programs involved in OA progression among several types of human joint tissues^4–6,15,34^, only a few studies have examined molecular changes in multiple matched tissues in human OA^4^, and none have been carried out on a genomic scale. This study is the first, to our knowledge, that explored and characterized the cellular and transcriptional heterogeneity on a single cell level in matched cartilage and synovium from patients. To gain a deeper understanding of the biological cross-talk of tissues of the joint organ as they relate to the pathogenesis of OA, we evaluated the potential origins of upstream cytokines and growth factors that may control phenotypic changes of OA chondrocytes. Despite a limited number of study participants for scRNA-seq, we determined that the majority (55%) of key OA-related cytokines were produced by synoviocytes (expression of 38% of the cytokines was exclusive to synoviocytes based on an expression threshold of > 1% of cells) while only 16% of the cytokines were produced by chondrocytes; none were expressed exclusively by OA chondrocytes. We identified a distinct subset of HLA-DRA^+^ synoviocytes that highly expressed key pro-inflammatory cytokines related to the pathogenesis of OA, including *IL1B*, *IL1A*, *IL6*, *TNF*, *CCL2 and CCL3.* Importantly, these pro-inflammatory cytokine producing cells also highly expressed several genes encoding cell surface proteins including *HLA-DQA1*, *HLA-DQA2*, *OLR1* and *TLR2*. Therapeutic strategies for OA, based on depleting or reprogramming these cells intra-articularly could be developed based on targeting these specific cell populations. Given the emerging observation that synovitis is present in early OA (patients who have minimal radiographic signs of cartilage loss), targeting of these specific cell populations may represent an opportunity for early therapeutic interventions^35^. However, conclusions on pathogenic mechanisms in this study are limited by the lack of non-OA controls and small sample size, therefore, a therapeutic strategy based on these results will require validation of these candidates as markers of pathogenic cell subsets in a larger patient population at different stages of OA and confirmation of their paucity in healthy normal non-arthritic synovium.

Several clinical studies have provided strong evidence that synovitis is associated with further worsening of OA structure^36,37^. Expression of *IL1B*, *TNF*, *IL6*, *IL15* and *IL18*, some of the most extensively studied cytokines in OA, was detected in < 1% of chondrocytes but in 5–36% of synoviocytes ranging from sixfold (*IL15*) to 291 fold (*IL1B*) greater expression in synoviocytes compared to MT chondrocytes from damaged regions of cartilage (Supplementary Table S6); these key cytokines were predicted to be upstream regulators for altering the course of disease at the level of cellular communication for chondrocytes. These cytokines, up-regulated in OA, lead to cartilage degradation and have synergistic effects on signaling pathways that increase inflammation and cartilage breakdown^38^. *TNF* and *IL1B* are key inflammatory cytokines involved in the pathogenesis of OA by promoting catabolic and destructive processes^39^; they can also block chondrocyte synthesis of ECM components, such as type-II collagen^40,41^. Both *TNF* and *IL1B* were expressed in up to 60% of *HLA-DRA*^+^ acquired cells in synovial tissues. *TNF* was significantly highly expressed in I-MΦ and may modulate the phenotypic changes for all states of chondrocyte populations, except RepC. *IL1B* was significantly highly expressed in I-MΦ and DC, and was identified as an upstream regulator for all states of chondrocyte populations, except RepC and preHTC. The antagonist of IL-1beta, IL-1 receptor antagonist (IL-1Ra, gene *ILRN*), was expressed by < 1% of chondrocytes from the MT damaged cartilage and not detected by scRNA-seq of chondrocytes from non-damaged regions; lack of this specific countermeasure in cartilage may be one reason why IL-1beta is such a potent OA promoting cytokine. This observation is congruent with recent evidence demonstrating the importance of *IL1RN* genetic polymorphisms (that impact IL-1Ra protein levels) for incidence and severity of OA^42^.

*IL6*, a pleiotropic cytokine with pro- and anti-inflammatory properties^43^, was widely expressed by synoviocytes (36.17% of acquired cells) and significantly differentially expressed in I-MΦ, SSF, iFIB and SMC, which may modulate and regulate the phenotypic changes for HomC, HTC, RegC, preFC and FC. *IL18*, another cytokine belonging to the *IL1* superfamily, was mainly expressed in *HLA-DRA*^+^ cells and mast cells and may affect the phenotypic changes of HomC and HTC. *IL15*, whose synovial fluid expression is known to be elevated in early stages of OA^44^, was expressed in 4.72% of acquired synoviocytes and may modulate RegC, preFC and FC; this is of particular importance given that the FC (and preFC) appear to be important for disease related protease production.

By both average gene expression and percentage of expressing cells, genes for key OA-related proteases (Supplementary Table S6), such as aggrecanases (*ADAMTS4* and *ADAMTS5*), predominated in synovium compared with cartilage. Expression of cathepsins (B, D and K) were similar for synovium and cartilage. Of the many metalloproteinases (MMPs) in humans, those reported to cleave triple-helical collagen are the collagenases (MMP-1, MMP-8, and MMP-13), gelatinase A (MMP-2), membrane type 1 MMP (MMP-14), and weakly, membrane type 2 MMP (MMP-15)^45^; of these, only *MMP1* (synoviocytes only), *MMP2* and *MMP14* (both synoviocytes and chondrocytes) were detected. Thus, although both MMP-1 and MMP-13 are considered rate-limiting in the process of collagen degradation^46^, at the gene expression level, only *MMP1* was detected and only in synoviocytes. MMP-2, MMP-3 and MMP-9 are elevated in arthritis, and degrade denatured and non-fibrillar collagen and non-collagen matrix components of joints^46^. In addition, MMP-3 can also activate other MMPs such as MMP-1, MMP-7, and MMP-9, rendering MMP-3 crucial in connective tissue remodeling^46^. *MMP3* was highly expressed (based on both average cell expression and % of cells expressing) by both synoviocytes and chondrocytes; *MMP2* and *MMP3* were expressed by both synoviocytes and chondrocytes while *MMP9* was only expressed by synoviocytes (based on the threshold of > 1% cells expressing). With the exception of *IL1RN* described above, countermeasures, such as *SERPINA1* (alpha-1-antitrypsin) and the *TIMP*s (tissue inhibitors of metalloproteinases) appeared to be robustly expressed by both synoviocytes and chondrocytes. Notably, *ADAM12* and *CTSS* were found to be preferentially expressed in chondrocytes in MT over OLT. These proteases could contribute to an exacerbation of the cartilage damage given that MT is usually the most affected region of an OA knee. Thus, these scRNA-seq data provide unique insights into the potential origin of many proteases involved in degradation of tissues in OA; taken together, the data underscore a key role of OA synovium as a source of proteolytic activity in addition to the pro-inflammatory cytokines that regulate these proteases.

A novel IR-MΦ population was identified in our study characterized by high expression of immune regulatory genes, including *STAB1*, *TXNIP* and *CD169.* The role of this macrophage subtype in OA is unclear. *STAB1* is an endocytic scavenger receptor known to be expressed in alternatively activated macrophages participating in anti-inflammatory responses and phagocytosis^47^. Expression of *STAB1* allows macrophages to retain immunosuppressive activity^48^ and maintain tissue homeostasis via suppressing production of the profibrogenic chemokine *CCL3*^49^. *TNXIP*, a suppressor of TNF-α–activated NF-κB activity and an activator of IL-1beta secretion via interaction with NLPR3^50^, was also highly expressed in IR-MΦ, supporting the potential immune regulatory activity of the IR-MΦ population in OA. On the other hand, *CD169*, known as sialoadhesin, is a cell adhesion molecule found on the surface of macrophages present in affected tissues from patients with inflammatory disorders such as rheumatoid arthritis^51,52^. *CD169* has been shown to mediate both immunity and immune tolerance for macrophages and is considered a biomarker for highly pathogenic phagocytes, but the precise mechanisms remain unclear^25^. Taken together, this IR-MΦ population may play a role in clearance of cell remnants and degraded tissues and modulate immune responses of the synovium.

Despite the fact that cartilage has long been thought to consist of only one dominant cell type, consistent with a prior study^15^, we too identified multiple cell types in articular OA cartilage. In our study, using a different methodology and strategy for sample harvesting and processing compared with the previous study, we discovered five similar chondrocyte populations (HomC, preHTC, HTC, RegC, and FC) and two additional distinct chondrocyte populations (RepC and preFC). Three of their OA chondrocyte populations were not definitively identified in our analysis, including effector chondrocytes (EC), proliferative chondrocytes (ProC), and cartilage progenitor cells (CPC). Their EC were enriched for genes related to metabolism and cholesterol synthesis, such as *C2orf82*, *CLEC3A* and *CYTL1*; our RepC cluster was the most closely related population given differential expression for these marker genes. Their ProC population was enriched for genes related to RNA metabolic processes and RNA stabilization; our HomC population was the most similar given enrichment for genes involved in regulation of RNA splicing, RNA binding and Poly(A) RNA binding. However, their markers of CPC (*BIRC5*, *CENPU*, *UNE2C*, *DHFR* and STMN1) related to human OA cartilage regeneration were not significantly expressed in any of our putative chondrocyte populations.

HomC cells highly expressed genes for modulating cellular homeostasis (*MMP3*, *FSOB* and *JUN*) in response to external stimuli. Both HTC and preHTC highly expressed genes related to skeletal development (*COL10A1* and *IBSP*); 56.67% of the differentially expressed genes were differentially expressed in both HTC or preHTC. HTC were enriched in genes related to cellular homeostasis (JUN, FOS and TF); preHTC were enriched in genes related to ECM component (*CLEC3A*, COL9A2 and *COL11A2*).

RepC were enriched for extracellular matrix signaling and collagen fibril organization, such as *COL2A*, *CLEC3A*, *CILP* and *COMP*, indicating a high reparative ability. Both FC and preFC highly expressed a fibroblast-related gene, *CD55*, and were enriched mainly for type I collagen organization and ECM assembly (*COL1A2*, *COL5A1*, and *HTRA1*); these two cell states also shared 18.38% of their differentially expressed genes. PreFC highly expressed *IL11* that plays an essential role in ERK-dependent autocrine signaling and is required for initiation of fibrogenic protein synthesis^53^. FC were enriched for genes related to cell migration (*TMSB4X*) and vasculature (S100A4) as well as several OA related aggrecanases^54^ (ADAMTS1, ADAMTS5) and other proteases (MMP2, MMP 14 and HTRA1) suggesting they play a key role in OA pathogenesis. RegC were enriched for genes of signaling pathways related to response to endogenous stimuli and inhibition of biological processes (*CHI3L2*, *CLU*, *CHI3L1* and *CRTAC1*).

Non-OA healthy control synovium was unavailable for our research therefore we could not directly address the differences in cell patterns between OA and non-OA synovium. There is in general a significant knowledge gap in the literature concerning gene expression of non-arthritic healthy synovium and considerable ethical challenges to acquiring such samples. A number of studies have attempted to fill this knowledge gap but data from these synovial tissues have major limitations as they have been acquired as cadaveric tissue^27,29^, from traumatic joint injury^29,35,55^, by arthroscopy for unspecified indication^28^, or from normal appearing areas of synovium within an OA joint^30^. Additionally, the confound of time from event to tissue acquisition was not described for these studies. To our knowledge, scRNA-seq of control healthy synovial cells has also not been published. Ungethuem et al.^27^, who performed bulk RNA gene expression profiling and validation in OA, RA and non-disease synovial tissues derived from fatal accidents, identified 262 significantly differentially up-regulated genes in OA compared to non-disease synovial tissues (their Supplementary Tables S1A,B). A total of 34 of these 262 up-regulated genes were identified as differentially and highly expressed in the *HLA-DR*^+^ clusters of our study, including *AIF1*, *FLOR2*, *HLA*-*DMA*, *HLA*-*DMB* and *HLA-DQB1*. However, several well-known OA associated proinflammatory cytokines expressed in the HLA-DR^+^  clusters, such as *TNF* and *IL1B*, were down-regulated in OA and RA synovial tissues in Ungethuem’s study relative to normal donors (from fatal trauma). Such genes may be highly induced by an acute inflammatory response to trauma or post-mortem, indicating the limitations and challenges to acquiring non-disease controls in gene expression studies of synovial tissues.

Enzymatic isolation may alter the metabolic activity and growth rate of chondrocytes. Our protocol for chondrocyte isolation was designed to minimize cell stress, maximize cell viability and retain native cell phenotypes; our protocol succeeded in yielding 90% viable cells and a cell yield of 7.60 ± 0.92 × 10^5^/g wet weight of cartilage. However, it is possible that longer digestion periods would favor higher cell yields and reveal additional cell phenotypes or proportions of cells of the different phenotypes than identified with the shorter enzymatic digestion protocol. We do not believe this is a major limitation since Hayman 2006 et al.^56^ showed that the cell yield of a short pronase/collagenase protocol for 4.5 h (our protocol was identical but for a total of 3 h) was the same as the yield from the digestion of cartilage with collagenase for 22 h from a standardized 6-mm-diameter punch-biopsy of cartilage.

Steroid injection is a standard treatment modality for painful OA of the knee. While this anti-inflammatory treatment reduces pain, it does not prevent disease progression and recent data suggest that it may even exacerbate it^57^. This suggests that a more precise strategy would be needed for disease course modification. Our insights could open up new avenues for therapeutic intervention, including potential disease modifying approaches, such as the use of targeted biologics vs. the more common approach of pain modulation based on patient reported outcomes. Senescent cells have a proinflammatory phenotype^58^ and their presence was recently reported to increase in knee OA patients^59^. Senolysis (i.e. the therapeutic elimination of senescent cells), therefore, is an appealing precision strategy for the treatment of OA supported by preclinical model efficacy^60^. This strategy has shown promise in a murine model of OA and senolytic agents are in development for OA in humans^61^. Based upon a phase I study, a senolytic inhibitor of the MDM2/p53 protein–protein interaction (UBX0101) was well-tolerated and demonstrated a dose-dependent reduction of knee OA pain following a single IA injection. It will be of great interest in future studies to correlate senescence markers with the various synovial and chondrocyte cell types to determine the precise cell phenotypes targeted by senolytic strategies. Other disease modifying strategies are currently under investigation in clinical trials (ADAMTS-5 inhibitor (GLP1972), a Wnt inhibitor (SM04690), and the growth factor, FGF-18 (Sprifermin)), but none of them are believed to be targeting a specific cellular subset.

In summary, using a methodology that provides a characterization of cartilage and synovium proximal to the disease process and without cell culture, we identified 7 major states (cell populations) and 12 major cell clusters in human OA cartilages and synovia, respectively. Putative upstream cytokines (n = 31) and growth factors (n = 30) were linked to phenotypic changes of OA chondrocytes. Among these upstream regulators, 12 cytokines and 7 growth factors were expressed by synoviocytes and detected in synovial fluid, with no expression detected in chondrocytes. Based on our data, immune subsets including macrophages, dendritic cells, mast cells and T cells, activated fibroblasts, and smooth muscle cells likely contribute to OA disease etiology and progression through expression of proinflammatory cytokines and growth factors, such *as IL1A*, *IL1B*, *TNF*, *IL6* and *IGF1*. In particular, key OA inflammatory mediators, such as *TNF*, *IL1B* and *IL6*, were mainly produced and released to the joint space by *HLA-DRA*^+^ synoviocytes (macrophages and DC), but not chondrocytes. These results provide a unique perspective on OA that suggests that synovial cell populations play a major role in the pathogenesis of cartilage degradation of OA. Thus, this scRNA-seq study has identified the origin of key pathogenic catabolic and anabolic mediators in OA that could allow more focused tissue specific targeting of pathogenic cells and molecules in strategies to treat OA.

## Methods

### Acquisition and processing of synovial and cartilage tissue and synovial fluid

Matched articular cartilage, synovial membrane and synovial fluid biospecimens were acquired as anonymized surgical waste from 22 patients, mean age 69.1 (SD 7.0) years, 72% (n = 16) female, mean BMI 29.3 (SD 5.9) kg/m^2^, all undergoing total knee replacement for medial compartment dominant knee OA. Joint tissues (cartilage and synovial membrane) from 3 OA patients of mean age 67.7 (SD 2.31) years, 2 female, BMI 39 (SD 4.6) kg/m^2^ were randomly selected, from the total of 22, for scRNA-seq analysis. Use of samples from 22 patients were as follows: 3 were used for scRNAseq and an additional 9 patients’ samples were used for synovial fluid analyses; 5 of these additional 9 patients’ cartilage and synovial membrane specimens were used for confirmatory immunofluorescence; samples from the remaining 10 patients were utilized for cartilage RNA isolation for qPCR validation of gene expression. The study protocol was approved by the Institutional Review Board of Duke University and conformed to the relevant ethical guidelines and regulations. For acquisition and use of biospecimens other than the anonymized surgical waste tissue, which was exempt humans subjects research, Informed consent was obtained from all subjects.

We harvested the outer lateral (intact) and inner medial (damaged) tibial articular cartilages for isolation of cells or extraction of cartilage matrix. The anatomic orientation was indicated on the freshly isolated specimens by marker pen to ensure consistency of sampling at prespecified regions of interest. All joint specimens were processed within 2 h of procurement (time of surgery for joint replacement). For chondrocyte dissociation, the tibial plateaus were washed three times with sterile phosphate buffered saline (PBS) (Thermo scientific, IL). Cartilage (~ 1 g) from intact and damaged regions of interest was minced finely with a sterile scalpel and collected in a 15 ml tube. Adjacent sections and matched synovial tissue were preserved for histological evaluation of OA severity and for immunofluorescence staining. A two-step procedure for cell isolation from cartilage was used, consisting of an initial tissue digestion with pronase, followed by collagenase. A total of 15 mls of 1% Pronase (Sigma-Aldrich, MO) made up in DMEM/F12 (Thermo scientific, IL) and sterile filtered with a 0.22 μm filter (Sigma-Aldrich, MO), was added to minced cartilage tissue, incubated for 30 min at 37 °C rotating at 250 revolutions/mins (rpm), and then washed twice with PBS. A total of 15 mls of 0.4% Collagenase type II from Clostridium histolyticum (Sigma-Aldrich, Mo) made up in DMEM/F12 and sterile filtered with a 0.22 μm filter, was added to the cartilage residua (after pronase treatment), incubated for 150 min at 37 °C rotating at 250 rpm. After enzymatic digestion, cells were pelleted by centrifugation, washed, sieved through a 30 μm cell strainer (Miltenyi Biotec, CA), pelleted by centrifugation again and resuspended in 1 ml PBS. Synovial membranes (~ 1 g) were dissected into small pieces, digested with 0.1% Collagenease Type IV from Clostridium histolyticum (Sigma-Aldrich, MO) in 50 mls DMEM/F12 for 1 h at 37 °C rotating at 250 rpm, then washed twice with PBS. After lysing red blood cells with 5 mls ACK lysis buffer (Thermo scientific, IL), cells were centrifuged, washed, and sieved through a 30 μm cell strainer (Miltenyi Biotec, CA), pelleted by centrifugation again and resuspended in 1 ml PBS.

### RNA-seq library preparation and sequencing

Cells were prepared for cDNA amplification and chromium library construction using single-cell RNA-Seq library kit v2 according to the manufacturers’ recommendations (10 × Genomics, CA) by the Molecular Genomics Core facility at the Duke Molecular Physiology Institute (DMPI). cDNA libraries were sequenced on the Illumina HiSeq 4,000 with read length of 150 bp by the Duke Center for Genomic and Computational Biology (GCB) core facility.

### Analysis of scRNA-seq data

The primary analysis of the scRNA-seq data followed the recommended protocols from 10X Genomics. Briefly, we demultiplexed raw base call (BCL) files generated by Illumina sequencers into FASTQ files, upon which alignment was carried out to the h19 human transcriptome; filtering, barcode counting, and UMI counting were performed using Cell Ranger software version 3.1 and default parameters (10X Genomics). The secondary statistical analysis was performed using the R package Seurat^62^, which performed quality control and subsequent analyses on the feature-barcode matrices produced by Cell Ranger. In Seurat, data were first normalized and scaled after basic filtering for minimum gene and cell observance frequency cut-offs^63^. We then examined the data and performed further filtering (nFeature 7,500, nCount 100,000, percent_mt 0.3) based on a range of metrics in an attempt to identify and exclude possible multiplets (i.e. instances where more than one cell was present and sequenced in a single emulsified gel bead). The removal of further technical artifacts was performed using regression methods to reduce noise^63^.

After quality control procedures were complete, we performed linear dimensional reduction, calculating principal components using the most variably expressed genes in our dataset^63^. The genes underlying the resulting principal components were examined to confirm they were not enriched in genes involved in cell division or other standard cellular processes^64^. Significant principal components for downstream analyses were determined through methods mirroring those implemented by Macosko et al^64^, and these principal components were carried forward for two main purposes: to perform cell clustering and to enhance visualization^63^. Cells were grouped into an optimal number of clusters for de novo cell type discovery using Seurat’s FindNeighbors and FindClusters functions^63^; graph-based clustering approaches with visualization of cells was achieved through the use of manifold learning technique UMAP (Uniform Manifold Approximation and Projection)^65^, which reduces the information captured in the selected significant principal components to two dimensions^63^. In total, 14,613, 11,579 and 10,640 cells from intact cartilage, damaged cartilage and synovium, respectively, were used for downstream analyses. Differentially expressed genes for a particular cell type were defined by a *p* value threshold < 0.05 and log FC > 0.25; all provided *p* values were adjusted by Bonferroni correction.

Our analysis revealed 12 distinct cell types with a total of fifteen clusters visualized using UMAP in synovial tissues (Supplementary Fig. S1). Clusters 0, 1, 2, 4, 5, 6, 7 and 8, could be further divided into 2 distinct subtypes, SSF and SIF (Supplementary Fig. S1a,b): We also observed four immune subpopulations (clusters 3, 9, 12 and 13) that all expressed *PTPRC* (*CD45*) (Supplementary Fig. S1l), and three unique subpopulations, clusters 10, 11 and 14, that expressed specific markers of known cell types, including EC (*PLVAP*), SMC (*ACTA2*) and ProIC (*HIST1H4C*, *BIRC5*, and *CCL3*), respectively. T cells in cluster 12 and mast cells in cluster 13 highly expressed *CD3* and *TPSAP1*, respectively. Proliferating immune cells (ProIC) in cluster 14, expressing genes related to immune cell markers (*HLA-DRA* and *PTPRC*), were also characterized by high differential expression of genes responsible for regulation of cell division, the cell cycle, chromatin structure regulation and apoptosis inhibition (namely CENPF, CDK1, HIST1H4C and BIRC5). Overall, among these 15 clusters, there were eight distinct cell populations observed from each of three patients, including SSF, SIF, *HLA-DRA*^+^ cells, EC, SMC, T cells, mast cells, ProIC; the *HLA-DRA*^+^ subtype could be further subdivided into 5 cell types for a total of 12 distinct cell types.

The methods for upstream mediator analysis, Trajectory Analysis, qRT-PCR for validation of gene expression in cartilage and immunofluorescence staining for detection of cytokine expression are described in Supplementary Information (Supplementary Methods).

## Supplementary information

Supplementary file1

## Data Availability

There are no restrictions on the availability of materials or information once the manuscript is published. All RNAseq data are available at NCBI GEO GSE152805.
