# Supplementary material for: Synovial cell cross-talk with cartilage plays a major role in the pathogenesis of osteoarthritis
Source: Sci Rep. 2020 Jul 2;10:10868. doi: 10.1038/s41598-020-67730-y (PMC7331607; doi:10.1038/s41598-020-67730-y)
Supplement: Supplementary file 1 — Supplementary file1 [file 41598_2020_67730_MOESM1_ESM.pdf]

SUPPLEMENTARY INFORMATION

Synovial and Cartilage Cell Cross-Talk in Osteoarthritis Pathogenesis Based on Single Cell RNA Sequencing

Ching-Heng Chou, Vaibhav Jain, Jason Gibson, David E. Attarian, Collin A. Haraden, Christopher B. Yohn, Remi-Martin Laberge, Simon Gregory, Virginia B. Kraus

Supplementary Methods

Upstream mediator analysis

Ingenuity Pathway Analysis (IPA, Qiagen, German) was performed to determine the biological functions, processes and diseases and potential upstream mediators associated with the highly expressed genes of HomC (190 genes input), HTC (188 genes input), preHTC (141 genes input), RegC (48 genes input), PreFC (157 genes input), FC (339 genes input) and RepC (61 genes input). Potential upstream cytokine and growth factor regulators of the chondrocyte genes were identified based on prior knowledge stored in the IPA database of expected effects between transcriptional regulators and their target genes. The activation z-score (determined by IPA representing the likelihood of the predicted transcriptional regulator to activate the downstream gene expression) and overlap p-value (calculated using Fisher's Exact Test for testing whether there is a statistically significant overlap between the dataset genes and the genes that are regulated by a transcriptional regulator) were then computed for each potential upstream regulator to infer the activation state of the predicted regulator and the statistically significant overlap between the dataset genes and highly expressed genes under regulation. For the clustering analysis of scRNA-seq data from intact and damaged cartilage, the alignment strategy of Butler et al.[51] was utilized to promote the identification of common cell types and enable comparative analyses.

Trajectory Analysis

In order to clearly understand the transition between different chondrocyte subtypes in response to disease progression, we performed trajectory analysis on all chondrocyte clusters identified in the clustering and differential expression analysis using the R package Monocle2. The underlying algorithm, 'DDRTree', uses reversed graph embedding to describe multiple fate decisions in a

fully unsupervised manner[52]. Data were imported from the existing Seurat object used in clustering and differential expression analysis. Basic filtering for minimum gene and cell observance frequency cut-offs was then performed as well as size and dispersion estimates on count data. We reduced dimensionality by performing a Principle Component Analysis (PCA) followed by t-SNE to project cells into two dimensions[52]. Density peak clustering, based on each cell's local density (P) and the nearest distance (Δ) of a cell to another cell with higher distance, identifies cell clusters in 2-D t-SNE space[52]. We performed differential gene expression testing as a way to extract the genes that distinguish clusters. We used select top significant genes across all clusters as input for the reversed graph embedding (RGE) algorithm that was used to define progress through the trajectory[52]. We used the RGE algorithm, 'DDRTree', to construct a principal tree on the population of single cells that describes changes in global gene expression as a cell progresses through the biological process under study (pseudotime) as well as identifies branch points that describe significant divergences in cellular state. Genes with significant branch-dependent expression were identified using branch expression analysis modeling (BEAM)[51]. GO pathway enrichment analyses were performed using String Database: (https://string-db.org/) for all differentially expressed (DE) genes of each state. Ingenuity Pathway analysis (Qiagen, German) was conducted to identify upstream regulators of Highly expressed genes from each state.

qRT-PCR for validation of gene expression in cartilage

The processes of cartilage harvest, sectioning, grinding, and extraction were performed as previously described[4]. Regions of interest were sectioned and powdered under liquid nitrogen; ~100 mg of cartilage powder was used for RNA isolation with Trizol (Thermo Scientific, IL). A total of 1000 ng RNA from each sample was converted into cDNA using the iScript cDNA synthesis kit (Bio-rad, CA). To determine expression of upstream regulators, we performed qRT-

PCR for Interleukin 1 alpha (*IL1A*), Interleukin 1 beta (*IL1B*), Interleukin 6 (*IL6*), TNF alpha (*TNF*), and the Glyceraldehyde Phosphate Dehydrogenase (*GAPDH*) housekeeping gene on 20 cDNA samples from 10 matched damaged and non-damaged knee articular cartilages. If gene expression was undetectable, the Ct value of 40 was assigned. cDNA from the C28/I2 immortalized chondrocyte cell line was used as a positive control for every qRT-PCR run. Forward (F) and Reverse (R) primer sequences used to detect expression were: *IL1A-F: TGGTAGTAGCAACCAACGGGA, IL1A-R: ACTTTGATTGAGGGCGTCATTC; IL1B-F: ATGATGGCTTATTACAGTGGCAA, IL1B-R: GTCGGAGATTCGTAGCTGGA; IL6-F: ACTCACCTCTTCAGAACGAATTG, IL6-R: CCATCTTTGGAAGGTTCAGGTTG; TNF-F: CCTCTCTCTAATCAGCCCTCTG, TNF-R: GAGGACCTGGGAGTAGATGAG; GAPDH-F: GAGTCAACGGATTTGGTCGT, GAPDH-R: TTGATTTTGGAGGGATCTCG).*

Immunofluorescence staining for detection of cytokine expression Synovial tissue and full depth cartilage (~2cm length x 5mm width) from 5 OA knees were snap frozen in liquid nitrogen and embedded in optimal cutting temperature compound (VWR, PA); 10 μm and 5 μm sections were prepared from matched cartilage and synovial tissues, respectively, and stored at -80°C. The staining protocol followed the manufacturer's instructions (Abcam, MA). After 4% paraformaldehyde fixation, methanol permeabilization, and 1% bovine serum albumin blocking, sections were incubated at 4°C overnight with primary antibodies. After washing, sections were reacted with secondary antibodies for 1 hour. Slides were then mounted with Vector® trueVIEW™ autofluorescence quenching reagent with DAPI (Vector, CA) and imaged via Zeiss axio imager. Primary antibodies were as follow anti-IL1B (1:100, Cat#12703 from Cell Signaling Technology, MA; 1:100, TA506443S from Thermo scientific, IL), anti-HLA-DQA1(1:100, ab211930, Abcam, MA), anti-HLA-DQA2 (1:300, SAB2501821, Sigma-Aldrich,

Mo), anti-TLR2 (1:50, MA532787, Thermo Scientific, IL), anti-OLR1 (1:300, PA580872, Thermo Scientific, IL), anti-CCL3 (1:50, Cat# 710127, Thermo Scientific, IL), anti-CD163 (1:200, ab156769, Abcam, MA), anti-CD169 (1:50, MABT328, Sigma-Aldrich, Mo), anti-STAB1 (1:100, ab101035, Abcam, MA), anti-IL6 (1:200, PA126811, Thermo Scientific, IL), anti-CD14 (1:25, Cat# 14-0149-82 Thermo Scientific, IL). Secondary antibodies used were as follows: donkey anti-rabbit Alexfluor-488, donkey anti-mouse Alexfluor-488, Donkey anti-goat Alexfluor-568, donkey anti-rabbit Alexfluor-647 and donkey anti-mous Alexfluor-647 (1:500, Thermo scientific, IL)

Quantification of Cytokines and growth factors in OA synovial fluids

Synovial fluid was obtained by direct aspiration from OA knees; the supernatant was collected by centrifugation for 15 minutes at 3000rpm in 4°C, aliquoted and stored immediately at -80°C until analysis. TNF, IL6, IL1A, IL1B, IL15, CCL5 and BDNF in OA synovial fluid samples were quantified using multi-analyte profile (Myriad RBM, TX) as described in our previous study53 (Supplementary Fig. S8a). Protein expression of cytokines in the synovial fluid of the three scRNA-seq cases was profiled using the proteome profiler array human XL cytokine array kit (ARY022B, R&D Systems, MN) for TNF, IL6, IL1A, IL1B, CXCL12, IL15, C5, CCL5, TNFSF13B, BDNF, HGF, ANGPT2 and PDGF-BB (PDGFB) (Supplementary Fig. S8b). Synovial fluid protein expression of BMP6 (ARY022B, RayBiotech, GA), FGF10 (LS-F11445, LSBio, WA) and IL18 (L45SA-1, Mesoscale Discovery, MD) was analyzed in 12 OA synovial fluid samples (original 3 plus 9 independent patients) using ELISA-based methods according to the manufacturers' instructions.

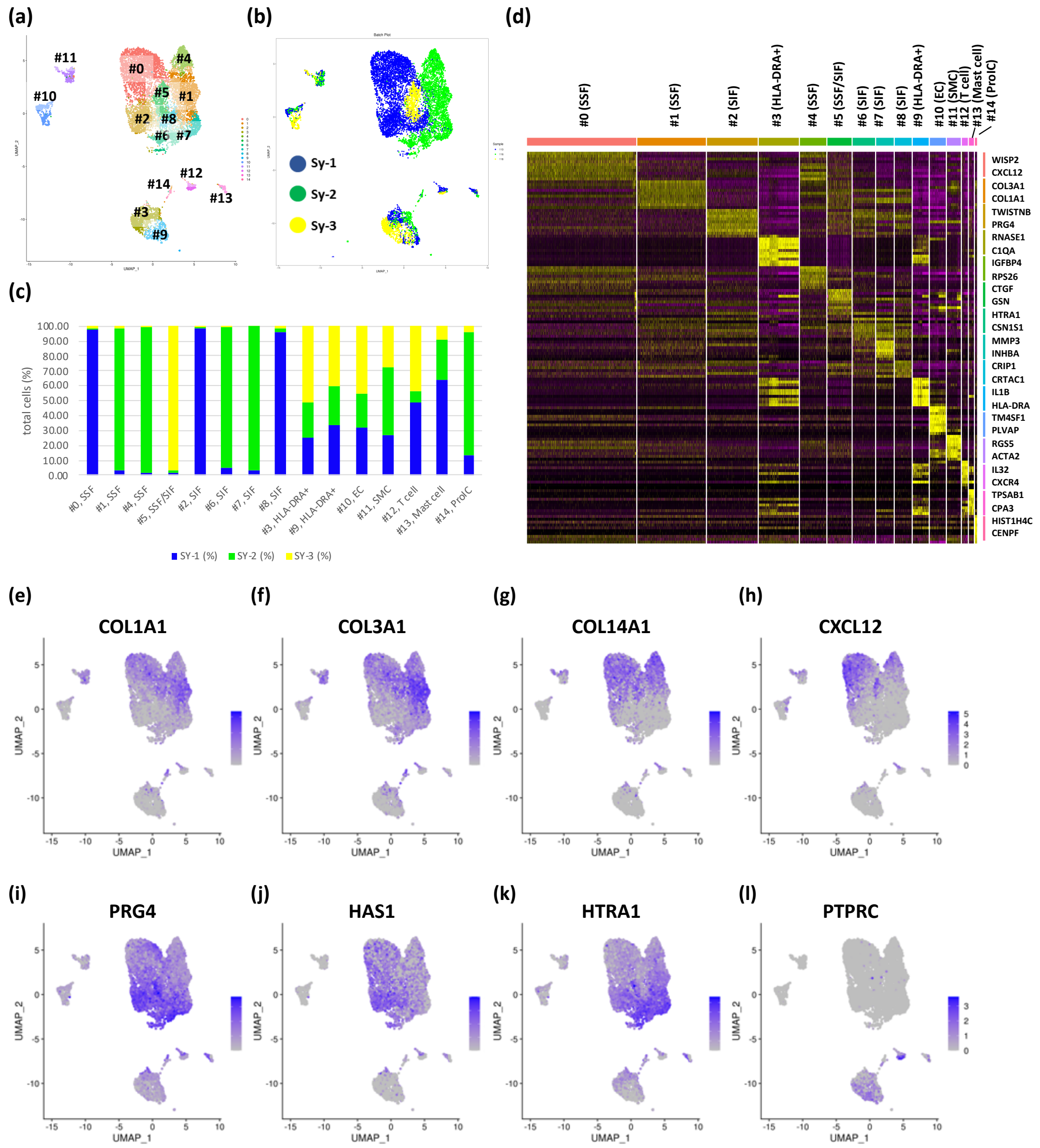

Supplementary Figure S1. Sc-RNA-seq analysis of OA synovial tissues. (a) UMAP plots of scRNA-seq showing the 15 clusters from unsupervised analysis (colored according to putative cell types for human OA synovium) from (b) three human OA synovial tissues, and (c) the bar graphic shows the proportion of acquired cells for each cluster among individuals. (d) Heatmap of unsupervised clustering analysis showing the top ten highly expressed genes per cluster as determined by Seurat analysis with top two genes per cluster highlighted on the right. Expression level is scaled based on z-score distribution. Expression of the selected matrix component

associated highly expressed genes, (e) *COL1A1*, (f) *COL3A1*, (g) *COL14A1* and (h) the stromal cell-derived factor 1 (*CXCL12*) were mainly detected in clusters 0, 1, 4, and half of 5. Expression of genes producing essential constituents of synovial fluid, including (i) lubricin (*PRG4*) and (j) hyaluronan (*HAS1*), and synovial fibroblast serine protease, (k) *HTRA1* were mainly detected in clusters 2, 6, 7, 8 and the other half of 5. (l) Four immune subpopulations with broadly expressed *PTPRC* (CD45, a known leukocyte common antigen) were mainly detected in clusters 3, 9, 12 and 13. SMC, smooth muscle cell; EC, endothelial cell; ProIC, proliferating immune cell; SSF, synovial subintimal fibroblast; SIF, synovial intimal fibroblast; UMAP, uniform manifold approximation and projection. SY-1, -2, -3 represent cells originating from three separate patients.

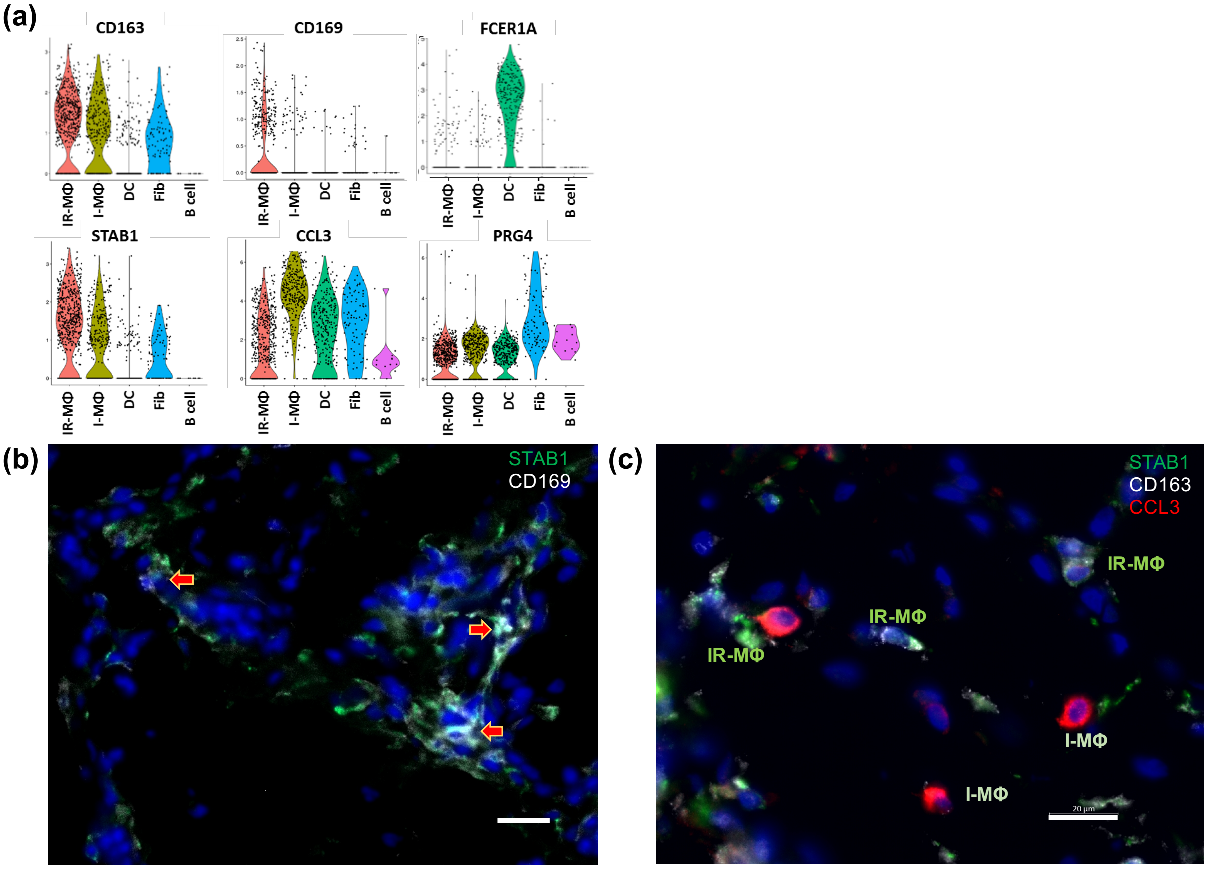

Supplementary Figure S2. Gene expression profiles of HLA-DRA+ synoviocytes. (a) Violin plots showing expression levels of specific marker genes across *HLA-DRA* expressing cells. (b) Representative immunofluorescence staining of human OA synovium demonstrating expression of immune regulatory biomarkers, CD169 and STAB1, by IR-MΦ. (c) Representative immunofluorescence staining of human OA synovium shows CCL3 was highly expressed in I-MΦ ($STAB1^{low}$), but not IR-MΦ ($STAB1^{high}$). DNA was stained using DAPI (blue). Scale bar= 20µm.

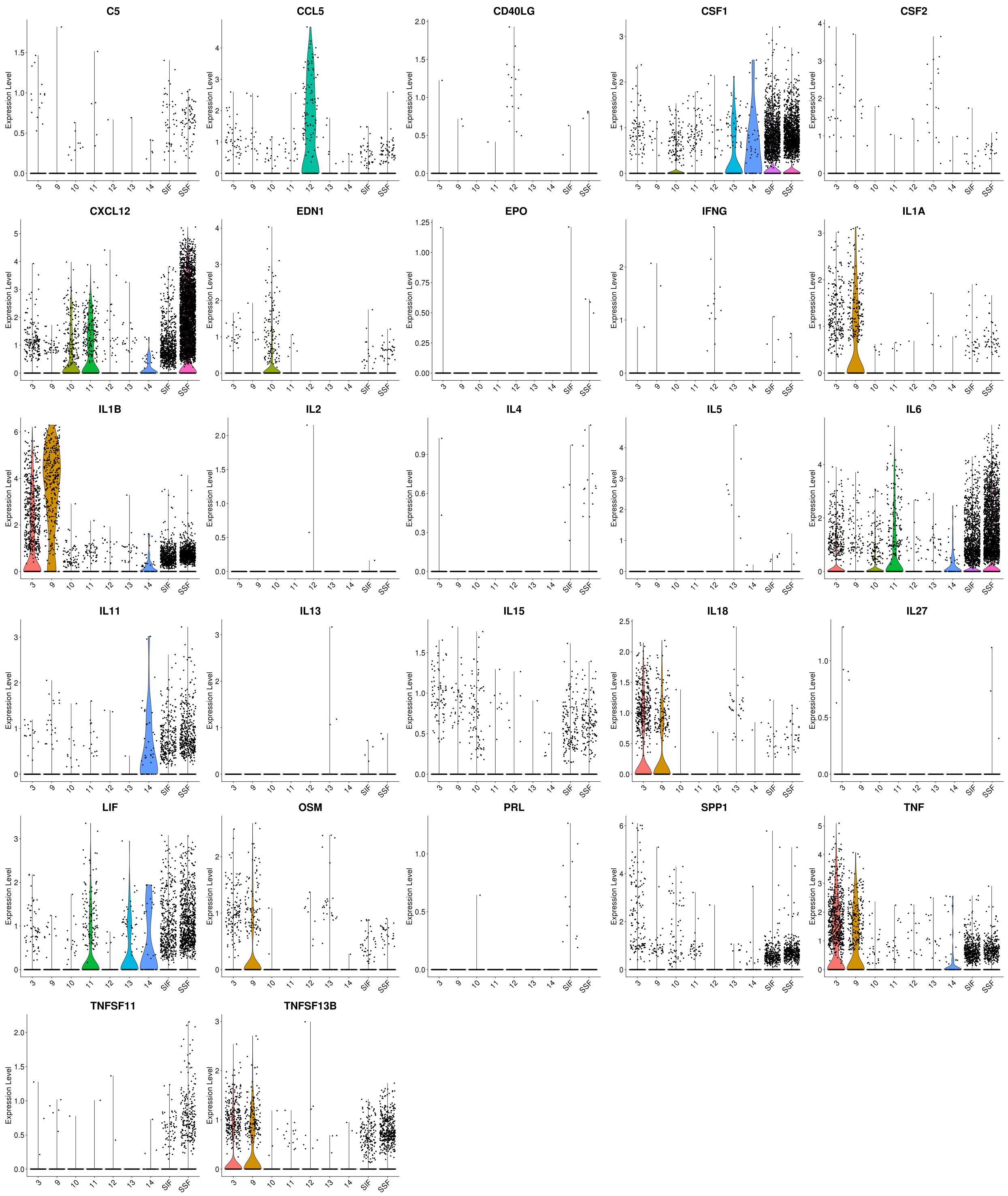

Supplementary Figure S3. Violin plots of synovial cell expression of upstream cytokines predicted to regulate OA chondrocyte phenotypes. 27 of 31 cytokines were detected in OA synovial tissues across all synovial cell clusters; 17 cytokines *(TNF, IL6, IL1B, IL1A, EDN1, OSM,*

CXCL12, IL15, IL18, C5, CCL5, TNFSF13B, TNFSF11, CSF1, LIF, SPP1 and IL11) were expressed in >1% of synoviocytes. Clusters left to right: #3, *HLA-DRA+* cells; #9, *HLA-DRA*+ cells; #10, SMC; #11, EC; #12, T cells; #13, mast cells; #14, ProIC, synovial intimal, and subintimal fibroblast. 4 predicted upstream cytokines (*IFNA2*, *IL17A*, *IL3* and *WNT1*) were not detected in synovial tissues.

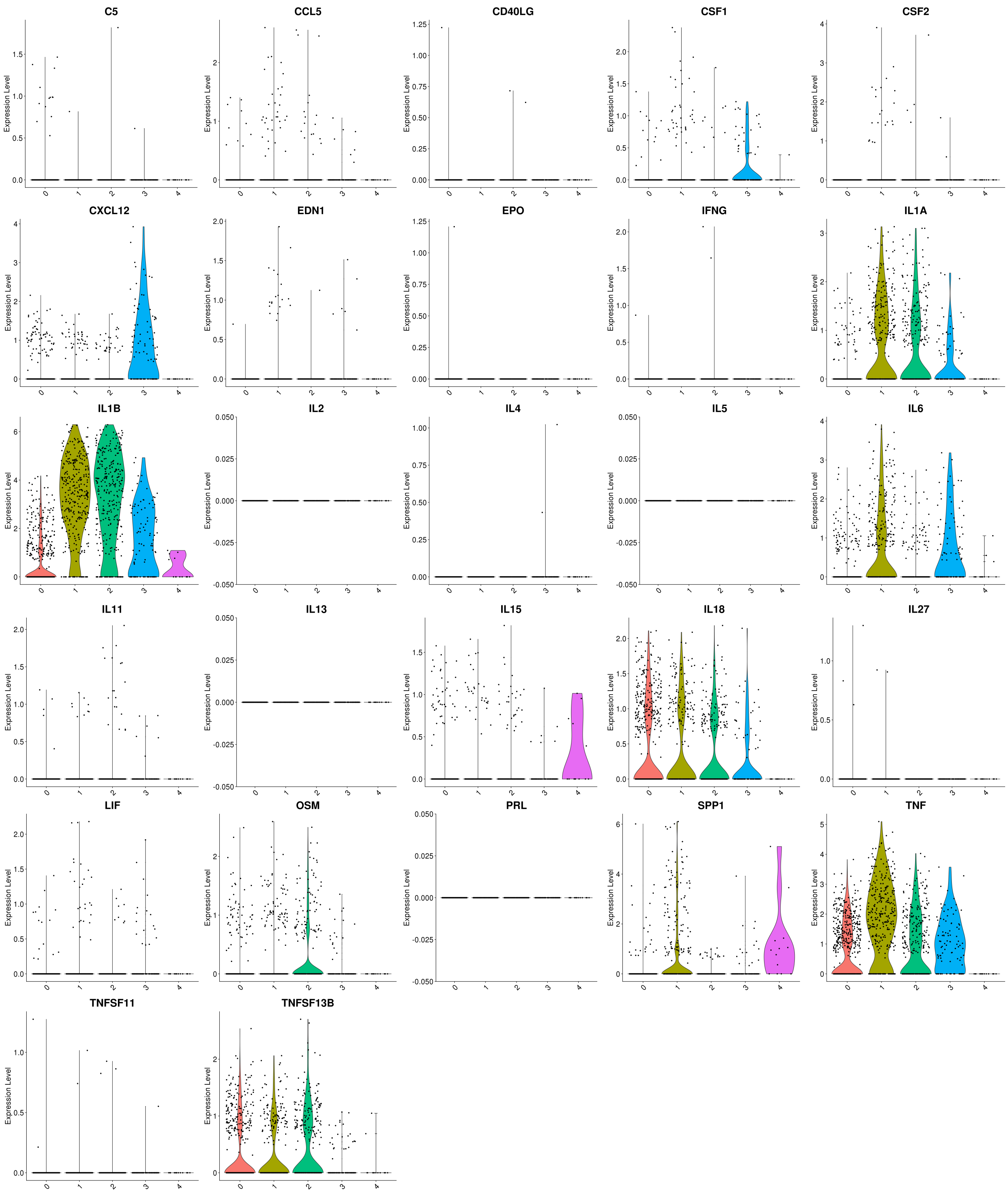

Supplementary Figure S4. Violin plots of synovial *HLA-DRA*+ cell clusters expressing upstream cytokines predicted to regulate OA chondrocyte phenotypes. 27 expressing upstream cytokines were plotted across all synovial *HLA-DRA*$^{+}$ cell clusters (clusters left to right):

#0 IR-MΦ, #1, I-MΦ, #2, DC, #3, FIB, #4, B cells). 4 predicted upstream cytokines (*IFNA2, IL17A, IL3* and *WNT1*) are not expressed in *HLA-DRA*$^+$ cells.

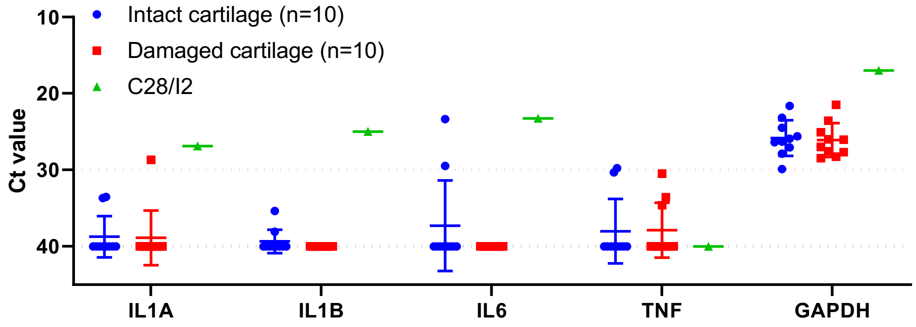

Supplementary Figure S5. General lack of cytokine expression by chondrocytes. Top predicted upstream cytokines regulators of the OA chondrocyte phenotypes, *IL1A, IL1B, IL6* and *TNF,* were not detected or detected at low levels in bulk mRNA from either human intact or damaged OA cartilage. The immortalized C28/I2 chondrocyte cell line was used as a positive control. *GAPDH* was used as a housekeeping control.

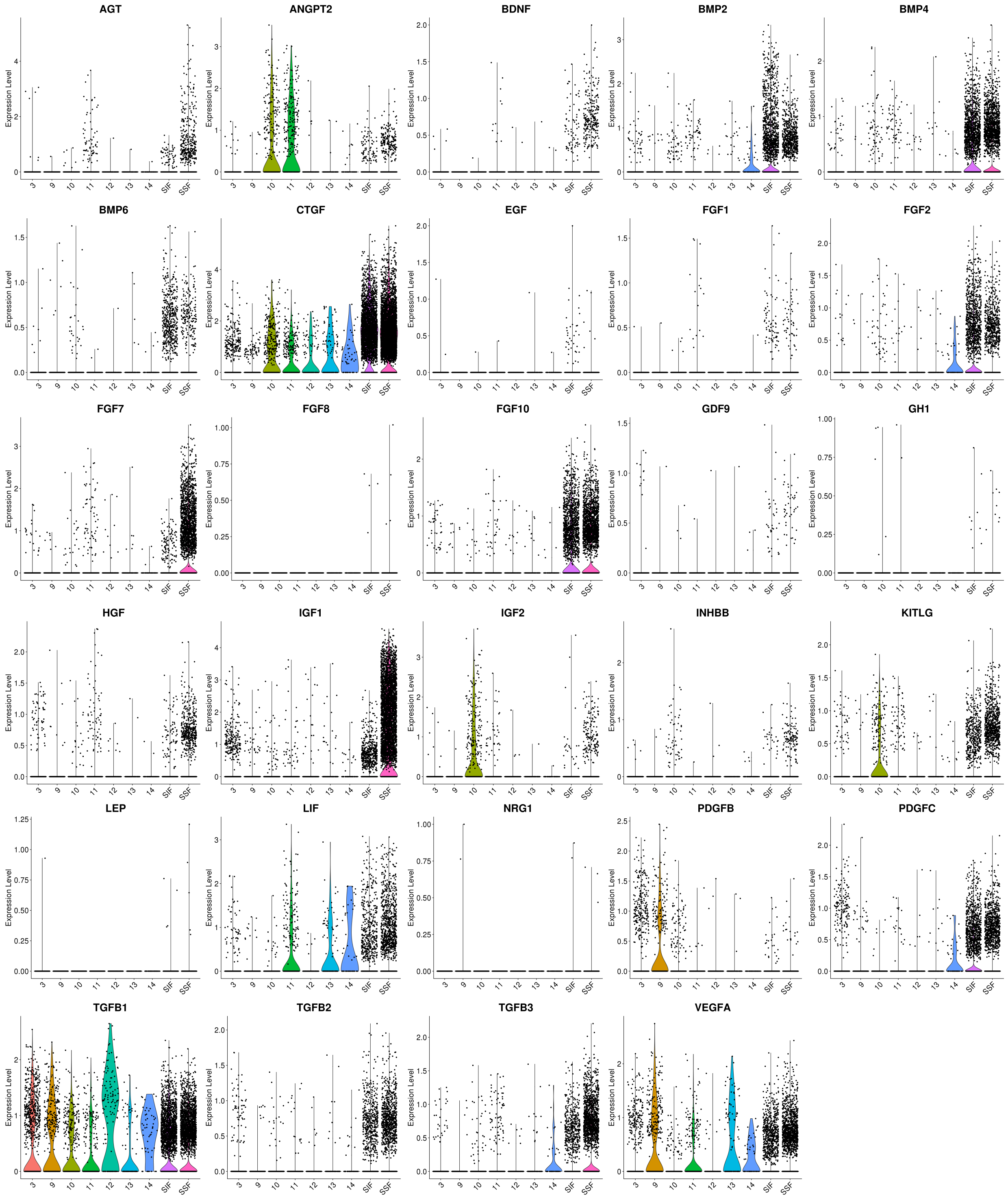

Supplementary Figure S6. Violin plots of synovial cell expression of upstream growth factors predicted to regulate OA chondrocyte phenotypes. 29 of 30 growth factors were detected in OA synovial tissues across all synovial cell clusters; 23 growth factors (*IGF1, HGF, ANGP2,*

PDGFB, FGF10, INHBB, BMP6, BDNF, TGFB1, TGFB2, TGFB3, AGT, FGF2, FGF7, BMP2, BMP4, VEGFA, CTGF, KITLG, NGF, PDGFC, IGF2, FGF1) were expressed in >1% of synoviocytes. Clusters left to right): #3, HLA-DR+ cells; #9, HLA-DR+ cells; #10, SMC; #11 EC, #12 T cells, #13 mast cell, #14 ProIC, synovial intimal and subintimal fibroblast). Predicted upstream growth factor, GDF2, was not expressed in synovial tissues.

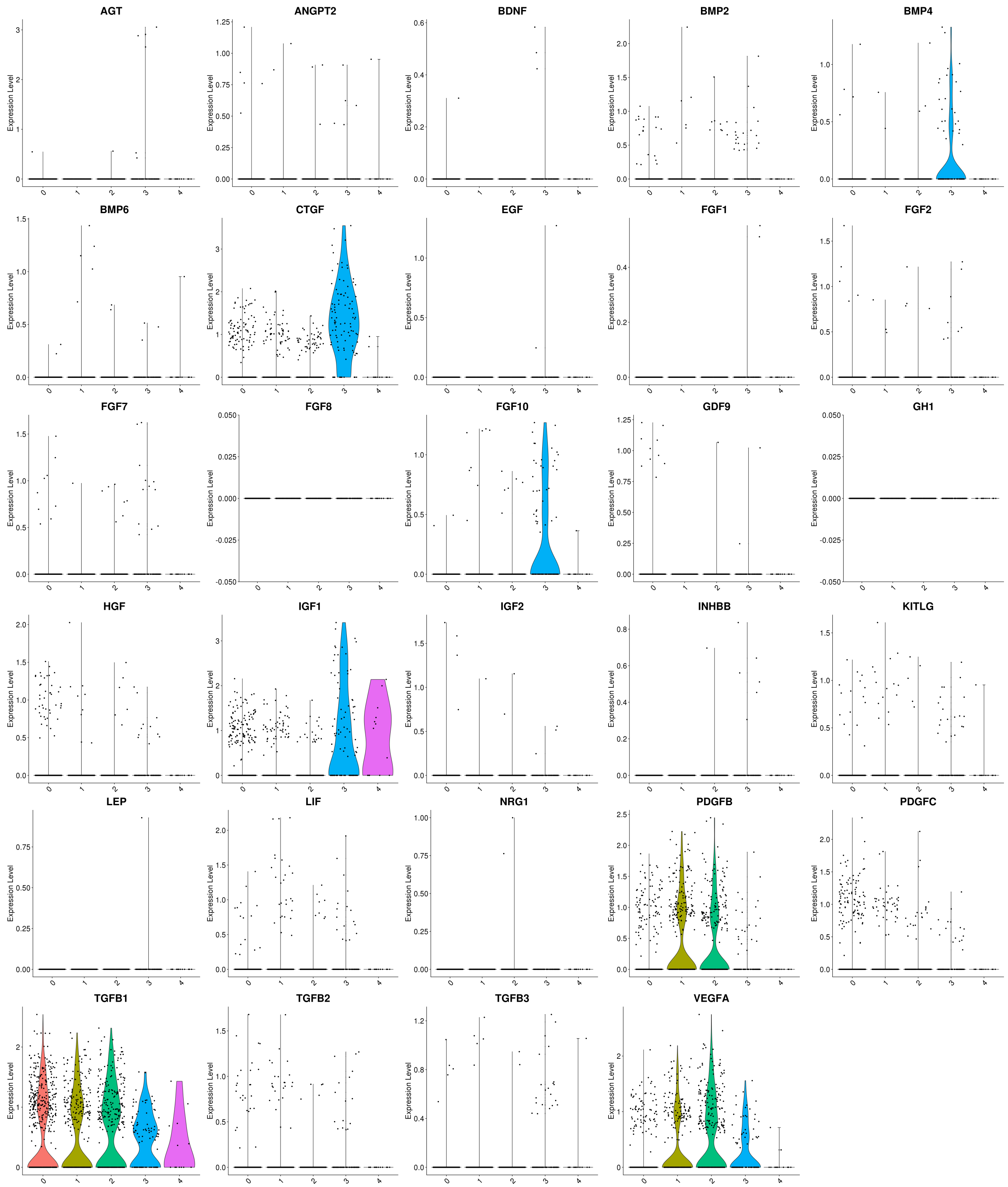

Supplementary Figure S7. Violin plots of synovial *HLA-DRA*+ cell clusters expressing upstream growth factors predicted to regulate OA chondrocyte phenotypes. 29 of 30 growth factors were plotted across all synovial *HLA-DRA*$^{+}$ cell clusters (clusters left to right): #0 IR-MΦ,

#1, I-MΦ, #2, DC, #3, FIB, #4, B cells). Predicted upstream growth factor, GDF2, is not expressed in *HLA-DRA*$^{+}$ cells.

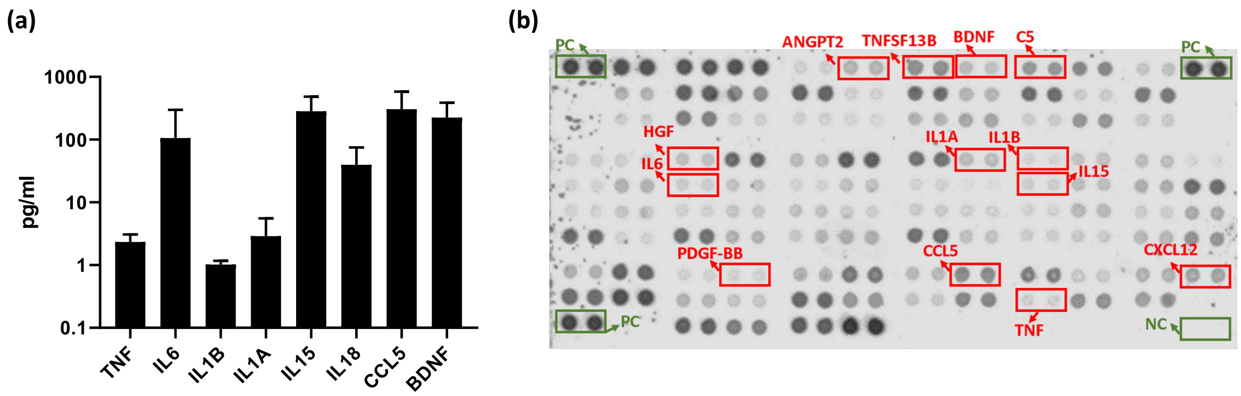

Supplementary Figure S8. Identification of regulatory cytokines in OA synovial fluid. (a) Predicted upstream regulators TNF, IL6, IL1A, IL1B, IL15, CCL5 and BDNF in OA synovial fluid samples were quantified using multi-analyte profile (Myriad RBM, TX) as described in our previous study[31]. (b) Protein expression of cytokines in the synovial fluid of the three scRNA-seq cases was profiled using the proteome profiler array human XL cytokine array kit (ARY022B, R&D Systems, MN) for TNF, IL6, IL1A, IL1B, CXCL12, IL15, C5, CCL5, TNFSF13B, BDNF, HGF, ANGPT2 and PDGF-BB (PDGFB).

Supplementary Table S1A. List of differentially expressed genes in osteoarthritic synoviocytes.

Gene	Putative cell type of the cluster	The percentage of cells expressing the gene in the cluster	The percentage of cells expressing the gene in the other clusters	Log fold-change of the average expression between the two groups	Adjusted p-value (Bonferroni correction)
RNASE1	HLA-DR+-1	98.40%	19.30%	3.21	0.00E+00
C1QA	HLA-DR+-1	98.80%	14.10%	3.21	0.00E+00
C1QB	HLA-DR+-1	97.40%	12.40%	2.99	0.00E+00
C1QC	HLA-DR+-1	97.10%	8.00%	2.85	0.00E+00
CCL4	HLA-DR+-1	70.90%	31.10%	2.79	0.00E+00
MARCO	HLA-DR+-1	90.80%	8.00%	2.65	0.00E+00
TYROBP	HLA-DR+-1	99.60%	14.20%	2.63	0.00E+00
CCL3	HLA-DR+-1	75.90%	38.40%	2.60	0.00E+00
AIF1	HLA-DR+-1	97.90%	9.40%	2.47	0.00E+00
FOLR2	HLA-DR+-1	90.90%	5.70%	2.35	0.00E+00
CD74	HLA-DR+-1	99.20%	45.50%	2.28	0.00E+00
FCER1G	HLA-DR+-1	98.40%	13.10%	2.24	0.00E+00
CTSZ	HLA-DR+-1	95.70%	40.50%	2.08	0.00E+00
HLA-DRB1	HLA-DR+-1	94.60%	25.20%	2.03	0.00E+00
LAPTM5	HLA-DR+-1	97.00%	14.80%	2.00	0.00E+00
HLA-DRA	HLA-DR+-1	97.80%	34.50%	1.90	0.00E+00
CD14	HLA-DR+-1	92.50%	27.80%	1.90	0.00E+00
MS4A6A	HLA-DR+-1	92.20%	7.00%	1.87	0.00E+00
LYZ	HLA-DR+-1	90.90%	8.60%	1.87	0.00E+00
CTSS	HLA-DR+-1	95.50%	25.90%	1.85	0.00E+00
SEPP1	HLA-DR+-1	92.80%	71.20%	1.85	0.00E+00
FTL	HLA-DR+-1	100.00%	99.80%	1.84	0.00E+00
LYVE1	HLA-DR+-1	66.80%	5.00%	1.84	0.00E+00
VAMP8	HLA-DR+-1	92.90%	11.80%	1.80	0.00E+00
LGMN	HLA-DR+-1	85.30%	40.10%	1.78	0.00E+00
VSIG4	HLA-DR+-1	85.90%	5.80%	1.76	0.00E+00
HLA-DPA1	HLA-DR+-1	92.50%	25.00%	1.72	0.00E+00
CYBB	HLA-DR+-1	83.90%	3.70%	1.67	0.00E+00
TNF	HLA-DR+-1	62.50%	11.50%	1.66	0.00E+00
HLA-DRB5	HLA-DR+-1	91.60%	13.20%	1.63	0.00E+00
HLA-DPB1	HLA-DR+-1	90.90%	35.10%	1.63	0.00E+00

F13A1	HLA-DR+-1	63.10%	3.60%	1.60	0.00E+00
ALOX5AP	HLA-DR+-1	83.40%	7.70%	1.59	0.00E+00
HLA-DMA	HLA-DR+-1	85.70%	14.80%	1.57	0.00E+00
NCF1	HLA-DR+-1	68.40%	3.80%	1.52	0.00E+00
STAB1	HLA-DR+-1	74.00%	5.70%	1.49	0.00E+00
CTSB	HLA-DR+-1	98.00%	79.60%	1.46	0.00E+00
CD83	HLA-DR+-1	80.80%	21.10%	1.42	0.00E+00
CD163	HLA-DR+-1	77.30%	3.30%	1.41	0.00E+00
CYBA	HLA-DR+-1	97.80%	76.00%	1.40	0.00E+00
FCGR2A	HLA-DR+-1	81.20%	6.80%	1.38	0.00E+00
RGS1	HLA-DR+-1	69.10%	11.70%	1.37	0.00E+00
MSR1	HLA-DR+-1	75.40%	3.10%	1.36	0.00E+00
MS4A7	HLA-DR+-1	73.90%	3.10%	1.34	0.00E+00
FCGR3A	HLA-DR+-1	54.80%	3.00%	1.30	0.00E+00
CD68	HLA-DR+-1	94.00%	57.80%	1.29	0.00E+00
EMB	HLA-DR+-1	68.30%	3.40%	1.28	0.00E+00
CTSD	HLA-DR+-1	93.90%	79.10%	1.26	0.00E+00
GMFG	HLA-DR+-1	79.00%	10.10%	1.24	0.00E+00
FCGR2B	HLA-DR+-1	66.60%	4.70%	1.23	0.00E+00
MAFB	HLA-DR+-1	84.90%	39.20%	1.23	0.00E+00
HLA-DMB	HLA-DR+-1	69.20%	5.70%	1.22	0.00E+00
ACP5	HLA-DR+-1	62.90%	6.90%	1.21	0.00E+00
HLA-DQB1	HLA-DR+-1	74.60%	10.50%	1.21	0.00E+00
SPI1	HLA-DR+-1	63.00%	2.80%	1.20	0.00E+00
GPX1	HLA-DR+-1	97.30%	82.60%	1.19	0.00E+00
DUSP1	HLA-DR+-1	84.20%	53.90%	1.17	0.00E+00
MS4A4A	HLA-DR+-1	62.40%	2.40%	1.16	0.00E+00
FTH1	HLA-DR+-1	100.00%	100.00%	1.15	0.00E+00
SLC40A1	HLA-DR+-1	55.20%	11.20%	1.15	0.00E+00
LST1	HLA-DR+-1	73.20%	5.60%	1.13	0.00E+00
PLEK	HLA-DR+-1	62.50%	6.30%	1.11	0.00E+00
LILRB5	HLA-DR+-1	62.60%	1.30%	1.10	0.00E+00
S100A9	HLA-DR+-1	41.50%	2.10%	1.09	0.00E+00
ARHGDIB	HLA-DR+-1	80.50%	15.60%	1.08	0.00E+00
CSF1R	HLA-DR+-1	61.50%	2.80%	1.08	0.00E+00
ITGB2	HLA-DR+-1	69.20%	5.40%	1.08	0.00E+00
FPR3	HLA-DR+-1	61.80%	2.60%	1.06	0.00E+00
ARHGAP18	HLA-DR+-1	69.60%	12.10%	1.06	0.00E+00
YBX1	HLA-DR+-1	94.50%	80.80%	1.06	0.00E+00
CSTB	HLA-DR+-1	93.00%	81.10%	1.04	0.00E+00
CPVL	HLA-DR+-1	66.40%	6.80%	1.04	0.00E+00

HLA-DQA1	HLA-DR+-1	59.70%	7.70%	1.04	0.00E+00
BLVRB	HLA-DR+-1	79.00%	48.00%	1.03	0.00E+00
PYCARD	HLA-DR+-1	74.80%	26.90%	1.01	0.00E+00
NPC2	HLA-DR+-1	99.40%	91.50%	0.99	0.00E+00
SRGN	HLA-DR+-1	91.20%	39.30%	0.99	0.00E+00
GRN	HLA-DR+-1	92.90%	80.40%	0.98	0.00E+00
MAF	HLA-DR+-1	65.40%	24.40%	0.96	0.00E+00
ALDH1A1	HLA-DR+-1	48.50%	3.20%	0.96	0.00E+00
C5AR1	HLA-DR+-1	58.00%	5.40%	0.95	0.00E+00
HNMT	HLA-DR+-1	65.50%	17.60%	0.94	0.00E+00
HPGDS	HLA-DR+-1	51.40%	3.10%	0.93	0.00E+00
FCGRT	HLA-DR+-1	93.50%	83.10%	0.93	0.00E+00
GPR34	HLA-DR+-1	49.00%	1.10%	0.93	0.00E+00
CREG1	HLA-DR+-1	60.40%	16.40%	0.90	0.00E+00
CD37	HLA-DR+-1	60.70%	5.80%	0.89	0.00E+00
DAB2	HLA-DR+-1	87.70%	70.90%	0.88	0.00E+00
ITGAM	HLA-DR+-1	54.10%	2.50%	0.88	0.00E+00
ARRB2	HLA-DR+-1	60.00%	8.50%	0.88	0.00E+00
DOK2	HLA-DR+-1	53.30%	3.10%	0.87	0.00E+00
KCTD12	HLA-DR+-1	72.00%	32.60%	0.87	0.00E+00
C1orf162	HLA-DR+-1	51.10%	4.10%	0.87	0.00E+00
C3AR1	HLA-DR+-1	53.40%	2.00%	0.86	0.00E+00
RNF130	HLA-DR+-1	82.60%	55.40%	0.86	0.00E+00
ARPC3	HLA-DR+-1	95.80%	86.70%	0.86	0.00E+00
AP1S2	HLA-DR+-1	74.50%	36.70%	0.84	0.00E+00
CD53	HLA-DR+-1	60.00%	5.90%	0.84	0.00E+00
RBM47	HLA-DR+-1	51.50%	3.90%	0.84	0.00E+00
RGS10	HLA-DR+-1	79.70%	48.00%	0.83	0.00E+00
COTL1	HLA-DR+-1	58.60%	13.00%	0.83	0.00E+00
CD4	HLA-DR+-1	60.10%	11.70%	0.83	0.00E+00
RNASE6	HLA-DR+-1	50.10%	3.30%	0.82	0.00E+00
EVI2B	HLA-DR+-1	52.40%	4.30%	0.82	0.00E+00
MNDA	HLA-DR+-1	47.60%	2.40%	0.81	0.00E+00
TMSB10	HLA-DR+-1	100.00%	97.80%	0.80	0.00E+00
PTPRC	HLA-DR+-1	59.70%	5.90%	0.80	0.00E+00
MPEG1	HLA-DR+-1	46.20%	1.60%	0.79	0.00E+00
SLCO2B1	HLA-DR+-1	43.80%	1.70%	0.78	0.00E+00
MFSD1	HLA-DR+-1	69.60%	34.60%	0.78	0.00E+00
FAM49B	HLA-DR+-1	59.70%	15.50%	0.77	0.00E+00
ADAP2	HLA-DR+-1	42.60%	1.80%	0.74	0.00E+00
IGSF6	HLA-DR+-1	41.10%	1.90%	0.73	0.00E+00

TREM2	HLA-DR+-1	39.00%	0.80%	0.72	0.00E+00
AP1B1	HLA-DR+-1	53.20%	13.70%	0.71	0.00E+00
PEPD	HLA-DR+-1	62.30%	40.80%	0.70	0.00E+00
CD84	HLA-DR+-1	44.20%	2.00%	0.69	0.00E+00
ATP6V0B	HLA-DR+-1	88.10%	75.00%	0.69	0.00E+00
LILRB4	HLA-DR+-1	42.50%	1.70%	0.66	0.00E+00
NCF4	HLA-DR+-1	42.20%	2.10%	0.68	1.04E-315
LTC4S	HLA-DR+-1	45.10%	3.60%	0.85	6.72E-315
CTD-2337J16.1	HLA-DR+-1	34.30%	0.50%	0.56	2.68E-314
CEBPB	HLA-DR+-1	68.60%	40.80%	0.85	5.32E-314
TGFBI	HLA-DR+-1	96.50%	61.80%	0.98	8.63E-313
PFN1	HLA-DR+-1	98.80%	95.70%	0.71	1.45E-311
FYB	HLA-DR+-1	48.00%	3.80%	0.72	2.84E-310
FRMD4B	HLA-DR+-1	64.90%	23.80%	0.85	5.69E-310
LCP1	HLA-DR+-1	51.80%	4.90%	0.79	2.63E-308
HCK	HLA-DR+-1	40.80%	1.90%	0.60	9.25E-305
TMSB4X	HLA-DR+-1	100.00%	99.90%	0.56	3.23E-304
NCF2	HLA-DR+-1	41.30%	1.90%	0.63	3.30E-304
GNAI2	HLA-DR+-1	79.20%	53.90%	0.68	1.46E-303
TBXAS1	HLA-DR+-1	44.70%	3.10%	0.66	6.67E-303
CLEC7A	HLA-DR+-1	47.50%	3.70%	0.73	1.50E-300
IL8	HLA-DR+-1	82.50%	66.50%	1.39	1.56E-294
SNX2	HLA-DR+-1	64.80%	43.20%	0.64	1.14E-289
LAMP1	HLA-DR+-1	65.70%	42.40%	0.66	1.82E-289
HSPA1A	HLA-DR+-1	77.40%	66.50%	1.20	9.96E-283
RBPJ	HLA-DR+-1	85.60%	68.90%	0.72	7.02E-280
PILRA	HLA-DR+-1	37.70%	1.90%	0.56	3.82E-279
S100A11	HLA-DR+-1	99.60%	98.70%	0.67	3.10E-278
ASAH1	HLA-DR+-1	68.50%	49.80%	0.65	4.13E-278
IRF8	HLA-DR+-1	49.00%	5.30%	0.71	5.71E-278
PPT1	HLA-DR+-1	71.70%	47.90%	0.66	6.72E-277
ATP5E	HLA-DR+-1	99.40%	98.30%	0.57	3.01E-272
SNCA	HLA-DR+-1	39.10%	2.30%	0.59	3.57E-271
RP11-290F20.3	HLA-DR+-1	32.00%	0.90%	0.54	3.33E-270
RNASET2	HLA-DR+-1	66.80%	28.20%	0.78	1.48E-269
ACTR2	HLA-DR+-1	71.00%	49.40%	0.60	8.19E-269
TNFAIP3	HLA-DR+-1	66.60%	26.00%	1.15	7.16E-266
HCST	HLA-DR+-1	52.00%	6.80%	0.87	1.68E-264
SLC7A7	HLA-DR+-1	38.50%	2.70%	0.55	3.67E-264
CAP1	HLA-DR+-1	81.90%	69.00%	0.60	4.27E-264

CORO1A	HLA-DR+-1	48.70%	5.70%	0.70	6.47E-264
HCLS1	HLA-DR+-1	48.30%	6.30%	0.70	4.58E-263
BLVRA	HLA-DR+-1	53.10%	28.50%	0.59	2.46E-260
TIMD4	HLA-DR+-1	29.00%	0.60%	0.58	2.94E-256
ATP6V1F	HLA-DR+-1	80.70%	75.00%	0.55	5.31E-256
ME1	HLA-DR+-1	37.50%	4.30%	0.60	4.46E-254
CCR1	HLA-DR+-1	31.00%	0.90%	0.50	9.33E-254
MPP1	HLA-DR+-1	46.10%	13.20%	0.59	3.26E-250
UCP2	HLA-DR+-1	53.90%	13.70%	0.74	1.03E-249
CST3	HLA-DR+-1	100.00%	98.90%	0.79	3.42E-249
SNX10	HLA-DR+-1	40.00%	4.00%	0.66	4.78E-249
IL18	HLA-DR+-1	36.80%	2.20%	0.56	3.24E-247
CCDC88A	HLA-DR+-1	56.40%	26.00%	0.66	6.09E-247
IQGAP1	HLA-DR+-1	80.60%	64.10%	0.58	3.28E-243
SH3BGRL	HLA-DR+-1	86.70%	78.40%	0.57	9.97E-242
MAMDC2	HLA-DR+-1	71.00%	42.70%	0.73	1.87E-241
B2M	HLA-DR+-1	100.00%	100.00%	0.53	6.62E-241
LY86	HLA-DR+-1	35.70%	2.00%	0.55	9.64E-241
ARPC5	HLA-DR+-1	84.00%	73.50%	0.58	3.04E-240
MT-CO1	HLA-DR+-1	99.80%	99.50%	0.72	5.75E-240
GPR183	HLA-DR+-1	62.20%	16.10%	1.22	6.54E-240
AP2A2	HLA-DR+-1	48.40%	19.30%	0.58	2.03E-237
TPM3	HLA-DR+-1	87.70%	71.10%	0.64	1.10E-236
TPP1	HLA-DR+-1	57.70%	31.50%	0.58	1.58E-236
HAVCR2	HLA-DR+-1	37.70%	2.70%	0.57	8.07E-236
RHOG	HLA-DR+-1	66.90%	40.30%	0.64	3.85E-235
RENBP	HLA-DR+-1	35.80%	2.70%	0.55	5.43E-231
CXCL3	HLA-DR+-1	73.70%	52.00%	1.46	2.24E-230
LAIR1	HLA-DR+-1	32.10%	1.60%	0.49	3.14E-230
TRA2B	HLA-DR+-1	70.80%	60.30%	0.63	3.24E-230
TALDO1	HLA-DR+-1	72.80%	63.70%	0.51	3.93E-230
ALDH2	HLA-DR+-1	61.10%	37.80%	0.64	5.51E-230
FERMT3	HLA-DR+-1	44.50%	6.70%	0.59	1.26E-229
CPM	HLA-DR+-1	48.90%	7.60%	0.71	1.46E-229
RAC1	HLA-DR+-1	71.30%	54.70%	0.55	7.74E-229
43160	HLA-DR+-1	30.70%	1.30%	0.51	2.97E-227
GPSM3	HLA-DR+-1	42.50%	4.90%	0.58	4.23E-227
KLF6	HLA-DR+-1	90.00%	79.40%	0.81	9.39E-227
IFI30	HLA-DR+-1	39.30%	4.00%	0.78	2.88E-224
ACTB	HLA-DR+-1	100.00%	99.90%	0.61	9.73E-224
MEF2C	HLA-DR+-1	57.90%	18.50%	0.78	2.05E-223

COLEC12	HLA-DR+-1	60.20%	26.00%	0.70	1.07E-222
IQGAP2	HLA-DR+-1	35.30%	2.40%	0.57	1.22E-222
OAZ1	HLA-DR+-1	99.80%	99.10%	0.45	2.28E-222
GLUL	HLA-DR+-1	89.00%	74.90%	0.68	3.22E-220
SIGLEC1	HLA-DR+-1	26.00%	0.70%	0.46	2.19E-216
CLEC2B	HLA-DR+-1	56.20%	12.80%	0.73	5.43E-214
TNS1	HLA-DR+-1	59.60%	38.40%	0.57	1.30E-212
CYTH4	HLA-DR+-1	28.30%	1.10%	0.49	4.53E-212
SH2B3	HLA-DR+-1	47.00%	11.40%	0.61	1.70E-211
LCP2	HLA-DR+-1	36.90%	3.40%	0.53	2.59E-209
ARHGAP30	HLA-DR+-1	34.00%	2.80%	0.54	1.27E-208
SCN9A	HLA-DR+-1	25.10%	0.60%	0.45	3.01E-208
FAM105A	HLA-DR+-1	36.10%	3.80%	0.55	1.06E-207
GATM	HLA-DR+-1	30.60%	1.90%	0.48	1.48E-207
TSPO	HLA-DR+-1	97.20%	92.90%	0.56	1.20E-206
TCN2	HLA-DR+-1	41.30%	15.90%	0.52	2.96E-205
BST2	HLA-DR+-1	70.30%	24.30%	0.71	5.57E-205
HSPA1B	HLA-DR+-1	61.30%	53.20%	0.98	6.98E-205
PIK3AP1	HLA-DR+-1	30.00%	1.60%	0.45	1.42E-204
BRI3	HLA-DR+-1	44.50%	19.70%	0.51	3.36E-204
SNX6	HLA-DR+-1	58.70%	45.50%	0.50	1.09E-202
GLIPR1	HLA-DR+-1	75.60%	47.40%	0.66	1.05E-200
PTPN6	HLA-DR+-1	32.70%	3.10%	0.51	4.37E-200
LYN	HLA-DR+-1	38.30%	7.30%	0.50	2.58E-197
PABPC4	HLA-DR+-1	51.40%	26.80%	0.55	4.65E-197
LY96	HLA-DR+-1	55.00%	34.80%	0.52	1.34E-196
SYNGR2	HLA-DR+-1	48.70%	15.90%	0.59	1.00E-195
CTSC	HLA-DR+-1	48.60%	10.90%	0.68	2.40E-193
PNRC1	HLA-DR+-1	75.40%	56.10%	0.71	1.23E-191
PLEC	HLA-DR+-1	61.70%	42.40%	0.56	1.06E-190
CCL3L1	HLA-DR+-1	34.50%	3.60%	1.18	1.16E-189
GNAS	HLA-DR+-1	82.90%	74.30%	0.51	2.19E-189
LILRB2	HLA-DR+-1	28.80%	1.60%	0.43	2.81E-189
SLC43A2	HLA-DR+-1	40.70%	10.20%	0.55	1.48E-188
LGALS9	HLA-DR+-1	36.30%	5.00%	0.51	1.99E-188
KIAA1598	HLA-DR+-1	34.70%	5.70%	0.51	5.81E-188
CD86	HLA-DR+-1	33.20%	2.90%	0.46	5.93E-186
SYK	HLA-DR+-1	27.20%	1.50%	0.43	3.60E-184
STXBP2	HLA-DR+-1	30.70%	2.30%	0.43	1.49E-183
CPEB4	HLA-DR+-1	54.80%	32.50%	0.56	3.72E-183
VMO1	HLA-DR+-1	31.50%	2.60%	0.55	2.40E-181

SAMHD1	HLA-DR+-1	55.70%	23.30%	0.62	3.14E-180
GAS7	HLA-DR+-1	45.20%	15.80%	0.53	3.55E-180
PDK4	HLA-DR+-1	50.70%	25.40%	0.89	4.55E-180
C4orf48	HLA-DR+-1	33.50%	6.00%	0.56	5.07E-180
PLIN2	HLA-DR+-1	50.80%	29.00%	0.80	5.86E-180
PELI1	HLA-DR+-1	44.60%	25.40%	0.61	1.36E-179
RASGEF1B	HLA-DR+-1	51.90%	19.80%	0.72	8.01E-179
MYO5A	HLA-DR+-1	36.40%	12.60%	0.48	1.17E-178
RCSD1	HLA-DR+-1	29.90%	2.30%	0.46	3.79E-178
LHFPL2	HLA-DR+-1	47.40%	25.80%	0.50	5.01E-178
MERTK	HLA-DR+-1	24.20%	1.30%	0.39	9.97E-178
GNAQ	HLA-DR+-1	48.90%	27.20%	0.50	1.31E-177
MCOLN1	HLA-DR+-1	39.30%	17.70%	0.47	1.13E-175
GNA15	HLA-DR+-1	33.40%	3.30%	0.44	2.34E-175
FUCA1	HLA-DR+-1	33.20%	19.10%	0.42	2.99E-173
SELPLG	HLA-DR+-1	27.40%	2.20%	0.43	6.04E-173
CXCR4	HLA-DR+-1	40.20%	8.90%	0.33	1.19E-171
DOCK2	HLA-DR+-1	26.00%	1.50%	0.39	4.09E-171
PLAUR	HLA-DR+-1	72.20%	58.90%	0.84	5.41E-171
UNC93B1	HLA-DR+-1	35.80%	10.60%	0.48	6.56E-171
TNFAIP2	HLA-DR+-1	71.20%	48.80%	0.66	6.65E-171
NPL	HLA-DR+-1	25.60%	2.20%	0.37	9.69E-171
NINJ1	HLA-DR+-1	62.10%	35.70%	0.66	3.15E-170
FLNA	HLA-DR+-1	74.30%	56.20%	0.58	3.75E-170
IL10RA	HLA-DR+-1	33.50%	3.70%	0.47	1.37E-169
IL1B	HLA-DR+-1	52.00%	21.60%	1.32	1.79E-169
ARHGAP4	HLA-DR+-1	27.80%	2.20%	0.43	1.63E-168
RAB5C	HLA-DR+-1	71.90%	66.10%	0.42	2.87E-168
HLA-DQA2	HLA-DR+-1	35.20%	5.00%	0.60	7.79E-168
P2RY14	HLA-DR+-1	23.50%	1.10%	0.53	1.32E-167
DAZAP2	HLA-DR+-1	65.00%	54.50%	0.44	3.64E-167
MT-ND2	HLA-DR+-1	99.80%	99.50%	0.54	1.07E-164
CFLAR	HLA-DR+-1	59.30%	37.70%	0.65	5.21E-164
OTUD1	HLA-DR+-1	31.10%	6.00%	0.50	7.95E-163
SPRED1	HLA-DR+-1	39.60%	19.10%	0.44	8.84E-163
CAPZA2	HLA-DR+-1	77.20%	71.10%	0.43	4.32E-162
SKAP2	HLA-DR+-1	38.80%	7.50%	0.52	5.32E-162
BMP2K	HLA-DR+-1	33.90%	8.20%	0.46	5.62E-162
NCKAP1L	HLA-DR+-1	23.60%	1.20%	0.37	7.52E-162
SLC11A1	HLA-DR+-1	24.60%	1.80%	0.38	9.98E-162
ALOX5	HLA-DR+-1	25.90%	1.60%	0.38	1.01E-161

RAP1A	HLA-DR+-1	71.20%	62.30%	0.43	2.49E-161
PLXNB2	HLA-DR+-1	36.60%	15.00%	0.44	4.07E-161
CSF2RA	HLA-DR+-1	29.70%	3.20%	0.38	5.44E-161
SLA	HLA-DR+-1	26.70%	2.00%	0.40	9.17E-161
AP2S1	HLA-DR+-1	70.90%	67.60%	0.40	1.03E-160
BTK	HLA-DR+-1	26.20%	1.70%	0.40	1.46E-160
CCL18	HLA-DR+-1	20.90%	0.90%	0.79	1.53E-160
PIK3R5	HLA-DR+-1	26.70%	1.90%	0.38	2.01E-160
FGL2	HLA-DR+-1	59.30%	22.60%	0.69	7.57E-160
RGS19	HLA-DR+-1	33.60%	7.20%	0.44	4.15E-159
PLXND1	HLA-DR+-1	37.40%	8.20%	0.49	4.96E-159
CAPG	HLA-DR+-1	82.90%	61.40%	0.65	1.75E-158
EVI2A	HLA-DR+-1	43.70%	12.50%	0.59	3.32E-158
CSTA	HLA-DR+-1	34.20%	4.60%	0.50	5.42E-158
MTRNR2L1	HLA-DR+-1	49.80%	17.30%	1.13	6.08E-157
RP11-1143G9.4	HLA-DR+-1	27.80%	2.40%	0.46	1.30E-156
SPINT2	HLA-DR+-1	32.60%	3.80%	0.43	2.31E-156
FABP5	HLA-DR+-1	55.00%	18.70%	0.79	2.32E-156
CECR1	HLA-DR+-1	33.10%	6.30%	0.47	4.72E-154
WAS	HLA-DR+-1	26.00%	1.90%	0.38	7.41E-154
MT-ND1	HLA-DR+-1	99.70%	99.10%	0.54	8.09E-153
TFRC	HLA-DR+-1	33.70%	13.60%	0.47	2.80E-152
DUSP6	HLA-DR+-1	47.80%	15.40%	0.66	2.91E-152
EMILIN2	HLA-DR+-1	30.00%	4.00%	0.45	4.02E-152
ZEB2	HLA-DR+-1	66.10%	48.10%	0.51	4.28E-152
PSTPIP1	HLA-DR+-1	24.70%	1.70%	0.36	1.11E-151
TFEC	HLA-DR+-1	23.80%	1.40%	0.36	3.22E-151
VASP	HLA-DR+-1	58.20%	31.60%	0.54	6.60E-151
QKI	HLA-DR+-1	53.70%	37.60%	0.45	1.52E-150
LRRFIP1	HLA-DR+-1	81.60%	62.80%	0.57	1.53E-148
TM6SF1	HLA-DR+-1	25.20%	1.80%	0.36	1.78E-148
ATP2B1	HLA-DR+-1	66.80%	48.20%	0.57	7.00E-148
PDXK	HLA-DR+-1	33.50%	12.70%	0.41	1.11E-147
GABRB2	HLA-DR+-1	17.10%	0.30%	0.31	3.52E-147
PTAFR	HLA-DR+-1	23.30%	1.40%	0.35	5.91E-147
RB1	HLA-DR+-1	38.40%	18.30%	0.46	7.34E-147
DSC2	HLA-DR+-1	19.30%	0.60%	0.31	1.44E-146
SLC15A3	HLA-DR+-1	27.50%	3.60%	0.36	1.18E-145
MAN1A1	HLA-DR+-1	49.70%	42.90%	0.39	1.48E-142
KLF4	HLA-DR+-1	76.40%	71.00%	0.62	2.02E-141
KIF1B	HLA-DR+-1	43.60%	30.90%	0.39	2.59E-141

CD33	HLA-DR+-1	23.50%	1.60%	0.34	4.29E-141
MARCKS	HLA-DR+-1	50.80%	27.70%	0.63	8.66E-140
CTSH	HLA-DR+-1	57.40%	20.60%	0.55	3.23E-139
GRB2	HLA-DR+-1	55.70%	38.70%	0.44	4.51E-138
NCOA4	HLA-DR+-1	51.20%	31.90%	0.42	4.53E-138
CLEC12A	HLA-DR+-1	32.40%	7.60%	0.47	4.66E-138
TXNIP	HLA-DR+-1	64.80%	42.10%	0.83	6.42E-138
GAS6	HLA-DR+-1	46.70%	17.70%	0.60	6.56E-138
SPP1	HLA-DR+-1	14.20%	9.20%	1.58	1.07E-137
DPEP2	HLA-DR+-1	19.60%	0.90%	0.32	1.35E-137
JUND	HLA-DR+-1	66.90%	58.90%	0.45	1.37E-137
OSTF1	HLA-DR+-1	49.60%	34.30%	0.40	2.61E-136
THEMIS2	HLA-DR+-1	28.10%	7.70%	0.38	7.44E-136
LINC01094	HLA-DR+-1	17.00%	0.50%	0.29	4.37E-134
FAM96A	HLA-DR+-1	39.50%	24.00%	0.38	6.44E-134
LRRC25	HLA-DR+-1	17.90%	0.50%	0.28	4.42E-133
CXCL16	HLA-DR+-1	48.80%	20.50%	0.54	4.90E-133
ARPC4	HLA-DR+-1	64.80%	60.00%	0.38	1.16E-132
RASSF4	HLA-DR+-1	28.80%	4.60%	0.40	2.14E-132
TRMT1	HLA-DR+-1	33.40%	15.00%	0.40	2.82E-132
CARD16	HLA-DR+-1	30.90%	4.90%	0.42	3.21E-132
CITED2	HLA-DR+-1	37.00%	22.00%	0.47	1.54E-131
MTSS1	HLA-DR+-1	37.20%	14.30%	0.45	1.74E-131
RNF150	HLA-DR+-1	16.50%	0.40%	0.28	3.96E-131
CLTC	HLA-DR+-1	50.80%	38.30%	0.38	5.31E-131
RAB31	HLA-DR+-1	68.20%	51.40%	0.49	7.45E-131
RGL1	HLA-DR+-1	28.90%	8.50%	0.41	1.30E-130
MGAT4A	HLA-DR+-1	26.90%	3.80%	0.40	8.98E-130
TMEM37	HLA-DR+-1	18.30%	0.80%	0.28	4.44E-129
COMT	HLA-DR+-1	83.40%	76.90%	0.43	5.18E-129
S100A6	HLA-DR+-1	100.00%	99.90%	0.51	2.28E-128
NUP214	HLA-DR+-1	31.30%	9.30%	0.41	2.74E-128
GNB2	HLA-DR+-1	58.90%	46.10%	0.38	3.16E-128
FBP1	HLA-DR+-1	23.30%	2.80%	0.39	3.19E-128
MT-CO2	HLA-DR+-1	99.80%	99.30%	0.50	7.90E-127
CYTIP	HLA-DR+-1	28.10%	4.70%	0.33	8.30E-127
FAM26F	HLA-DR+-1	22.90%	2.20%	0.35	1.12E-126
LAMTOR2	HLA-DR+-1	57.20%	52.40%	0.32	4.00E-126
DBI	HLA-DR+-1	85.20%	83.80%	0.42	4.73E-126
HMHA1	HLA-DR+-1	25.10%	2.70%	0.38	1.60E-125
PET100	HLA-DR+-1	65.10%	62.20%	0.34	7.71E-125

MT-ND3	HLA-DR+-1	99.70%	98.90%	0.48	8.12E-125
MOB1A	HLA-DR+-1	51.30%	41.20%	0.37	4.68E-124
A2M	HLA-DR+-1	52.80%	19.60%	0.45	5.46E-124
ETS2	HLA-DR+-1	57.50%	29.60%	0.62	1.50E-123
GPR137B	HLA-DR+-1	30.80%	7.30%	0.40	2.29E-123
VOPP1	HLA-DR+-1	38.30%	20.70%	0.39	9.17E-123
PRR13	HLA-DR+-1	53.20%	43.00%	0.36	2.57E-122
COLGALT1	HLA-DR+-1	33.30%	12.80%	0.39	1.25E-121
CNPY3	HLA-DR+-1	38.30%	24.70%	0.36	1.62E-121
IER5	HLA-DR+-1	36.40%	16.00%	0.46	2.94E-121
S100A4	HLA-DR+-1	100.00%	99.40%	0.42	3.79E-121
RALGDS	HLA-DR+-1	36.50%	11.40%	0.45	5.53E-121
NEU1	HLA-DR+-1	55.80%	50.40%	0.39	1.41E-120
FMNL1	HLA-DR+-1	31.60%	7.90%	0.39	1.54E-119
LIPA	HLA-DR+-1	40.00%	22.70%	0.41	1.75E-119
CORO1C	HLA-DR+-1	44.50%	28.10%	0.40	4.06E-119
TKT	HLA-DR+-1	63.70%	53.90%	0.39	6.08E-119
PDGFB	HLA-DR+-1	24.50%	2.80%	0.38	6.25E-119
BAX	HLA-DR+-1	53.10%	41.00%	0.38	1.71E-118
S100A8	HLA-DR+-1	17.70%	2.00%	1.00	3.36E-118
SH3BP2	HLA-DR+-1	28.80%	6.50%	0.37	5.00E-118
GIMAP4	HLA-DR+-1	26.50%	3.60%	0.38	6.36E-118
KCNQ1	HLA-DR+-1	17.00%	0.80%	0.26	2.60E-117
DUSP2	HLA-DR+-1	27.00%	4.00%	0.44	8.76E-117
TTYH3	HLA-DR+-1	24.40%	5.60%	0.34	2.21E-115
KYNU	HLA-DR+-1	41.30%	11.70%	0.51	3.37E-115
CD300A	HLA-DR+-1	20.30%	1.50%	0.29	1.70E-114
BCL2A1	HLA-DR+-1	37.70%	10.50%	0.91	2.73E-114
PDE4DIP	HLA-DR+-1	60.40%	55.90%	0.38	1.36E-113
CYFIP1	HLA-DR+-1	37.60%	22.50%	0.36	4.19E-113
POU2F2	HLA-DR+-1	26.10%	5.50%	0.40	2.03E-112
MYO1G	HLA-DR+-1	24.30%	2.80%	0.34	1.05E-111
CXCL2	HLA-DR+-1	72.50%	58.70%	0.93	9.27E-111
CMTM6	HLA-DR+-1	57.60%	43.00%	0.41	1.29E-110
HSPA6	HLA-DR+-1	27.80%	4.50%	0.74	1.45E-109
H2AFY	HLA-DR+-1	50.40%	43.90%	0.32	2.06E-109
SIRPA	HLA-DR+-1	34.60%	14.80%	0.38	2.62E-109
PSAP	HLA-DR+-1	99.20%	98.30%	0.44	2.84E-109
FPR1	HLA-DR+-1	18.00%	1.10%	0.26	6.41E-109
NAGA	HLA-DR+-1	32.40%	15.00%	0.34	1.40E-108
TTC7A	HLA-DR+-1	24.30%	6.00%	0.34	1.79E-108

NFKBIA	HLA-DR+-1	92.50%	92.40%	0.61	2.53E-108
CHCHD10	HLA-DR+-1	30.30%	7.30%	0.44	3.00E-108
PLD3	HLA-DR+-1	76.20%	79.80%	0.36	3.65E-108
NR4A2	HLA-DR+-1	66.80%	54.50%	0.62	7.95E-108
LAMTOR4	HLA-DR+-1	84.40%	83.20%	0.34	9.43E-108
KLF2	HLA-DR+-1	36.30%	14.80%	0.49	9.85E-108
ADAM17	HLA-DR+-1	43.40%	27.20%	0.42	1.52E-107
HPGD	HLA-DR+-1	22.20%	2.70%	0.33	5.89E-107
AHNAK	HLA-DR+-1	90.80%	90.30%	0.40	9.80E-107
CD36	HLA-DR+-1	25.80%	3.80%	0.49	2.22E-106
CCL3L3	HLA-DR+-1	19.50%	1.50%	0.55	3.75E-106
RAB11FIP1	HLA-DR+-1	24.30%	3.50%	0.34	4.14E-106
PLEKHO1	HLA-DR+-1	32.80%	11.60%	0.38	5.29E-106
TYMP	HLA-DR+-1	45.90%	33.00%	0.49	7.30E-106
FGR	HLA-DR+-1	25.30%	4.90%	0.37	1.13E-105
PARVB	HLA-DR+-1	36.60%	21.40%	0.35	1.50E-105
PTPN18	HLA-DR+-1	29.20%	12.90%	0.34	1.60E-105
SMAP2	HLA-DR+-1	33.30%	9.90%	0.42	3.11E-105
ST3GAL6	HLA-DR+-1	23.10%	3.20%	0.33	1.98E-104
GLUD1	HLA-DR+-1	50.00%	33.50%	0.42	3.36E-104
CD302	HLA-DR+-1	38.90%	21.50%	0.40	4.04E-104
SGMS2	HLA-DR+-1	29.40%	17.10%	0.32	4.56E-104
GSTO1	HLA-DR+-1	73.70%	69.80%	0.37	6.93E-104
EIF4E	HLA-DR+-1	59.60%	61.30%	0.42	8.93E-104
ZYX	HLA-DR+-1	42.70%	25.70%	0.38	3.23E-103
HSPH1	HLA-DR+-1	50.90%	50.70%	0.36	3.73E-103
RASSF2	HLA-DR+-1	22.20%	3.30%	0.33	1.83E-102
ARL8A	HLA-DR+-1	25.10%	13.20%	0.29	5.88E-102
AES	HLA-DR+-1	46.70%	34.40%	0.36	1.23E-101
BIN2	HLA-DR+-1	19.40%	1.90%	0.25	1.29E-101
NLRP3	HLA-DR+-1	23.70%	3.20%	0.38	4.32E-101
SNHG12	HLA-DR+-1	35.80%	25.70%	0.41	2.93E-100
IRF5	HLA-DR+-1	15.30%	0.80%	0.26	1.42E-99
CDC42	HLA-DR+-1	86.50%	77.20%	0.42	5.73E-99
FCGBP	HLA-DR+-1	12.00%	0.30%	0.38	1.74E-98
STK17B	HLA-DR+-1	41.20%	14.90%	0.47	2.81E-98
INPP5D	HLA-DR+-1	16.60%	1.10%	0.26	4.46E-98
IL1A	HLA-DR+-1	22.80%	2.90%	0.46	5.06E-98
RNF13	HLA-DR+-1	59.20%	55.00%	0.33	8.22E-98
MTRNR2L2	HLA-DR+-1	85.30%	80.20%	0.76	1.54E-97
TAGLN2	HLA-DR+-1	97.80%	96.00%	0.39	1.63E-97

NAGK	HLA-DR+-1	43.40%	35.80%	0.29	4.78E-97
PCBP1	HLA-DR+-1	69.60%	65.40%	0.32	1.02E-96
SCAMP2	HLA-DR+-1	37.40%	33.60%	0.25	1.74E-96
PLEKHO2	HLA-DR+-1	31.90%	13.40%	0.34	3.23E-96
SMIM4	HLA-DR+-1	43.50%	33.10%	0.32	1.56E-95
EFHD2	HLA-DR+-1	39.30%	25.60%	0.34	2.04E-95
TNFRSF1B	HLA-DR+-1	31.90%	8.00%	0.34	2.71E-95
RHOA	HLA-DR+-1	94.50%	94.20%	0.35	3.72E-95
PARVG	HLA-DR+-1	16.90%	1.20%	0.25	6.32E-95
WDR26	HLA-DR+-1	35.10%	20.60%	0.32	6.46E-95
DSE	HLA-DR+-1	42.30%	22.60%	0.43	8.58E-95
ATP6V0D1	HLA-DR+-1	56.70%	52.90%	0.31	8.75E-95
DNAJB1	HLA-DR+-1	76.50%	83.20%	0.49	1.63E-94
MGAT1	HLA-DR+-1	65.20%	62.80%	0.31	2.91E-94
GNS	HLA-DR+-1	34.50%	20.40%	0.33	3.72E-94
SPG21	HLA-DR+-1	40.30%	27.20%	0.32	6.99E-93
GAA	HLA-DR+-1	48.20%	36.80%	0.34	7.24E-93
HLA-B	HLA-DR+-1	99.00%	98.10%	0.28	8.92E-93
SGK1	HLA-DR+-1	81.50%	72.90%	0.53	2.02E-92
MYO1F	HLA-DR+-1	19.00%	2.20%	0.28	7.11E-92
B3GNT5	HLA-DR+-1	25.60%	5.50%	0.37	7.20E-92
SQRDL	HLA-DR+-1	39.80%	28.70%	0.31	9.94E-92
ADORA3	HLA-DR+-1	13.70%	0.80%	0.31	1.93E-91
ABCC3	HLA-DR+-1	17.80%	1.90%	0.25	3.58E-91
PABPC1	HLA-DR+-1	96.10%	92.70%	0.39	8.69E-91
RP11-701P16.5	HLA-DR+-1	17.90%	1.60%	0.45	9.61E-91
ABCA1	HLA-DR+-1	35.90%	16.40%	0.41	2.02E-90
ADRBK1	HLA-DR+-1	22.20%	6.70%	0.29	2.71E-90
NISCH	HLA-DR+-1	27.80%	11.80%	0.33	5.99E-90
LAT2	HLA-DR+-1	22.50%	3.30%	0.31	1.43E-89
SLC8A1	HLA-DR+-1	22.80%	5.00%	0.32	1.51E-89
IDH1	HLA-DR+-1	30.70%	15.00%	0.31	2.83E-88
B3GNT7	HLA-DR+-1	18.10%	2.60%	0.29	2.99E-88
NFKB1	HLA-DR+-1	44.80%	33.50%	0.47	4.72E-88
SORL1	HLA-DR+-1	19.50%	2.20%	0.28	5.08E-88
EMP3	HLA-DR+-1	98.50%	94.10%	0.42	1.07E-87
VPS29	HLA-DR+-1	62.60%	62.80%	0.27	1.19E-87
ZFP36L2	HLA-DR+-1	68.70%	56.70%	0.47	1.50E-87
USF2	HLA-DR+-1	31.90%	18.10%	0.30	3.01E-87
DAPK1	HLA-DR+-1	21.20%	4.50%	0.29	3.18E-87
CALM3	HLA-DR+-1	49.50%	49.20%	0.27	3.21E-87

IL6R	HLA-DR+-1	31.40%	15.30%	0.33	3.37E-87
MCL1	HLA-DR+-1	78.50%	71.00%	0.42	5.16E-87
MBNL1	HLA-DR+-1	60.80%	51.30%	0.36	5.83E-87
DUSP23	HLA-DR+-1	46.20%	35.00%	0.35	1.23E-86
YWHAH	HLA-DR+-1	41.10%	31.60%	0.33	1.27E-86
TLR2	HLA-DR+-1	23.20%	5.60%	0.33	2.03E-86
DRAM2	HLA-DR+-1	37.40%	30.00%	0.28	2.19E-86
CSK	HLA-DR+-1	22.30%	8.50%	0.29	2.46E-86
ARPC2	HLA-DR+-1	91.70%	91.70%	0.34	4.73E-86
OLR1	HLA-DR+-1	21.00%	3.10%	0.36	4.77E-86
CCL4L2	HLA-DR+-1	14.20%	0.90%	0.28	6.86E-86
HPCAL1	HLA-DR+-1	25.50%	7.60%	0.33	1.18E-85
TWF2	HLA-DR+-1	46.80%	38.20%	0.28	1.78E-85
RIN3	HLA-DR+-1	21.90%	4.20%	0.30	3.24E-85
MKNK1	HLA-DR+-1	31.20%	16.70%	0.31	4.21E-85
LPXN	HLA-DR+-1	30.30%	7.60%	0.34	4.86E-85
SAT1	HLA-DR+-1	98.20%	95.10%	0.44	5.59E-85
MTRNR2L12	HLA-DR+-1	72.00%	65.00%	0.53	5.61E-85
GNB1	HLA-DR+-1	50.40%	43.20%	0.29	8.02E-85
RNF149	HLA-DR+-1	41.10%	28.90%	0.33	9.49E-85
IL10	HLA-DR+-1	23.10%	4.30%	0.38	1.46E-84
ADCY7	HLA-DR+-1	24.90%	12.30%	0.29	2.50E-84
IL1RN	HLA-DR+-1	21.80%	3.50%	0.55	5.49E-84
GLRX	HLA-DR+-1	72.80%	64.10%	0.53	5.93E-84
TMEM123	HLA-DR+-1	55.60%	42.80%	0.37	3.34E-83
LACTB	HLA-DR+-1	35.10%	17.40%	0.35	7.63E-83
MT-CO3	HLA-DR+-1	99.60%	99.50%	0.41	1.31E-82
SLC38A2	HLA-DR+-1	79.60%	77.40%	0.37	2.77E-82
C10orf118	HLA-DR+-1	39.60%	36.90%	0.29	3.57E-82
TAGAP	HLA-DR+-1	18.80%	2.40%	0.29	4.86E-82
METRNL	HLA-DR+-1	35.10%	23.70%	0.30	8.15E-82
DMXL2	HLA-DR+-1	21.00%	5.70%	0.26	2.07E-81
SMS	HLA-DR+-1	47.40%	37.50%	0.34	3.66E-81
SLC6A6	HLA-DR+-1	38.00%	23.30%	0.34	3.95E-81
TAOK3	HLA-DR+-1	43.70%	35.40%	0.30	4.17E-81
TNFSF13B	HLA-DR+-1	28.50%	7.80%	0.36	9.16E-81
STT3B	HLA-DR+-1	33.40%	21.40%	0.30	1.12E-80
MT-ATP6	HLA-DR+-1	99.70%	99.20%	0.41	1.56E-80
GM2A	HLA-DR+-1	23.80%	9.90%	0.27	5.15E-80
ITSN2	HLA-DR+-1	44.20%	32.90%	0.33	1.02E-79
GNPDA1	HLA-DR+-1	22.80%	9.00%	0.28	3.58E-79

DAAM1	HLA-DR+-1	42.80%	26.70%	0.38	3.78E-79
SLC4A7	HLA-DR+-1	31.50%	20.20%	0.31	2.09E-78
ARF6	HLA-DR+-1	42.50%	26.70%	0.37	4.02E-78
PPP1R9B	HLA-DR+-1	24.10%	9.80%	0.27	5.33E-78
ENTPD1	HLA-DR+-1	27.70%	6.70%	0.34	8.25E-78
ACTR3	HLA-DR+-1	65.10%	61.30%	0.30	9.73E-78
CLIC1	HLA-DR+-1	96.90%	94.60%	0.35	1.21E-77
CD97	HLA-DR+-1	40.80%	33.70%	0.30	1.61E-77
MEF2A	HLA-DR+-1	47.00%	43.10%	0.29	2.89E-77
CAB39	HLA-DR+-1	29.70%	16.10%	0.29	4.07E-77
HLA-DOA	HLA-DR+-1	16.00%	1.60%	0.26	5.73E-77
YWHAZ	HLA-DR+-1	75.60%	74.10%	0.30	7.17E-77
PLEKHB2	HLA-DR+-1	43.80%	32.00%	0.32	1.53E-76
ZFAND5	HLA-DR+-1	54.20%	51.20%	0.29	6.85E-76
NCEH1	HLA-DR+-1	20.70%	5.80%	0.25	1.28E-75
CREBL2	HLA-DR+-1	26.00%	16.20%	0.26	1.46E-75
MCTP1	HLA-DR+-1	23.80%	4.80%	0.28	1.57E-75
REL	HLA-DR+-1	64.50%	45.20%	0.49	2.13E-75
SLC48A1	HLA-DR+-1	23.30%	14.20%	0.25	3.89E-75
TLR4	HLA-DR+-1	19.30%	4.60%	0.27	5.04E-75
FKBP5	HLA-DR+-1	37.00%	26.60%	0.32	5.28E-75
GNG2	HLA-DR+-1	22.00%	4.50%	0.30	6.89E-75
SLC12A7	HLA-DR+-1	19.30%	4.90%	0.27	1.50E-74
ZFHX3	HLA-DR+-1	53.60%	45.90%	0.31	1.52E-74
DUSP5	HLA-DR+-1	23.10%	12.00%	0.29	3.44E-74
ARHGAP6	HLA-DR+-1	25.60%	11.50%	0.31	4.07E-74
STX7	HLA-DR+-1	36.60%	28.00%	0.27	8.69E-74
RPL38	HLA-DR+-1	99.80%	99.20%	0.29	1.27E-73
SGMS1	HLA-DR+-1	25.70%	15.60%	0.26	2.01E-73
RASGRP3	HLA-DR+-1	20.80%	3.70%	0.31	2.15E-73
PPP1R18	HLA-DR+-1	40.10%	31.40%	0.28	3.80E-73
CYB5R4	HLA-DR+-1	25.40%	10.40%	0.27	9.05E-73
CYTH1	HLA-DR+-1	35.40%	17.10%	0.33	9.72E-73
ARID3A	HLA-DR+-1	20.10%	3.90%	0.30	1.10E-72
TMEM176B	HLA-DR+-1	62.50%	32.40%	0.48	1.15E-72
CAPZA1	HLA-DR+-1	47.20%	37.40%	0.30	1.40E-72
ABHD12	HLA-DR+-1	26.70%	14.00%	0.27	2.87E-72
TGOLN2	HLA-DR+-1	55.50%	51.40%	0.26	3.77E-72
OGFRL1	HLA-DR+-1	26.20%	10.80%	0.31	4.47E-72
ALCAM	HLA-DR+-1	28.90%	11.90%	0.35	1.20E-71
CMTM3	HLA-DR+-1	32.90%	20.70%	0.31	1.35E-71

HEXB	HLA-DR+-1	70.60%	72.50%	0.26	4.86E-71
SLC9A9	HLA-DR+-1	24.90%	11.70%	0.28	5.05E-71
CXCL1	HLA-DR+-1	54.20%	45.50%	0.88	5.27E-71
LAMP2	HLA-DR+-1	64.40%	55.90%	0.33	5.57E-71
ACSL1	HLA-DR+-1	24.10%	11.40%	0.28	6.02E-71
FOXN3	HLA-DR+-1	39.20%	34.00%	0.26	7.24E-71
SLC31A2	HLA-DR+-1	31.00%	23.30%	0.25	9.17E-71
GNA13	HLA-DR+-1	27.40%	15.30%	0.28	1.18E-70
RNF144B	HLA-DR+-1	22.40%	5.70%	0.30	2.01E-70
NAPRT1	HLA-DR+-1	29.80%	17.80%	0.28	3.41E-70
M6PR	HLA-DR+-1	41.70%	33.70%	0.26	3.57E-70
TLE4	HLA-DR+-1	47.30%	42.30%	0.32	4.85E-70
ATP13A3	HLA-DR+-1	32.80%	25.40%	0.30	5.42E-70
PTBP3	HLA-DR+-1	29.80%	21.10%	0.26	7.96E-70
CELF1	HLA-DR+-1	34.30%	24.80%	0.29	9.30E-70
P2RX4	HLA-DR+-1	26.10%	11.20%	0.28	1.14E-69
DIAPH1	HLA-DR+-1	38.70%	33.20%	0.26	4.00E-69
MBP	HLA-DR+-1	35.40%	15.20%	0.37	4.66E-69
PREX1	HLA-DR+-1	23.10%	6.70%	0.27	9.86E-69
KHDRBS1	HLA-DR+-1	36.40%	30.80%	0.25	1.29E-68
CRYL1	HLA-DR+-1	35.80%	26.00%	0.28	2.22E-68
LACC1	HLA-DR+-1	19.50%	5.50%	0.25	2.40E-68
CMTM7	HLA-DR+-1	21.70%	8.50%	0.26	3.83E-68
RPS29	HLA-DR+-1	100.00%	99.40%	0.26	1.28E-67
MAP2K3	HLA-DR+-1	58.30%	55.40%	0.30	1.56E-67
ZNF267	HLA-DR+-1	37.80%	23.70%	0.37	2.02E-67
DDX3X	HLA-DR+-1	73.80%	69.70%	0.33	2.28E-67
RP11-108M9.4	HLA-DR+-1	19.00%	8.90%	0.25	2.64E-67
PRKACB	HLA-DR+-1	20.30%	7.60%	0.25	2.80E-67
HHEX	HLA-DR+-1	19.60%	4.20%	0.27	3.99E-67
CBX4	HLA-DR+-1	23.60%	14.30%	0.25	4.40E-67
LAP3	HLA-DR+-1	36.90%	30.60%	0.26	5.16E-67
USP9X	HLA-DR+-1	32.00%	21.40%	0.27	7.69E-67
SLC7A8	HLA-DR+-1	25.20%	13.00%	0.28	1.21E-66
TRIM28	HLA-DR+-1	32.50%	24.40%	0.25	1.73E-66
FNIP2	HLA-DR+-1	44.50%	43.40%	0.32	2.27E-66
PFDN5	HLA-DR+-1	98.40%	98.20%	0.27	4.53E-66
PLA2G16	HLA-DR+-1	42.20%	35.30%	0.27	5.08E-66
PTPRE	HLA-DR+-1	38.80%	18.30%	0.39	9.23E-66
TPT1	HLA-DR+-1	99.90%	99.70%	0.27	1.16E-65
PICALM	HLA-DR+-1	39.50%	31.00%	0.27	1.56E-65

NBPF10	HLA-DR+-1	36.20%	30.70%	0.27	2.23E-65
CD44	HLA-DR+-1	91.20%	90.10%	0.37	6.40E-65
SLC25A24	HLA-DR+-1	27.20%	15.20%	0.26	9.82E-65
SNX29	HLA-DR+-1	28.20%	17.40%	0.26	2.77E-64
LFNG	HLA-DR+-1	17.80%	3.70%	0.26	2.99E-64
AHR	HLA-DR+-1	51.50%	36.00%	0.40	3.67E-64
ABI1	HLA-DR+-1	39.50%	31.60%	0.25	8.80E-64
PTPLAD2	HLA-DR+-1	24.80%	10.00%	0.26	9.75E-64
MAP3K2	HLA-DR+-1	38.00%	29.60%	0.28	1.50E-63
RPS6KA3	HLA-DR+-1	38.40%	28.00%	0.27	1.73E-63
ARHGEF10L	HLA-DR+-1	21.10%	7.20%	0.26	2.11E-63
C6orf62	HLA-DR+-1	58.50%	57.20%	0.27	3.11E-63
MSN	HLA-DR+-1	71.30%	69.80%	0.27	8.26E-63
ADAM9	HLA-DR+-1	45.80%	34.10%	0.30	1.44E-62
CELF2	HLA-DR+-1	50.20%	41.20%	0.31	1.50E-62
RAP2B	HLA-DR+-1	27.00%	10.90%	0.31	3.15E-62
RIT1	HLA-DR+-1	35.10%	24.00%	0.29	9.91E-62
FLI1	HLA-DR+-1	21.90%	6.60%	0.26	1.37E-61
GIMAP1	HLA-DR+-1	17.70%	3.00%	0.26	1.38E-61
MIS18BP1	HLA-DR+-1	29.80%	17.60%	0.31	2.74E-61
S100A10	HLA-DR+-1	99.40%	99.30%	0.40	9.25E-61
RPS21	HLA-DR+-1	99.60%	98.60%	0.29	1.38E-60
ATP5D	HLA-DR+-1	41.40%	31.30%	0.33	1.69E-60
GCHFR	HLA-DR+-1	18.30%	8.20%	0.26	3.18E-60
FRMD4A	HLA-DR+-1	19.10%	4.60%	0.26	9.67E-60
MMP19	HLA-DR+-1	20.80%	15.60%	0.25	1.11E-59
ANTXR2	HLA-DR+-1	38.30%	27.90%	0.29	3.63E-59
MT-ND4	HLA-DR+-1	99.80%	99.60%	0.31	4.80E-59
TMEM160	HLA-DR+-1	40.20%	32.90%	0.26	1.51E-58
CLN8	HLA-DR+-1	30.50%	20.20%	0.27	1.57E-58
NSMAF	HLA-DR+-1	23.40%	9.30%	0.30	1.73E-58
C19orf60	HLA-DR+-1	61.60%	58.10%	0.26	3.51E-58
CCL13	HLA-DR+-1	15.60%	2.40%	0.36	1.03E-57
PLK3	HLA-DR+-1	35.00%	23.20%	0.31	4.65E-57
PRDM1	HLA-DR+-1	22.60%	5.60%	0.32	8.08E-57
IFNGR2	HLA-DR+-1	37.00%	28.20%	0.26	8.37E-57
PIM3	HLA-DR+-1	33.00%	24.10%	0.32	3.45E-56
KIAA1033	HLA-DR+-1	31.90%	21.80%	0.25	5.09E-56
STX11	HLA-DR+-1	32.00%	14.30%	0.29	6.79E-56
SLC16A10	HLA-DR+-1	27.00%	15.50%	0.30	1.29E-55
NRP2	HLA-DR+-1	45.30%	32.50%	0.34	2.12E-55

RAB32	HLA-DR+-1	41.40%	34.00%	0.25	2.27E-55
WSB1	HLA-DR+-1	67.60%	68.50%	0.27	2.33E-55
THBD	HLA-DR+-1	44.70%	30.30%	0.51	2.89E-55
TGFB1	HLA-DR+-1	48.70%	43.10%	0.26	4.62E-55
LPCAT2	HLA-DR+-1	30.20%	15.60%	0.29	3.32E-54
STMN1	HLA-DR+-1	35.00%	14.90%	0.35	4.18E-54
MMP9	HLA-DR+-1	17.50%	4.30%	0.43	7.95E-54
MYL12A	HLA-DR+-1	90.30%	91.00%	0.27	8.86E-54
ABL2	HLA-DR+-1	62.50%	66.70%	0.26	1.83E-53
NFKBID	HLA-DR+-1	31.40%	14.20%	0.32	2.60E-53
MT-ND4L	HLA-DR+-1	88.30%	86.30%	0.32	4.73E-53
MTRNR2L8	HLA-DR+-1	69.40%	63.80%	0.40	4.75E-53
COCH	HLA-DR+-1	16.20%	5.50%	0.28	7.35E-53
MAP3K11	HLA-DR+-1	21.20%	8.30%	0.26	8.95E-53
MT-ATP8	HLA-DR+-1	38.70%	28.90%	0.28	2.04E-52
RHOB	HLA-DR+-1	57.30%	52.90%	0.34	5.60E-52
MAP2K1	HLA-DR+-1	28.70%	17.20%	0.27	9.57E-52
HSP90AA1	HLA-DR+-1	97.00%	97.80%	0.39	2.30E-51
MIR155HG	HLA-DR+-1	22.40%	7.00%	0.38	4.44E-50
CFL1	HLA-DR+-1	98.70%	98.40%	0.25	5.24E-50
NRP1	HLA-DR+-1	37.10%	25.80%	0.29	3.18E-49
BID	HLA-DR+-1	35.60%	25.70%	0.27	3.45E-49
UPP1	HLA-DR+-1	40.10%	26.80%	0.31	3.72E-49
ADRB2	HLA-DR+-1	23.40%	10.60%	0.27	4.93E-49
EMP1	HLA-DR+-1	88.00%	87.30%	0.35	1.39E-48
RPL36A	HLA-DR+-1	92.10%	94.90%	0.27	4.43E-46
CCRL2	HLA-DR+-1	16.50%	4.80%	0.25	2.56E-45
NABP1	HLA-DR+-1	31.30%	20.90%	0.29	5.34E-45
APOC1	HLA-DR+-1	14.50%	10.10%	0.57	2.06E-44
CALM2	HLA-DR+-1	97.00%	98.00%	0.26	2.58E-44
ICAM1	HLA-DR+-1	52.10%	38.00%	0.40	3.84E-44
ARPC1B	HLA-DR+-1	90.30%	86.20%	0.28	8.33E-44
INSIG1	HLA-DR+-1	64.10%	61.50%	0.37	1.31E-43
KDM6B	HLA-DR+-1	60.90%	55.20%	0.31	2.24E-43
ASAP1	HLA-DR+-1	34.00%	23.10%	0.25	6.50E-43
SLC43A3	HLA-DR+-1	36.50%	25.30%	0.27	3.73E-42
LUCAT1	HLA-DR+-1	28.80%	13.50%	0.33	1.30E-41
CXCL5	HLA-DR+-1	10.50%	1.30%	0.39	1.53E-41
LPAR6	HLA-DR+-1	21.70%	7.50%	0.31	6.48E-39
JUN	HLA-DR+-1	88.10%	87.40%	0.41	3.50E-38
MGST2	HLA-DR+-1	31.80%	15.90%	0.26	3.94E-38

MT-ND5	HLA-DR+-1	98.80%	98.40%	0.25	3.96E-38
PLAU	HLA-DR+-1	51.60%	33.30%	0.40	5.90E-38
PPP1R15A	HLA-DR+-1	87.90%	91.00%	0.29	4.75E-37
NEAT1	HLA-DR+-1	99.50%	99.40%	0.31	8.98E-37
TNFAIP8	HLA-DR+-1	38.10%	24.90%	0.35	1.42E-36
XIST	HLA-DR+-1	60.90%	43.10%	0.39	1.51E-36
BTG1	HLA-DR+-1	70.30%	68.90%	0.30	2.05E-36
RN7SL1	HLA-DR+-1	17.70%	10.80%	0.31	7.94E-30
HMOX1	HLA-DR+-1	67.00%	55.10%	0.41	1.02E-27
CCL2	HLA-DR+-1	69.90%	70.50%	0.46	4.51E-25
PMAIP1	HLA-DR+-1	40.30%	25.60%	0.32	7.48E-25
FOSB	HLA-DR+-1	82.00%	86.00%	0.27	1.73E-23
FILIP1L	HLA-DR+-1	52.20%	42.50%	0.29	2.95E-23
SOD2	HLA-DR+-1	81.50%	87.40%	0.26	9.32E-18
7SK.2	HLA-DR+-1	25.10%	20.00%	0.26	1.51E-16
APOE	HLA-DR+-1	18.20%	14.20%	0.45	2.17E-16
IL1B	HLA-DR+-2	86.80%	22.10%	3.45	0.00E+00
HLA-DRA	HLA-DR+-2	97.20%	38.20%	3.10	0.00E+00
IL8	HLA-DR+-2	95.60%	66.90%	3.00	0.00E+00
G0S2	HLA-DR+-2	65.50%	17.80%	2.98	0.00E+00
HLA-DPB1	HLA-DR+-2	97.40%	38.10%	2.87	0.00E+00
HLA-DPA1	HLA-DR+-2	99.20%	28.60%	2.86	0.00E+00
FCER1A	HLA-DR+-2	81.10%	2.80%	2.75	0.00E+00
HLA-DQB1	HLA-DR+-2	96.40%	13.40%	2.70	0.00E+00
HLA-DQA1	HLA-DR+-2	95.10%	9.40%	2.70	0.00E+00
HLA-DRB5	HLA-DR+-2	96.60%	17.50%	2.59	0.00E+00
HLA-DRB1	HLA-DR+-2	98.70%	29.00%	2.58	0.00E+00
CD74	HLA-DR+-2	100.00%	48.60%	2.45	0.00E+00
SRGN	HLA-DR+-2	98.70%	42.00%	2.37	0.00E+00
GPR183	HLA-DR+-2	92.20%	17.60%	2.35	0.00E+00
PLAUR	HLA-DR+-2	93.80%	58.90%	2.03	0.00E+00
HLA-DQA2	HLA-DR+-2	77.70%	5.10%	2.02	0.00E+00
HLA-DMA	HLA-DR+-2	95.90%	18.50%	1.79	0.00E+00
IL1R2	HLA-DR+-2	66.60%	1.60%	1.73	0.00E+00
REL	HLA-DR+-2	92.70%	45.20%	1.70	0.00E+00
CLEC10A	HLA-DR+-2	76.90%	1.60%	1.65	0.00E+00
TNFAIP8	HLA-DR+-2	79.80%	24.10%	1.52	0.00E+00
SERPINB9	HLA-DR+-2	79.80%	16.40%	1.51	0.00E+00
INSIG1	HLA-DR+-2	90.20%	60.60%	1.48	0.00E+00
C15orf48	HLA-DR+-2	67.10%	7.90%	2.03	3.46209011288063e-315

TYROBP	HLA-DR+-2	95.90%	19.20%	1.41	2.46239003348338e-311
BCL2A1	HLA-DR+-2	75.90%	10.70%	2.37	3.62E-308
LYZ	HLA-DR+-2	93.30%	13.30%	2.11	2.86E-304
RGS1	HLA-DR+-2	90.40%	14.20%	2.09	4.34E-303
LGALS2	HLA-DR+-2	56.50%	0.40%	1.12	1.00E-297
CD83	HLA-DR+-2	93.00%	24.00%	1.86	6.44E-292
STX11	HLA-DR+-2	74.60%	13.80%	1.27	7.04E-288
LST1	HLA-DR+-2	86.80%	9.00%	1.35	3.03E-286
FCER1G	HLA-DR+-2	95.10%	18.20%	1.53	6.20E-281
CXCL3	HLA-DR+-2	88.90%	52.70%	2.46	3.75E-274
LAPTM5	HLA-DR+-2	94.60%	19.70%	1.34	5.88E-268
TRAF1	HLA-DR+-2	64.50%	5.50%	1.35	7.18E-268
PLEK	HLA-DR+-2	79.50%	8.90%	1.58	5.51E-259
CD1C	HLA-DR+-2	46.40%	0.20%	1.32	3.60E-256
HLA-DMB	HLA-DR+-2	82.10%	8.90%	1.29	5.19E-253
CFLAR	HLA-DR+-2	86.00%	37.90%	1.16	3.52E-249
CYTIP	HLA-DR+-2	69.20%	4.50%	1.31	4.83E-248
AMICA1	HLA-DR+-2	54.40%	1.00%	0.91	8.30E-248
CSF2RA	HLA-DR+-2	63.20%	3.50%	1.11	2.54E-244
AIF1	HLA-DR+-2	88.90%	14.80%	1.06	4.70E-243
CPVL	HLA-DR+-2	77.50%	9.80%	1.50	3.06E-242
MS4A6A	HLA-DR+-2	86.80%	12.10%	1.39	9.03E-241
HCST	HLA-DR+-2	78.50%	8.40%	1.27	2.35E-235
RILPL2	HLA-DR+-2	70.50%	30.80%	0.97	5.58E-235
ATP2B1	HLA-DR+-2	84.70%	48.60%	1.06	1.60E-233
PABPC1	HLA-DR+-2	99.50%	92.70%	0.93	6.76E-233
LINC00936	HLA-DR+-2	60.10%	16.60%	1.09	1.89E-231
FCGR2B	HLA-DR+-2	77.50%	7.80%	1.21	1.56E-227
MIR155HG	HLA-DR+-2	55.70%	6.70%	1.52	3.53E-226
KYNU	HLA-DR+-2	67.90%	12.40%	1.22	4.89E-226
CXCR4	HLA-DR+-2	76.70%	9.40%	1.40	1.48E-216
RHOF	HLA-DR+-2	54.70%	4.90%	0.98	4.69E-213
CCL3	HLA-DR+-2	81.10%	40.40%	2.18	4.51E-212
IL10	HLA-DR+-2	52.80%	4.30%	1.62	1.31E-211
LUCAT1	HLA-DR+-2	62.40%	13.10%	1.10	1.65E-211
PHACTR1	HLA-DR+-2	60.40%	4.60%	1.09	1.72E-211
BTG1	HLA-DR+-2	87.60%	68.30%	1.17	1.24E-208
EHD1	HLA-DR+-2	68.70%	35.20%	0.91	9.16E-208
CCR7	HLA-DR+-2	46.10%	1.50%	1.65	6.00E-207
CYBA	HLA-DR+-2	99.00%	77.20%	1.02	1.84E-205
OLR1	HLA-DR+-2	53.40%	2.90%	1.30	2.15E-204

CFP	HLA-DR+-2	50.30%	1.50%	0.82	2.65E-204
CD86	HLA-DR+-2	59.80%	3.70%	0.92	4.75E-203
NLRP3	HLA-DR+-2	56.50%	3.10%	1.07	1.30E-202
SAT1	HLA-DR+-2	99.00%	95.20%	1.25	1.63E-202
TXNRD1	HLA-DR+-2	63.70%	31.40%	0.96	6.71E-202
CD48	HLA-DR+-2	50.30%	1.60%	0.78	1.25E-201
AGPAT9	HLA-DR+-2	47.40%	2.20%	0.92	3.60E-198
STK4	HLA-DR+-2	66.30%	15.40%	1.03	2.90E-196
TNFAIP3	HLA-DR+-2	84.50%	27.60%	1.44	2.04E-195
NFKB1	HLA-DR+-2	72.00%	33.10%	1.05	2.91E-192
CXCL16	HLA-DR+-2	69.20%	21.30%	0.98	1.22E-191
FCGR2A	HLA-DR+-2	76.40%	11.30%	1.11	6.24E-190
MIR29A	HLA-DR+-2	56.20%	14.40%	0.82	1.70E-187
PNRC1	HLA-DR+-2	88.30%	56.70%	1.08	6.63E-187
TNFRSF1B	HLA-DR+-2	62.20%	8.20%	1.00	2.95E-185
RNASET2	HLA-DR+-2	74.60%	30.10%	0.99	2.98E-182
ITGB2	HLA-DR+-2	72.30%	8.90%	0.97	5.46E-182
SERPINB1	HLA-DR+-2	68.90%	49.60%	0.85	5.46E-181
BID	HLA-DR+-2	61.40%	25.30%	0.82	2.83E-180
EIF4E	HLA-DR+-2	80.60%	60.40%	0.99	2.66E-178
TMEM123	HLA-DR+-2	76.90%	42.80%	0.84	5.94E-178
CD53	HLA-DR+-2	70.20%	8.70%	0.95	2.82E-177
LPXN	HLA-DR+-2	62.40%	7.70%	0.90	2.73E-175
RNASE6	HLA-DR+-2	62.20%	5.50%	0.96	4.37E-175
CLEC7A	HLA-DR+-2	61.90%	5.60%	0.94	4.97E-175
BIRC3	HLA-DR+-2	79.80%	21.30%	1.40	5.65E-174
IRF8	HLA-DR+-2	60.10%	7.40%	1.16	1.32E-173
NFKBID	HLA-DR+-2	64.80%	14.00%	0.90	4.24E-172
LITAF	HLA-DR+-2	81.90%	60.40%	0.81	2.48E-171
VSIG4	HLA-DR+-2	71.50%	10.90%	0.89	8.37E-169
PPIF	HLA-DR+-2	54.70%	10.30%	0.86	1.48E-164
PTPRC	HLA-DR+-2	66.10%	8.80%	0.80	4.07E-163
MAP2K3	HLA-DR+-2	81.90%	54.70%	0.80	4.23E-162
EREG	HLA-DR+-2	45.90%	9.70%	1.73	8.18E-162
CD37	HLA-DR+-2	67.40%	8.70%	0.85	2.66E-160
MXD1	HLA-DR+-2	44.00%	19.70%	0.74	4.58E-160
PDE4B	HLA-DR+-2	69.70%	20.10%	0.89	4.16E-157
CTSS	HLA-DR+-2	89.60%	30.20%	0.93	3.00E-156
CORO1A	HLA-DR+-2	63.20%	7.60%	0.85	1.00E-155
TNIP1	HLA-DR+-2	57.00%	32.50%	0.67	2.06E-155
IL1A	HLA-DR+-2	47.90%	3.10%	1.08	1.08E-154

LCP1	HLA-DR+-2	62.20%	7.30%	0.78	6.71E-152
DUSP4	HLA-DR+-2	49.20%	15.70%	1.07	3.66E-150
THAP2	HLA-DR+-2	47.90%	17.10%	0.78	1.71E-146
IL10RA	HLA-DR+-2	53.10%	4.70%	0.73	5.60E-145
MAP3K8	HLA-DR+-2	79.00%	51.40%	0.82	1.37E-143
CMTM6	HLA-DR+-2	72.00%	43.30%	0.73	6.57E-143
IL1RN	HLA-DR+-2	44.60%	3.70%	1.47	1.69E-141
ARL8B	HLA-DR+-2	61.70%	29.20%	0.69	2.32E-141
VASP	HLA-DR+-2	73.10%	32.60%	0.80	8.50E-141
HCLS1	HLA-DR+-2	60.90%	8.30%	0.70	1.28E-140
PTPRE	HLA-DR+-2	65.80%	18.50%	0.91	1.61E-140
CCL22	HLA-DR+-2	28.20%	0.70%	1.18	4.04E-140
GMFG	HLA-DR+-2	72.00%	14.40%	0.74	2.18E-138
PMAIP1	HLA-DR+-2	73.60%	25.20%	1.15	3.32E-138
VAMP8	HLA-DR+-2	71.80%	17.30%	0.57	4.53E-138
DNAJB6	HLA-DR+-2	84.50%	70.50%	0.70	5.91E-138
NFKBIA	HLA-DR+-2	98.40%	92.20%	1.10	1.14E-137
ISG20	HLA-DR+-2	47.90%	17.90%	0.82	2.80E-137
EZR	HLA-DR+-2	84.20%	59.10%	0.85	4.19E-137
BAZ1A	HLA-DR+-2	81.60%	56.10%	0.78	1.31E-136
NINJ1	HLA-DR+-2	66.80%	37.00%	0.89	8.48E-136
SAMSN1	HLA-DR+-2	48.70%	4.50%	1.16	1.25E-135
FTH1	HLA-DR+-2	100.00%	100.00%	0.91	3.36E-135
CXCL2	HLA-DR+-2	85.80%	59.00%	1.68	3.88E-135
HLA-B	HLA-DR+-2	99.70%	98.10%	0.71	3.88E-134
GLIPR1	HLA-DR+-2	78.50%	48.90%	0.85	1.38E-133
PPP1R15A	HLA-DR+-2	98.40%	90.40%	0.96	9.33E-133
ETV3	HLA-DR+-2	49.20%	10.50%	0.66	4.90E-130
CREM	HLA-DR+-2	69.40%	44.70%	1.01	1.74E-129
C1orf162	HLA-DR+-2	55.40%	6.60%	0.85	1.91E-129
PKIB	HLA-DR+-2	31.10%	1.00%	0.55	3.72E-128
FNBP1	HLA-DR+-2	61.90%	21.90%	0.75	5.84E-127
GNA15	HLA-DR+-2	47.70%	4.50%	0.69	1.11E-125
GPX1	HLA-DR+-2	95.60%	83.60%	0.76	8.89E-122
MCL1	HLA-DR+-2	90.40%	70.90%	0.77	2.39E-120
H3F3A	HLA-DR+-2	98.70%	98.30%	0.58	6.32E-120
DSE	HLA-DR+-2	56.70%	23.20%	0.74	1.36E-117
AKAP13	HLA-DR+-2	81.90%	61.10%	0.71	2.25E-117
GPBP1	HLA-DR+-2	75.90%	56.80%	0.63	4.52E-117
CD1E	HLA-DR+-2	22.30%	0.10%	0.43	5.00E-116
IL4I1	HLA-DR+-2	27.70%	0.80%	0.58	2.75E-114

GPSM3	HLA-DR+-2	51.80%	6.70%	0.67	2.93E-114
RALA	HLA-DR+-2	53.90%	35.40%	0.58	5.94E-113
RP6-91H8.3	HLA-DR+-2	26.40%	0.50%	0.49	1.45E-112
NAMPT	HLA-DR+-2	77.50%	51.70%	0.76	9.00E-112
ANKRD28	HLA-DR+-2	72.50%	58.60%	0.73	1.08E-110
FABP5	HLA-DR+-2	68.10%	20.30%	1.07	5.01E-110
ATP13A3	HLA-DR+-2	53.10%	25.10%	0.67	2.39E-109
TCHH	HLA-DR+-2	30.80%	1.70%	0.76	5.24E-109
GPR132	HLA-DR+-2	35.80%	2.00%	0.50	2.46E-108
ADAM8	HLA-DR+-2	31.30%	1.40%	0.51	3.18E-107
SPINT2	HLA-DR+-2	45.30%	5.00%	0.59	1.19E-106
AP1S2	HLA-DR+-2	78.50%	38.80%	0.73	8.00E-106
DAPP1	HLA-DR+-2	27.70%	0.80%	0.51	2.95E-105
NFKB2	HLA-DR+-2	56.50%	22.20%	0.61	7.79E-105
B3GNT5	HLA-DR+-2	43.30%	6.00%	0.65	1.09E-104
TMSB10	HLA-DR+-2	100.00%	98.00%	0.63	1.16E-104
SDCBP	HLA-DR+-2	92.50%	90.50%	0.70	1.38E-104
MCOLN2	HLA-DR+-2	28.50%	1.00%	0.46	1.91E-104
EVI2B	HLA-DR+-2	50.30%	7.20%	0.61	2.38E-102
MIR142	HLA-DR+-2	33.70%	2.00%	0.54	8.78E-101
RHOG	HLA-DR+-2	71.50%	41.70%	0.68	1.27E-100
ARHGDIB	HLA-DR+-2	70.50%	19.70%	0.68	3.99E-100
PIM3	HLA-DR+-2	59.30%	23.60%	0.73	9.09E-100
RASGEF1B	HLA-DR+-2	60.40%	21.30%	0.85	2.68E-99
PTGIR	HLA-DR+-2	39.90%	11.10%	0.61	9.70E-99
DNTTIP2	HLA-DR+-2	64.00%	41.50%	0.62	2.44E-98
ALCAM	HLA-DR+-2	50.00%	12.10%	0.63	7.48E-98
CSTA	HLA-DR+-2	44.80%	5.90%	0.65	1.25E-97
PLD4	HLA-DR+-2	21.20%	0.20%	0.35	4.46E-97
MYO1G	HLA-DR+-2	38.90%	3.50%	0.53	2.23E-96
HNRNPC	HLA-DR+-2	93.30%	81.60%	0.57	4.52E-96
CLEC5A	HLA-DR+-2	31.30%	1.70%	0.55	6.35E-96
ALOX5AP	HLA-DR+-2	58.00%	13.00%	0.62	2.71E-94
FGR	HLA-DR+-2	42.00%	5.40%	0.49	2.17E-93
ZNF267	HLA-DR+-2	56.70%	23.80%	0.68	3.26E-93
NR4A2	HLA-DR+-2	71.50%	55.10%	0.95	5.98E-93
CD40	HLA-DR+-2	38.60%	24.00%	0.49	6.58E-92
SMAP2	HLA-DR+-2	49.20%	10.60%	0.63	8.48E-92
LINC00152	HLA-DR+-2	61.70%	47.80%	0.82	1.31E-91
MNDA	HLA-DR+-2	42.70%	5.20%	0.73	2.99E-91
C5AR1	HLA-DR+-2	46.90%	8.90%	0.87	2.94E-89

LY86	HLA-DR+-2	38.10%	3.80%	0.51	8.13E-89
SORL1	HLA-DR+-2	33.40%	2.70%	0.48	6.84E-87
CARD16	HLA-DR+-2	40.40%	6.00%	0.60	3.56E-86
ACSL5	HLA-DR+-2	36.00%	4.70%	0.45	4.15E-86
TCOF1	HLA-DR+-2	31.90%	11.90%	0.59	5.63E-86
FCN1	HLA-DR+-2	18.90%	0.20%	0.50	5.77E-86
ETS2	HLA-DR+-2	69.40%	30.80%	0.79	2.41E-85
CCL3L1	HLA-DR+-2	39.40%	5.20%	1.31	5.44E-85
CCL4	HLA-DR+-2	64.20%	33.70%	1.50	7.37E-85
RIT1	HLA-DR+-2	49.00%	24.10%	0.57	8.00E-85
OGFRL1	HLA-DR+-2	41.50%	11.10%	0.56	1.18E-84
YBX1	HLA-DR+-2	85.20%	81.90%	0.76	1.49E-84
LAT2	HLA-DR+-2	37.00%	3.90%	0.46	7.11E-84
TNF	HLA-DR+-2	54.70%	14.70%	1.08	8.95E-84
TET2	HLA-DR+-2	42.50%	7.60%	0.52	1.07E-83
ADAM28	HLA-DR+-2	21.00%	0.40%	0.35	3.76E-83
PRDM1	HLA-DR+-2	40.20%	5.90%	0.71	4.26E-83
CDC42EP3	HLA-DR+-2	55.70%	16.80%	0.75	4.61E-83
SPI1	HLA-DR+-2	42.70%	7.10%	0.49	9.42E-83
BASP1	HLA-DR+-2	46.10%	30.30%	0.55	2.65E-82
PTPN1	HLA-DR+-2	56.50%	33.60%	0.57	2.27E-81
LCP2	HLA-DR+-2	38.90%	5.30%	0.60	7.42E-81
SLC7A11	HLA-DR+-2	31.30%	6.70%	0.75	1.03E-80
RBM47	HLA-DR+-2	44.00%	6.90%	0.57	1.32E-80
AREG	HLA-DR+-2	24.40%	1.00%	0.41	1.87E-80
MAFG	HLA-DR+-2	50.50%	25.00%	0.53	1.97E-80
TNIP3	HLA-DR+-2	21.50%	1.00%	0.68	9.37E-80
DDX3X	HLA-DR+-2	79.80%	69.70%	0.58	2.30E-79
RPS29	HLA-DR+-2	100.00%	99.40%	0.47	2.64E-79
MMP9	HLA-DR+-2	34.70%	4.40%	0.73	3.12E-79
CST3	HLA-DR+-2	98.40%	99.10%	0.72	7.22E-79
TES	HLA-DR+-2	52.30%	32.50%	0.52	1.20E-78
HMGA1	HLA-DR+-2	56.50%	20.80%	0.63	1.59E-78
KLF4	HLA-DR+-2	60.40%	71.90%	0.63	1.89E-78
MBP	HLA-DR+-2	50.30%	15.80%	0.61	2.82E-78
IL7R	HLA-DR+-2	29.50%	2.40%	0.63	2.85E-78
GK	HLA-DR+-2	31.30%	7.60%	0.49	3.69E-78
TMEM120B	HLA-DR+-2	27.20%	3.70%	0.43	4.46E-78
CTSH	HLA-DR+-2	67.10%	22.40%	0.60	6.80E-78
CHD2	HLA-DR+-2	54.90%	32.00%	0.56	1.90E-77
B2M	HLA-DR+-2	100.00%	100.00%	0.39	3.98E-77

VMO1	HLA-DR+-2	35.50%	4.10%	0.66	4.92E-77
CCRL2	HLA-DR+-2	33.40%	4.90%	0.53	9.15E-77
PDGFB	HLA-DR+-2	34.20%	3.70%	0.51	3.66E-76
TBXAS1	HLA-DR+-2	39.90%	5.70%	0.48	6.17E-76
CD58	HLA-DR+-2	57.30%	38.30%	0.49	1.60E-75
IL23A	HLA-DR+-2	21.00%	0.80%	0.65	1.82E-74
SFR1	HLA-DR+-2	28.20%	8.80%	0.44	3.23E-74
TAGAP	HLA-DR+-2	30.80%	2.90%	0.53	3.77E-74
SIPA1L1	HLA-DR+-2	45.60%	19.60%	0.49	1.41E-73
ARRB2	HLA-DR+-2	49.70%	11.90%	0.58	1.45E-73
TANK	HLA-DR+-2	61.40%	38.20%	0.52	2.11E-73
HSPA1A	HLA-DR+-2	51.00%	68.10%	0.82	3.01E-73
UPP1	HLA-DR+-2	56.50%	27.00%	0.60	4.02E-73
ZEB2	HLA-DR+-2	66.80%	49.10%	0.56	4.73E-73
FAM49B	HLA-DR+-2	55.20%	18.20%	0.57	5.36E-73
ADAM19	HLA-DR+-2	21.80%	2.50%	0.43	6.97E-73
CD52	HLA-DR+-2	33.20%	4.20%	0.62	1.01E-72
NSMAF	HLA-DR+-2	36.30%	9.70%	0.55	1.25E-72
RUNX3	HLA-DR+-2	30.30%	3.00%	0.43	1.47E-72
RASGRP3	HLA-DR+-2	34.20%	4.20%	0.50	1.63E-72
MAFF	HLA-DR+-2	51.30%	26.80%	0.56	1.76E-72
ARL5B	HLA-DR+-2	40.70%	18.30%	0.50	2.55E-72
HLA-DOA	HLA-DR+-2	27.50%	2.00%	0.38	2.74E-72
RP11-701P16.5	HLA-DR+-2	27.70%	2.20%	0.81	9.98E-72
CSF3R	HLA-DR+-2	22.00%	0.90%	0.33	1.17E-71
HLA-DQB2	HLA-DR+-2	17.90%	0.30%	0.28	1.89E-71
GRB2	HLA-DR+-2	65.50%	39.30%	0.52	2.62E-71
EIF1	HLA-DR+-2	100.00%	99.80%	0.39	3.48E-71
RASSF5	HLA-DR+-2	34.50%	5.20%	0.47	3.81E-71
CSF1R	HLA-DR+-2	39.10%	7.00%	0.40	4.56E-71
PTGS2	HLA-DR+-2	58.50%	28.00%	0.91	6.63E-71
CCL3L3	HLA-DR+-2	28.00%	2.20%	0.79	9.74E-71
HCAR2	HLA-DR+-2	22.50%	1.20%	0.44	2.11E-70
TPT1	HLA-DR+-2	99.70%	99.70%	0.45	4.57E-70
ARL4C	HLA-DR+-2	45.60%	17.80%	0.65	6.05E-70
ATP1B3	HLA-DR+-2	78.00%	70.10%	0.53	1.04E-69
CHMP1B	HLA-DR+-2	72.80%	63.70%	0.61	1.64E-69
PPP1R15B	HLA-DR+-2	50.50%	29.00%	0.48	4.34E-69
MS4A7	HLA-DR+-2	43.80%	8.40%	0.57	1.16E-68
AHR	HLA-DR+-2	64.00%	36.50%	0.63	2.15E-68
CKLF	HLA-DR+-2	52.80%	36.50%	0.49	2.87E-68

PELI1	HLA-DR+-2	48.40%	26.40%	0.63	2.95E-68
HAVCR2	HLA-DR+-2	35.50%	4.80%	0.46	4.20E-68
DOK2	HLA-DR+-2	39.60%	6.50%	0.54	2.79E-67
LIMD2	HLA-DR+-2	25.60%	2.30%	0.35	2.08E-66
SYAP1	HLA-DR+-2	54.40%	40.50%	0.46	5.55E-66
OSM	HLA-DR+-2	26.40%	2.30%	0.49	7.72E-66
COTL1	HLA-DR+-2	53.10%	15.90%	0.54	1.59E-65
KDM6B	HLA-DR+-2	83.20%	54.70%	0.61	4.47E-65
IFI30	HLA-DR+-2	35.50%	6.20%	0.83	5.35E-65
HSPA1B	HLA-DR+-2	36.50%	54.60%	0.72	5.65E-65
SFPQ	HLA-DR+-2	78.00%	64.20%	0.51	1.52E-64
YWHAZ	HLA-DR+-2	83.90%	73.90%	0.48	1.98E-64
RNF144B	HLA-DR+-2	31.30%	6.30%	0.52	3.67E-64
DUSP2	HLA-DR+-2	32.60%	5.10%	0.59	5.64E-64
CECR1	HLA-DR+-2	39.10%	7.60%	0.45	6.31E-64
CLEC4E	HLA-DR+-2	20.70%	1.00%	0.40	1.16E-63
TNFSF13B	HLA-DR+-2	40.40%	8.50%	0.51	1.30E-63
KMO	HLA-DR+-2	22.30%	1.30%	0.39	1.75E-63
CLN8	HLA-DR+-2	42.20%	20.30%	0.48	2.42E-63
ICAM1	HLA-DR+-2	70.50%	38.10%	0.71	4.93E-63
BACH1	HLA-DR+-2	46.10%	24.90%	0.47	7.06E-63
ADA	HLA-DR+-2	29.50%	7.90%	0.48	8.39E-63
VEGFA	HLA-DR+-2	48.40%	21.60%	0.53	8.72E-63
MIR181A1HG	HLA-DR+-2	22.50%	1.70%	0.35	1.28E-62
MAP2K1	HLA-DR+-2	43.00%	17.30%	0.49	1.59E-62
SPAG9	HLA-DR+-2	71.80%	61.30%	0.47	2.03E-62
NRP2	HLA-DR+-2	54.90%	32.90%	0.57	3.48E-62
GPR84	HLA-DR+-2	23.30%	1.60%	0.34	4.48E-62
MGAT1	HLA-DR+-2	72.30%	62.70%	0.43	6.70E-62
CD69	HLA-DR+-2	24.60%	3.10%	0.28	2.86E-61
RAB11FIP1	HLA-DR+-2	30.80%	4.40%	0.50	1.03E-60
RPS16	HLA-DR+-2	99.70%	99.40%	0.38	1.08E-60
DNAJB1	HLA-DR+-2	59.10%	83.50%	0.32	1.60E-60
TLR2	HLA-DR+-2	31.60%	6.30%	0.45	3.12E-60
NABP1	HLA-DR+-2	50.30%	20.80%	0.54	3.66E-60
IL18	HLA-DR+-2	32.40%	4.30%	0.43	3.91E-60
RAC2	HLA-DR+-2	29.30%	4.20%	0.33	4.71E-60
LPAR6	HLA-DR+-2	35.50%	7.80%	0.64	5.56E-60
TNFAIP2	HLA-DR+-2	69.40%	50.10%	0.61	8.96E-60
HCAR3	HLA-DR+-2	17.90%	0.70%	0.34	8.99E-60
YME1L1	HLA-DR+-2	55.70%	45.30%	0.44	1.48E-59

CKS2	HLA-DR+-2	52.80%	53.90%	0.48	2.18E-59
RPS10	HLA-DR+-2	97.90%	96.00%	0.43	3.77E-59
PRMT10	HLA-DR+-2	24.60%	6.00%	0.47	4.25E-59
SGK1	HLA-DR+-2	77.50%	73.60%	0.69	1.33E-58
RPS21	HLA-DR+-2	100.00%	98.60%	0.40	1.43E-58
ITGAX	HLA-DR+-2	25.10%	2.60%	0.37	2.18E-58
RGS2	HLA-DR+-2	75.10%	59.00%	0.87	1.45E-57
NCF2	HLA-DR+-2	31.90%	4.60%	0.39	2.06E-57
RP11-1143G9.4	HLA-DR+-2	29.30%	3.80%	0.51	2.91E-57
STK17B	HLA-DR+-2	49.70%	16.10%	0.57	3.89E-57
FAM49A	HLA-DR+-2	49.70%	28.90%	0.46	4.78E-57
KB-1507C5.4	HLA-DR+-2	18.40%	0.80%	0.28	5.80E-57
RAB9A	HLA-DR+-2	28.80%	23.70%	0.36	6.44E-57
IL2RG	HLA-DR+-2	21.80%	2.00%	0.27	1.48E-56
SOD2	HLA-DR+-2	93.00%	86.60%	0.97	1.82E-56
HCK	HLA-DR+-2	31.60%	4.50%	0.39	2.75E-56
CD44	HLA-DR+-2	96.60%	90.00%	0.51	3.58E-56
CYBB	HLA-DR+-2	37.60%	10.10%	0.28	5.81E-56
ST3GAL6	HLA-DR+-2	29.30%	4.10%	0.41	6.24E-56
FPR3	HLA-DR+-2	35.20%	7.00%	0.39	6.48E-56
LILRB2	HLA-DR+-2	28.00%	3.20%	0.38	6.63E-56
RAPGEF1	HLA-DR+-2	35.80%	11.00%	0.42	9.29E-56
KLF6	HLA-DR+-2	91.50%	79.90%	0.69	1.74E-55
ZFYVE16	HLA-DR+-2	38.90%	19.10%	0.42	6.17E-55
JARID2	HLA-DR+-2	33.20%	10.70%	0.42	1.29E-54
CXorf21	HLA-DR+-2	16.80%	0.60%	0.27	2.13E-54
RPL36A	HLA-DR+-2	98.40%	94.50%	0.48	3.28E-54
WAS	HLA-DR+-2	26.40%	3.30%	0.29	3.46E-54
SLAMF7	HLA-DR+-2	14.50%	0.40%	0.28	9.42E-54
TRA2B	HLA-DR+-2	68.90%	61.00%	0.50	1.60E-53
GLA	HLA-DR+-2	51.80%	36.10%	0.47	4.92E-53
PLK3	HLA-DR+-2	44.60%	23.50%	0.48	8.35E-53
PID1	HLA-DR+-2	40.40%	23.60%	0.42	1.33E-52
PNP	HLA-DR+-2	56.70%	39.30%	0.47	1.68E-52
YPEL5	HLA-DR+-2	58.00%	60.40%	0.34	1.79E-52
PHLDA2	HLA-DR+-2	64.50%	55.30%	0.64	2.27E-52
CTB-58E17.1	HLA-DR+-2	25.60%	5.70%	0.34	3.44E-52
AKIRIN2	HLA-DR+-2	43.80%	32.50%	0.35	4.36E-52
CST7	HLA-DR+-2	23.10%	2.10%	0.48	1.12E-51
SH2B3	HLA-DR+-2	43.50%	13.50%	0.48	2.38E-51
MT-CO1	HLA-DR+-2	100.00%	99.50%	0.43	2.73E-51

RPS19	HLA-DR+-2	99.70%	99.50%	0.35	7.08E-51
STMN1	HLA-DR+-2	47.20%	15.60%	0.59	1.02E-50
MARCKSL1	HLA-DR+-2	43.50%	17.70%	0.50	1.43E-50
CD300A	HLA-DR+-2	24.10%	2.40%	0.33	1.44E-50
SERPINB8	HLA-DR+-2	38.60%	16.10%	0.40	1.54E-49
RP11-386I14.4	HLA-DR+-2	27.70%	6.70%	0.55	7.76E-49
MSR1	HLA-DR+-2	37.30%	8.70%	0.43	1.03E-48
WSB1	HLA-DR+-2	70.50%	68.30%	0.43	2.58E-48
FAU	HLA-DR+-2	100.00%	99.50%	0.31	3.53E-48
NEU1	HLA-DR+-2	46.40%	51.10%	0.32	5.50E-48
IKZF1	HLA-DR+-2	20.20%	1.90%	0.26	1.23E-47
RP11-58E21.3	HLA-DR+-2	19.20%	2.70%	0.30	2.26E-47
IQGAP2	HLA-DR+-2	29.00%	4.50%	0.39	2.57E-47
ARID3A	HLA-DR+-2	27.50%	4.50%	0.39	2.88E-47
ATP1B1	HLA-DR+-2	44.30%	22.00%	0.49	3.62E-47
SEMA6B	HLA-DR+-2	23.10%	3.40%	0.32	3.87E-47
GLRX	HLA-DR+-2	72.00%	64.60%	0.62	4.06E-47
MMP19	HLA-DR+-2	23.60%	15.80%	0.38	4.99E-47
NFE2L2	HLA-DR+-2	87.30%	78.60%	0.49	5.41E-47
RAB8B	HLA-DR+-2	40.70%	18.90%	0.40	7.57E-47
FAM107B	HLA-DR+-2	45.90%	30.20%	0.41	8.91E-47
RPL28	HLA-DR+-2	100.00%	99.70%	0.35	9.88E-47
PPT1	HLA-DR+-2	63.70%	49.60%	0.45	1.06E-46
ARHGAP26	HLA-DR+-2	26.70%	4.70%	0.35	2.09E-46
TFEC	HLA-DR+-2	23.60%	2.80%	0.31	2.61E-46
ARPC3	HLA-DR+-2	92.70%	87.40%	0.41	2.99E-46
NUMB	HLA-DR+-2	42.70%	26.10%	0.40	4.74E-46
RNF145	HLA-DR+-2	46.90%	29.80%	0.43	5.90E-46
PLEKHF2	HLA-DR+-2	27.70%	8.70%	0.35	8.07E-46
LGALS9	HLA-DR+-2	32.40%	7.00%	0.35	3.19E-45
THBS1	HLA-DR+-2	20.20%	17.70%	0.58	4.09E-45
FGL2	HLA-DR+-2	56.70%	24.80%	0.60	4.66E-45
SNHG15	HLA-DR+-2	45.60%	36.90%	0.38	4.86E-45
ATP6V0D1	HLA-DR+-2	66.10%	52.80%	0.40	9.78E-45
DBI	HLA-DR+-2	89.90%	83.70%	0.43	1.97E-44
SNX10	HLA-DR+-2	31.90%	6.40%	0.39	2.69E-44
NRIP3	HLA-DR+-2	26.90%	10.90%	0.35	2.98E-44
JMJD1C	HLA-DR+-2	64.80%	59.80%	0.41	2.00E-43
HMHA1	HLA-DR+-2	25.40%	4.00%	0.30	2.49E-43
FERMT3	HLA-DR+-2	36.30%	9.20%	0.41	3.42E-43
PTGER2	HLA-DR+-2	38.10%	28.70%	0.40	3.57E-43

ATF5	HLA-DR+-2	30.60%	10.40%	0.35	3.74E-43
ARF6	HLA-DR+-2	43.50%	27.60%	0.43	4.96E-43
PIK3R5	HLA-DR+-2	24.10%	3.50%	0.28	5.18E-43
NBPF10	HLA-DR+-2	47.90%	30.60%	0.43	1.25E-42
CXCL5	HLA-DR+-2	17.10%	1.60%	0.86	2.05E-42
RP11-796E2.4	HLA-DR+-2	19.70%	2.40%	0.33	2.40E-42
PIM2	HLA-DR+-2	22.50%	6.10%	0.39	2.55E-42
RHOH	HLA-DR+-2	19.20%	2.10%	0.28	7.26E-42
C10orf128	HLA-DR+-2	24.10%	3.60%	0.34	1.10E-41
TYMP	HLA-DR+-2	53.90%	33.40%	0.52	1.60E-41
IGSF6	HLA-DR+-2	25.40%	4.70%	0.28	1.77E-41
CDKN1A	HLA-DR+-2	95.60%	82.80%	0.49	2.85E-41
SNORD3B-2	HLA-DR+-2	18.90%	3.80%	0.28	5.24E-41
CCDC88A	HLA-DR+-2	52.60%	27.90%	0.45	2.86E-40
MARCKS	HLA-DR+-2	47.90%	29.20%	0.55	5.39E-40
ZNF706	HLA-DR+-2	65.30%	65.70%	0.30	5.98E-40
RP11-356I2.4	HLA-DR+-2	19.20%	2.20%	0.26	7.20E-40
UBE2D3	HLA-DR+-2	90.20%	84.80%	0.36	1.43E-39
RIN3	HLA-DR+-2	26.70%	5.00%	0.31	1.48E-39
ZBTB43	HLA-DR+-2	43.80%	30.90%	0.36	1.52E-39
CYTH1	HLA-DR+-2	42.00%	18.00%	0.40	1.65E-39
CSGALNACT2	HLA-DR+-2	38.60%	22.80%	0.35	3.79E-39
STXBP2	HLA-DR+-2	25.10%	4.10%	0.28	5.09E-39
NCF4	HLA-DR+-2	26.40%	5.00%	0.28	5.50E-39
PDE4DIP	HLA-DR+-2	55.70%	56.40%	0.32	7.70E-39
ALOX5	HLA-DR+-2	21.80%	3.10%	0.25	1.58E-38
ETF1	HLA-DR+-2	65.30%	51.90%	0.36	1.73E-38
CASP1	HLA-DR+-2	29.00%	8.70%	0.36	2.14E-38
HOTAIRM1	HLA-DR+-2	29.80%	14.10%	0.33	7.62E-38
MCTP1	HLA-DR+-2	26.70%	5.80%	0.27	8.80E-38
PTGER4	HLA-DR+-2	38.90%	21.80%	0.42	9.32E-38
HSPH1	HLA-DR+-2	42.20%	51.00%	0.27	1.28E-37
SLC25A19	HLA-DR+-2	27.70%	8.10%	0.32	3.88E-37
ZFAND5	HLA-DR+-2	54.40%	51.30%	0.33	4.07E-37
CLEC2B	HLA-DR+-2	44.30%	15.70%	0.53	9.43E-37
FBP1	HLA-DR+-2	23.30%	4.00%	0.26	9.88E-37
NFAT5	HLA-DR+-2	54.90%	39.30%	0.36	1.20E-36
DENND5A	HLA-DR+-2	36.80%	23.00%	0.31	1.55E-36
HLA-C	HLA-DR+-2	98.70%	97.40%	0.51	2.01E-36
NR4A3	HLA-DR+-2	46.10%	24.30%	0.46	4.33E-36
FAM26F	HLA-DR+-2	22.30%	3.50%	0.29	6.20E-36

ANTXR2	HLA-DR+-2	44.00%	28.30%	0.37	6.61E-36
RPLP1	HLA-DR+-2	100.00%	99.80%	0.27	1.07E-35
IFNGR2	HLA-DR+-2	42.50%	28.50%	0.35	1.57E-35
GNA13	HLA-DR+-2	37.00%	15.60%	0.35	1.66E-35
MAP4K4	HLA-DR+-2	30.30%	16.50%	0.33	3.63E-35
LACTB	HLA-DR+-2	36.00%	18.40%	0.37	3.75E-35
LRRFIP1	HLA-DR+-2	79.80%	63.90%	0.46	5.61E-35
ZC3H12A	HLA-DR+-2	60.10%	44.60%	0.42	6.90E-35
CLK1	HLA-DR+-2	61.40%	59.90%	0.33	1.54E-34
LYPD3	HLA-DR+-2	10.60%	3.40%	0.27	1.71E-34
BNIP3L	HLA-DR+-2	60.40%	60.60%	0.29	1.81E-34
HERPUD1	HLA-DR+-2	76.40%	87.30%	0.27	2.08E-34
DUSP6	HLA-DR+-2	42.00%	17.50%	0.50	2.68E-34
MAP1LC3B	HLA-DR+-2	82.10%	82.50%	0.34	2.74E-34
ALDH2	HLA-DR+-2	58.80%	39.20%	0.41	3.34E-34
SLC31A2	HLA-DR+-2	39.40%	23.40%	0.34	3.37E-34
RAB7A	HLA-DR+-2	83.90%	82.90%	0.31	3.43E-34
ST8SIA4	HLA-DR+-2	25.90%	5.80%	0.33	5.25E-34
STK10	HLA-DR+-2	24.60%	7.20%	0.28	1.16E-33
ATP1A1	HLA-DR+-2	76.70%	71.10%	0.35	1.52E-33
SLC43A2	HLA-DR+-2	33.90%	12.30%	0.39	1.74E-33
ARHGAP30	HLA-DR+-2	25.10%	4.90%	0.31	1.77E-33
RGS10	HLA-DR+-2	67.90%	50.30%	0.40	3.31E-33
SRSF2	HLA-DR+-2	88.10%	83.70%	0.37	3.89E-33
MYO9B	HLA-DR+-2	31.10%	13.10%	0.32	4.01E-33
PAK1	HLA-DR+-2	31.60%	11.10%	0.33	6.02E-33
IRF7	HLA-DR+-2	32.90%	24.10%	0.31	9.20E-33
H2AFZ	HLA-DR+-2	93.80%	91.00%	0.49	1.18E-32
EIF4A3	HLA-DR+-2	71.20%	68.00%	0.43	1.91E-32
ARL6IP1	HLA-DR+-2	75.90%	65.80%	0.39	2.53E-32
RELT	HLA-DR+-2	23.80%	6.10%	0.26	3.02E-32
LILRB4	HLA-DR+-2	23.80%	4.70%	0.27	4.46E-32
TP53BP2	HLA-DR+-2	27.20%	13.70%	0.29	4.56E-32
MAPKAPK2	HLA-DR+-2	44.80%	28.80%	0.32	5.16E-32
SKIL	HLA-DR+-2	65.00%	52.50%	0.39	6.21E-32
PILRA	HLA-DR+-2	23.30%	4.50%	0.26	6.74E-32
CEP170	HLA-DR+-2	35.80%	20.40%	0.33	1.05E-31
PDE4A	HLA-DR+-2	32.10%	13.10%	0.33	1.28E-31
RPS24	HLA-DR+-2	100.00%	99.70%	0.29	1.46E-31
CYCS	HLA-DR+-2	90.90%	81.40%	0.42	1.49E-31
IER5	HLA-DR+-2	37.00%	17.20%	0.39	2.02E-31

ARFGAP3	HLA-DR+-2	49.20%	40.30%	0.30	2.16E-31
PPP2CA	HLA-DR+-2	57.00%	46.50%	0.32	2.30E-31
CXCR3	HLA-DR+-2	16.60%	2.50%	0.33	2.78E-31
RNF213	HLA-DR+-2	21.20%	31.90%	0.26	4.64E-31
SRC	HLA-DR+-2	20.20%	6.30%	0.26	4.83E-31
BIN2	HLA-DR+-2	19.70%	2.90%	0.25	5.26E-31
BST2	HLA-DR+-2	56.00%	27.50%	0.27	7.55E-31
FTL	HLA-DR+-2	100.00%	99.80%	0.33	1.36E-30
TM6SF1	HLA-DR+-2	20.70%	3.30%	0.26	1.38E-30
ZFAND2A	HLA-DR+-2	31.60%	26.20%	0.29	1.40E-30
SLC7A5	HLA-DR+-2	34.70%	22.80%	0.35	1.93E-30
ICAM3	HLA-DR+-2	25.10%	17.00%	0.27	2.93E-30
EEF1B2	HLA-DR+-2	98.20%	94.80%	0.35	3.35E-30
TPRA1	HLA-DR+-2	32.40%	19.30%	0.29	4.48E-30
FYB	HLA-DR+-2	27.50%	7.10%	0.30	8.56E-30
RP2	HLA-DR+-2	24.40%	8.50%	0.29	9.50E-30
FILIP1L	HLA-DR+-2	54.90%	43.00%	0.51	1.15E-29
EVI2A	HLA-DR+-2	39.10%	14.50%	0.33	1.16E-29
VMP1	HLA-DR+-2	62.70%	59.40%	0.34	1.28E-29
IRAK2	HLA-DR+-2	31.30%	14.20%	0.28	1.60E-29
RB1	HLA-DR+-2	37.00%	19.60%	0.35	2.36E-29
NFKBIE	HLA-DR+-2	21.50%	6.10%	0.26	3.08E-29
USP12	HLA-DR+-2	32.40%	20.60%	0.29	6.52E-29
DENND1B	HLA-DR+-2	24.90%	8.70%	0.28	8.59E-29
RPS27	HLA-DR+-2	100.00%	99.90%	0.26	1.03E-28
SKAP2	HLA-DR+-2	30.60%	9.60%	0.27	1.50E-28
RPL27	HLA-DR+-2	100.00%	99.10%	0.26	1.50E-28
IFNGR1	HLA-DR+-2	49.00%	45.10%	0.28	1.76E-28
PLEKHB2	HLA-DR+-2	46.90%	32.60%	0.33	1.92E-28
RPL23	HLA-DR+-2	100.00%	99.10%	0.28	2.06E-28
ENTPD1	HLA-DR+-2	29.30%	7.90%	0.29	2.42E-28
GLS	HLA-DR+-2	43.50%	35.50%	0.29	2.56E-28
PIK3AP1	HLA-DR+-2	20.70%	3.60%	0.28	2.82E-28
DOCK8	HLA-DR+-2	22.80%	5.70%	0.26	2.85E-28
C1QC	HLA-DR+-2	37.80%	15.40%	0.31	3.23E-28
SNHG12	HLA-DR+-2	29.00%	26.60%	0.31	3.89E-28
EDEM1	HLA-DR+-2	29.00%	11.30%	0.28	4.91E-28
PFN1	HLA-DR+-2	97.20%	96.00%	0.36	5.48E-28
FNIP2	HLA-DR+-2	45.60%	43.40%	0.34	8.08E-28
ATP6V0B	HLA-DR+-2	81.60%	76.00%	0.33	1.43E-27
CDC42	HLA-DR+-2	87.30%	77.70%	0.36	2.20E-27

MAPK6	HLA-DR+-2	45.10%	31.80%	0.32	6.95E-27
CD93	HLA-DR+-2	22.00%	4.70%	0.27	7.32E-27
C3orf58	HLA-DR+-2	24.10%	17.40%	0.27	7.33E-27
SYNGR2	HLA-DR+-2	37.30%	18.20%	0.37	8.81E-27
SNN	HLA-DR+-2	21.20%	6.30%	0.26	1.07E-26
ZBTB1	HLA-DR+-2	34.20%	18.70%	0.32	1.18E-26
PABPC4	HLA-DR+-2	42.20%	28.60%	0.33	1.49E-26
HIVEP2	HLA-DR+-2	35.20%	27.90%	0.26	1.97E-26
TAB2	HLA-DR+-2	30.60%	17.70%	0.27	2.08E-26
RPS7	HLA-DR+-2	99.50%	99.00%	0.28	2.65E-26
CYLD	HLA-DR+-2	44.30%	32.10%	0.31	4.51E-26
PFKFB3	HLA-DR+-2	44.60%	30.20%	0.35	4.82E-26
SLC3A2	HLA-DR+-2	78.50%	75.80%	0.39	5.86E-26
MED13	HLA-DR+-2	33.90%	25.30%	0.26	1.28E-25
RIPK2	HLA-DR+-2	35.80%	25.10%	0.28	1.34E-25
DENND4A	HLA-DR+-2	22.80%	8.60%	0.26	1.67E-25
C9orf72	HLA-DR+-2	20.70%	5.70%	0.26	1.95E-25
HNRNPU	HLA-DR+-2	70.50%	67.50%	0.30	3.58E-25
ATP6V1B2	HLA-DR+-2	48.70%	38.40%	0.31	4.12E-25
VIM-AS1	HLA-DR+-2	26.70%	12.20%	0.27	6.81E-25
ARHGEF2	HLA-DR+-2	38.60%	24.20%	0.29	7.67E-25
SUB1	HLA-DR+-2	85.50%	82.20%	0.30	2.24E-24
PPP1R2	HLA-DR+-2	47.70%	39.00%	0.27	2.71E-24
ITSN2	HLA-DR+-2	47.70%	33.50%	0.31	3.81E-24
MTHFD2	HLA-DR+-2	60.10%	47.80%	0.37	5.33E-24
IFRD1	HLA-DR+-2	62.40%	59.70%	0.32	5.40E-24
LIMS1	HLA-DR+-2	60.90%	59.00%	0.26	5.99E-24
ATF3	HLA-DR+-2	67.60%	69.30%	0.45	8.12E-24
H2AFY	HLA-DR+-2	54.40%	44.10%	0.27	9.26E-24
ELF1	HLA-DR+-2	62.20%	51.70%	0.32	1.02E-23
TPM3	HLA-DR+-2	76.90%	72.50%	0.33	1.04E-23
RAP2B	HLA-DR+-2	26.90%	11.80%	0.32	1.37E-23
LYN	HLA-DR+-2	25.60%	9.60%	0.26	1.57E-23
CHMP4B	HLA-DR+-2	65.80%	61.70%	0.26	3.50E-23
RCSD1	HLA-DR+-2	20.50%	4.20%	0.28	4.02E-23
CPM	HLA-DR+-2	31.30%	10.70%	0.31	6.21E-23
ACSL1	HLA-DR+-2	27.20%	12.00%	0.29	7.14E-23
MARCH1	HLA-DR+-2	18.10%	3.50%	0.29	2.71E-22
PEA15	HLA-DR+-2	60.90%	53.70%	0.28	2.79E-22
TAOK3	HLA-DR+-2	46.40%	35.80%	0.28	3.24E-22
POU2F2	HLA-DR+-2	23.10%	6.80%	0.29	3.79E-22

HLA-A	HLA-DR+-2	99.00%	98.20%	0.28	4.42E-22
TGIF1	HLA-DR+-2	59.10%	46.80%	0.35	4.51E-22
MAPRE1	HLA-DR+-2	54.70%	43.70%	0.27	5.15E-22
NFKBIZ	HLA-DR+-2	71.80%	60.20%	0.39	8.34E-22
FAM105A	HLA-DR+-2	22.80%	6.20%	0.26	1.05E-21
MEF2C	HLA-DR+-2	44.30%	21.30%	0.37	1.16E-21
RPL38	HLA-DR+-2	100.00%	99.20%	0.25	1.24E-21
TPP1	HLA-DR+-2	50.50%	33.30%	0.31	2.29E-21
TGFB1	HLA-DR+-2	54.40%	43.20%	0.30	3.23E-21
MIS18BP1	HLA-DR+-2	25.90%	18.50%	0.28	3.82E-21
SERPINA1	HLA-DR+-2	32.40%	13.10%	0.30	4.48E-21
DDIT4	HLA-DR+-2	71.80%	60.90%	0.41	5.82E-21
VOPP1	HLA-DR+-2	35.00%	21.90%	0.28	7.39E-21
RPS2	HLA-DR+-2	100.00%	99.60%	0.27	8.80E-21
SLC25A5	HLA-DR+-2	84.20%	80.80%	0.29	9.49E-21
FOSL2	HLA-DR+-2	49.50%	39.60%	0.29	9.60E-21
EPB41L3	HLA-DR+-2	37.80%	27.10%	0.29	1.07E-20
BIRC2	HLA-DR+-2	47.20%	40.00%	0.26	1.73E-20
USP53	HLA-DR+-2	48.20%	41.30%	0.28	2.68E-20
NUP214	HLA-DR+-2	26.40%	10.70%	0.26	2.87E-20
RP11-138A9.1	HLA-DR+-2	39.10%	26.50%	0.29	8.89E-20
SERP1	HLA-DR+-2	83.70%	78.20%	0.28	1.71E-19
CD14	HLA-DR+-2	52.60%	33.00%	0.32	1.88E-19
TOP1	HLA-DR+-2	58.30%	53.90%	0.27	2.83E-19
RPPH1	HLA-DR+-2	18.40%	5.50%	0.28	2.83E-19
AC016831.7	HLA-DR+-2	24.60%	11.40%	0.25	3.75E-19
ZFAS1	HLA-DR+-2	91.20%	90.20%	0.30	4.54E-19
ABCA1	HLA-DR+-2	29.50%	17.80%	0.31	5.07E-19
DUSP1	HLA-DR+-2	66.80%	56.30%	0.44	6.99E-19
CDC42SE1	HLA-DR+-2	46.10%	31.00%	0.34	7.08E-19
PPP1CB	HLA-DR+-2	63.70%	58.40%	0.28	8.85E-19
ZNF331	HLA-DR+-2	37.80%	24.20%	0.44	9.51E-19
DDX5	HLA-DR+-2	99.50%	98.00%	0.31	1.01E-18
CSTB	HLA-DR+-2	82.90%	82.20%	0.32	1.03E-18
RASSF4	HLA-DR+-2	21.80%	6.30%	0.26	1.08E-18
CLEC12A	HLA-DR+-2	26.40%	9.30%	0.26	1.15E-18
LRRC8C	HLA-DR+-2	30.60%	18.40%	0.27	1.31E-18
MTSS1	HLA-DR+-2	31.30%	15.90%	0.28	2.31E-18
CSRNP1	HLA-DR+-2	60.90%	56.60%	0.28	2.38E-18
MFSD2A	HLA-DR+-2	37.80%	24.50%	0.29	2.62E-18
C3AR1	HLA-DR+-2	22.30%	6.10%	0.27	3.15E-18

RBM8A	HLA-DR+-2	83.70%	78.60%	0.27	3.90E-18
PYCARD	HLA-DR+-2	48.70%	30.70%	0.29	4.24E-18
RNF130	HLA-DR+-2	67.10%	57.50%	0.27	6.28E-18
WTAP	HLA-DR+-2	63.50%	57.60%	0.38	1.03E-17
RP1-313I6.12	HLA-DR+-2	15.50%	5.10%	0.25	1.25E-17
NAP1L1	HLA-DR+-2	91.20%	87.80%	0.28	1.85E-17
TIPARP	HLA-DR+-2	66.10%	50.70%	0.34	2.66E-17
CAPZA1	HLA-DR+-2	49.00%	37.90%	0.26	2.84E-17
MTRNR2L1	HLA-DR+-2	39.60%	19.60%	0.44	4.75E-17
CD4	HLA-DR+-2	33.70%	15.40%	0.31	8.66E-17
OTUD1	HLA-DR+-2	21.80%	7.80%	0.26	1.17E-16
KCNN4	HLA-DR+-2	33.70%	19.30%	0.27	1.42E-16
HSPA6	HLA-DR+-2	21.50%	6.10%	0.43	2.07E-16
TLE4	HLA-DR+-2	47.20%	42.60%	0.26	2.36E-16
IER2	HLA-DR+-2	71.50%	82.10%	0.25	2.49E-16
ANKRD12	HLA-DR+-2	73.60%	70.60%	0.26	3.94E-16
GLUL	HLA-DR+-2	72.00%	76.40%	0.29	4.52E-16
NEDD9	HLA-DR+-2	28.80%	14.80%	0.29	4.94E-16
PLIN2	HLA-DR+-2	39.60%	30.70%	0.40	8.30E-16
ACTR2	HLA-DR+-2	60.60%	51.00%	0.26	1.06E-15
MFSD1	HLA-DR+-2	47.20%	37.40%	0.27	3.02E-15
DNAJA1	HLA-DR+-2	86.00%	90.90%	0.26	3.29E-15
SETD5-AS1	HLA-DR+-2	47.90%	40.30%	0.26	4.11E-15
CA2	HLA-DR+-2	19.40%	6.60%	0.30	7.19E-15
ITGA5	HLA-DR+-2	43.00%	36.00%	0.25	1.00E-14
B4GALT1	HLA-DR+-2	42.70%	34.60%	0.34	2.23E-14
HNRNPA2B1	HLA-DR+-2	96.10%	95.80%	0.26	2.32E-14
ID2	HLA-DR+-2	90.20%	89.60%	0.38	1.69E-13
GTF2B	HLA-DR+-2	51.00%	41.20%	0.25	2.90E-13
KMT2E	HLA-DR+-2	67.40%	58.90%	0.27	4.33E-13
RP11-138A9.2	HLA-DR+-2	30.80%	16.60%	0.28	4.62E-13
S100A11	HLA-DR+-2	99.00%	98.80%	0.25	4.70E-13
CEBPB	HLA-DR+-2	46.90%	43.30%	0.26	1.62E-11
SQSTM1	HLA-DR+-2	93.80%	92.40%	0.28	8.67E-10
FOSB	HLA-DR+-2	80.60%	85.80%	0.27	5.91E-08
MIR24-2	HLA-DR+-2	42.00%	31.10%	0.27	6.86E-08
MAFB	HLA-DR+-2	42.50%	43.40%	0.28	1.46E-07
LSP1	HLA-DR+-2	75.40%	69.70%	0.26	5.46E-07
XIST	HLA-DR+-2	58.30%	44.20%	0.30	8.65E-07
CXCL1	HLA-DR+-2	53.10%	46.10%	0.52	1.52E-06
HBB	HLA-DR+-2	15.50%	7.80%	0.54	4.74E-02

ZFP36	HLA-DR+-2	94.80%	92.30%	0.27	4.25E-01
CCL20	HLA-DR+-2	15.50%	13.30%	0.53	5.77E-01
TM4SF1	EC	99.50%	49.10%	2.57	0.00E+00
PLVAP	EC	87.00%	2.10%	2.31	0.00E+00
DARC	EC	69.40%	1.70%	2.31	0.00E+00
RAMP2	EC	97.70%	30.30%	2.05	0.00E+00
GNG11	EC	98.70%	62.80%	1.99	0.00E+00
EMCN	EC	92.50%	1.00%	1.78	0.00E+00
IFI27	EC	97.90%	59.50%	1.77	0.00E+00
CALCRL	EC	91.50%	5.00%	1.76	0.00E+00
BCAM	EC	93.50%	3.00%	1.75	0.00E+00
ECSCR	EC	90.40%	1.00%	1.62	0.00E+00
GIMAP7	EC	80.10%	3.60%	1.61	0.00E+00
AC011526.1	EC	90.20%	0.90%	1.59	0.00E+00
ELTD1	EC	88.10%	1.00%	1.57	0.00E+00
RAMP3	EC	84.70%	9.20%	1.56	0.00E+00
ESAM	EC	84.70%	3.60%	1.53	0.00E+00
CLEC14A	EC	84.70%	1.40%	1.52	0.00E+00
SPRY1	EC	80.30%	15.10%	1.49	0.00E+00
VWF	EC	75.10%	1.40%	1.42	0.00E+00
SLC9A3R2	EC	64.00%	10.70%	1.38	0.00E+00
PDLIM1	EC	97.20%	48.90%	1.33	0.00E+00
HLA-E	EC	100.00%	90.90%	1.32	0.00E+00
ARHGAP29	EC	82.90%	13.30%	1.29	0.00E+00
NPDC1	EC	80.60%	9.50%	1.26	0.00E+00
HLA-B	EC	100.00%	98.10%	1.25	0.00E+00
CD93	EC	75.60%	2.70%	1.21	0.00E+00
TM4SF18	EC	72.30%	0.40%	1.20	0.00E+00
HYAL2	EC	70.20%	18.80%	1.12	0.00E+00
PODXL	EC	74.10%	4.50%	1.12	0.00E+00
SLCO2A1	EC	66.30%	0.60%	1.09	0.00E+00
CYYR1	EC	68.10%	0.50%	0.98	0.00E+00
PTPRB	EC	67.40%	0.60%	0.95	0.00E+00
TIE1	EC	69.20%	0.50%	0.90	0.00E+00
MYCT1	EC	57.80%	0.20%	0.70	0.00E+00
MGST2	EC	81.10%	15.00%	1.11	7.56912083440904e-310
CD200	EC	63.70%	1.40%	1.10	0.00E+00
CDH5	EC	60.10%	0.60%	0.80	0.00E+00
CXorf36	EC	58.00%	0.50%	0.75	1.62E-305
TSPAN7	EC	76.90%	5.00%	1.05	3.77E-305
SYNE2	EC	84.20%	14.60%	1.26	2.41E-300

HSPG2	EC	85.20%	40.00%	1.12	6.32E-298
B2M	EC	100.00%	100.00%	0.88	2.00E-296
LUZP1	EC	85.80%	40.40%	1.04	1.30E-292
ITM2A	EC	87.80%	21.20%	1.53	1.45E-288
ITGA6	EC	75.40%	10.40%	1.04	2.83E-287
GIMAP5	EC	66.80%	2.60%	1.14	2.32E-284
STC1	EC	53.60%	0.80%	1.72	5.42E-283
SELE	EC	49.50%	2.10%	2.45	1.41E-282
ERG	EC	68.40%	5.70%	0.88	1.92E-280
SOX7	EC	53.60%	0.40%	0.81	3.12E-279
GIMAP6	EC	63.50%	1.70%	0.79	1.23E-271
A2M	EC	88.90%	20.20%	1.62	9.06E-270
JAM2	EC	69.90%	9.60%	0.92	2.52E-264
FKBP1A	EC	95.10%	82.40%	0.89	4.31E-264
ARL4A	EC	79.00%	46.30%	1.11	3.53E-259
TSC22D1	EC	89.10%	52.40%	1.56	3.61E-259
FLT1	EC	55.40%	1.00%	0.76	3.82E-257
HLA-A	EC	100.00%	98.10%	0.97	2.21E-256
MALL	EC	68.10%	10.30%	0.90	1.23E-253
ELK3	EC	81.30%	35.10%	0.93	3.80E-253
ENG	EC	86.80%	53.90%	0.96	1.29E-247
EPAS1	EC	84.50%	25.10%	1.04	1.19E-246
ETS2	EC	88.30%	30.10%	1.21	4.82E-244
WWTR1	EC	73.80%	33.90%	0.89	4.18E-238
IGFBP7	EC	98.70%	63.60%	1.52	2.31E-237
ADAM15	EC	77.20%	29.40%	0.89	5.55E-237
ADAMTS9	EC	58.50%	3.80%	1.16	1.02E-236
KTN1	EC	92.70%	71.00%	0.90	1.52E-235
SPTBN1	EC	83.90%	46.50%	0.94	2.54E-229
BCL6B	EC	45.10%	0.40%	0.48	9.13E-227
PRKCH	EC	63.50%	3.70%	0.74	2.26E-226
F2R	EC	60.60%	3.00%	0.81	3.67E-226
SNHG7	EC	79.50%	34.20%	0.91	1.44E-223
KDR	EC	43.50%	0.30%	0.65	2.54E-222
RDX	EC	86.50%	55.20%	0.88	6.97E-222
NUAK1	EC	58.00%	4.90%	0.80	4.49E-220
FAM198B	EC	72.30%	12.30%	0.92	1.10E-219
SRP14	EC	99.20%	97.60%	0.77	4.26E-217
RND1	EC	57.00%	9.90%	0.98	1.05E-216
EFNA1	EC	58.80%	4.50%	0.78	7.64E-216
MCTP1	EC	64.00%	4.40%	0.89	1.18E-215

EGFL7	EC	53.10%	2.20%	0.97	2.51E-215
ITGB4	EC	46.40%	0.90%	0.63	2.04E-214
IL3RA	EC	57.00%	2.30%	0.71	1.73E-213
MMRN2	EC	46.10%	0.80%	0.66	6.42E-212
PCDH17	EC	46.90%	0.80%	0.57	2.86E-208
CCL14	EC	38.60%	0.10%	0.60	2.13E-207
SWAP70	EC	71.20%	24.10%	0.92	1.10E-206
PVRL2	EC	75.10%	29.60%	0.84	8.01E-206
TCF4	EC	90.40%	65.30%	0.93	8.98E-206
F2RL3	EC	39.90%	0.30%	0.82	3.59E-205
CRIP2	EC	96.90%	74.40%	0.98	5.99E-204
CNKSR3	EC	49.70%	1.90%	0.70	6.36E-203
KCNN3	EC	50.30%	2.10%	0.66	7.48E-203
SLC2A3	EC	80.10%	33.30%	1.03	3.15E-202
MTUS1	EC	76.90%	21.80%	0.98	2.52E-197
PTMA	EC	100.00%	99.90%	0.68	1.61E-194
RHOJ	EC	53.10%	3.60%	0.67	3.28E-194
TINAGL1	EC	56.50%	3.60%	0.71	1.13E-190
HLA-C	EC	99.70%	97.30%	0.84	1.28E-189
S1PR1	EC	50.30%	1.80%	0.57	1.52E-189
MCAM	EC	59.60%	4.60%	0.71	1.57E-189
RAB11A	EC	85.80%	49.30%	0.77	2.68E-188
ABCB1	EC	39.90%	0.40%	0.42	1.04E-187
TPM3	EC	95.30%	71.80%	0.82	3.60E-187
AKR1C3	EC	79.30%	32.90%	1.02	1.49E-186
LCN6.1	EC	35.80%	0.10%	0.47	4.43E-186
GIMAP4	EC	58.00%	3.70%	0.81	1.29E-185
CD34	EC	71.20%	10.90%	0.87	4.27E-185
CLDN5	EC	45.60%	2.00%	1.05	5.78E-184
CDC37	EC	88.90%	65.60%	0.73	6.37E-183
HLA-F	EC	76.90%	23.40%	0.84	1.18E-182
SPARCL1	EC	97.20%	76.00%	1.16	1.74E-177
NEDD9	EC	64.50%	13.50%	0.84	1.84E-177
COL15A1	EC	79.50%	23.10%	1.07	2.54E-177
RPS28	EC	100.00%	99.80%	0.60	5.00E-177
GIMAP1	EC	51.80%	2.60%	0.69	1.19E-175
RBP7	EC	44.00%	1.30%	0.57	1.24E-174
PLAT	EC	54.70%	13.50%	0.84	5.20E-174
CD59	EC	95.90%	81.80%	0.87	5.92E-173
ADCY4	EC	40.40%	0.80%	0.45	1.81E-172
SOX17	EC	33.90%	0.20%	0.58	5.22E-171

ENTPD1	EC	62.20%	6.60%	0.77	7.96E-170
RPS2	EC	100.00%	99.60%	0.73	1.47E-165
RPL36A	EC	99.20%	94.50%	0.82	1.40E-164
CDC42EP3	EC	68.40%	16.30%	1.03	2.43E-164
SERPINE1	EC	68.10%	35.50%	1.20	5.69E-162
ITGA2	EC	36.80%	0.70%	0.46	2.54E-161
GPR56	EC	43.30%	1.60%	0.45	3.40E-161
CLEC2B	EC	71.00%	14.70%	1.04	2.83E-160
ARHGEF15	EC	31.10%	0.10%	0.35	8.47E-160
CAV2	EC	79.80%	37.70%	0.79	5.33E-159
ROBO4	EC	39.60%	1.00%	0.45	5.60E-159
RAPGEF4	EC	46.90%	3.80%	0.52	3.97E-158
ETS1	EC	68.10%	18.30%	0.78	1.02E-156
DUSP23	EC	67.40%	34.90%	0.75	1.28E-156
MEF2C	EC	78.80%	20.00%	0.94	2.80E-155
GPR116	EC	47.40%	2.40%	0.61	5.25E-155
IGF2	EC	44.80%	2.40%	1.00	8.00E-154
PALMD	EC	75.90%	37.10%	0.84	3.02E-152
ABI3	EC	43.00%	1.70%	0.45	4.14E-151
LMCD1	EC	72.50%	22.00%	0.87	6.57E-151
TEK	EC	32.90%	0.40%	0.36	6.65E-151
TMSB10	EC	100.00%	98.00%	0.82	1.89E-149
SPNS2	EC	31.90%	0.30%	0.33	4.49E-149
RASIP1	EC	32.90%	0.40%	0.35	7.44E-149
PRCP	EC	78.00%	48.90%	0.72	1.13E-148
TSPAN13	EC	39.60%	1.50%	0.50	1.41E-146
IFI16	EC	92.50%	68.90%	0.89	9.93E-146
SOX18	EC	28.80%	0.10%	0.33	1.63E-144
MMRN1	EC	27.20%	0.10%	0.52	2.15E-143
CCDC85B	EC	91.20%	68.60%	0.71	4.17E-143
MEOX1	EC	48.40%	4.20%	0.64	1.22E-141
C4orf32	EC	54.40%	6.40%	0.63	1.38E-141
SSFA2	EC	62.40%	13.80%	0.66	2.19E-141
C19orf33	EC	31.10%	0.40%	0.42	1.58E-139
STOM	EC	84.20%	48.10%	0.77	4.86E-139
BMPR2	EC	67.40%	29.20%	0.66	1.55E-138
SEMA6A	EC	32.10%	0.50%	0.36	1.24E-137
AKAP13	EC	86.30%	60.90%	0.76	3.41E-137
NES	EC	43.80%	2.60%	0.51	7.10E-137
ECE1	EC	72.80%	24.20%	0.70	2.34E-135
C10orf128	EC	44.60%	2.90%	0.43	3.90E-135

CD74	EC	95.60%	48.80%	0.54	1.13E-134
EDN1	EC	31.30%	0.80%	0.79	1.26E-134
DLL4	EC	30.80%	0.40%	0.40	1.51E-134
RPS7	EC	99.50%	99.00%	0.52	2.99E-134
MGLL	EC	75.60%	28.40%	0.86	6.64E-134
PKP4	EC	44.60%	4.10%	0.52	1.31E-133
SHROOM4	EC	43.80%	4.00%	0.48	2.40E-133
FAM84B	EC	39.10%	1.90%	0.49	8.89E-133
RALGAPA2	EC	42.50%	2.60%	0.42	1.74E-129
TGFBR2	EC	74.40%	35.00%	0.75	2.87E-129
RPS6	EC	100.00%	99.80%	0.54	3.28E-129
KANK3	EC	26.90%	0.20%	0.30	5.81E-127
CLIC2	EC	66.80%	20.20%	0.67	9.34E-127
GIMAP8	EC	32.90%	1.00%	0.33	2.73E-126
AFAP1L1	EC	29.00%	0.40%	0.33	3.05E-126
EHD4	EC	60.40%	16.40%	0.66	5.85E-126
KRT18	EC	44.30%	3.20%	0.56	3.60E-125
SMAGP	EC	47.40%	5.50%	0.53	5.63E-125
FLI1	EC	52.80%	6.30%	0.54	1.56E-124
RPS19	EC	100.00%	99.50%	0.56	5.90E-123
DOCK9	EC	49.20%	6.00%	0.49	8.40E-123
APOLD1	EC	50.00%	5.60%	0.62	2.79E-122
SMAD1	EC	39.90%	3.50%	0.48	7.06E-122
LDB2	EC	46.60%	5.90%	0.54	7.44E-122
MAGI1	EC	40.90%	3.50%	0.49	1.98E-121
NOTCH4	EC	33.90%	1.70%	0.45	2.91E-121
PSMB8	EC	78.50%	40.70%	0.64	9.70E-121
TMEM204	EC	46.40%	7.30%	0.58	3.70E-120
NDRG1	EC	84.70%	60.10%	0.67	1.18E-119
YPEL2	EC	67.90%	29.00%	0.69	1.98E-119
MYH9	EC	79.30%	42.40%	0.71	2.04E-119
ACTN4	EC	76.40%	50.80%	0.65	2.92E-119
CAV1	EC	98.70%	80.10%	0.86	4.89E-119
IFITM3	EC	99.50%	92.80%	0.67	1.80E-118
C1orf115	EC	24.60%	0.20%	0.26	6.78E-118
COL4A1	EC	68.40%	29.10%	1.02	3.14E-117
ADM5	EC	31.10%	1.20%	0.41	3.30E-116
RAI14	EC	60.40%	17.20%	0.60	3.52E-116
HOXB7	EC	35.20%	1.70%	0.35	7.10E-116
RUNDC3B	EC	26.70%	0.40%	0.29	1.43E-115
CCDC3	EC	41.20%	3.50%	0.45	9.62E-114

ARHGDIB	EC	73.30%	19.60%	0.55	1.17E-113
CNTNAP3B	EC	35.20%	2.20%	0.40	3.18E-113
RPS15A	EC	100.00%	99.60%	0.48	9.63E-113
KIAA0355	EC	56.70%	13.10%	0.58	1.76E-112
ARHGEF3	EC	45.10%	4.50%	0.45	1.78E-112
MBNL1	EC	80.10%	51.10%	0.67	6.58E-112
IL6ST	EC	80.60%	58.90%	0.63	7.84E-112
ATP1A1	EC	85.20%	70.80%	0.61	2.45E-111
PITPNC1	EC	34.50%	1.60%	0.34	4.24E-111
HEG1	EC	69.90%	34.60%	0.64	1.65E-110
RPL26	EC	100.00%	99.70%	0.47	3.41E-110
THBD	EC	77.70%	29.90%	0.92	7.02E-109
CD320	EC	67.90%	31.10%	0.61	2.74E-108
TNFRSF10D	EC	47.20%	11.90%	0.59	7.35E-108
HHEX	EC	43.50%	4.20%	0.43	4.05E-107
GMFG	EC	64.00%	14.70%	0.49	4.75E-107
FAM167B	EC	27.20%	0.80%	0.28	6.71E-107
NRP1	EC	71.20%	25.10%	0.67	5.76E-106
FAM107A	EC	25.60%	0.50%	0.35	6.69E-106
RPL32	EC	100.00%	99.80%	0.49	9.88E-105
RAPGEF3	EC	37.60%	3.00%	0.42	3.16E-104
ANGPT2	EC	40.40%	4.90%	0.94	6.74E-104
DAAM1	EC	65.80%	26.70%	0.68	7.96E-104
EIF4B	EC	88.10%	64.60%	0.62	1.50E-102
PDE2A	EC	31.10%	1.30%	0.33	4.02E-102
CPLX1	EC	20.50%	0.10%	0.30	4.69E-102
RPL36	EC	100.00%	99.20%	0.47	1.17E-101
MKL2	EC	38.60%	4.30%	0.44	2.05E-101
GABRD	EC	20.70%	0.10%	0.26	1.18E-99
COL4A2	EC	66.80%	33.20%	0.88	3.00E-99
GRB10	EC	45.60%	7.80%	0.46	4.32E-99
RPS3	EC	99.50%	99.40%	0.49	1.54E-98
NRGN	EC	33.70%	2.00%	0.33	6.41E-98
MYLK	EC	39.10%	4.50%	0.33	2.09E-97
TMEM2	EC	63.20%	21.90%	0.62	2.77E-97
TAP1	EC	60.90%	24.20%	0.61	4.86E-97
CDH13	EC	63.50%	28.30%	0.70	3.53E-96
VWA1	EC	40.90%	6.60%	0.47	3.54E-96
COL18A1	EC	73.30%	24.90%	0.72	4.05E-96
GPRC5B	EC	24.10%	0.50%	0.25	5.02E-96
APLNR	EC	28.50%	1.10%	0.33	3.84E-95

ZNF366	EC	20.70%	0.20%	0.25	4.08E-95
MAST4	EC	45.10%	8.60%	0.54	1.40E-94
RPS8	EC	100.00%	99.70%	0.51	3.23E-94
RPS18	EC	100.00%	99.70%	0.54	1.46E-93
INSR	EC	48.20%	16.10%	0.64	3.32E-93
LPAR6	EC	48.40%	7.30%	0.48	4.56E-93
SLC44A2	EC	45.90%	9.30%	0.45	7.79E-93
BACE2	EC	44.80%	7.60%	0.43	2.48E-92
ARAP3	EC	25.90%	0.90%	0.27	2.72E-92
PTP4A3	EC	38.90%	4.00%	0.47	2.78E-92
RAPGEF5	EC	22.80%	0.40%	0.27	2.86E-92
AQP1	EC	96.90%	77.40%	0.72	4.11E-92
RASGRP3	EC	37.60%	4.10%	0.31	8.34E-92
GJA1	EC	66.80%	47.10%	0.60	3.76E-91
EDNRB	EC	36.00%	3.40%	0.80	1.75E-90
FRY	EC	35.80%	3.50%	0.39	4.18E-90
SDPR	EC	41.50%	5.40%	0.62	2.90E-89
DOCK6	EC	35.80%	3.40%	0.36	5.31E-89
MYL12A	EC	96.40%	90.80%	0.58	1.17E-88
FZD6	EC	35.50%	3.20%	0.33	2.09E-88
SSH1	EC	44.80%	6.60%	0.44	2.31E-88
NOTCH1	EC	36.80%	3.50%	0.36	2.83E-88
PTPRK	EC	50.30%	9.80%	0.44	8.13E-88
SNCG	EC	53.40%	11.60%	0.62	8.90E-88
TSHZ1	EC	43.00%	7.40%	0.44	1.15E-87
KCNK5	EC	30.30%	2.00%	0.27	3.78E-87
CD79B	EC	34.20%	3.40%	0.39	6.72E-87
FAM60A	EC	45.30%	6.80%	0.40	1.20E-86
PLEKHG1	EC	32.40%	2.40%	0.28	8.71E-86
RASA4	EC	36.80%	4.00%	0.44	1.12E-85
NEURL1B	EC	26.70%	1.20%	0.26	1.21E-85
ACVRL1	EC	36.00%	3.40%	0.33	1.44E-85
EPHA4	EC	28.00%	2.00%	0.32	7.55E-85
NOSTRIN	EC	29.30%	2.50%	0.38	8.11E-85
H3F3A	EC	98.20%	98.30%	0.49	9.93E-85
MFNG	EC	31.90%	2.50%	0.34	1.48E-83
GFOD1	EC	34.20%	3.00%	0.32	1.55E-83
CRHBP	EC	18.90%	0.20%	0.28	2.26E-82
EFNB2	EC	39.60%	6.10%	0.45	8.06E-82
LMO2	EC	36.00%	3.80%	0.39	8.11E-82
HSPA12B	EC	29.30%	2.20%	0.32	8.21E-82

RPL38	EC	99.50%	99.20%	0.40	2.12E-81
SPHK1	EC	67.90%	29.10%	0.60	2.80E-81
PLCB1	EC	34.20%	3.70%	0.34	4.54E-81
POSTN	EC	38.30%	5.60%	0.56	4.65E-81
ASAP1	EC	60.90%	22.70%	0.56	5.32E-81
ELF1	EC	73.10%	51.30%	0.57	6.18E-81
TSPAN14	EC	47.70%	9.40%	0.43	1.82E-80
RPS21	EC	99.70%	98.60%	0.45	2.52E-80
RPL3	EC	100.00%	99.70%	0.46	1.61E-79
OLFML2A	EC	38.60%	4.70%	0.38	1.78E-79
RPL18A	EC	100.00%	99.70%	0.39	2.98E-79
PRDM1	EC	41.50%	5.90%	0.57	5.77E-79
TNFAIP1	EC	42.70%	8.50%	0.38	8.64E-79
RPL34	EC	100.00%	99.90%	0.45	9.34E-79
PREX2	EC	27.50%	1.90%	0.28	1.25E-78
CCDC69	EC	47.40%	10.00%	0.45	1.32E-78
TACSTD2	EC	22.80%	0.80%	0.33	3.84E-78
PSMB9	EC	63.20%	27.00%	0.51	5.38E-78
DOCK4	EC	32.90%	3.20%	0.32	6.90E-78
TNFAIP8L1	EC	30.10%	2.30%	0.30	1.79E-77
IFIT3	EC	46.40%	10.70%	0.61	2.07E-77
NUDT14	EC	41.70%	6.20%	0.38	2.97E-77
GATA2	EC	38.30%	4.80%	0.48	3.75E-77
TXNIP	EC	62.70%	43.50%	0.99	1.08E-76
SPRY4	EC	32.40%	3.30%	0.32	1.27E-76
VAMP5	EC	79.80%	59.10%	0.51	1.31E-76
UPP1	EC	62.70%	26.70%	0.61	2.53E-76
RPL9	EC	99.70%	99.40%	0.40	6.97E-76
NCOA7	EC	65.30%	40.10%	0.61	7.96E-76
RPL18	EC	99.50%	99.20%	0.41	8.56E-76
RPL5	EC	99.70%	99.30%	0.43	1.50E-75
HDAC7	EC	47.20%	10.90%	0.47	1.81E-75
MECOM	EC	27.50%	2.00%	0.30	1.11E-74
PIK3C2A	EC	52.30%	14.30%	0.45	1.36E-74
TESC	EC	23.10%	1.00%	0.26	1.42E-74
COL21A1	EC	30.60%	2.70%	0.32	2.79E-74
NRN1	EC	51.30%	13.50%	0.52	3.26E-74
LPHN2	EC	29.00%	2.30%	0.27	5.76E-74
HECW2	EC	31.10%	3.70%	0.36	6.02E-74
RPL35A	EC	100.00%	99.60%	0.35	2.20E-73
MRPL33	EC	84.70%	72.00%	0.49	2.50E-73

GNAI2	EC	77.50%	55.50%	0.56	2.60E-73
SYNPO	EC	52.60%	18.20%	0.49	4.80E-73
UTRN	EC	61.90%	25.80%	0.56	5.57E-73
IPO11	EC	32.60%	3.80%	0.32	6.70E-73
ARPC5L	EC	58.00%	33.20%	0.46	9.65E-73
IL33	EC	25.40%	1.90%	0.38	2.09E-72
ARID5A	EC	66.10%	42.40%	0.58	3.13E-72
RPL37A	EC	99.70%	99.90%	0.36	5.19E-72
GLTSCR2	EC	92.70%	84.10%	0.54	8.86E-72
LIMS2	EC	46.60%	12.20%	0.42	1.22E-71
LAMA5	EC	30.80%	2.80%	0.29	1.42E-71
SLCO4A1	EC	33.40%	4.90%	0.38	1.95E-71
PNP	EC	65.30%	39.00%	0.57	2.58E-71
KIAA1462	EC	38.60%	7.30%	0.36	4.87E-71
PTPRE	EC	56.70%	18.80%	0.65	9.32E-71
LAYN	EC	27.70%	2.50%	0.28	2.10E-70
ARHGAP31	EC	35.20%	4.30%	0.31	2.37E-70
RPL15	EC	100.00%	99.60%	0.39	4.11E-70
CCL23	EC	15.80%	0.20%	0.35	5.22E-70
TJP1	EC	61.10%	26.80%	0.49	7.13E-70
ACTN1	EC	77.50%	42.00%	0.61	9.76E-70
PGM5	EC	25.60%	1.90%	0.26	1.25E-69
SH2D3C	EC	21.50%	0.80%	0.26	1.80E-69
ZNF385D	EC	55.70%	36.30%	0.51	3.37E-69
TMEM255B	EC	34.20%	4.20%	0.31	4.42E-69
ICAM1	EC	69.40%	38.20%	0.78	4.78E-69
OCIAD2	EC	44.00%	9.70%	0.41	5.68E-69
CRIM1	EC	53.60%	17.80%	0.51	9.43E-69
MYL12B	EC	95.90%	91.40%	0.50	1.15E-68
CNST	EC	46.40%	12.70%	0.44	1.89E-68
SVIL	EC	65.80%	31.60%	0.53	3.01E-68
RPS24	EC	99.70%	99.70%	0.40	4.16E-68
MX1	EC	42.50%	8.30%	0.47	7.65E-68
MAP3K11	EC	43.30%	8.20%	0.40	2.12E-67
MSN	EC	81.30%	69.50%	0.48	2.18E-67
SASH1	EC	58.30%	22.30%	0.49	3.13E-67
RPS13	EC	99.70%	99.50%	0.38	3.72E-67
SKAP2	EC	44.00%	9.10%	0.32	4.32E-67
RPL31	EC	100.00%	99.60%	0.40	2.10E-66
RPL11	EC	99.70%	99.90%	0.35	6.31E-66
BCAR1	EC	47.20%	13.60%	0.41	1.57E-65

RAB13	EC	82.40%	62.70%	0.53	9.47E-65
SNRK	EC	42.20%	8.70%	0.38	1.06E-64
DUSP6	EC	56.00%	17.00%	0.62	2.02E-63
SERBP1	EC	89.90%	83.40%	0.45	2.48E-63
ICAM2	EC	42.50%	13.80%	0.40	1.26E-62
PLXNA2	EC	45.10%	10.50%	0.40	2.69E-62
RPS9	EC	99.70%	99.60%	0.34	3.13E-62
PLXND1	EC	44.30%	9.60%	0.41	1.34E-61
TNFSF10	EC	59.10%	22.80%	0.56	4.55E-61
JAK1	EC	72.80%	50.60%	0.49	4.94E-61
ADAMTS4	EC	45.10%	10.30%	0.45	7.62E-61
CPXM2	EC	30.10%	4.20%	0.33	1.28E-60
LRRFIP1	EC	88.60%	63.60%	0.55	1.72E-60
HIPK3	EC	61.40%	28.50%	0.47	2.41E-60
RPL29	EC	99.70%	99.20%	0.39	3.08E-60
ATP11C	EC	39.90%	9.00%	0.36	3.44E-60
RPS29	EC	99.50%	99.40%	0.36	4.92E-60
RPGR	EC	40.40%	9.20%	0.37	1.07E-59
TSPAN9	EC	33.90%	5.70%	0.29	1.85E-59
ARGLU1	EC	75.40%	52.50%	0.49	3.87E-59
RPL37	EC	100.00%	99.70%	0.39	4.11E-59
VGLL4	EC	64.50%	39.70%	0.48	5.36E-59
IFITM2	EC	97.20%	87.60%	0.43	6.94E-59
RPL8	EC	100.00%	99.60%	0.35	1.09E-58
RPS3A	EC	100.00%	99.60%	0.36	1.91E-58
SP100	EC	72.00%	44.00%	0.48	2.31E-58
CYTH1	EC	54.10%	17.50%	0.46	7.80E-58
RPL13	EC	100.00%	99.90%	0.35	8.56E-58
ADIRF	EC	96.60%	88.40%	0.69	2.50E-57
LAP3	EC	61.70%	30.00%	0.45	2.71E-57
ITGA9	EC	32.10%	4.90%	0.30	4.86E-57
GPR68	EC	26.70%	3.30%	0.27	7.18E-57
AGRN	EC	30.60%	4.20%	0.31	1.84E-56
RPS25	EC	99.70%	99.80%	0.32	3.62E-56
ATP8B1	EC	24.90%	2.50%	0.26	4.08E-56
TMOD3	EC	66.10%	38.20%	0.45	1.12E-55
ITPR1	EC	36.80%	7.10%	0.32	2.04E-55
RPS27A	EC	100.00%	99.80%	0.32	2.93E-55
RILPL2	EC	64.80%	31.00%	0.52	3.29E-55
IL32	EC	43.50%	11.30%	0.70	6.19E-55
RPL21	EC	100.00%	99.90%	0.31	7.92E-55

GBP4	EC	25.60%	2.80%	0.26	2.44E-54
RPL22	EC	100.00%	99.10%	0.36	2.81E-54
RPS15	EC	100.00%	99.80%	0.31	2.99E-54
VPS37B	EC	48.20%	13.00%	0.38	3.50E-54
PTPRM	EC	43.50%	13.80%	0.37	2.68E-53
PPFIBP1	EC	66.80%	37.20%	0.47	3.07E-53
CTTNBP2NL	EC	45.60%	13.00%	0.38	2.85E-52
FRMD4A	EC	31.60%	5.00%	0.27	4.03E-52
IFIT1	EC	33.40%	8.90%	0.43	1.20E-51
PRMT1	EC	72.50%	52.20%	0.43	1.81E-51
ITGA10	EC	52.80%	17.10%	0.48	2.51E-51
KBTBD2	EC	48.70%	16.50%	0.38	3.50E-51
LRRC8C	EC	50.00%	17.60%	0.44	3.88E-51
BTF3	EC	99.70%	97.70%	0.34	4.42E-51
CPNE8	EC	49.00%	18.90%	0.40	4.51E-51
FBXO34	EC	36.80%	9.00%	0.35	7.72E-51
EIF4G2	EC	92.50%	88.20%	0.40	9.87E-51
LIFR	EC	38.60%	14.60%	0.38	1.14E-50
EIF2S2	EC	81.90%	68.60%	0.45	2.98E-50
CFLAR	EC	71.00%	38.50%	0.52	2.98E-50
SEC14L1	EC	55.40%	23.30%	0.41	5.21E-50
CCND1	EC	73.60%	47.90%	0.59	5.41E-50
NACA	EC	99.70%	99.10%	0.33	7.21E-50
EEF1A1	EC	100.00%	100.00%	0.29	8.46E-50
SYNGR2	EC	52.60%	17.70%	0.44	1.56E-49
PGF	EC	44.80%	13.60%	0.55	1.63E-49
INPP1	EC	46.10%	14.20%	0.33	2.82E-49
GFOD2	EC	39.10%	10.60%	0.30	5.82E-49
ARL15	EC	25.10%	4.20%	0.28	9.55E-49
CRK	EC	61.70%	34.00%	0.42	2.61E-48
EVA1C	EC	36.30%	9.00%	0.33	3.14E-48
HEY1	EC	23.60%	2.90%	0.35	5.05E-48
TJP2	EC	36.30%	8.20%	0.29	5.12E-48
FLNB	EC	43.80%	13.90%	0.37	6.80E-48
RAC1	EC	70.50%	55.70%	0.41	9.05E-48
APOL3	EC	38.10%	9.60%	0.33	1.61E-47
ANKRD28	EC	70.70%	58.60%	0.50	1.67E-47
RPS14	EC	100.00%	99.50%	0.33	2.64E-47
RPL19	EC	99.70%	99.80%	0.28	3.37E-47
BCR	EC	32.40%	6.00%	0.26	4.28E-47
RBPMS	EC	42.20%	11.00%	0.31	6.26E-47

HOXD8	EC	36.80%	11.90%	0.33	1.43E-46
FNBP1L	EC	29.50%	5.20%	0.26	2.81E-46
ZFP36L2	EC	79.50%	57.00%	0.55	3.28E-46
CCDC50	EC	60.10%	26.90%	0.42	3.43E-46
APP	EC	91.20%	78.50%	0.49	3.72E-46
KLF2	EC	45.30%	15.70%	0.50	4.61E-46
FGD5	EC	40.20%	12.00%	0.33	5.47E-46
RPL30	EC	100.00%	99.60%	0.30	6.53E-46
FABP5	EC	51.60%	20.90%	0.80	9.68E-46
GRAMD1A	EC	31.90%	6.00%	0.28	1.57E-45
LAPTM4B	EC	36.00%	10.30%	0.31	2.29E-45
TMEM70	EC	56.00%	33.50%	0.41	3.19E-45
YBX1	EC	91.20%	81.70%	0.52	5.39E-45
C19orf43	EC	85.00%	76.20%	0.37	6.44E-45
RPLP2	EC	100.00%	99.90%	0.27	1.08E-44
RPS4X	EC	100.00%	99.80%	0.34	1.43E-44
ATP1B3	EC	79.80%	70.00%	0.44	1.44E-44
TAGLN2	EC	98.40%	96.10%	0.38	1.98E-44
RPL35	EC	99.50%	99.50%	0.30	2.09E-44
SET	EC	79.50%	57.80%	0.49	4.05E-44
CTSH	EC	58.00%	22.70%	0.34	6.46E-44
MARCKSL1	EC	50.50%	17.40%	0.40	1.05E-43
RPL24	EC	99.50%	99.30%	0.30	1.30E-43
RPS10	EC	96.40%	96.10%	0.36	2.77E-43
FAM65A	EC	46.90%	16.00%	0.35	3.94E-43
TNFRSF10B	EC	45.10%	15.60%	0.35	5.05E-43
PTRF	EC	94.30%	79.00%	0.47	9.31E-43
MACF1	EC	61.90%	34.20%	0.42	1.79E-42
ADAR	EC	53.10%	25.00%	0.38	2.23E-42
RALGDS	EC	43.30%	12.60%	0.34	2.31E-42
CTSC	EC	44.30%	13.20%	0.39	4.02E-42
FXYD5	EC	95.10%	82.00%	0.38	4.35E-42
JAG1	EC	39.90%	12.10%	0.37	5.90E-42
HNRNPA3	EC	86.30%	76.10%	0.39	6.56E-42
RPL27A	EC	100.00%	99.60%	0.28	7.75E-42
SLFN5	EC	37.80%	10.70%	0.33	1.04E-41
RPS5	EC	99.50%	99.10%	0.31	1.61E-41
HNRNPF	EC	78.80%	66.20%	0.41	2.32E-41
PLK2	EC	56.00%	25.90%	0.53	2.49E-41
KIAA1551	EC	37.30%	9.40%	0.30	2.73E-41
XRN2	EC	71.00%	48.30%	0.40	3.10E-41

ZNF462	EC	27.20%	5.00%	0.26	4.32E-41
EMP1	EC	93.80%	87.10%	0.49	4.55E-41
RPL6	EC	100.00%	99.60%	0.28	4.60E-41
CARD8	EC	32.40%	7.90%	0.31	6.47E-41
KMT2E	EC	80.10%	58.40%	0.44	1.37E-40
FBL	EC	72.00%	52.50%	0.39	6.12E-40
FLOT1	EC	78.80%	61.20%	0.37	1.25E-39
ID1	EC	63.20%	48.60%	0.65	1.38E-39
U2AF1	EC	82.90%	76.90%	0.37	1.65E-39
KIFC3	EC	37.30%	13.60%	0.31	4.12E-39
IFI44L	EC	30.60%	6.50%	0.28	4.70E-39
SNRPB	EC	80.60%	76.10%	0.34	5.11E-39
RPL12	EC	100.00%	99.60%	0.32	8.17E-39
YES1	EC	44.30%	18.40%	0.33	8.28E-39
RPS12	EC	100.00%	99.80%	0.31	1.00E-38
GSN	EC	97.70%	92.10%	0.38	2.12E-38
RPS23	EC	100.00%	99.80%	0.30	2.37E-38
RPL7	EC	100.00%	99.70%	0.29	2.42E-38
RGL2	EC	52.10%	26.30%	0.33	2.78E-38
S100A16	EC	75.60%	46.50%	0.43	6.88E-38
TENC1	EC	41.50%	16.60%	0.33	8.16E-38
YWHAE	EC	89.60%	83.60%	0.35	1.06E-37
PICALM	EC	58.50%	30.70%	0.39	1.57E-37
CLDN11	EC	17.10%	1.90%	0.27	3.31E-37
MLLT4	EC	37.30%	11.70%	0.28	3.36E-37
RPS27	EC	100.00%	99.90%	0.26	3.88E-37
SNTB2	EC	50.80%	25.20%	0.35	5.57E-37
SH2B3	EC	41.70%	13.60%	0.26	8.59E-37
BNIP2	EC	65.00%	39.40%	0.38	1.21E-36
CDK17	EC	37.60%	10.60%	0.28	1.26E-36
RPL27	EC	100.00%	99.10%	0.28	4.26E-36
SDE2	EC	34.20%	11.00%	0.27	6.47E-36
FRMD4B	EC	59.60%	26.40%	0.41	6.95E-36
LYAR	EC	53.90%	26.50%	0.36	7.53E-36
SVIP	EC	47.40%	19.60%	0.35	8.97E-36
PCGF2	EC	44.00%	19.10%	0.30	1.32E-35
TSHZ2	EC	74.90%	47.40%	0.49	1.68E-35
FRYL	EC	38.60%	12.80%	0.28	1.69E-35
PARP14	EC	45.10%	17.40%	0.33	1.90E-35
OLFM1	EC	35.20%	13.10%	0.29	1.99E-35
GNB2L1	EC	99.70%	99.10%	0.30	2.03E-35

DDX3X	EC	82.90%	69.60%	0.41	2.22E-35
VAPA	EC	84.50%	77.90%	0.34	3.02E-35
TMEM173	EC	72.00%	51.00%	0.38	4.26E-35
HSD17B11	EC	67.60%	43.80%	0.39	5.86E-35
XAF1	EC	33.40%	9.30%	0.28	7.83E-35
RPL23	EC	99.70%	99.10%	0.29	1.75E-34
KAT6A	EC	45.90%	17.50%	0.31	2.39E-34
HNRNPA1	EC	98.70%	96.60%	0.33	2.91E-34
TRIM25	EC	38.60%	12.30%	0.29	1.40E-33
PTPN12	EC	57.00%	38.60%	0.35	2.10E-33
PIM3	EC	52.10%	23.90%	0.43	2.23E-33
CRBN	EC	46.60%	23.20%	0.30	2.23E-33
PON2	EC	46.90%	21.70%	0.31	2.62E-33
CMIP	EC	48.40%	20.80%	0.34	3.04E-33
PTPN14	EC	46.60%	22.70%	0.33	3.38E-33
SLC12A2	EC	37.60%	12.80%	0.30	3.63E-33
TBC1D1	EC	39.40%	13.20%	0.30	4.21E-33
SDCBP	EC	94.80%	90.50%	0.41	4.50E-33
C8orf4	EC	36.80%	11.30%	0.31	6.43E-33
RGS16	EC	77.20%	57.00%	0.63	1.02E-32
DNAJB4	EC	59.80%	33.80%	0.41	1.89E-32
ZNF267	EC	52.60%	23.90%	0.40	2.80E-32
NET1	EC	42.50%	20.40%	0.33	3.35E-32
SLK	EC	50.30%	25.20%	0.33	4.73E-32
RPL17	EC	89.40%	80.00%	0.36	5.36E-32
NKTR	EC	56.00%	29.40%	0.39	7.89E-32
WNK1	EC	60.90%	39.70%	0.35	8.07E-32
MEF2A	EC	66.60%	42.50%	0.38	1.50E-31
LAMB1	EC	39.40%	13.50%	0.31	1.63E-31
DDX21	EC	77.50%	72.90%	0.36	5.29E-31
ANKRD11	EC	59.10%	42.80%	0.36	5.49E-31
RASAL2	EC	34.50%	11.30%	0.25	1.11E-30
CDC42EP2	EC	37.80%	12.70%	0.29	1.70E-30
SUN1	EC	46.40%	24.60%	0.32	2.43E-30
ABCD4	EC	45.60%	20.10%	0.30	5.93E-30
GNAS	EC	84.20%	74.80%	0.34	1.39E-29
MTRNR2L1	EC	45.90%	19.40%	0.72	1.43E-29
LAMA4	EC	64.00%	41.90%	0.40	2.20E-29
UACA	EC	63.50%	37.90%	0.40	2.26E-29
RPL10A	EC	100.00%	99.30%	0.28	2.84E-29
WARS	EC	46.40%	24.40%	0.33	2.97E-29

RBM8A	EC	83.90%	78.50%	0.32	3.01E-29
RAP1A	EC	79.80%	62.40%	0.35	5.07E-29
PIK3R3	EC	40.20%	18.30%	0.30	6.02E-29
KLF6	EC	87.80%	80.10%	0.43	6.21E-29
RNF19A	EC	44.80%	19.30%	0.33	6.45E-29
YBX3	EC	77.70%	57.00%	0.35	9.78E-29
FEZ2	EC	64.50%	40.40%	0.33	2.37E-28
NBEAL1	EC	86.50%	79.10%	0.34	5.76E-28
ITGA5	EC	61.10%	35.30%	0.38	1.41E-27
FUBP1	EC	53.60%	28.90%	0.30	2.00E-27
RPL4	EC	97.20%	97.80%	0.25	2.86E-27
PERP	EC	38.30%	21.30%	0.36	4.69E-27
RHOC	EC	88.60%	79.80%	0.37	5.85E-27
UBE2D3	EC	85.20%	85.00%	0.27	1.21E-26
ZEB1	EC	56.00%	33.10%	0.35	1.33E-26
TNPO1	EC	52.80%	30.30%	0.32	2.75E-26
C10orf10	EC	15.50%	9.00%	0.37	3.77E-26
SOCS2	EC	40.40%	17.80%	0.29	5.26E-26
MSRB3	EC	46.10%	24.00%	0.29	6.61E-26
AP1S2	EC	68.10%	39.10%	0.35	8.13E-26
IL13RA2	EC	19.90%	6.90%	0.30	1.00E-25
SHC1	EC	50.30%	25.50%	0.28	1.37E-25
SON	EC	93.00%	89.30%	0.29	1.56E-25
NR2F2	EC	39.10%	14.70%	0.33	1.98E-25
SLC3A2	EC	81.30%	75.70%	0.40	2.90E-25
HES1	EC	65.00%	48.20%	0.61	3.23E-25
MOB2	EC	51.80%	27.30%	0.28	3.52E-25
ODC1	EC	37.00%	14.70%	0.27	3.86E-25
HMOX1	EC	60.60%	56.00%	0.59	1.35E-24
TCEB1	EC	80.10%	78.90%	0.27	1.50E-24
HAPLN3	EC	43.00%	17.80%	0.30	2.04E-24
MBNL2	EC	45.90%	21.60%	0.27	2.19E-24
SNAI1	EC	56.00%	32.90%	0.39	3.67E-24
CCND3	EC	47.20%	22.60%	0.29	3.85E-24
SELP	EC	29.50%	10.40%	0.30	5.79E-24
NASP	EC	69.90%	55.30%	0.35	1.21E-23
RPL7A	EC	99.70%	99.30%	0.25	1.52E-23
PLA2G16	EC	60.60%	35.00%	0.32	1.62E-23
PRPF4B	EC	62.40%	42.10%	0.32	1.95E-23
UBXN1	EC	82.60%	72.10%	0.29	2.29E-23
HMGB1	EC	98.70%	96.80%	0.28	3.44E-23

UBE2J1	EC	56.00%	37.40%	0.29	3.74E-23
RAB5C	EC	75.90%	66.30%	0.29	4.54E-23
FABP4	EC	12.40%	1.50%	0.53	8.08E-23
ELF2	EC	56.70%	35.00%	0.31	8.46E-23
CSNK1A1	EC	79.30%	74.20%	0.29	8.84E-23
SNHG8	EC	79.80%	71.40%	0.32	9.64E-23
ERBB2IP	EC	43.50%	19.90%	0.26	9.91E-23
PAK2	EC	66.10%	45.20%	0.31	1.13E-22
MYCBP2	EC	63.50%	39.50%	0.32	1.30E-22
FILIP1	EC	41.70%	18.60%	0.35	1.49E-22
HSPB1	EC	98.20%	94.40%	0.34	1.60E-22
SLC25A25	EC	37.60%	20.20%	0.28	1.65E-22
SEPW1	EC	82.40%	75.80%	0.30	2.48E-22
RORA	EC	60.60%	44.30%	0.34	4.65E-22
PHF14	EC	54.40%	33.50%	0.30	8.40E-22
GUK1	EC	93.80%	90.60%	0.27	1.78E-21
FOSL2	EC	57.30%	39.30%	0.33	2.05E-21
GBP2	EC	51.00%	35.80%	0.27	2.58E-21
PLSCR1	EC	62.20%	41.20%	0.34	2.67E-21
DLC1	EC	59.10%	42.40%	0.32	3.38E-21
CTNNB1	EC	75.10%	61.20%	0.38	3.74E-21
C9orf3	EC	62.70%	40.60%	0.31	3.77E-21
ODF2L	EC	43.30%	22.50%	0.26	4.67E-21
LRRC8A	EC	40.20%	18.90%	0.26	5.04E-21
MAP1LC3B	EC	86.00%	82.40%	0.27	6.46E-21
MYO6	EC	45.30%	25.60%	0.26	6.49E-21
OAZ2	EC	68.70%	58.60%	0.28	1.90E-20
POLE4	EC	64.20%	46.50%	0.29	2.20E-20
SCAF11	EC	69.70%	56.50%	0.31	3.37E-20
SAV1	EC	43.80%	24.50%	0.27	8.32E-20
XIST	EC	59.30%	44.20%	0.44	1.43E-19
SPAG9	EC	74.10%	61.20%	0.32	1.62E-19
PTPRG	EC	41.70%	22.00%	0.26	1.78E-19
FAM13C	EC	38.60%	17.00%	0.27	1.82E-19
SETD5	EC	49.20%	26.60%	0.26	1.89E-19
ABCE1	EC	49.50%	28.60%	0.27	1.95E-19
ATRX	EC	76.90%	57.10%	0.30	2.72E-19
LIMS1	EC	69.70%	58.70%	0.29	3.07E-19
TAPBP	EC	64.50%	48.90%	0.29	3.72E-19
MSX1	EC	36.00%	16.30%	0.25	4.53E-19
RNPS1	EC	67.40%	52.80%	0.27	4.90E-19

SERTAD4-AS1	EC	48.40%	26.90%	0.28	8.19E-19
ARID4B	EC	74.60%	61.60%	0.31	1.09E-18
MIDN	EC	67.10%	53.00%	0.33	1.26E-18
TPM4	EC	88.10%	80.30%	0.29	1.59E-18
CBLB	EC	40.20%	20.10%	0.26	1.82E-18
IRF7	EC	43.00%	23.70%	0.27	2.05E-18
CALM1	EC	97.70%	96.80%	0.30	3.67E-18
CEBPG	EC	49.20%	29.90%	0.27	3.79E-18
FOXP1	EC	74.60%	63.70%	0.30	6.28E-18
RCAN1	EC	72.00%	51.50%	0.42	6.63E-18
GNAQ	EC	52.10%	28.30%	0.27	1.33E-17
PRRC2C	EC	78.50%	73.10%	0.25	2.01E-17
GNG5	EC	88.90%	81.70%	0.27	2.71E-17
ATP2B1	EC	73.30%	49.00%	0.27	3.28E-17
NAP1L1	EC	90.90%	87.80%	0.26	4.00E-17
ARHGEF12	EC	50.30%	30.70%	0.26	4.67E-17
PLS3	EC	73.10%	54.70%	0.32	6.17E-17
ISG15	EC	52.80%	29.80%	0.33	7.13E-17
CTNNA1	EC	70.20%	58.90%	0.26	1.08E-16
DDX17	EC	68.40%	53.80%	0.29	1.16E-16
DDX46	EC	69.20%	54.10%	0.28	1.17E-16
PLEKHB2	EC	53.60%	32.30%	0.28	1.46E-16
C9orf16	EC	76.40%	67.70%	0.26	1.77E-16
G3BP1	EC	61.40%	44.20%	0.27	4.00E-16
EGLN2	EC	51.30%	32.10%	0.25	6.72E-16
PLIN2	EC	52.10%	30.20%	0.28	7.28E-16
POLR1D	EC	71.50%	54.90%	0.25	2.70E-15
ARPP19	EC	52.60%	35.70%	0.25	3.36E-15
PPP2R2A	EC	54.90%	36.50%	0.25	4.60E-15
BAZ1A	EC	69.90%	56.50%	0.30	1.03E-14
SRSF11	EC	74.60%	64.20%	0.26	5.11E-14
RN7SL1	EC	27.50%	10.80%	0.26	6.68E-14
FKBP9	EC	62.20%	40.70%	0.27	1.25E-13
MTRNR2L8	EC	69.70%	64.10%	0.36	1.28E-13
DNTTIP2	EC	62.20%	41.60%	0.26	2.49E-13
N4BP2L2	EC	74.40%	61.00%	0.27	2.56E-13
CLIC4	EC	65.80%	53.40%	0.25	7.24E-13
SKIL	EC	67.90%	52.40%	0.29	1.38E-12
C1orf54	EC	45.10%	29.50%	0.27	2.30E-12
TRIB1	EC	47.70%	28.70%	0.27	3.09E-12
TAF1D	EC	69.90%	55.40%	0.26	5.58E-12

HMGN1	EC	72.80%	60.40%	0.26	5.77E-12
TXNRD1	EC	49.00%	32.00%	0.29	2.29E-11
VMP1	EC	65.80%	59.30%	0.26	2.84E-11
MTRNR2L2	EC	79.80%	80.70%	0.39	4.97E-09
HSPA1A	EC	71.00%	67.40%	0.37	1.14E-08
DNAJA1	EC	92.20%	90.70%	0.26	1.53E-08
PDLIM3	EC	69.40%	54.20%	0.26	1.62E-07
IGFBP3	EC	14.00%	6.00%	0.25	3.43E-07
7SK.2	EC	26.40%	20.30%	0.33	4.15E-06
ADAMTS1	EC	59.80%	42.40%	0.26	5.82E-06
SPP1	EC	15.80%	9.50%	0.41	7.03E-06
HSPA1B	EC	57.30%	53.80%	0.27	1.48E-04
RGS5	SMC	95.10%	4.90%	3.44	0.00E+00
ACTA2	SMC	93.10%	16.40%	3.19	0.00E+00
TAGLN	SMC	99.70%	38.80%	2.78	0.00E+00
TPM2	SMC	96.00%	43.70%	2.23	0.00E+00
IGFBP7	SMC	99.70%	63.70%	2.10	0.00E+00
MYL9	SMC	97.10%	64.20%	1.93	0.00E+00
COL4A1	SMC	82.20%	28.80%	1.84	0.00E+00
COL18A1	SMC	93.70%	24.40%	1.74	0.00E+00
COL4A2	SMC	86.00%	32.70%	1.68	0.00E+00
TPM1	SMC	92.80%	41.50%	1.61	0.00E+00
PPP1R14A	SMC	73.40%	0.80%	1.58	0.00E+00
TINAGL1	SMC	80.80%	3.00%	1.58	0.00E+00
CALD1	SMC	100.00%	83.60%	1.55	0.00E+00
NOTCH3	SMC	84.00%	5.40%	1.47	0.00E+00
MAP1B	SMC	88.80%	25.70%	1.40	1.53E-292
MYLK	SMC	70.20%	3.60%	1.27	6.94E-290
GJA4	SMC	63.90%	1.00%	1.11	1.60E-289
MCAM	SMC	72.50%	4.30%	1.43	6.20E-287
A2M	SMC	93.40%	20.30%	1.64	1.03E-280
ADAMTS4	SMC	65.60%	9.70%	1.38	2.00E-269
COX4I2	SMC	54.70%	0.70%	1.02	2.08E-255
EPS8	SMC	83.10%	45.50%	0.97	7.90E-252
CHN1	SMC	61.90%	2.90%	0.99	4.71E-242
CSRP1	SMC	77.90%	56.40%	1.01	4.94E-242
MYH11	SMC	49.60%	1.90%	1.70	7.74E-220
LPP	SMC	80.80%	43.60%	0.93	7.76E-213
NR2F2	SMC	65.30%	13.90%	1.02	8.27E-208
ITGA7	SMC	50.40%	1.60%	0.69	4.11E-206
PPP1R12A	SMC	77.70%	46.90%	0.90	9.68E-206

LGI4	SMC	58.50%	5.70%	1.07	6.35E-203
SEP4	SMC	54.40%	3.10%	0.76	1.52E-194
SEP4	SMC	44.10%	2.30%	0.96	5.33E-184
GPR116	SMC	54.20%	2.40%	0.79	8.10E-183
SYNPO2	SMC	54.40%	5.70%	0.88	7.09E-180
HIGD1B	SMC	38.70%	0.70%	0.85	1.68E-174
ABCC9	SMC	52.40%	3.10%	0.77	4.90E-170
EPAS1	SMC	76.80%	25.60%	0.94	5.93E-168
CNN1	SMC	39.00%	1.00%	1.09	1.29E-167
ITGA1	SMC	57.90%	10.40%	0.78	4.62E-164
SPARCL1	SMC	100.00%	75.90%	1.19	7.50E-164
MYO1B	SMC	65.60%	27.10%	0.77	2.87E-163
KCNE4	SMC	63.30%	21.30%	1.22	5.69E-162
C20orf27	SMC	58.70%	26.30%	0.72	5.99E-162
CDH6	SMC	35.50%	0.30%	0.52	7.16E-159
FAM162B	SMC	34.10%	0.30%	0.50	7.91E-155
CCDC102B	SMC	44.70%	2.30%	0.66	5.81E-154
SEPT7	SMC	95.40%	82.70%	0.81	2.01E-153
PLN	SMC	34.70%	0.60%	0.97	1.02E-147
ANGPT2	SMC	53.90%	4.60%	1.03	1.65E-146
SSTR2	SMC	37.20%	1.40%	0.72	1.83E-142
ISYNA1	SMC	53.90%	9.40%	0.75	9.09E-142
GJC1	SMC	39.00%	2.20%	0.55	4.05E-138
MYH9	SMC	79.90%	42.50%	0.79	3.50E-136
CD36	SMC	47.60%	4.40%	1.18	3.61E-134
NDRG2	SMC	64.80%	23.20%	0.71	1.02E-130
EDNRB	SMC	46.10%	3.20%	0.95	2.43E-130
SGIP1	SMC	46.10%	6.00%	0.62	5.66E-129
GUCY1A3	SMC	51.90%	5.90%	0.69	3.47E-127
ENPEP	SMC	30.10%	0.40%	0.42	3.73E-127
ESAM	SMC	50.40%	5.10%	0.58	1.85E-126
ITGB1	SMC	97.10%	86.60%	0.72	5.06E-126
STOM	SMC	82.80%	48.30%	0.78	9.97E-125
KCNJ8	SMC	35.20%	1.70%	0.55	3.23E-123
GGT5	SMC	67.60%	13.30%	0.95	2.28E-119
MRVI1	SMC	29.20%	0.40%	0.42	4.44E-118
EFHD1	SMC	32.40%	1.50%	0.46	6.24E-118
MTUS1	SMC	59.60%	22.60%	0.83	2.14E-113
CPE	SMC	48.70%	6.10%	0.77	6.11E-112
MIR4435-1HG	SMC	69.30%	42.40%	0.94	4.99E-109
FLNA	SMC	79.10%	57.20%	0.84	1.03E-108

THY1	SMC	78.50%	57.40%	1.12	2.09E-108
PGF	SMC	59.00%	13.20%	0.82	5.15E-107
PPP1R12B	SMC	35.50%	5.30%	0.58	5.22E-107
EBF2	SMC	45.30%	5.40%	0.53	1.02E-105
SLC25A5	SMC	91.10%	80.60%	0.64	1.09E-104
CYP26B1	SMC	34.40%	3.60%	0.59	2.17E-104
PALLD	SMC	62.80%	27.00%	0.65	3.32E-103
FBLIM1	SMC	39.00%	3.50%	0.50	5.14E-103
GUCY1B3	SMC	40.40%	3.40%	0.49	6.68E-103
MT1A	SMC	74.80%	45.20%	1.40	8.93E-103
ACTN4	SMC	73.40%	51.00%	0.65	1.72E-102
FAM213A	SMC	40.70%	5.90%	0.60	1.34E-101
CYFIP2	SMC	27.80%	1.40%	0.54	7.80E-101
SCN4B	SMC	25.20%	0.50%	0.35	2.19E-100
FILIP1L	SMC	77.90%	42.30%	0.89	2.21E-100
OAZ2	SMC	73.90%	58.40%	0.56	3.12E-100
PARM1	SMC	38.10%	4.00%	0.49	7.04E-100
DNAJB4	SMC	56.70%	34.00%	0.68	7.58E-100
TGFB1I1	SMC	66.20%	37.50%	0.64	8.23E-99
AOC3	SMC	31.80%	3.10%	0.58	9.67E-99
ADIRF	SMC	99.40%	88.30%	0.95	2.57E-98
FHL5	SMC	21.80%	0.10%	0.38	5.11E-98
RBPMS	SMC	45.80%	11.00%	0.54	7.90E-97
GUCY1A2	SMC	28.10%	0.90%	0.35	3.52E-95
LHFP	SMC	91.40%	64.00%	0.78	2.00E-94
CRISPLD2	SMC	66.20%	29.80%	0.85	8.05E-94
NCKAP5	SMC	26.90%	0.80%	0.41	2.66E-93
ARHGAP15	SMC	36.10%	2.40%	0.47	7.58E-92
VCL	SMC	60.70%	31.60%	0.58	1.01E-91
BCAM	SMC	44.40%	5.00%	0.76	2.36E-91
RP11-332H18.4	SMC	22.10%	0.30%	0.34	6.87E-91
PTK2	SMC	43.00%	14.90%	0.55	1.31E-90
LINC00152	SMC	72.50%	47.40%	0.83	3.34E-90
CSRP2	SMC	49.60%	14.30%	0.74	9.64E-90
KRT18	SMC	36.10%	3.60%	0.65	1.25E-89
CPM	SMC	55.30%	10.00%	0.72	1.56E-89
PHLDA1	SMC	83.40%	64.50%	0.88	2.05E-88
UTRN	SMC	63.00%	25.80%	0.64	2.54E-88
TFPI	SMC	73.10%	45.10%	0.69	5.77E-88
LPL	SMC	29.50%	1.80%	0.54	8.93E-86
FRZB	SMC	36.40%	3.40%	0.63	1.96E-85

PTMA	SMC	100.00%	99.90%	0.50	2.75E-85
CCL8	SMC	32.10%	4.60%	1.32	2.35E-84
PDE5A	SMC	38.70%	6.60%	0.51	5.51E-84
LPPR4	SMC	20.30%	0.30%	0.29	8.80E-83
DDX21	SMC	80.50%	72.80%	0.63	6.42E-82
RNF152	SMC	29.80%	2.10%	0.43	7.97E-82
MAP2	SMC	24.40%	0.70%	0.31	1.84E-81
ID4	SMC	44.10%	14.40%	0.57	9.05E-81
RARRES2	SMC	57.30%	12.30%	0.71	2.12E-80
PDLIM1	SMC	85.10%	49.50%	0.72	8.51E-80
UBA2	SMC	65.30%	39.50%	0.52	1.52E-79
EDNRA	SMC	28.70%	1.90%	0.39	2.71E-78
TPM4	SMC	92.30%	80.20%	0.60	1.58E-77
FABP4	SMC	25.80%	1.00%	0.76	1.82E-77
NEXN	SMC	41.00%	11.10%	0.60	8.93E-76
PTP4A3	SMC	35.50%	4.20%	0.49	1.26E-75
PAWR	SMC	26.10%	1.60%	0.33	1.34E-75
COL5A3	SMC	38.40%	5.20%	0.47	2.31E-75
ARHGDIB	SMC	67.60%	20.00%	0.76	2.54E-75
ACTN1	SMC	77.40%	42.10%	0.66	3.29E-74
ARHGEF17	SMC	33.00%	4.70%	0.39	9.30E-74
MLTK	SMC	62.50%	37.30%	0.53	1.59E-73
INPP4B	SMC	28.10%	3.50%	0.39	2.47E-73
UACA	SMC	65.00%	37.90%	0.61	7.63E-73
C1QTNF1	SMC	58.20%	25.50%	0.55	9.76E-73
APOLD1	SMC	39.30%	6.10%	0.56	7.67E-72
FRMD3	SMC	26.90%	2.00%	0.30	8.39E-72
SYTL2	SMC	44.40%	17.30%	0.51	1.34E-71
ARHGAP29	SMC	56.40%	14.50%	0.61	1.37E-70
NID1	SMC	38.70%	6.50%	0.46	1.62E-70
DUSP14	SMC	64.80%	48.70%	0.56	4.87E-70
IGFBP2	SMC	24.60%	1.40%	0.33	1.36E-69
NPNT	SMC	22.60%	0.90%	0.28	2.09E-69
PRKG1	SMC	27.20%	2.50%	0.37	3.45E-69
HLA-B	SMC	98.90%	98.10%	0.57	3.87E-69
TBX2	SMC	19.20%	0.50%	0.27	6.52E-69
LDHB	SMC	77.40%	57.20%	0.54	7.10E-68
PCSK7	SMC	48.70%	22.40%	0.53	1.65E-66
MYL12A	SMC	93.70%	90.90%	0.54	1.73E-65
SORBS2	SMC	32.40%	35.20%	0.45	1.76E-65
DLX5	SMC	29.20%	4.00%	0.45	2.93E-65

IFITM3	SMC	98.30%	92.90%	0.53	3.88E-65
RCAN2	SMC	23.80%	1.50%	0.37	4.44E-65
CAV1	SMC	96.60%	80.20%	0.63	3.70E-64
SMTN	SMC	46.40%	23.00%	0.44	9.43E-64
EHD2	SMC	57.00%	31.20%	0.50	3.23E-63
NCL	SMC	90.50%	88.30%	0.55	1.20E-62
EIF4A1	SMC	98.00%	97.60%	0.61	1.49E-61
NEURL1B	SMC	23.20%	1.40%	0.29	4.59E-61
CCL2	SMC	82.80%	70.00%	1.23	3.26E-60
ANO1	SMC	43.30%	11.20%	0.46	4.77E-60
CRIM1	SMC	45.60%	18.20%	0.52	1.49E-59
NGF	SMC	31.80%	7.90%	0.53	2.06E-59
HOPX	SMC	21.80%	1.10%	0.33	7.38E-59
ADAMTS1	SMC	71.90%	42.10%	0.81	9.92E-59
SPARC	SMC	93.40%	82.90%	0.78	1.49E-58
SEZ6L2	SMC	21.20%	1.40%	0.26	1.94E-58
DLC1	SMC	63.30%	42.30%	0.51	6.40E-58
ADAMTS9	SMC	33.50%	4.80%	0.49	1.23E-57
OLFML2B	SMC	59.30%	28.30%	0.61	1.63E-57
JAG1	SMC	41.50%	12.20%	0.49	1.75E-57
PFKFB3	SMC	52.40%	30.00%	0.52	1.76E-56
CAV2	SMC	62.50%	38.40%	0.52	4.92E-56
MFGE8	SMC	89.70%	66.70%	0.63	2.68E-55
KCNMB1	SMC	27.20%	3.60%	0.40	3.25E-54
PDGFRB	SMC	67.90%	38.20%	0.61	3.73E-54
CHSY1	SMC	45.30%	23.60%	0.43	4.54E-54
RPS7	SMC	98.30%	99.00%	0.38	5.49E-54
KLF9	SMC	65.90%	47.00%	0.50	7.97E-54
CTD-3193K9.4	SMC	20.90%	1.90%	0.30	8.90E-54
MSRB3	SMC	46.10%	24.10%	0.45	1.51E-53
MAP3K7CL	SMC	20.60%	3.80%	0.35	1.64E-53
MIR143HG	SMC	14.00%	0.30%	0.32	2.21E-53
RPL3	SMC	99.10%	99.70%	0.39	1.09E-52
MTHFD2	SMC	70.20%	47.50%	0.55	1.21E-52
ROCK1	SMC	73.40%	54.30%	0.47	1.93E-52
MAT2A	SMC	71.10%	56.40%	0.61	5.31E-52
HNRNPA1	SMC	95.70%	96.70%	0.41	1.65E-51
CNN3	SMC	87.70%	67.40%	0.57	1.65E-51
ANXA6	SMC	68.80%	47.30%	0.47	2.07E-51
ITIH5	SMC	40.40%	14.80%	0.50	2.19E-51
FMO3	SMC	19.50%	1.20%	0.33	3.30E-51

ADRA2A	SMC	16.90%	1.40%	0.30	4.91E-51
IFI16	SMC	82.20%	69.40%	0.60	5.55E-51
RPS8	SMC	99.10%	99.70%	0.40	9.44E-51
RPS19	SMC	98.90%	99.60%	0.38	1.18E-50
FABP5	SMC	58.50%	20.80%	0.68	2.40E-50
SIK1	SMC	41.30%	16.50%	0.48	7.48E-50
CLMN	SMC	25.20%	4.50%	0.34	2.07E-48
IFITM2	SMC	96.60%	87.60%	0.52	2.54E-48
C11orf96	SMC	71.30%	40.10%	0.61	5.41E-48
NPM1	SMC	97.40%	96.40%	0.49	6.81E-48
SNCG	SMC	25.50%	12.70%	0.57	1.42E-47
RASL12	SMC	22.10%	4.30%	0.33	2.90E-47
RERGL	SMC	11.50%	0.20%	0.58	3.44E-47
CALM2	SMC	98.60%	97.90%	0.45	3.74E-47
SARS	SMC	65.60%	52.70%	0.39	4.14E-47
NOP16	SMC	58.70%	36.70%	0.50	1.05E-46
RPL5	SMC	98.60%	99.30%	0.37	1.27E-46
HLA-A	SMC	98.90%	98.20%	0.43	1.50E-46
PLEKHA4	SMC	61.30%	38.60%	0.46	2.40E-46
SLC25A6	SMC	95.70%	94.90%	0.41	5.91E-46
BST2	SMC	65.30%	27.30%	0.57	8.28E-46
GPRC5C	SMC	28.70%	9.60%	0.36	1.34E-45
NAP1L1	SMC	91.70%	87.80%	0.42	3.46E-45
CD59	SMC	90.50%	82.00%	0.51	4.65E-45
SLIT3	SMC	40.40%	13.20%	0.46	1.48E-44
ARHGEF25	SMC	19.50%	2.70%	0.26	2.67E-44
STEAP4	SMC	60.70%	26.80%	0.72	3.14E-44
SERBP1	SMC	88.00%	83.50%	0.41	6.57E-44
C12orf44	SMC	61.30%	46.10%	0.38	1.26E-43
RPS16	SMC	99.10%	99.40%	0.34	2.08E-43
COBLL1	SMC	43.60%	19.20%	0.40	2.87E-43
DMD	SMC	19.80%	2.20%	0.27	1.49E-42
SLC7A2	SMC	51.90%	20.00%	0.33	2.41E-42
PABPC1	SMC	94.30%	92.90%	0.39	4.48E-42
NOP56	SMC	66.80%	54.50%	0.41	4.50E-42
MALAT1	SMC	100.00%	100.00%	0.32	9.70E-42
OLFML2A	SMC	27.80%	5.20%	0.37	1.40E-41
CTSC	SMC	43.00%	13.40%	0.58	1.57E-41
MPRIP	SMC	39.00%	15.00%	0.36	1.84E-41
ADAMTS12	SMC	21.80%	2.50%	0.25	2.72E-41
PTRF	SMC	93.40%	79.10%	0.53	3.76E-41

SOD3	SMC	44.10%	25.10%	0.70	3.99E-41
PDGFA	SMC	48.70%	23.20%	0.48	6.25E-41
A1BG	SMC	34.10%	7.30%	0.33	1.08E-40
PAG1	SMC	28.10%	4.70%	0.34	3.58E-40
PRKAR1A	SMC	71.60%	62.40%	0.37	5.02E-40
TBC1D1	SMC	37.50%	13.30%	0.37	5.67E-40
KANK2	SMC	54.20%	34.50%	0.41	8.77E-40
MRPL32	SMC	67.30%	56.30%	0.41	1.21E-39
FHL2	SMC	55.60%	33.00%	0.54	1.24E-39
RSL1D1	SMC	76.50%	68.60%	0.40	4.01E-39
EIF5B	SMC	77.10%	69.60%	0.40	5.53E-39
ASAP1	SMC	48.70%	23.30%	0.43	7.80E-39
THBS1	SMC	46.40%	16.90%	0.57	1.22E-38
TLN1	SMC	65.60%	50.10%	0.39	1.25E-38
LRRC32	SMC	37.50%	9.80%	0.37	2.06E-38
NGFR	SMC	22.30%	3.80%	0.37	2.55E-38
LURAP1L	SMC	21.80%	3.90%	0.26	3.49E-38
GPM6B	SMC	31.50%	7.60%	0.37	6.68E-38
SLC2A3	SMC	61.60%	34.00%	0.52	6.73E-38
HLA-C	SMC	98.60%	97.40%	0.44	8.32E-38
TNS1	SMC	59.90%	39.70%	0.45	1.14E-37
TUBA1B	SMC	96.80%	96.20%	0.46	1.53E-37
MEF2C	SMC	53.90%	21.00%	0.44	1.57E-37
PDLIM3	SMC	67.30%	54.30%	0.50	2.56E-37
TOP1	SMC	65.30%	53.60%	0.41	8.07E-37
ATF4	SMC	95.40%	92.30%	0.42	1.02E-36
HIPK2	SMC	38.10%	17.50%	0.35	1.10E-36
RPL23A	SMC	99.10%	99.80%	0.28	1.21E-36
HEXIM1	SMC	79.40%	71.40%	0.47	2.97E-36
SLC1A5	SMC	47.30%	36.30%	0.36	7.26E-36
NES	SMC	23.50%	3.40%	0.30	8.29E-36
SLN	SMC	30.70%	11.20%	0.87	1.00E-34
EGFLAM	SMC	33.00%	8.90%	0.33	1.20E-34
ARID5B	SMC	91.10%	77.30%	0.52	1.54E-34
RPL35	SMC	98.60%	99.50%	0.27	1.83E-34
U2AF1	SMC	78.20%	77.10%	0.34	2.37E-34
PLK2	SMC	48.70%	26.30%	0.54	3.74E-34
FAU	SMC	98.90%	99.60%	0.26	3.91E-34
HSPD1	SMC	78.20%	71.90%	0.40	9.86E-34
VMP1	SMC	71.60%	59.10%	0.43	1.20E-33
CHD1	SMC	67.00%	57.00%	0.42	1.74E-33

HNRNPF	SMC	74.20%	66.40%	0.38	3.78E-33
RASGRP2	SMC	21.20%	3.40%	0.26	6.01E-33
RPL10A	SMC	99.40%	99.40%	0.32	6.52E-33
ZNF703	SMC	47.00%	26.80%	0.40	9.82E-33
RAN	SMC	86.00%	86.70%	0.34	1.10E-32
PA2G4	SMC	74.50%	67.60%	0.34	1.18E-32
PFDN2	SMC	71.60%	67.30%	0.31	3.91E-32
B2M	SMC	100.00%	100.00%	0.30	5.19E-32
CCT2	SMC	64.80%	52.30%	0.35	5.44E-32
RPL18	SMC	98.60%	99.30%	0.29	8.50E-32
SNHG15	SMC	53.00%	36.70%	0.37	1.05E-31
HNRNPK	SMC	91.10%	89.90%	0.34	2.86E-31
PVRL2	SMC	54.70%	30.40%	0.39	3.15E-31
RASAL2	SMC	28.10%	11.60%	0.30	3.25E-31
RPL37A	SMC	100.00%	99.90%	0.27	2.29E-30
PRMT1	SMC	65.60%	52.50%	0.34	2.64E-30
ANGPT1	SMC	24.60%	5.40%	0.31	3.57E-30
UQCRH	SMC	86.20%	84.00%	0.31	1.39E-29
WDR43	SMC	53.00%	36.20%	0.38	2.47E-29
ITGA8	SMC	11.70%	1.30%	0.27	3.03E-29
ITGA3	SMC	18.60%	3.20%	0.25	3.35E-29
SYNE2	SMC	43.30%	16.30%	0.43	4.56E-29
TUSC3	SMC	28.10%	6.70%	0.26	4.88E-29
CEBPZ	SMC	50.40%	38.80%	0.33	5.53E-29
CDC42EP4	SMC	41.80%	26.00%	0.32	8.03E-29
ARHGAP6	SMC	36.10%	12.00%	0.34	8.33E-29
NCOA7	SMC	52.40%	40.60%	0.41	1.20E-28
NASP	SMC	67.00%	55.50%	0.37	1.21E-28
ATP5B	SMC	87.40%	86.70%	0.30	1.63E-28
NACA	SMC	98.60%	99.20%	0.28	2.21E-28
NDUFS2	SMC	55.00%	39.40%	0.32	4.46E-28
HGF	SMC	19.80%	6.10%	0.29	5.03E-28
UBE2N	SMC	68.80%	56.00%	0.33	1.17E-27
LGALS1	SMC	99.10%	97.30%	0.28	1.31E-27
ADCY3	SMC	28.40%	7.50%	0.26	1.42E-27
CYGB	SMC	26.90%	7.10%	0.27	2.12E-27
MYL6	SMC	100.00%	99.10%	0.35	2.17E-27
CDC42EP1	SMC	45.00%	22.80%	0.33	3.06E-27
RSU1	SMC	55.90%	39.80%	0.33	3.92E-27
FAM46A	SMC	71.30%	63.40%	0.50	3.98E-27
CCT3	SMC	69.60%	59.10%	0.30	5.75E-27

RPS5	SMC	98.90%	99.10%	0.29	8.17E-27
SEMA5A	SMC	37.80%	12.70%	0.34	8.42E-27
ATP5G2	SMC	90.80%	88.80%	0.31	9.10E-27
TEX41	SMC	29.20%	12.90%	0.29	9.41E-27
F2R	SMC	23.20%	4.50%	0.26	1.05E-26
TMEM47	SMC	40.10%	25.10%	0.32	1.10E-26
CSNK1A1	SMC	79.10%	74.30%	0.32	1.34E-26
SPRY2	SMC	34.10%	15.40%	0.31	2.63E-26
EIF3J	SMC	67.60%	54.70%	0.36	3.39E-26
CSNK1D	SMC	53.00%	35.50%	0.34	4.90E-26
SLC3A2	SMC	77.10%	75.80%	0.40	6.86E-26
NR3C1	SMC	59.60%	51.70%	0.31	8.90E-26
RPL6	SMC	98.60%	99.70%	0.25	8.98E-26
PLAU	SMC	58.50%	34.20%	0.58	1.07E-25
NXT1	SMC	53.00%	40.20%	0.32	2.98E-25
ROCK2	SMC	44.40%	25.20%	0.33	3.10E-25
FAM13C	SMC	36.70%	17.20%	0.36	3.51E-25
NT5DC2	SMC	26.60%	12.20%	0.25	3.68E-25
TTLL7	SMC	22.10%	10.40%	0.26	4.01E-25
SERPINI1	SMC	26.10%	13.00%	0.29	4.59E-25
IMPDH2	SMC	68.50%	54.90%	0.33	4.74E-25
HNRNPA3	SMC	81.40%	76.30%	0.31	5.67E-25
FUS	SMC	92.60%	87.30%	0.37	6.33E-25
BRD2	SMC	86.00%	82.80%	0.37	7.64E-25
SH3BGRL	SMC	87.40%	78.90%	0.35	8.78E-25
IFIT3	SMC	28.90%	11.40%	0.40	9.52E-25
COL12A1	SMC	44.10%	22.60%	0.39	1.12E-24
FOSL2	SMC	52.40%	39.50%	0.35	2.87E-24
KIAA0040	SMC	51.60%	26.80%	0.41	3.20E-24
ILK	SMC	64.80%	57.50%	0.29	3.25E-24
EIF1AX	SMC	79.70%	76.90%	0.30	3.39E-24
SORBS3	SMC	39.50%	29.60%	0.26	6.81E-24
CYCS	SMC	86.50%	81.60%	0.37	7.60E-24
HSPB6	SMC	37.20%	18.30%	0.36	1.41E-23
RPL9	SMC	99.10%	99.40%	0.26	1.83E-23
NOLC1	SMC	51.30%	35.70%	0.34	2.43E-23
BAZ1A	SMC	69.60%	56.60%	0.39	2.45E-23
ETS1	SMC	41.80%	19.30%	0.36	2.72E-23
GNL3	SMC	60.50%	52.30%	0.31	3.51E-23
BTF3	SMC	97.40%	97.80%	0.26	4.46E-23
PDE1A	SMC	40.10%	18.40%	0.31	6.02E-23

SAFB2	SMC	53.30%	42.10%	0.30	1.63E-22
CCT5	SMC	62.80%	56.90%	0.28	1.65E-22
RPL18A	SMC	99.40%	99.70%	0.25	1.75E-22
RPL7A	SMC	99.10%	99.30%	0.27	1.85E-22
LAMC1	SMC	47.90%	34.60%	0.30	2.00E-22
EIF3A	SMC	72.50%	63.90%	0.31	2.59E-22
BCAR1	SMC	31.80%	14.20%	0.27	4.56E-22
RPL29	SMC	98.60%	99.30%	0.26	6.04E-22
LBH	SMC	53.60%	56.20%	0.34	1.12E-21
ATP1A1	SMC	77.90%	71.10%	0.31	1.59E-21
EEF2	SMC	99.10%	97.70%	0.28	1.69E-21
LIF	SMC	35.50%	13.40%	0.52	1.87E-21
TOB1	SMC	61.30%	52.50%	0.34	3.88E-21
UGCG	SMC	39.00%	22.10%	0.30	3.91E-21
SASH1	SMC	40.10%	23.10%	0.31	3.97E-21
HNRNPH3	SMC	71.60%	64.80%	0.29	5.15E-21
ZBTB38	SMC	53.30%	40.40%	0.32	5.82E-21
KIF5B	SMC	81.10%	70.80%	0.30	6.62E-21
SEPHS2	SMC	50.70%	36.90%	0.31	7.27E-21
LOXL2	SMC	40.40%	18.60%	0.38	8.21E-21
SUMO2	SMC	94.60%	92.40%	0.26	1.13E-20
RPS3A	SMC	98.90%	99.60%	0.26	1.41E-20
LITAF	SMC	70.20%	60.90%	0.33	1.71E-20
HNRNPDL	SMC	86.80%	84.50%	0.29	1.95E-20
SF1	SMC	63.90%	51.90%	0.30	2.18E-20
SRSF2	SMC	87.70%	83.70%	0.32	2.59E-20
RBM39	SMC	88.00%	85.30%	0.28	3.08E-20
CAMK2N1	SMC	26.40%	11.30%	0.26	4.78E-20
FKBP4	SMC	42.40%	26.70%	0.29	5.17E-20
TAX1BP1	SMC	72.80%	65.70%	0.29	5.29E-20
STAT3	SMC	77.70%	68.90%	0.33	6.10E-20
HLA-F	SMC	45.30%	24.70%	0.35	6.35E-20
NFE2L2	SMC	79.10%	78.90%	0.33	6.82E-20
EIF4G2	SMC	92.80%	88.30%	0.29	7.25E-20
NR4A3	SMC	42.40%	24.50%	0.37	1.09E-19
ZYX	SMC	34.10%	27.00%	0.26	3.23E-19
EIF4A3	SMC	77.70%	67.80%	0.38	5.37E-19
ECE1	SMC	45.60%	25.30%	0.32	5.60E-19
HIF1A	SMC	66.80%	63.60%	0.29	6.59E-19
RPS18	SMC	99.70%	99.70%	0.29	7.93E-19
SLC7A5	SMC	39.30%	22.70%	0.32	8.81E-19

AKAP12	SMC	43.80%	20.80%	0.42	8.83E-19
RPL36A	SMC	95.40%	94.60%	0.29	9.68E-19
HSPB1	SMC	97.70%	94.40%	0.39	1.31E-18
HNRNPM	SMC	78.80%	69.70%	0.29	1.32E-18
B3GNT2	SMC	29.50%	12.10%	0.26	2.91E-18
RPS3	SMC	99.10%	99.50%	0.26	2.97E-18
PTPN1	SMC	41.80%	34.20%	0.28	3.40E-18
RAB13	SMC	72.20%	63.10%	0.31	5.44E-18
AGT	SMC	18.60%	4.00%	0.42	1.35E-17
EPHX1	SMC	74.80%	57.20%	0.35	1.58E-17
PRPF38B	SMC	67.60%	56.10%	0.30	1.70E-17
DDX18	SMC	60.50%	51.80%	0.27	1.73E-17
C1orf54	SMC	50.70%	29.30%	0.32	1.79E-17
PPP2CA	SMC	58.50%	46.50%	0.28	2.69E-17
NR4A1	SMC	79.10%	74.40%	0.39	3.85E-17
ATF3	SMC	75.60%	69.00%	0.43	6.88E-17
SON	SMC	93.40%	89.30%	0.28	8.27E-17
SNAI2	SMC	45.30%	25.30%	0.40	9.01E-17
NDUFAF4	SMC	39.50%	26.60%	0.26	9.02E-17
ARHGAP1	SMC	39.80%	23.00%	0.26	9.89E-17
FBXO32	SMC	26.60%	20.40%	0.25	1.02E-16
MT-CO3	SMC	99.70%	99.50%	0.26	1.14E-16
BBX	SMC	59.90%	51.90%	0.27	1.31E-16
PPP2R2A	SMC	50.10%	36.70%	0.27	1.51E-16
MGLL	SMC	51.90%	29.40%	0.36	1.70E-16
CHCHD10	SMC	23.20%	8.90%	0.34	1.90E-16
HNRNPU	SMC	73.60%	67.40%	0.27	2.72E-16
SERPINH1	SMC	73.60%	59.80%	0.35	3.63E-16
RPL4	SMC	98.00%	97.80%	0.26	3.85E-16
ANKRD10	SMC	55.60%	41.00%	0.28	4.12E-16
TARS	SMC	46.10%	35.50%	0.25	6.09E-16
DSTN	SMC	98.30%	96.50%	0.39	6.19E-16
FAT1	SMC	37.50%	23.40%	0.25	9.23E-16
TOB2	SMC	56.70%	50.00%	0.26	1.06E-15
PRKCDBP	SMC	86.20%	77.60%	0.34	1.08E-15
SRSF3	SMC	78.50%	79.10%	0.28	1.92E-15
DYNLL1	SMC	96.00%	96.20%	0.25	2.74E-15
COL6A1	SMC	92.80%	81.30%	0.44	4.42E-15
ARID4B	SMC	67.30%	61.90%	0.26	1.13E-14
C12orf57	SMC	91.40%	83.10%	0.28	1.29E-14
MAFF	SMC	40.40%	27.20%	0.30	1.55E-14

DDX3X	SMC	77.90%	69.80%	0.32	1.73E-14
NAA15	SMC	41.80%	27.70%	0.26	1.79E-14
POR	SMC	45.00%	27.40%	0.25	4.00E-14
KPNB1	SMC	60.50%	51.50%	0.25	4.25E-14
LAMA4	SMC	57.60%	42.20%	0.30	4.90E-14
RBM25	SMC	74.50%	70.20%	0.26	6.02E-14
DDX5	SMC	98.30%	98.10%	0.30	6.42E-14
TACC1	SMC	72.50%	67.20%	0.27	6.50E-14
LGALS3BP	SMC	73.90%	52.10%	0.31	7.54E-14
SET	SMC	69.30%	58.20%	0.31	1.60E-13
CREM	SMC	52.10%	45.40%	0.40	1.96E-13
PDK4	SMC	43.60%	27.20%	0.51	2.04E-13
VASP	SMC	51.30%	33.40%	0.30	2.99E-13
ZFAND5	SMC	58.20%	51.20%	0.26	4.59E-13
7SK.2	SMC	13.80%	20.70%	0.36	6.83E-13
PRRX1	SMC	76.50%	66.00%	0.32	7.96E-13
MYC	SMC	78.50%	65.10%	0.48	8.71E-13
SFPQ	SMC	70.20%	64.50%	0.26	9.76E-13
SPTBN1	SMC	57.60%	47.50%	0.27	2.11E-12
DDIT4	SMC	65.00%	61.20%	0.47	2.71E-12
KPNA2	SMC	63.30%	52.30%	0.32	7.60E-12
TXNRD1	SMC	42.40%	32.30%	0.30	1.17E-11
EIF4B	SMC	73.60%	65.20%	0.26	1.67E-11
CDKN1A	SMC	88.30%	83.10%	0.34	1.97E-11
MT1G	SMC	27.80%	20.50%	0.57	6.46E-11
ID3	SMC	84.00%	73.90%	0.44	9.81E-11
TUBA1C	SMC	72.50%	67.50%	0.26	1.02E-10
IFITM1	SMC	45.60%	29.60%	0.28	1.04E-10
MT2A	SMC	99.40%	95.10%	0.31	1.06E-10
MCL1	SMC	78.50%	71.40%	0.27	1.23E-10
GEM	SMC	85.40%	75.20%	0.36	3.06E-10
RASD1	SMC	55.90%	37.30%	0.39	3.21E-10
NTRK2	SMC	35.00%	31.40%	0.30	3.96E-10
CLEC11A	SMC	31.50%	19.20%	0.25	6.40E-10
SLC38A2	SMC	84.20%	77.40%	0.27	7.91E-10
IFRD1	SMC	68.80%	59.50%	0.26	1.52E-09
HMGN2	SMC	79.10%	73.40%	0.26	1.66E-09
ETS2	SMC	49.60%	31.60%	0.26	2.00E-09
ENAH	SMC	53.00%	40.80%	0.26	9.28E-09
CKB	SMC	42.10%	25.40%	0.30	1.76E-08
TSC22D1	SMC	65.30%	53.30%	0.40	6.62E-08

SMOC2	SMC	54.70%	35.60%	0.26	7.79E-08
HES1	SMC	45.00%	48.90%	0.29	1.02E-07
FILIP1	SMC	34.10%	18.90%	0.25	1.65E-07
ISG15	SMC	43.00%	30.20%	0.30	2.09E-07
RGS16	SMC	65.90%	57.40%	0.37	2.48E-06
APOE	SMC	28.40%	14.10%	0.27	9.14E-06
C8orf4	SMC	23.50%	11.90%	0.34	2.03E-04
IL6	SMC	41.80%	36.00%	0.60	1.99E-03
CFH	SMC	66.80%	64.70%	0.32	8.51E-02
IGFBP5	SMC	51.00%	51.30%	0.32	9.71E-01
MTRNR2L1	SMC	26.90%	20.10%	0.39	1.00E+00
IL32	T cell	90.30%	11.40%	2.38	1.15E-264
CXCR4	T cell	97.20%	10.60%	3.21	2.73E-260
CREM	T cell	86.10%	45.00%	2.13	1.43E-245
LEPROTL1	T cell	76.40%	26.50%	1.38	1.39E-244
CD2	T cell	76.40%	0.80%	1.81	4.37E-200
CD52	T cell	88.90%	4.10%	2.44	3.14E-198
CD3D	T cell	76.40%	0.70%	1.63	1.55E-195
TMEM66	T cell	93.80%	85.30%	1.27	2.73E-188
CD3E	T cell	70.80%	0.30%	1.41	4.59E-187
PTPRC	T cell	90.30%	9.80%	1.98	8.93E-184
SPOCK2	T cell	70.80%	1.00%	1.37	4.31E-182
IL7R	T cell	81.20%	2.30%	1.99	2.51E-177
PTPRCAP	T cell	69.40%	0.80%	1.41	3.73E-167
CCL5	T cell	63.90%	2.00%	2.32	1.06E-163
B2M	T cell	100.00%	100.00%	1.14	1.73E-158
RPS29	T cell	100.00%	99.40%	1.11	9.29E-155
IL2RG	T cell	67.40%	1.90%	1.24	4.38E-142
ICAM3	T cell	59.70%	16.80%	0.99	4.96E-141
TUBA4A	T cell	54.20%	17.40%	1.12	1.05E-139
CD3G	T cell	52.10%	0.30%	1.10	1.16E-131
ARHGDIB	T cell	94.40%	20.60%	1.74	6.97E-128
CD7	T cell	50.00%	1.80%	1.27	9.93E-127
LTB	T cell	52.80%	1.00%	1.64	1.60E-126
ACAP1	T cell	59.00%	6.50%	0.99	2.39E-126
RPS27	T cell	99.30%	100.00%	0.86	1.08E-121
SRGN	T cell	98.60%	43.30%	1.84	5.31E-116
HLA-B	T cell	100.00%	98.10%	1.30	2.60E-115
PPP2R5C	T cell	67.40%	29.90%	0.97	2.15E-114
SYTL3	T cell	54.20%	7.20%	1.03	3.50E-114
GZMA	T cell	46.50%	0.40%	1.39	1.21E-111

KLRB1	T cell	43.10%	0.30%	1.93	3.35E-111
LCK	T cell	47.90%	0.50%	0.85	1.41E-107
BTG1	T cell	90.30%	68.70%	1.45	4.83E-107
SAMSN1	T cell	75.00%	5.20%	1.43	3.46E-99
CORO1A	T cell	72.90%	8.80%	1.41	1.21E-98
RPS19	T cell	100.00%	99.50%	0.86	1.64E-98
RUNX3	T cell	54.20%	3.30%	1.14	7.51E-97
CDC42SE2	T cell	63.90%	14.10%	0.91	1.68E-92
HLA-A	T cell	100.00%	98.20%	1.02	2.01E-91
RHOH	T cell	54.90%	2.00%	1.12	8.85E-91
CTSW	T cell	45.10%	1.10%	1.13	6.67E-88
STK4	T cell	75.00%	16.40%	1.14	1.86E-87
HCST	T cell	79.90%	10.00%	1.34	3.69E-84
CD48	T cell	53.50%	2.60%	1.11	7.81E-84
EZR	T cell	84.00%	59.70%	1.07	1.10E-83
EML4	T cell	56.90%	15.90%	0.84	6.37E-82
CYTIP	T cell	68.10%	6.00%	1.32	9.64E-82
NKG7	T cell	36.10%	0.40%	1.47	2.86E-81
GZMM	T cell	32.60%	0.30%	0.73	1.49E-80
STAT4	T cell	41.00%	0.70%	0.68	2.83E-80
SLA	T cell	55.60%	3.60%	0.99	6.98E-79
RPL28	T cell	100.00%	99.70%	0.77	1.14E-78
PBXIP1	T cell	61.80%	32.30%	0.79	7.00E-78
RNF19A	T cell	52.80%	19.70%	0.82	1.79E-77
RPS21	T cell	99.30%	98.60%	0.78	5.12E-76
RPLP2	T cell	100.00%	99.90%	0.65	7.42E-76
PDCD4	T cell	66.70%	33.00%	0.84	7.68E-75
HLA-C	T cell	99.30%	97.40%	0.93	1.12E-73
FAM129A	T cell	58.30%	27.50%	0.84	5.07E-72
CD6	T cell	31.90%	0.30%	0.69	5.58E-72
CD53	T cell	68.80%	10.10%	1.20	8.33E-72
TRAT1	T cell	30.60%	0.10%	0.67	8.49E-72
CST7	T cell	50.00%	2.20%	1.10	1.74E-71
FYN	T cell	62.50%	33.80%	0.78	3.89E-71
GZMK	T cell	31.20%	0.40%	1.16	8.13E-70
STK17B	T cell	64.60%	16.70%	1.07	8.52E-70
RPS15A	T cell	99.30%	99.60%	0.67	6.31E-69
RP11-347P5.1	T cell	43.80%	2.90%	0.90	1.11E-67
SLC38A1	T cell	41.00%	9.40%	0.72	2.16E-67
S1PR4	T cell	31.20%	0.40%	0.66	2.45E-65
TNFRSF18	T cell	31.90%	4.60%	0.98	3.13E-65

BCL11B	T cell	28.50%	0.20%	0.55	1.24E-64
CD96	T cell	29.20%	0.30%	0.61	1.63E-64
CXCR3	T cell	37.50%	2.50%	0.93	8.39E-63
PTPN7	T cell	35.40%	1.30%	0.77	1.04E-62
CD247	T cell	28.50%	0.20%	0.56	3.73E-62
RPS3	T cell	100.00%	99.40%	0.70	1.17E-61
CD37	T cell	68.10%	10.00%	1.10	1.57E-61
ANKRD12	T cell	84.70%	70.50%	0.78	1.38E-60
SC5D	T cell	38.90%	11.30%	0.65	2.27E-60
ISG20	T cell	56.90%	18.50%	0.92	4.03E-60
RPLP1	T cell	100.00%	99.80%	0.64	7.98E-60
STK17A	T cell	50.70%	22.70%	0.72	3.16E-59
RPS27A	T cell	100.00%	99.80%	0.60	5.78E-59
G3BP2	T cell	54.90%	32.10%	0.67	1.02E-57
CAMK4	T cell	25.70%	0.20%	0.50	1.23E-57
HMGB2	T cell	62.50%	47.00%	0.81	1.80E-56
ITGA4	T cell	34.00%	2.40%	0.67	4.03E-56
ICOS	T cell	25.00%	0.20%	0.53	6.19E-56
SYAP1	T cell	55.60%	40.80%	0.67	1.30E-55
TGFB1	T cell	65.30%	43.30%	0.71	1.40E-55
LAPTM5	T cell	84.00%	21.50%	0.84	2.49E-55
CNOT6L	T cell	36.80%	10.40%	0.68	2.68E-55
RPSAP58	T cell	77.80%	62.40%	0.65	2.96E-55
P2RY8	T cell	27.10%	0.60%	0.61	7.05E-55
SYNE2	T cell	62.50%	16.50%	1.00	7.47E-55
RAC2	T cell	47.90%	4.50%	0.95	8.14E-55
PBX4	T cell	27.10%	1.20%	0.53	1.01E-54
FNBP1	T cell	58.30%	22.90%	0.81	1.06E-53
RPL27A	T cell	100.00%	99.60%	0.57	1.53E-53
PDCL3	T cell	52.10%	24.90%	0.66	6.80E-53
TC2N	T cell	25.00%	0.50%	0.53	1.29E-52
GPR65	T cell	41.70%	2.20%	0.81	2.20E-52
PRPF38B	T cell	73.60%	56.30%	0.69	3.13E-52
AIM1	T cell	31.90%	4.90%	0.66	1.24E-51
GIMAP7	T cell	54.90%	5.70%	0.88	4.81E-51
RPL38	T cell	99.30%	99.30%	0.60	5.08E-51
RPL23A	T cell	100.00%	99.70%	0.57	6.81E-51
RPS10	T cell	98.60%	96.00%	0.68	7.50E-51
AC092580.4	T cell	22.90%	0.20%	0.59	2.05E-50
NR3C1	T cell	62.50%	51.80%	0.64	4.71E-50
TNFRSF1B	T cell	54.90%	9.60%	0.93	6.83E-50

CD69	T cell	45.10%	3.30%	1.18	1.13E-49
GATA3	T cell	25.00%	0.30%	0.50	5.95E-49
CD8A	T cell	20.10%	0.10%	0.66	6.62E-49
RPS28	T cell	100.00%	99.80%	0.59	7.66E-49
PPP1R2	T cell	51.40%	39.10%	0.59	1.01E-48
CCR6	T cell	22.20%	0.40%	0.58	1.29E-48
RASGRP1	T cell	22.20%	0.50%	0.38	2.08E-48
ISCA1	T cell	55.60%	37.80%	0.54	1.09E-47
RPL30	T cell	100.00%	99.60%	0.55	1.45E-47
YPEL5	T cell	78.50%	60.10%	0.67	1.82E-47
FYB	T cell	52.10%	7.30%	0.89	2.71E-47
PDCD1	T cell	20.80%	0.10%	0.51	4.30E-47
CCND3	T cell	42.40%	23.20%	0.58	7.43E-47
P2RY10	T cell	24.30%	0.30%	0.46	8.39E-47
TSC22D3	T cell	94.40%	83.10%	0.93	9.71E-47
RPL31	T cell	100.00%	99.60%	0.58	1.79E-46
RPL21	T cell	100.00%	99.90%	0.52	4.86E-46
MPZL3	T cell	21.50%	1.10%	0.47	6.94E-46
VPS37B	T cell	41.00%	13.90%	0.66	9.03E-46
APOBEC3G	T cell	26.40%	7.30%	0.53	1.26E-45
PIK3IP1	T cell	45.80%	13.80%	0.68	1.49E-45
HN1	T cell	52.80%	35.40%	0.57	5.64E-45
GPR171	T cell	22.20%	0.20%	0.48	2.22E-44
KMT2E	T cell	74.30%	59.00%	0.69	1.30E-43
GPR183	T cell	72.90%	19.60%	1.26	3.05E-43
RPL36A	T cell	97.20%	94.60%	0.69	3.91E-43
RPS15	T cell	99.30%	99.90%	0.46	4.59E-43
SH2D2A	T cell	22.90%	1.40%	0.41	4.65E-43
RPSA	T cell	98.60%	97.70%	0.65	5.54E-43
RPL36	T cell	99.30%	99.20%	0.54	9.64E-43
RPS16	T cell	100.00%	99.40%	0.52	9.83E-43
FAM177A1	T cell	60.40%	59.60%	0.52	2.07E-42
MCL1	T cell	70.10%	71.70%	0.74	2.14E-42
CLEC2D	T cell	25.00%	1.30%	0.54	1.19E-41
RNF125	T cell	27.80%	2.00%	0.59	2.28E-41
LAT	T cell	31.90%	3.10%	0.64	2.34E-41
CCR7	T cell	37.50%	2.70%	0.89	2.86E-41
PTGER4	T cell	46.50%	22.10%	0.70	3.59E-41
43349	T cell	50.70%	26.40%	0.60	5.54E-41
SF1	T cell	67.40%	52.10%	0.58	7.87E-41
SUB1	T cell	90.30%	82.20%	0.60	9.79E-41

ETS1	T cell	50.00%	19.70%	0.69	1.19E-40
MBP	T cell	54.20%	16.50%	0.74	2.03E-40
RPS25	T cell	100.00%	99.80%	0.51	2.81E-40
GNG2	T cell	37.50%	5.70%	0.68	1.02E-39
ZAP70	T cell	16.70%	0.10%	0.35	1.17E-39
PLAC8	T cell	22.20%	0.30%	0.40	1.34E-39
BIN2	T cell	31.90%	3.10%	0.63	4.76E-39
RP11-640M9.1	T cell	31.20%	10.90%	0.53	5.21E-39
C9orf78	T cell	58.30%	43.80%	0.53	6.00E-39
PRF1	T cell	18.10%	0.20%	0.49	3.25E-38
HOPX	T cell	25.00%	1.50%	0.65	3.74E-38
RPL41	T cell	100.00%	100.00%	0.47	4.48E-38
WHSC1L1	T cell	50.00%	37.20%	0.52	5.44E-38
WIPF1	T cell	44.40%	32.30%	0.51	6.46E-38
ZFP36L2	T cell	75.70%	57.60%	0.81	7.16E-38
KIAA1551	T cell	42.40%	10.00%	0.64	8.17E-38
SMCHD1	T cell	40.30%	15.60%	0.63	9.32E-38
RPS2	T cell	100.00%	99.60%	0.56	1.52E-37
EVL	T cell	36.10%	16.70%	0.51	9.91E-37
SLBP	T cell	30.60%	15.90%	0.49	1.00E-36
MECP2	T cell	41.70%	26.00%	0.53	1.08E-36
AKNA	T cell	31.20%	7.60%	0.54	1.58E-36
ATP1B3	T cell	79.20%	70.30%	0.64	2.47E-36
ARL4C	T cell	53.50%	18.30%	0.78	3.90E-36
RPL35	T cell	100.00%	99.50%	0.46	5.34E-36
AQP3	T cell	30.60%	12.10%	0.56	6.86E-36
CD27	T cell	21.50%	0.40%	0.46	6.87E-36
PRKCQ-AS1	T cell	16.70%	1.00%	0.37	7.68E-36
GPSM3	T cell	48.60%	7.80%	0.71	8.68E-36
TBC1D10C	T cell	20.10%	0.40%	0.40	2.21E-35
HMOX2	T cell	44.40%	28.80%	0.46	4.23E-35
LCP1	T cell	52.10%	8.70%	0.74	1.23E-34
RPS14	T cell	100.00%	99.60%	0.50	1.77E-34
SRSF2	T cell	88.20%	83.80%	0.62	2.63E-34
RILPL2	T cell	43.80%	32.10%	0.62	4.29E-34
AP3M2	T cell	24.30%	4.00%	0.49	6.77E-34
CD97	T cell	48.60%	34.10%	0.55	7.99E-34
DNAJC9	T cell	36.80%	14.30%	0.49	2.02E-33
CD5	T cell	19.40%	0.30%	0.40	2.05E-33
RP11-138A9.1	T cell	41.00%	26.80%	0.55	2.67E-33
SIT1	T cell	17.40%	0.20%	0.40	3.44E-33

SRSF7	T cell	73.60%	65.60%	0.60	4.18E-33
H2AFV	T cell	61.10%	50.80%	0.49	5.97E-33
CASP8	T cell	22.90%	4.40%	0.47	9.56E-33
SH2D1A	T cell	20.10%	0.60%	0.44	1.16E-32
YWHAZ	T cell	79.90%	74.20%	0.54	1.37E-32
ARF6	T cell	52.80%	27.80%	0.63	1.62E-32
ITGB7	T cell	22.20%	1.20%	0.41	1.96E-32
RPL14	T cell	100.00%	99.50%	0.46	2.27E-32
CXCR6	T cell	16.70%	0.10%	0.36	3.16E-32
RPS7	T cell	98.60%	99.00%	0.47	9.12E-32
ZBTB1	T cell	36.10%	19.00%	0.53	2.84E-31
RPS12	T cell	99.30%	99.80%	0.50	9.74E-31
PABPC1	T cell	96.50%	92.90%	0.67	1.44E-30
RPL17	T cell	84.70%	80.20%	0.53	2.43E-30
PDE4A	T cell	34.00%	13.50%	0.51	3.10E-30
ITGB2	T cell	52.80%	10.70%	0.82	3.39E-30
ARHGAP15	T cell	29.20%	3.20%	0.58	4.13E-30
SMAP2	T cell	46.50%	11.50%	0.66	4.77E-30
CDKN1B	T cell	36.80%	20.60%	0.47	6.10E-30
RCAN3	T cell	25.70%	4.80%	0.43	7.38E-30
APOBEC3H	T cell	13.90%	0.10%	0.27	8.33E-30
SPN	T cell	18.10%	0.80%	0.39	8.42E-30
LIMD2	T cell	24.30%	2.80%	0.52	9.61E-30
BIRC3	T cell	68.80%	22.80%	0.91	1.09E-29
GNLY	T cell	12.50%	0.50%	1.85	1.13E-29
GZMH	T cell	13.90%	0.20%	0.65	1.43E-29
GZMB	T cell	16.70%	0.30%	0.99	1.50E-29
RP11-51J9.5	T cell	25.00%	6.10%	0.48	2.01E-29
RORA	T cell	54.20%	44.80%	0.53	2.30E-29
MAL	T cell	18.10%	0.60%	0.33	3.74E-29
TIGIT	T cell	14.60%	0.20%	0.52	1.58E-28
TRABD	T cell	30.60%	18.80%	0.40	1.78E-28
PIK3R1	T cell	47.20%	33.20%	0.56	2.36E-28
USP15	T cell	37.50%	28.30%	0.43	3.52E-28
ITK	T cell	15.30%	0.30%	0.33	3.53E-28
RPL13	T cell	100.00%	99.90%	0.47	4.37E-28
SAMD3	T cell	15.30%	0.20%	0.37	4.73E-28
EVI2B	T cell	46.50%	8.20%	0.66	4.75E-28
RPL34	T cell	99.30%	99.90%	0.47	5.34E-28
DUSP4	T cell	43.80%	16.50%	0.88	5.52E-28
AKIRIN1	T cell	41.70%	25.90%	0.46	6.06E-28

PAIP2	T cell	63.90%	58.10%	0.44	8.42E-28
EMB	T cell	47.90%	8.80%	0.65	9.78E-28
CHD2	T cell	51.40%	32.60%	0.56	1.60E-27
CD8B	T cell	13.90%	2.40%	0.39	2.19E-27
AAK1	T cell	41.00%	25.20%	0.47	3.20E-27
IKZF1	T cell	26.40%	2.20%	0.47	3.54E-27
NARF	T cell	41.70%	31.20%	0.40	4.15E-27
RPL18A	T cell	100.00%	99.70%	0.44	4.86E-27
OCIAD2	T cell	39.60%	10.60%	0.48	5.36E-27
IL10RA	T cell	36.80%	6.00%	0.67	1.05E-26
PDE3B	T cell	20.80%	2.60%	0.40	1.24E-26
MIR142	T cell	28.50%	2.80%	0.56	1.43E-26
BATF	T cell	27.10%	2.50%	0.63	1.98E-26
RPL37	T cell	100.00%	99.70%	0.45	2.64E-26
TBCC	T cell	31.20%	16.70%	0.40	3.24E-26
SKAP1	T cell	18.10%	0.60%	0.35	4.73E-26
RPS20	T cell	99.30%	99.80%	0.40	7.21E-26
RALGAPA1	T cell	25.70%	16.30%	0.40	7.65E-26
NKTR	T cell	44.40%	30.20%	0.52	1.11E-25
WDR74	T cell	37.50%	25.10%	0.43	1.23E-25
CCNH	T cell	26.40%	22.10%	0.36	1.32E-25
RASSF5	T cell	31.20%	5.90%	0.55	1.84E-25
RPL32	T cell	99.30%	99.80%	0.43	1.97E-25
JOSD1	T cell	32.60%	12.30%	0.45	2.18E-25
VPS4B	T cell	41.00%	32.80%	0.40	2.25E-25
FBXO34	T cell	27.10%	9.80%	0.46	2.48E-25
IL2RB	T cell	12.50%	0.20%	0.31	2.68E-25
ALOX5AP	T cell	56.20%	14.10%	0.80	2.72E-25
CYTH1	T cell	41.00%	18.50%	0.54	2.82E-25
XCL1	T cell	11.80%	0.10%	0.40	3.51E-25
ARAP2	T cell	20.10%	3.50%	0.39	3.70E-25
TNFAIP3	T cell	71.50%	29.10%	0.80	4.24E-25
RP11-94L15.2	T cell	16.00%	0.40%	0.42	4.63E-25
CAPZA1	T cell	50.00%	38.20%	0.46	5.36E-25
RGS1	T cell	56.20%	16.40%	0.66	1.11E-24
FAM65B	T cell	18.10%	0.90%	0.41	1.24E-24
SCAF11	T cell	59.70%	57.00%	0.43	1.49E-24
SKIL	T cell	56.90%	52.90%	0.50	1.61E-24
NOSIP	T cell	46.50%	42.40%	0.33	1.96E-24
ORAI2	T cell	25.00%	20.60%	0.31	2.05E-24
RPL39	T cell	99.30%	99.30%	0.47	2.30E-24

ARID5A	T cell	63.20%	43.00%	0.57	2.69E-24
RPL26	T cell	99.30%	99.80%	0.41	2.94E-24
FAU	T cell	99.30%	99.50%	0.37	2.96E-24
ZC3HAV1	T cell	48.60%	42.20%	0.45	3.36E-24
GSTK1	T cell	65.30%	59.20%	0.44	3.68E-24
SAFB2	T cell	50.70%	42.30%	0.42	4.12E-24
DDIT4	T cell	82.60%	61.00%	0.80	8.93E-24
FAM102A	T cell	20.10%	6.30%	0.32	2.27E-23
PARP8	T cell	21.50%	4.60%	0.40	3.62E-23
RHOF	T cell	34.70%	6.30%	0.56	5.14E-23
PCSK1N	T cell	11.80%	0.20%	0.33	7.06E-23
PPP1R16B	T cell	15.30%	0.50%	0.26	1.02E-22
LDHB	T cell	66.00%	57.70%	0.46	1.09E-22
RPL18	T cell	100.00%	99.20%	0.43	1.27E-22
RBM38	T cell	20.10%	1.90%	0.31	1.27E-22
RPL27	T cell	98.60%	99.20%	0.40	1.52E-22
DOCK8	T cell	26.40%	6.10%	0.45	1.78E-22
RPL11	T cell	100.00%	99.90%	0.39	1.99E-22
ARHGAP9	T cell	22.20%	3.00%	0.40	4.41E-22
ITM2A	T cell	59.00%	23.10%	0.85	5.73E-22
C9orf142	T cell	35.40%	29.00%	0.34	6.45E-22
RNF168	T cell	27.80%	17.30%	0.38	7.06E-22
CCDC107	T cell	52.80%	47.90%	0.37	7.75E-22
PGAP1	T cell	15.30%	4.80%	0.34	7.80E-22
FGFR1OP2	T cell	47.90%	42.80%	0.36	8.68E-22
RPL13A	T cell	100.00%	99.90%	0.39	9.57E-22
IDS	T cell	56.90%	54.30%	0.37	9.81E-22
GPATCH8	T cell	34.70%	31.20%	0.33	1.17E-21
TNFSF14	T cell	20.10%	1.10%	0.42	1.21E-21
TERF2IP	T cell	61.10%	54.20%	0.42	2.16E-21
GLTSCR2	T cell	92.40%	84.30%	0.53	2.16E-21
RPL19	T cell	100.00%	99.80%	0.39	2.86E-21
RPS6	T cell	100.00%	99.80%	0.41	3.67E-21
TRAF3IP3	T cell	16.70%	0.60%	0.32	6.40E-21
TMSB4X	T cell	100.00%	100.00%	0.33	7.08E-21
SESN3	T cell	22.20%	14.50%	0.36	9.19E-21
HLA-F	T cell	49.30%	25.00%	0.55	1.15E-20
HLA-E	T cell	95.80%	91.20%	0.53	1.33E-20
ARHGEF1	T cell	32.60%	20.40%	0.38	1.42E-20
TOB1	T cell	52.10%	52.80%	0.42	1.51E-20
ANP32E	T cell	39.60%	39.50%	0.33	1.66E-20

PTP4A1	T cell	36.80%	32.40%	0.34	2.58E-20
RPL9	T cell	100.00%	99.40%	0.40	2.89E-20
IQGAP2	T cell	33.30%	5.00%	0.52	3.16E-20
BUB3	T cell	43.80%	40.80%	0.30	4.72E-20
GMFG	T cell	54.90%	15.90%	0.62	4.80E-20
FLT3LG	T cell	20.80%	7.80%	0.32	6.93E-20
CSNK1D	T cell	47.20%	35.90%	0.41	8.81E-20
RP1-313I6.12	T cell	20.80%	5.30%	0.47	9.06E-20
SYTL1	T cell	13.20%	0.40%	0.26	1.30E-19
AC016831.7	T cell	27.80%	11.70%	0.41	1.81E-19
HERPUD2	T cell	25.70%	21.10%	0.31	1.84E-19
CDK6	T cell	18.10%	10.20%	0.33	2.04E-19
UBA52	T cell	97.90%	99.30%	0.33	2.30E-19
DUSP10	T cell	37.50%	27.30%	0.46	2.35E-19
OASL	T cell	20.10%	1.90%	0.38	3.14E-19
PIP4K2A	T cell	22.20%	16.50%	0.32	4.23E-19
TSPYL2	T cell	43.10%	26.80%	0.47	5.71E-19
MYL12A	T cell	90.30%	91.00%	0.46	6.54E-19
GPBP1	T cell	63.90%	57.40%	0.42	7.20E-19
HIST1H4C	T cell	52.10%	43.20%	0.71	8.81E-19
LRRFIP1	T cell	81.90%	64.30%	0.57	1.05E-18
SS18L2	T cell	41.00%	29.60%	0.36	1.15E-18
GIMAP4	T cell	31.20%	5.30%	0.58	1.21E-18
PDE4D	T cell	39.60%	20.50%	0.45	1.45E-18
MED17	T cell	20.10%	13.10%	0.28	2.32E-18
RPL3	T cell	100.00%	99.70%	0.41	2.47E-18
PCSK7	T cell	32.60%	23.20%	0.39	2.64E-18
ZNF331	T cell	50.70%	24.30%	0.68	2.78E-18
PCNX	T cell	20.10%	11.70%	0.28	5.62E-18
USP36	T cell	30.60%	14.60%	0.37	6.06E-18
HMHA1	T cell	27.10%	4.40%	0.47	6.35E-18
PRKCB	T cell	16.70%	1.20%	0.33	6.63E-18
SLC2A3	T cell	61.10%	34.60%	0.59	8.55E-18
RPS18	T cell	100.00%	99.70%	0.44	1.04E-17
PPM1G	T cell	48.60%	39.30%	0.35	1.17E-17
STMN1	T cell	34.00%	16.50%	0.75	1.28E-17
MYO1G	T cell	27.80%	4.50%	0.48	1.31E-17
TESPA1	T cell	14.60%	0.50%	0.33	1.43E-17
KIF21A	T cell	23.60%	13.00%	0.30	1.59E-17
MALAT1	T cell	100.00%	100.00%	0.34	1.79E-17
CEBPZ	T cell	42.40%	39.20%	0.33	2.34E-17

ANXA1	T cell	95.10%	98.50%	0.55	2.51E-17
TMC6	T cell	18.80%	2.10%	0.35	2.85E-17
GCC2	T cell	48.60%	35.60%	0.41	3.21E-17
FMNL1	T cell	34.70%	9.80%	0.44	3.27E-17
TMEM123	T cell	57.60%	43.80%	0.45	3.94E-17
PMF1	T cell	41.70%	36.70%	0.29	4.25E-17
TMEM2	T cell	34.70%	23.30%	0.43	4.70E-17
NCOR1	T cell	54.90%	52.20%	0.33	4.83E-17
PDE4B	T cell	43.10%	21.60%	0.57	5.82E-17
RPL6	T cell	100.00%	99.60%	0.36	6.98E-17
DDX24	T cell	74.30%	68.60%	0.40	7.58E-17
CD28	T cell	11.80%	0.30%	0.27	8.26E-17
FNBP4	T cell	45.80%	34.50%	0.39	9.31E-17
TSTD1	T cell	28.50%	5.90%	0.40	1.01E-16
RABIF	T cell	25.00%	17.20%	0.27	1.04E-16
IDI1	T cell	48.60%	45.20%	0.39	1.06E-16
TNFRSF4	T cell	16.70%	1.60%	0.44	1.23E-16
DDX27	T cell	36.10%	28.40%	0.33	1.48E-16
HBS1L	T cell	27.80%	19.60%	0.30	1.63E-16
BCAS2	T cell	39.60%	36.80%	0.29	1.66E-16
PPP2CA	T cell	47.20%	46.80%	0.30	1.73E-16
ELF1	T cell	57.60%	52.00%	0.41	1.86E-16
FXYD7	T cell	11.10%	0.60%	0.30	3.43E-16
TAGAP	T cell	25.00%	3.60%	0.48	3.57E-16
TAP1	T cell	34.70%	25.40%	0.38	3.65E-16
METTL9	T cell	52.10%	48.70%	0.34	3.74E-16
ODF2L	T cell	39.60%	23.00%	0.39	3.94E-16
C19orf43	T cell	78.50%	76.50%	0.37	4.53E-16
DNAJB6	T cell	77.10%	71.00%	0.42	4.75E-16
RPS3A	T cell	100.00%	99.60%	0.39	4.80E-16
CCM2	T cell	25.70%	19.70%	0.26	5.21E-16
BAZ1A	T cell	60.40%	57.00%	0.43	5.79E-16
BDP1	T cell	47.90%	38.00%	0.37	7.56E-16
AES	T cell	45.10%	35.40%	0.37	9.60E-16
TSC22D4	T cell	28.50%	23.10%	0.26	1.08E-15
SLAMF1	T cell	13.20%	0.50%	0.31	1.10E-15
PHF1	T cell	34.70%	32.90%	0.28	1.26E-15
ZC3H12D	T cell	10.40%	0.30%	0.25	1.33E-15
FAM46C	T cell	31.20%	11.90%	0.40	1.38E-15
RNF213	T cell	37.50%	31.50%	0.36	1.48E-15
GYPC	T cell	59.70%	58.80%	0.37	1.71E-15

FAS	T cell	26.40%	15.40%	0.31	1.71E-15
SELL	T cell	10.40%	2.60%	0.34	2.74E-15
RNF149	T cell	43.10%	29.80%	0.40	2.79E-15
TNFRSF25	T cell	13.90%	1.20%	0.32	3.92E-15
CDK17	T cell	20.10%	11.40%	0.29	4.45E-15
VAMP8	T cell	48.60%	18.90%	0.31	4.63E-15
MTERFD2	T cell	24.30%	9.30%	0.33	4.77E-15
PERP	T cell	39.60%	21.70%	0.45	5.97E-15
MGAT4A	T cell	27.80%	5.60%	0.44	6.78E-15
MRPL1	T cell	29.90%	18.60%	0.35	7.00E-15
RPL10	T cell	100.00%	99.90%	0.33	7.27E-15
LPXN	T cell	34.00%	9.30%	0.49	7.32E-15
RBM39	T cell	80.60%	85.50%	0.35	7.39E-15
POLE3	T cell	37.50%	34.10%	0.28	8.24E-15
TPM3	T cell	80.60%	72.50%	0.46	1.03E-14
SEP1	T cell	32.60%	20.00%	0.32	1.09E-14
RP11-345J4.5	T cell	44.40%	39.00%	0.31	1.14E-14
AMICA1	T cell	22.90%	2.60%	0.41	1.32E-14
KLF3	T cell	36.10%	24.60%	0.39	1.39E-14
PITPNC1	T cell	16.70%	2.60%	0.31	1.56E-14
RPL29	T cell	100.00%	99.30%	0.33	1.59E-14
RPL35A	T cell	100.00%	99.60%	0.35	1.62E-14
BCL2	T cell	16.00%	2.80%	0.31	1.88E-14
JAK1	T cell	61.80%	51.20%	0.39	2.72E-14
MAP2K1	T cell	33.30%	18.10%	0.40	3.06E-14
MATK	T cell	16.00%	1.50%	0.37	3.82E-14
SEMA4D	T cell	13.90%	1.50%	0.27	4.03E-14
EEF1D	T cell	99.30%	97.70%	0.37	5.52E-14
PPP1CA	T cell	55.60%	57.10%	0.26	5.69E-14
OFD1	T cell	27.80%	19.10%	0.32	6.58E-14
CCDC12	T cell	49.30%	40.30%	0.32	7.79E-14
GLUD1	T cell	32.60%	35.00%	0.29	1.03E-13
FKBP5	T cell	42.40%	27.40%	0.41	1.03E-13
CNN2	T cell	26.40%	13.30%	0.35	1.30E-13
DNAJC1	T cell	27.80%	21.00%	0.32	1.49E-13
GPR132	T cell	20.10%	3.00%	0.40	1.53E-13
MAPK1IP1L	T cell	43.10%	36.50%	0.29	1.94E-13
CYBA	T cell	90.30%	77.80%	0.49	2.03E-13
EPC1	T cell	44.40%	43.30%	0.28	2.03E-13
HINT1	T cell	88.20%	90.80%	0.33	2.24E-13
ZCCHC11	T cell	27.10%	23.10%	0.27	2.84E-13

ARID4B	T cell	59.00%	62.10%	0.31	2.86E-13
RP11-138A9.2	T cell	27.80%	17.00%	0.42	3.45E-13
DHX36	T cell	47.20%	48.40%	0.27	3.46E-13
ATP5L	T cell	91.00%	93.80%	0.31	3.57E-13
EIF3J	T cell	54.90%	55.10%	0.31	4.61E-13
GTF3A	T cell	58.30%	59.20%	0.27	6.13E-13
PPM1K	T cell	19.40%	15.90%	0.25	8.81E-13
ARPP19	T cell	43.10%	36.20%	0.29	1.06E-12
RNF166	T cell	23.60%	5.80%	0.35	1.10E-12
SELK	T cell	77.80%	82.50%	0.32	1.38E-12
PIM2	T cell	23.60%	6.40%	0.38	1.39E-12
FXYD5	T cell	85.40%	82.40%	0.45	1.80E-12
CBLB	T cell	27.10%	20.80%	0.29	1.90E-12
FBL	T cell	51.40%	53.30%	0.26	2.05E-12
C12orf75	T cell	32.60%	16.90%	0.44	2.37E-12
CORO7	T cell	20.80%	6.00%	0.33	2.42E-12
KRAS	T cell	35.40%	32.60%	0.27	3.28E-12
CREBRF	T cell	38.90%	39.80%	0.28	3.65E-12
GIMAP5	T cell	26.40%	4.60%	0.38	3.76E-12
ABCF1	T cell	47.20%	43.30%	0.29	4.11E-12
WAPAL	T cell	38.90%	31.20%	0.30	4.33E-12
FAM126B	T cell	20.80%	9.90%	0.28	4.38E-12
TCF7	T cell	29.20%	12.50%	0.34	4.84E-12
CLEC2B	T cell	45.80%	16.40%	0.54	6.02E-12
HMGN1	T cell	59.70%	60.90%	0.29	6.07E-12
RASGRP2	T cell	19.40%	3.80%	0.30	7.41E-12
STAG2	T cell	40.30%	38.20%	0.27	7.71E-12
SRRM1	T cell	61.10%	67.00%	0.25	8.39E-12
EPB41	T cell	16.00%	3.90%	0.26	1.04E-11
SLFN5	T cell	24.30%	11.50%	0.33	1.16E-11
ABRACL	T cell	47.20%	35.60%	0.36	1.17E-11
UBE2D2	T cell	61.80%	64.10%	0.25	1.49E-11
RAB9A	T cell	27.80%	23.80%	0.28	1.56E-11
ZC3H18	T cell	29.20%	16.00%	0.31	1.66E-11
LINC-PINT	T cell	28.50%	23.80%	0.26	2.09E-11
PIM1	T cell	38.90%	27.10%	0.38	2.17E-11
SF3B1	T cell	59.00%	54.20%	0.32	2.53E-11
ARHGAP30	T cell	23.60%	5.40%	0.44	2.60E-11
SELT	T cell	63.90%	64.50%	0.26	3.07E-11
RBM4	T cell	22.20%	11.40%	0.27	5.71E-11
CYLD	T cell	38.90%	32.50%	0.30	5.73E-11

RPL4	T cell	98.60%	97.80%	0.35	6.16E-11
RPS4X	T cell	100.00%	99.80%	0.34	6.20E-11
CEP350	T cell	31.20%	25.10%	0.28	6.48E-11
SPSB3	T cell	36.80%	31.60%	0.26	7.44E-11
RHOG	T cell	46.50%	42.70%	0.34	8.34E-11
DDX39B	T cell	17.40%	10.50%	0.26	1.14E-10
MSN	T cell	67.40%	69.90%	0.29	1.17E-10
MFNG	T cell	20.80%	3.40%	0.31	1.54E-10
RPL10A	T cell	97.90%	99.40%	0.28	2.16E-10
ANP32B	T cell	59.70%	61.50%	0.27	2.25E-10
YWHAB	T cell	75.70%	82.60%	0.25	2.57E-10
PRKCH	T cell	23.60%	5.60%	0.35	2.88E-10
CDK11A	T cell	33.30%	22.20%	0.32	3.58E-10
PSME1	T cell	81.20%	78.80%	0.34	4.25E-10
CCND2	T cell	45.80%	42.70%	0.27	4.30E-10
PNISR	T cell	62.50%	61.00%	0.32	5.05E-10
PPP1CB	T cell	63.20%	58.50%	0.33	5.35E-10
RARRES3	T cell	38.20%	25.90%	0.37	5.42E-10
TES	T cell	34.70%	33.20%	0.26	5.90E-10
UBA6	T cell	29.20%	13.90%	0.30	6.03E-10
HCLS1	T cell	31.90%	9.90%	0.44	6.11E-10
JUND	T cell	63.90%	59.60%	0.36	6.15E-10
C20orf24	T cell	36.10%	31.20%	0.25	6.40E-10
PRRC2C	T cell	71.50%	73.30%	0.28	7.02E-10
HOXB2	T cell	12.50%	2.10%	0.29	7.48E-10
SETD2	T cell	29.20%	20.00%	0.28	9.04E-10
ITSN2	T cell	34.70%	34.00%	0.26	9.08E-10
PDE7A	T cell	19.40%	8.30%	0.25	9.15E-10
IL27RA	T cell	16.70%	4.80%	0.27	1.06E-09
KMT2A	T cell	33.30%	27.60%	0.26	1.25E-09
ABT1	T cell	32.60%	19.90%	0.28	1.49E-09
HECA	T cell	30.60%	19.60%	0.28	1.60E-09
CELF1	T cell	30.60%	25.60%	0.28	1.81E-09
AKAP13	T cell	60.40%	61.90%	0.31	1.83E-09
SNRNP200	T cell	28.50%	20.10%	0.28	1.92E-09
PRMT2	T cell	45.80%	42.30%	0.28	2.61E-09
PRMT10	T cell	19.40%	6.50%	0.35	4.10E-09
MYO1F	T cell	17.40%	3.60%	0.31	4.74E-09
TIPARP	T cell	59.00%	51.10%	0.45	4.85E-09
ZFP36	T cell	95.80%	92.40%	0.41	4.96E-09
BRD9	T cell	20.10%	10.70%	0.28	6.44E-09

ANKRD11	T cell	49.30%	43.30%	0.30	9.21E-09
PRKX	T cell	13.90%	3.10%	0.31	9.58E-09
NBEAL1	T cell	79.90%	79.30%	0.30	1.59E-08
ATXN7	T cell	27.10%	17.30%	0.26	1.61E-08
CDC42	T cell	77.80%	78.00%	0.34	1.65E-08
RPL36AL	T cell	95.10%	96.70%	0.29	1.76E-08
RPS8	T cell	98.60%	99.70%	0.30	1.94E-08
RPL37A	T cell	100.00%	99.90%	0.25	2.17E-08
CNTRL	T cell	13.20%	7.70%	0.25	2.49E-08
SNRK	T cell	20.10%	9.80%	0.25	4.03E-08
FAM107B	T cell	35.40%	30.70%	0.27	4.06E-08
CALM1	T cell	95.10%	96.90%	0.32	4.15E-08
MIR24-2	T cell	29.90%	31.60%	0.32	4.93E-08
TNRC6B	T cell	47.90%	46.00%	0.28	5.47E-08
PRDM1	T cell	25.70%	6.90%	0.37	6.36E-08
RBM8A	T cell	78.50%	78.70%	0.29	7.33E-08
SELPLG	T cell	20.10%	4.30%	0.31	1.59E-07
EEF1A1	T cell	100.00%	100.00%	0.27	2.25E-07
PFN1	T cell	93.80%	96.00%	0.31	2.63E-07
NIPBL	T cell	46.50%	41.10%	0.26	2.74E-07
PHF20	T cell	39.60%	32.50%	0.27	3.66E-07
RAP2B	T cell	22.20%	12.20%	0.33	4.21E-07
PLIN2	T cell	52.80%	30.70%	0.47	4.82E-07
ERO1LB	T cell	29.90%	25.20%	0.27	4.84E-07
SOCS1	T cell	11.80%	3.60%	0.26	6.61E-07
MARCKSL1	T cell	25.00%	18.50%	0.29	1.16E-06
REL	T cell	59.00%	46.80%	0.57	1.17E-06
RP11-356I2.4	T cell	12.50%	2.70%	0.26	2.02E-06
CD44	T cell	90.30%	90.20%	0.36	2.05E-06
SP100	T cell	53.50%	44.90%	0.29	2.77E-06
SVIP	T cell	32.60%	20.40%	0.29	3.47E-06
ATP2B1	T cell	53.50%	49.90%	0.34	5.41E-06
EEF1B2	T cell	93.80%	94.90%	0.31	5.81E-06
WAS	T cell	17.40%	3.90%	0.28	7.16E-06
MRPS6	T cell	53.50%	49.00%	0.30	8.10E-06
CFL1	T cell	98.60%	98.40%	0.29	8.39E-06
ARPC2	T cell	89.60%	91.70%	0.25	1.94E-05
GIMAP1	T cell	16.00%	4.20%	0.31	2.66E-05
NPM1	T cell	98.60%	96.40%	0.28	4.25E-05
CELF2	T cell	50.00%	41.90%	0.30	4.41E-05
HSPA8	T cell	97.90%	96.10%	0.36	5.96E-05

SERPINB9	T cell	34.00%	18.50%	0.44	7.80E-05
GNA15	T cell	20.80%	5.90%	0.32	7.94E-05
HNRNPF	T cell	67.40%	66.70%	0.25	1.30E-04
PLK3	T cell	33.30%	24.10%	0.28	1.97E-04
FAM49B	T cell	33.30%	19.40%	0.34	2.75E-04
RCSD1	T cell	18.10%	4.60%	0.28	2.76E-04
DNTTIP2	T cell	47.20%	42.20%	0.25	5.78E-04
FERMT3	T cell	22.90%	10.00%	0.30	6.04E-04
EVI2A	T cell	27.10%	15.20%	0.34	1.81E-03
RAB11FIP1	T cell	15.30%	5.30%	0.27	4.78E-03
ACRC	T cell	13.90%	8.10%	0.27	5.69E-03
CD4	T cell	29.90%	15.90%	0.31	8.46E-03
DUSP2	T cell	18.10%	5.90%	0.25	1.22E-02
RNASET2	T cell	38.20%	31.60%	0.29	1.55E-02
C1orf56	T cell	27.10%	22.30%	0.27	4.60E-02
CTSC	T cell	22.90%	14.20%	0.37	7.23E-02
UCP2	T cell	29.90%	17.20%	0.32	1.22E-01
PLP2	T cell	84.00%	79.50%	0.29	1.38E-01
ISG15	T cell	38.20%	30.50%	0.29	1.47E-01
ARRB2	T cell	27.10%	13.10%	0.30	1.83E-01
RN7SL1	T cell	13.90%	11.40%	0.26	6.49E-01
CDC42EP3	T cell	27.80%	18.10%	0.27	1.00E+00
TXNIP	T cell	52.10%	44.10%	0.30	1.00E+00
RPS4Y1	T cell	47.20%	36.20%	0.28	1.00E+00
RGS2	T cell	64.60%	59.50%	0.28	1.00E+00
TPSAB1	Mast cell	100.00%	10.70%	5.34	0.00E+00
CPA3	Mast cell	97.50%	1.00%	2.52	1.00E-270
RGS13	Mast cell	85.00%	1.40%	1.94	3.27E-207
SELK	Mast cell	97.50%	82.20%	1.45	1.07E-197
VWA5A	Mast cell	82.50%	17.20%	1.38	1.70E-192
SLC18A2	Mast cell	77.50%	0.30%	1.49	1.31E-182
GLUL	Mast cell	98.30%	75.90%	1.81	4.13E-180
CD69	Mast cell	84.20%	3.00%	2.50	1.96E-164
GCSAML	Mast cell	69.20%	0.30%	1.44	8.03E-156
HPGDS	Mast cell	91.70%	6.60%	2.10	7.39E-150
CALB2	Mast cell	64.20%	0.20%	1.16	1.88E-144
RAB27B	Mast cell	64.20%	0.40%	1.21	8.02E-142
C1orf186	Mast cell	62.50%	0.20%	1.09	7.47E-138
HDC	Mast cell	60.00%	0.40%	1.23	1.25E-133
KIT	Mast cell	61.70%	0.60%	1.12	4.35E-133
IL1RL1	Mast cell	71.70%	1.20%	1.44	1.62E-129

CTSG	Mast cell	50.80%	1.00%	1.53	1.14E-118
SRGN	Mast cell	100.00%	43.40%	2.15	2.95E-116
RAC2	Mast cell	77.50%	4.30%	1.36	2.62E-113
GPR65	Mast cell	66.70%	2.00%	1.49	4.73E-111
SAMSN1	Mast cell	83.30%	5.20%	1.87	2.36E-110
CST7	Mast cell	73.30%	2.00%	1.26	3.70E-109
ACSL4	Mast cell	67.50%	21.00%	1.01	6.15E-105
CREM	Mast cell	81.70%	45.20%	1.62	5.31E-104
FCER1A	Mast cell	79.20%	4.80%	1.13	1.07E-102
HPGD	Mast cell	66.70%	3.80%	1.32	3.51E-102
RP11-354E11.2	Mast cell	42.50%	0.10%	0.78	3.24E-93
GATA2	Mast cell	59.20%	5.50%	1.25	1.14E-92
BIRC3	Mast cell	83.30%	22.70%	2.04	2.91E-92
RGS2	Mast cell	92.50%	59.20%	1.68	1.12E-89
SDCBP	Mast cell	97.50%	90.50%	1.13	1.20E-82
AC020571.3	Mast cell	41.70%	0.30%	0.90	1.60E-81
ELF1	Mast cell	80.00%	51.70%	0.95	1.63E-75
RAB38	Mast cell	42.50%	1.60%	0.95	6.87E-75
DUSP14	Mast cell	73.30%	48.90%	0.96	2.68E-74
FCER1G	Mast cell	94.20%	20.10%	1.02	1.10E-70
KRT1	Mast cell	35.80%	0.20%	0.69	3.05E-70
REL	Mast cell	88.30%	46.50%	1.38	3.70E-67
SMYD3	Mast cell	51.70%	10.40%	0.70	6.42E-67
VAPA	Mast cell	95.00%	77.90%	0.82	1.59E-65
LAX1	Mast cell	36.70%	0.40%	0.67	2.90E-65
RHOH	Mast cell	51.70%	2.10%	1.00	1.52E-63
CTNNBL1	Mast cell	62.50%	28.00%	0.74	2.91E-63
ADRB2	Mast cell	58.30%	11.20%	0.95	1.56E-62
CD52	Mast cell	61.70%	4.60%	1.38	4.85E-61
PLIN2	Mast cell	65.80%	30.60%	1.36	1.67E-60
FTH1	Mast cell	100.00%	100.00%	0.97	3.19E-60
TYROBP	Mast cell	89.20%	21.20%	0.57	3.57E-60
MAOB	Mast cell	54.20%	15.70%	0.78	9.94E-60
CPEB4	Mast cell	68.30%	34.10%	0.91	1.46E-58
RGS1	Mast cell	81.70%	16.20%	1.95	1.50E-58
RPL36AL	Mast cell	100.00%	96.60%	0.68	1.29E-55
BATF	Mast cell	45.80%	2.30%	0.94	4.86E-55
AP1S3	Mast cell	36.70%	1.50%	0.62	5.25E-55
LAT	Mast cell	46.70%	3.00%	0.77	1.20E-54
SDPR	Mast cell	50.00%	6.20%	1.06	2.14E-54
ALOX5AP	Mast cell	79.20%	13.90%	1.19	5.19E-53

NSMCE1	Mast cell	60.80%	35.90%	0.64	5.47E-53
CKLF	Mast cell	69.20%	36.70%	0.79	2.49E-52
MS4A2	Mast cell	25.80%	0.00%	0.41	6.66E-52
ZNF331	Mast cell	59.20%	24.30%	1.12	1.67E-50
TNIK	Mast cell	35.80%	2.00%	0.55	7.73E-50
RPL34	Mast cell	100.00%	99.90%	0.63	4.36E-49
RBM8A	Mast cell	91.70%	78.60%	0.72	1.25E-48
CPM	Mast cell	63.30%	10.80%	1.07	2.88E-48
CDC42EP3	Mast cell	65.00%	17.70%	1.09	5.24E-48
LAPTM5	Mast cell	87.50%	21.60%	1.14	7.25E-48
SVOPL	Mast cell	20.80%	0.00%	0.30	3.61E-47
TESPA1	Mast cell	29.20%	0.30%	0.54	6.45E-46
ARHGDIB	Mast cell	82.50%	20.90%	1.13	1.06E-45
LAT2	Mast cell	50.00%	4.60%	0.72	2.84E-44
CTD-3203P2.2	Mast cell	26.70%	0.50%	0.53	7.22E-44
PTGS2	Mast cell	70.00%	28.60%	1.31	7.34E-44
NTRK1	Mast cell	25.00%	0.80%	0.47	2.76E-43
PAG1	Mast cell	43.30%	5.10%	0.74	2.00E-42
RPS29	Mast cell	100.00%	99.40%	0.60	6.01E-42
GRAP2	Mast cell	26.70%	0.30%	0.36	9.57E-42
DDIT4	Mast cell	85.00%	61.00%	1.16	5.23E-41
ARHGAP25	Mast cell	30.00%	1.20%	0.60	5.20E-40
KLRG1	Mast cell	31.70%	1.40%	0.53	5.43E-40
ASAH1	Mast cell	76.70%	51.20%	0.74	1.54E-39
RPL7	Mast cell	100.00%	99.70%	0.50	1.59E-39
CD82	Mast cell	72.50%	34.30%	0.81	1.95E-39
PAK1	Mast cell	43.30%	11.50%	0.67	3.36E-39
DUSP6	Mast cell	58.30%	17.90%	1.04	3.71E-39
STX11	Mast cell	65.00%	15.40%	0.91	8.51E-39
RPL21	Mast cell	100.00%	99.90%	0.47	4.12E-38
RAB9A	Mast cell	41.70%	23.70%	0.63	1.19E-37
GALNT3	Mast cell	26.70%	0.70%	0.47	1.31E-37
STK17B	Mast cell	65.00%	16.80%	0.85	1.32E-37
MARCH1	Mast cell	33.30%	15.30%	0.52	4.95E-37
CD37	Mast cell	61.70%	10.20%	0.91	5.58E-37
TDRD3	Mast cell	37.50%	13.10%	0.56	7.28E-37
NDUFA4	Mast cell	100.00%	92.90%	0.65	1.33E-36
NFATC1	Mast cell	35.00%	20.60%	0.54	1.77E-36
TANK	Mast cell	65.00%	38.70%	0.66	3.28E-36
BTK	Mast cell	41.70%	3.60%	0.68	8.94E-36
TPSD1	Mast cell	20.00%	0.10%	0.31	2.01E-35

CAPG	Mast cell	90.00%	63.10%	0.89	3.21E-35
PFDN5	Mast cell	99.20%	98.20%	0.54	6.87E-35
DUSP10	Mast cell	59.20%	27.00%	0.73	7.84E-35
TPSG1	Mast cell	18.30%	0.00%	0.36	1.14E-34
VAMP8	Mast cell	70.00%	18.70%	0.51	1.59E-34
ANKRD28	Mast cell	72.50%	58.90%	0.78	2.52E-34
CD83	Mast cell	78.30%	26.00%	1.21	3.46E-34
CLIC1	Mast cell	100.00%	94.70%	0.64	9.59E-33
FDX1	Mast cell	31.70%	14.10%	0.63	1.02E-32
PTGS1	Mast cell	44.20%	13.50%	0.62	1.26E-32
GPBP1	Mast cell	75.80%	57.30%	0.62	2.49E-32
CD44	Mast cell	98.30%	90.10%	0.72	1.03E-31
TESC	Mast cell	30.00%	1.50%	0.47	1.59E-31
PHACTR1	Mast cell	46.70%	6.20%	0.78	3.05E-31
SVIP	Mast cell	51.70%	20.30%	0.63	3.59E-31
RP11-501J20.5	Mast cell	15.80%	0.00%	0.26	6.83E-31
ANXA1	Mast cell	99.20%	98.50%	0.86	1.13E-30
HMGN3	Mast cell	79.20%	65.40%	0.59	1.15E-30
LEO1	Mast cell	44.20%	17.10%	0.52	2.02E-30
NTM	Mast cell	29.20%	5.10%	0.53	2.13E-30
ENPP3	Mast cell	17.50%	0.40%	0.36	4.42E-30
TSTD1	Mast cell	37.50%	5.90%	0.58	1.45E-29
RPS27A	Mast cell	100.00%	99.80%	0.42	4.63E-29
RP11-557H15.4	Mast cell	15.00%	0.20%	0.29	5.61E-29
TRIM63	Mast cell	15.00%	0.00%	0.41	6.86E-29
LMNB1	Mast cell	20.00%	1.50%	0.46	1.96E-28
RASSF5	Mast cell	37.50%	5.90%	0.64	3.16E-28
SCIN	Mast cell	19.20%	0.90%	0.40	1.17E-27
VPS37B	Mast cell	42.50%	14.00%	0.60	2.68E-27
GALC	Mast cell	34.20%	6.70%	0.48	5.62E-27
ALDH1A1	Mast cell	47.50%	6.90%	0.69	7.60E-27
B4GALT5	Mast cell	50.80%	19.10%	0.55	9.54E-27
PTPN1	Mast cell	55.80%	34.20%	0.61	1.99E-26
RPL14	Mast cell	100.00%	99.50%	0.48	3.05E-26
SUB1	Mast cell	88.30%	82.20%	0.53	1.19E-25
RPL26	Mast cell	100.00%	99.70%	0.38	1.58E-25
RPS27	Mast cell	100.00%	99.90%	0.38	5.82E-25
GNPTAB	Mast cell	31.70%	9.80%	0.49	8.42E-25
RHOG	Mast cell	57.50%	42.60%	0.61	1.05E-24
PRNP	Mast cell	78.30%	68.80%	0.57	2.90E-24

METTL21A	Mast cell	26.70%	13.50%	0.45	2.94E-24
AREG	Mast cell	22.50%	1.60%	0.60	3.54E-24
SGK1	Mast cell	91.70%	73.50%	0.82	4.13E-24
MAPRE1	Mast cell	59.20%	43.90%	0.48	4.90E-24
RNF145	Mast cell	47.50%	30.20%	0.55	1.64E-23
TNFRSF4	Mast cell	25.80%	1.60%	0.36	2.06E-23
RPL37A	Mast cell	100.00%	99.90%	0.44	2.47E-23
KRT19	Mast cell	12.50%	0.00%	0.27	5.83E-23
RPS14	Mast cell	100.00%	99.60%	0.37	2.38E-22
LPXN	Mast cell	40.00%	9.30%	0.71	5.19E-22
BST2	Mast cell	70.80%	28.10%	0.74	7.56E-22
LYST	Mast cell	38.30%	18.00%	0.48	2.14E-21
RPL32	Mast cell	100.00%	99.80%	0.38	3.57E-21
ARHGAP18	Mast cell	59.20%	16.90%	0.62	1.04E-20
ID2	Mast cell	94.20%	89.50%	0.85	1.05E-20
SAT1	Mast cell	100.00%	95.30%	0.57	1.16E-20
RENBP	Mast cell	35.80%	5.40%	0.56	3.13E-20
MLPH	Mast cell	19.20%	1.20%	0.28	6.47E-20
NMT2	Mast cell	28.30%	10.60%	0.41	7.78E-20
RPS25	Mast cell	100.00%	99.80%	0.36	1.14E-19
RPL41	Mast cell	100.00%	100.00%	0.29	1.15E-19
RSL24D1	Mast cell	83.30%	74.40%	0.47	1.79E-19
STARD10	Mast cell	21.70%	5.80%	0.35	2.48E-19
AQP2	Mast cell	10.80%	0.10%	0.26	2.60E-19
ALOX5	Mast cell	29.20%	3.50%	0.48	2.89E-19
STMN1	Mast cell	53.30%	16.30%	0.69	4.34E-19
MAP3K8	Mast cell	70.80%	52.20%	0.60	4.49E-19
SLC26A2	Mast cell	37.50%	17.20%	0.42	4.59E-19
HAVCR2	Mast cell	36.70%	5.60%	0.55	5.21E-19
IL4R	Mast cell	33.30%	12.00%	0.47	5.46E-19
NFKBIA	Mast cell	99.20%	92.30%	0.62	7.07E-19
ALG13	Mast cell	41.70%	26.70%	0.42	5.20E-18
PRKX	Mast cell	26.70%	3.00%	0.44	5.44E-18
RP11-620J15.3	Mast cell	30.80%	9.40%	0.37	6.30E-18
LTC4S	Mast cell	40.00%	7.10%	0.60	6.87E-18
SEC22C	Mast cell	42.50%	28.50%	0.40	6.94E-18
CTSW	Mast cell	20.80%	1.40%	0.30	7.04E-18
RPS18	Mast cell	100.00%	99.70%	0.26	7.36E-18
C21orf91	Mast cell	28.30%	7.60%	0.39	1.05E-17
UBB	Mast cell	98.30%	99.10%	0.47	1.70E-17
SNHG8	Mast cell	85.80%	71.60%	0.51	2.15E-17

PLGRKT	Mast cell	39.20%	24.20%	0.37	3.06E-17
RPL11	Mast cell	100.00%	99.90%	0.35	3.58E-17
PRDX1	Mast cell	97.50%	96.10%	0.54	3.61E-17
H3F3B	Mast cell	100.00%	99.70%	0.44	6.85E-17
HEY1	Mast cell	23.30%	3.50%	0.51	1.07E-16
TMEM233	Mast cell	18.30%	2.10%	0.31	2.31E-16
RPL39	Mast cell	100.00%	99.30%	0.32	4.56E-16
MAPK6	Mast cell	50.00%	32.00%	0.46	7.24E-16
BHLHE40	Mast cell	80.80%	64.20%	0.56	8.15E-16
FER	Mast cell	36.70%	16.20%	0.40	1.24E-15
FAU	Mast cell	100.00%	99.50%	0.34	1.97E-15
RPS28	Mast cell	100.00%	99.80%	0.32	2.25E-15
SFT2D1	Mast cell	52.50%	51.30%	0.32	2.53E-15
RPS13	Mast cell	100.00%	99.50%	0.28	5.65E-15
TMEM66	Mast cell	95.00%	85.30%	0.47	6.33E-15
YME1L1	Mast cell	50.80%	45.60%	0.39	6.74E-15
MBOAT7	Mast cell	26.70%	8.00%	0.37	9.65E-15
RPS15	Mast cell	100.00%	99.80%	0.30	1.07E-14
ACOT7	Mast cell	37.50%	18.00%	0.44	1.14E-14
ZFP91	Mast cell	34.20%	24.10%	0.35	1.15E-14
SELT	Mast cell	68.30%	64.50%	0.36	1.40E-14
C10orf128	Mast cell	28.30%	4.10%	0.39	1.79E-14
DDX5	Mast cell	100.00%	98.10%	0.49	2.31E-14
RPL38	Mast cell	100.00%	99.20%	0.40	2.89E-14
SLC2A3	Mast cell	63.30%	34.60%	0.60	3.05E-14
RAB33A	Mast cell	15.80%	2.10%	0.29	4.25E-14
ATP6V1F	Mast cell	80.00%	75.50%	0.41	5.78E-14
DBI	Mast cell	91.70%	83.80%	0.45	6.61E-14
NFKB1	Mast cell	63.30%	34.20%	0.63	8.10E-14
DNAJB9	Mast cell	69.20%	61.10%	0.45	8.36E-14
RPL27A	Mast cell	100.00%	99.60%	0.32	1.09E-13
FAM195A	Mast cell	30.80%	13.50%	0.35	1.56E-13
GABPB1	Mast cell	23.30%	10.50%	0.33	1.75E-13
RILPL2	Mast cell	59.20%	31.90%	0.53	1.81E-13
VEGFA	Mast cell	46.70%	22.30%	0.49	2.13E-13
CD53	Mast cell	42.50%	10.50%	0.46	2.26E-13
CLCN3	Mast cell	32.50%	17.70%	0.35	2.38E-13
GMFG	Mast cell	50.00%	16.10%	0.37	2.71E-13
RPL30	Mast cell	100.00%	99.60%	0.35	2.89E-13
SLC1A5	Mast cell	48.30%	36.50%	0.41	4.75E-13
AKAP13	Mast cell	77.50%	61.70%	0.50	4.97E-13

SLC2A6	Mast cell	23.30%	4.60%	0.34	6.59E-13
FAM107B	Mast cell	51.70%	30.60%	0.45	7.13E-13
CSF2	Mast cell	13.30%	0.60%	0.85	7.26E-13
TNFRSF9	Mast cell	18.30%	3.30%	0.29	7.33E-13
TRIM24	Mast cell	18.30%	6.80%	0.29	7.78E-13
HAX1	Mast cell	50.80%	53.00%	0.26	8.12E-13
C1orf228	Mast cell	15.00%	0.70%	0.25	8.37E-13
EIF1	Mast cell	100.00%	99.80%	0.28	1.07E-12
EMR2	Mast cell	20.00%	2.20%	0.31	1.19E-12
TPST2	Mast cell	35.80%	20.50%	0.37	1.92E-12
RPL35	Mast cell	100.00%	99.50%	0.31	2.08E-12
SYAP1	Mast cell	55.00%	40.90%	0.40	2.50E-12
TNFRSF18	Mast cell	27.50%	4.70%	0.39	3.32E-12
RPL35A	Mast cell	100.00%	99.60%	0.30	3.42E-12
PHF20	Mast cell	42.50%	32.50%	0.37	4.27E-12
FTL	Mast cell	100.00%	99.80%	0.28	4.33E-12
LDHB	Mast cell	70.80%	57.70%	0.43	4.37E-12
RPS4Y1	Mast cell	55.00%	36.20%	0.69	4.57E-12
M6PR	Mast cell	50.00%	34.30%	0.38	9.65E-12
PRDX6	Mast cell	85.80%	80.00%	0.37	1.25E-11
MSRA	Mast cell	25.00%	6.90%	0.36	1.38E-11
RPS4X	Mast cell	100.00%	99.80%	0.34	1.51E-11
PTPRC	Mast cell	36.70%	10.60%	0.28	1.72E-11
IL18R1	Mast cell	15.80%	1.00%	0.27	1.93E-11
MECP2	Mast cell	38.30%	26.10%	0.36	2.27E-11
SYTL3	Mast cell	31.70%	7.60%	0.39	2.43E-11
POLR3GL	Mast cell	46.70%	39.90%	0.30	3.21E-11
OSTF1	Mast cell	50.80%	35.50%	0.38	3.37E-11
STK4	Mast cell	40.80%	17.00%	0.55	3.53E-11
DHRS9	Mast cell	11.70%	1.00%	0.45	3.71E-11
PTPN7	Mast cell	18.30%	1.60%	0.33	3.85E-11
RPS6	Mast cell	100.00%	99.80%	0.27	4.65E-11
U2AF1	Mast cell	84.20%	77.00%	0.39	7.50E-11
UBE2D3	Mast cell	90.00%	85.00%	0.38	9.47E-11
STXBP2	Mast cell	27.50%	4.60%	0.35	1.00E-10
RPL31	Mast cell	100.00%	99.60%	0.31	1.63E-10
PTMA	Mast cell	100.00%	99.90%	0.28	2.12E-10
RASGEF1B	Mast cell	49.20%	22.40%	0.60	2.29E-10
SPINT2	Mast cell	30.80%	6.20%	0.36	2.88E-10
RAB32	Mast cell	47.50%	34.50%	0.36	3.43E-10
OSM	Mast cell	20.80%	3.00%	0.45	3.65E-10

OSBPL8	Mast cell	70.80%	61.20%	0.36	5.19E-10
PPP2CA	Mast cell	58.30%	46.70%	0.36	5.59E-10
SERPINB1	Mast cell	57.50%	50.20%	0.40	7.46E-10
RGS10	Mast cell	70.00%	50.70%	0.45	7.67E-10
CREB3	Mast cell	45.00%	35.90%	0.29	1.08E-09
MALAT1	Mast cell	100.00%	100.00%	0.30	2.45E-09
TNFAIP8	Mast cell	45.00%	25.90%	0.61	4.05E-09
AMD1	Mast cell	80.80%	73.40%	0.40	4.32E-09
RAD21	Mast cell	43.30%	39.70%	0.28	4.52E-09
TMEM9B	Mast cell	53.30%	45.50%	0.30	5.36E-09
TDG	Mast cell	39.20%	29.10%	0.30	6.10E-09
SQSTM1	Mast cell	98.30%	92.40%	0.50	6.44E-09
EXOSC8	Mast cell	37.50%	25.80%	0.29	6.66E-09
HMGN1	Mast cell	72.50%	60.70%	0.38	6.75E-09
NFKBIB	Mast cell	33.30%	28.50%	0.25	7.17E-09
DDIT3	Mast cell	42.50%	29.40%	0.43	8.30E-09
IL2RG	Mast cell	19.20%	2.60%	0.28	9.04E-09
CD33	Mast cell	22.50%	3.40%	0.29	1.22E-08
CYLD	Mast cell	46.70%	32.40%	0.37	1.33E-08
ARL6IP1	Mast cell	70.80%	66.10%	0.37	1.37E-08
SPATA13	Mast cell	20.00%	6.70%	0.25	1.45E-08
STAG2	Mast cell	43.30%	38.20%	0.30	1.62E-08
ZEB2	Mast cell	66.70%	49.60%	0.41	1.86E-08
ACER3	Mast cell	25.00%	12.90%	0.30	1.90E-08
CD274	Mast cell	16.70%	3.40%	0.25	2.03E-08
ATP6V1G1	Mast cell	90.80%	87.40%	0.33	2.11E-08
AHR	Mast cell	57.50%	37.20%	0.49	2.36E-08
HNRNPM	Mast cell	72.50%	69.90%	0.33	2.96E-08
SH3KBP1	Mast cell	39.20%	34.00%	0.28	2.99E-08
CTSD	Mast cell	83.30%	80.40%	0.46	3.07E-08
PABPC1	Mast cell	98.30%	92.90%	0.36	3.11E-08
KDM6B	Mast cell	70.00%	55.60%	0.46	3.24E-08
YWHAZ	Mast cell	80.00%	74.20%	0.36	3.78E-08
BTN2A2	Mast cell	19.20%	4.90%	0.26	3.83E-08
RHOBTB3	Mast cell	33.30%	21.30%	0.33	4.02E-08
BEX4	Mast cell	33.30%	17.80%	0.35	4.08E-08
PDCD4	Mast cell	45.80%	33.30%	0.35	5.12E-08
RPS3	Mast cell	100.00%	99.40%	0.31	6.69E-08
GTF3C6	Mast cell	58.30%	50.30%	0.29	7.00E-08
SKIL	Mast cell	67.50%	52.80%	0.40	9.68E-08
ATP1B3	Mast cell	74.20%	70.30%	0.37	1.02E-07

CNRIP1	Mast cell	43.30%	37.40%	0.26	1.22E-07
NAP1L1	Mast cell	93.30%	87.80%	0.37	1.25E-07
CYCS	Mast cell	86.70%	81.70%	0.40	1.51E-07
EIF3E	Mast cell	92.50%	91.10%	0.34	1.51E-07
CHD2	Mast cell	50.00%	32.70%	0.39	2.11E-07
JUND	Mast cell	71.70%	59.50%	0.38	2.18E-07
PEPD	Mast cell	46.70%	42.70%	0.32	2.28E-07
ANP32E	Mast cell	57.50%	39.30%	0.36	2.31E-07
EMP3	Mast cell	100.00%	94.50%	0.31	2.60E-07
NXT1	Mast cell	52.50%	40.50%	0.33	2.90E-07
UQCRB	Mast cell	100.00%	95.60%	0.30	2.94E-07
TIPARP	Mast cell	70.00%	51.00%	0.49	3.11E-07
MRPL14	Mast cell	50.80%	43.80%	0.27	3.47E-07
ACTR3	Mast cell	73.30%	61.60%	0.37	3.83E-07
RAB8B	Mast cell	26.70%	19.60%	0.27	4.39E-07
STX3	Mast cell	27.50%	13.60%	0.32	5.30E-07
RPL36	Mast cell	100.00%	99.20%	0.29	6.58E-07
DCXR	Mast cell	35.80%	26.40%	0.27	7.84E-07
TM6SF1	Mast cell	19.20%	3.80%	0.33	8.50E-07
KMT2E	Mast cell	70.80%	59.10%	0.38	1.03E-06
PTPN6	Mast cell	24.20%	5.60%	0.28	1.19E-06
NFKBID	Mast cell	40.00%	15.50%	0.39	1.41E-06
SLC44A1	Mast cell	31.70%	23.40%	0.28	1.48E-06
FXYD5	Mast cell	96.70%	82.30%	0.33	1.58E-06
COX17	Mast cell	77.50%	67.20%	0.35	1.58E-06
IL18	Mast cell	24.20%	5.10%	0.35	1.78E-06
RPL23	Mast cell	99.20%	99.10%	0.27	2.26E-06
FAM96B	Mast cell	69.20%	66.10%	0.27	2.28E-06
ZFAS1	Mast cell	88.30%	90.30%	0.32	2.59E-06
LCP1	Mast cell	31.70%	9.00%	0.34	2.75E-06
RPS20	Mast cell	100.00%	99.80%	0.26	3.14E-06
RPL24	Mast cell	100.00%	99.30%	0.28	3.25E-06
RELB	Mast cell	25.80%	14.00%	0.27	4.00E-06
ARPP19	Mast cell	41.70%	36.20%	0.25	4.38E-06
STXBP3	Mast cell	39.20%	27.50%	0.28	4.64E-06
MT-ND2	Mast cell	100.00%	99.60%	0.26	4.80E-06
ARHGAP15	Mast cell	18.30%	3.30%	0.29	7.68E-06
RBBP8	Mast cell	25.00%	11.70%	0.26	1.24E-05
CALM2	Mast cell	98.30%	97.90%	0.33	1.65E-05
RPL22	Mast cell	100.00%	99.20%	0.27	1.76E-05
LEPROTL1	Mast cell	40.00%	27.00%	0.30	2.23E-05

NRIP3	Mast cell	20.80%	11.40%	0.26	2.24E-05
HCST	Mast cell	31.70%	10.70%	0.31	2.60E-05
EIF3D	Mast cell	70.80%	67.50%	0.25	2.96E-05
DENND4A	Mast cell	25.00%	9.00%	0.26	3.35E-05
AKNA	Mast cell	23.30%	7.80%	0.26	3.64E-05
PNPLA8	Mast cell	45.00%	36.80%	0.27	4.00E-05
ATP5G2	Mast cell	91.70%	88.80%	0.30	4.02E-05
CDC42	Mast cell	80.80%	78.00%	0.28	4.66E-05
BMP2K	Mast cell	27.50%	10.40%	0.31	4.86E-05
TAF9	Mast cell	62.50%	59.00%	0.26	8.83E-05
NR4A2	Mast cell	74.20%	55.40%	0.48	9.73E-05
MAST4	Mast cell	25.80%	9.80%	0.30	9.88E-05
H2AFZ	Mast cell	95.00%	91.10%	0.32	1.13E-04
LITAF	Mast cell	70.00%	61.10%	0.32	1.27E-04
LPCAT2	Mast cell	30.00%	16.80%	0.32	1.50E-04
IDI1	Mast cell	52.50%	45.10%	0.31	1.70E-04
CLK1	Mast cell	70.00%	59.90%	0.34	2.03E-04
ELL2	Mast cell	46.70%	38.40%	0.32	2.06E-04
BAZ1A	Mast cell	63.30%	56.90%	0.35	2.21E-04
OST4	Mast cell	97.50%	93.70%	0.31	2.39E-04
MT-CO2	Mast cell	99.20%	99.40%	0.28	2.57E-04
RBM39	Mast cell	90.00%	85.30%	0.29	2.94E-04
NR4A3	Mast cell	40.80%	24.90%	0.38	3.04E-04
SLC43A3	Mast cell	34.20%	26.20%	0.29	3.09E-04
SLC25A5	Mast cell	84.20%	80.90%	0.30	3.80E-04
CCL4	Mast cell	53.30%	34.60%	0.86	7.65E-04
COX7C	Mast cell	98.30%	96.20%	0.26	8.31E-04
DNAJA1	Mast cell	94.20%	90.70%	0.41	8.81E-04
PTTG1	Mast cell	41.70%	25.80%	0.32	9.14E-04
GPX1	Mast cell	89.20%	83.90%	0.39	9.19E-04
ITM2A	Mast cell	45.80%	23.30%	0.28	9.26E-04
C1orf162	Mast cell	26.70%	8.20%	0.31	1.16E-03
PPP1R15B	Mast cell	40.80%	29.60%	0.27	1.18E-03
RPS21	Mast cell	100.00%	98.60%	0.26	1.21E-03
CD84	Mast cell	21.70%	5.70%	0.28	1.69E-03
ABCA1	Mast cell	37.50%	18.00%	0.32	1.85E-03
THAP2	Mast cell	25.00%	18.20%	0.28	1.90E-03
EIF4E	Mast cell	57.50%	61.20%	0.31	2.15E-03
CAPZA1	Mast cell	46.70%	38.30%	0.26	2.51E-03
MED21	Mast cell	45.80%	32.00%	0.25	2.54E-03
BLOC1S2	Mast cell	56.70%	50.40%	0.25	2.69E-03

NFE2L2	Mast cell	87.50%	78.80%	0.36	3.15E-03
ATP6V0B	Mast cell	80.00%	76.10%	0.26	4.63E-03
EHD1	Mast cell	40.80%	36.30%	0.27	5.03E-03
USP15	Mast cell	42.50%	28.30%	0.26	1.52E-02
LIF	Mast cell	33.30%	14.00%	0.32	1.79E-02
NINJ1	Mast cell	53.30%	37.90%	0.35	2.90E-02
CKS2	Mast cell	57.50%	53.90%	0.30	4.14E-02
SLC3A2	Mast cell	83.30%	75.80%	0.35	4.56E-02
HNRNPK	Mast cell	92.50%	89.90%	0.25	5.61E-02
MYL12A	Mast cell	96.70%	90.90%	0.26	6.10E-02
TWISTNB	Mast cell	60.80%	47.30%	0.44	6.50E-02
MIR24-2	Mast cell	40.80%	31.40%	0.31	7.08E-02
NFKBIZ	Mast cell	67.50%	60.50%	0.28	7.63E-02
AP1S2	Mast cell	55.80%	40.00%	0.31	8.45E-02
SWAP70	Mast cell	41.70%	25.60%	0.26	3.91E-01
HSP90AB1	Mast cell	100.00%	98.50%	0.26	5.27E-01
TSPYL2	Mast cell	37.50%	26.90%	0.26	5.34E-01
MTHFD2	Mast cell	60.00%	48.10%	0.29	1.00E+00
KLF6	Mast cell	90.80%	80.20%	0.30	1.00E+00
LXN	Mast cell	30.80%	19.10%	0.25	1.00E+00
BTG2	Mast cell	65.80%	58.90%	0.32	1.00E+00
PHLDA2	Mast cell	65.80%	55.50%	0.31	1.00E+00
DUSP4	Mast cell	20.00%	16.80%	0.27	1.00E+00
TSC22D1	Mast cell	51.70%	53.70%	0.27	1.00E+00
HIST1H4C	ProIC	94.20%	43.10%	2.43	1.08E-177
PTTG1	ProIC	96.20%	25.60%	1.65	6.07E-119
CENPF	ProIC	96.20%	2.50%	1.84	8.95E-118
CDKN3	ProIC	88.50%	6.60%	1.45	5.62E-110
STMN1	ProIC	98.10%	16.40%	2.09	3.95E-107
UBE2C	ProIC	92.30%	0.80%	1.48	4.96E-102
MKI67	ProIC	94.20%	0.40%	1.22	1.77E-101
TOP2A	ProIC	94.20%	1.10%	1.51	7.59E-99
BIRC5	ProIC	92.30%	0.90%	1.30	1.83E-98
TPX2	ProIC	90.40%	0.50%	1.00	5.75E-95
NUSAP1	ProIC	94.20%	3.60%	1.43	3.55E-92
TK1	ProIC	88.50%	1.20%	1.36	9.99E-87
HMGN2	ProIC	100.00%	73.40%	1.43	1.41E-86
TUBB	ProIC	100.00%	83.20%	1.56	3.56E-86
PRC1	ProIC	90.40%	2.10%	1.20	3.42E-84
KIAA0101	ProIC	84.60%	1.80%	1.42	1.57E-83
RRM2	ProIC	76.90%	0.20%	1.01	4.38E-83

CDK1	ProIC	86.50%	1.40%	1.25	8.74E-79
TUBA1B	ProIC	100.00%	96.20%	1.64	2.32E-78
SMC4	ProIC	98.10%	25.80%	1.34	6.57E-76
CCNA2	ProIC	78.80%	0.40%	0.71	1.33E-75
ANLN	ProIC	76.90%	0.40%	0.74	1.80E-74
ASPM	ProIC	76.90%	0.60%	1.00	4.55E-74
GTSE1	ProIC	78.80%	1.00%	0.89	9.38E-74
FAM64A	ProIC	69.20%	0.20%	0.79	8.58E-72
CENPW	ProIC	84.60%	3.90%	1.13	1.12E-71
FOXM1	ProIC	76.90%	0.50%	0.65	2.00E-71
TYMS	ProIC	76.90%	1.10%	1.09	2.17E-70
KIF23	ProIC	78.80%	0.60%	0.61	1.66E-69
CENPM	ProIC	73.10%	0.30%	0.56	3.78E-69
CCNB2	ProIC	71.20%	0.40%	0.74	9.76E-69
CKAP2L	ProIC	73.10%	0.40%	0.62	3.12E-68
DIAPH3	ProIC	71.20%	0.40%	0.60	4.33E-68
DEPDC1	ProIC	65.40%	0.20%	0.55	6.95E-66
CEP55	ProIC	69.20%	0.30%	0.59	1.25E-65
PBK	ProIC	76.90%	1.50%	0.89	5.50E-65
CDCA5	ProIC	71.20%	0.50%	0.57	1.79E-64
SPC25	ProIC	61.50%	0.10%	0.59	4.47E-64
CDCA3	ProIC	69.20%	0.40%	0.66	8.38E-64
UBE2T	ProIC	76.90%	3.70%	0.94	1.31E-63
AURKB	ProIC	65.40%	0.20%	0.62	3.03E-63
CKS1B	ProIC	96.20%	47.50%	1.11	1.21E-62
CENPN	ProIC	88.50%	5.50%	0.92	1.25E-61
SHCBP1	ProIC	69.20%	0.50%	0.50	4.77E-61
SGOL1	ProIC	61.50%	0.20%	0.48	2.21E-60
HMMR	ProIC	65.40%	0.30%	0.62	4.31E-60
ZWINT	ProIC	80.80%	2.10%	0.76	6.36E-59
TMEM106C	ProIC	88.50%	31.00%	1.03	1.57E-58
CENPU	ProIC	78.80%	1.80%	0.88	7.79E-58
CASC5	ProIC	61.50%	0.30%	0.43	2.02E-57
TROAP	ProIC	59.60%	0.30%	0.47	1.99E-55
NCAPG	ProIC	57.70%	0.20%	0.44	7.30E-55
APOBEC3B	ProIC	61.50%	0.30%	0.57	1.25E-54
MELK	ProIC	61.50%	0.30%	0.43	1.69E-54
PKMYT1	ProIC	55.80%	0.10%	0.41	1.74E-54
HMGB2	ProIC	98.10%	46.90%	1.27	8.55E-54
DLGAP5	ProIC	50.00%	0.00%	0.40	9.64E-54
NUF2	ProIC	59.60%	0.40%	0.64	3.72E-53

RHEB	ProIC	96.20%	70.90%	1.04	2.53E-51
SKA3	ProIC	53.80%	0.20%	0.38	1.09E-49
RACGAP1	ProIC	80.80%	2.70%	0.58	5.82E-49
TUBA1C	ProIC	100.00%	67.50%	1.18	1.17E-48
ARHGAP11A	ProIC	63.50%	0.70%	0.49	1.71E-48
ESCO2	ProIC	65.40%	1.00%	0.51	3.15E-47
CDC20	ProIC	69.20%	1.90%	0.82	1.47E-46
CDKN2D	ProIC	82.70%	8.40%	0.87	4.56E-45
PRR11	ProIC	65.40%	1.30%	0.65	2.48E-44
KIF20A	ProIC	46.20%	0.10%	0.32	3.08E-44
ATAD2	ProIC	76.90%	5.10%	0.78	6.13E-44
LGALS1	ProIC	100.00%	97.30%	1.70	7.07E-44
PLK1	ProIC	51.90%	0.40%	0.54	7.90E-44
HMGB1	ProIC	100.00%	96.80%	1.06	3.04E-43
MXD3	ProIC	63.50%	1.70%	0.59	2.91E-42
POC1A	ProIC	59.60%	1.00%	0.35	1.66E-41
MAD2L1	ProIC	78.80%	6.30%	0.72	6.36E-41
TCF19	ProIC	69.20%	2.80%	0.61	8.50E-40
DTYMK	ProIC	86.50%	21.70%	0.88	1.17E-39
BUB1	ProIC	40.40%	0.10%	0.32	1.87E-39
KIF20B	ProIC	82.70%	6.20%	0.80	3.52E-39
HJURP	ProIC	40.40%	0.10%	0.30	5.06E-39
MYBL1	ProIC	59.60%	1.40%	0.38	5.21E-39
H2AFZ	ProIC	100.00%	91.10%	1.16	1.23E-38
CKAP2	ProIC	78.80%	5.80%	0.80	1.72E-38
MYBL2	ProIC	42.30%	0.10%	0.36	1.77E-38
HMGB3	ProIC	69.20%	4.30%	0.79	6.56E-38
NEK2	ProIC	42.30%	0.20%	0.34	9.70E-38
TACC3	ProIC	61.50%	1.70%	0.48	2.59E-37
PCNA	ProIC	75.00%	30.70%	0.87	5.20E-37
RPL39L	ProIC	78.80%	13.10%	0.80	6.41E-37
IQGAP3	ProIC	44.20%	0.30%	0.31	1.56E-36
C1QL1	ProIC	46.20%	0.40%	0.28	6.47E-36
COX8A	ProIC	100.00%	86.30%	0.85	1.18E-35
DEK	ProIC	98.10%	76.20%	0.94	1.98E-35
KIF2C	ProIC	42.30%	0.20%	0.29	6.04E-35
CENPH	ProIC	75.00%	4.50%	0.54	7.57E-35
CCNB1	ProIC	65.40%	4.20%	0.70	1.21E-34
LMNB2	ProIC	73.10%	4.10%	0.48	1.60E-34
CENPE	ProIC	67.30%	3.20%	0.62	5.00E-34
H2AFV	ProIC	100.00%	50.70%	0.85	5.41E-34

ASF1B	ProIC	57.70%	1.50%	0.48	9.33E-34
RAD51AP1	ProIC	51.90%	1.00%	0.35	1.50E-33
KIF11	ProIC	44.20%	0.40%	0.32	1.65E-33
DHFR	ProIC	67.30%	4.00%	0.67	3.16E-33
CDCA8	ProIC	40.40%	0.30%	0.27	6.16E-33
SKA2	ProIC	78.80%	16.50%	0.84	7.90E-33
CD70	ProIC	61.50%	17.00%	1.28	1.06E-32
PSMC3IP	ProIC	61.50%	3.40%	0.63	2.50E-32
KIF14	ProIC	46.20%	0.60%	0.35	3.10E-32
PHF19	ProIC	80.80%	9.80%	0.69	5.76E-32
GGH	ProIC	82.70%	15.30%	0.81	1.29E-31
CENPA	ProIC	40.40%	0.40%	0.38	1.49E-31
KIFC1	ProIC	46.20%	0.70%	0.26	1.57E-31
CLSPN	ProIC	55.80%	1.60%	0.50	1.63E-31
ZMYND10	ProIC	48.10%	1.10%	0.45	2.57E-31
MZT2B	ProIC	96.20%	58.10%	0.84	5.85E-31
PFN1	ProIC	100.00%	96.00%	0.88	1.22E-30
DNMT1	ProIC	92.30%	19.30%	0.74	1.70E-29
NUCKS1	ProIC	100.00%	86.70%	0.94	4.17E-29
GAPDH	ProIC	100.00%	99.30%	1.02	4.22E-29
CCDC34	ProIC	76.90%	11.50%	0.74	8.08E-29
KIF4A	ProIC	48.10%	1.20%	0.36	3.52E-28
ZNF367	ProIC	42.30%	0.60%	0.36	4.79E-28
FANCI	ProIC	46.20%	0.90%	0.34	7.90E-28
ECT2	ProIC	59.60%	3.00%	0.41	2.94E-27
RNASEH2A	ProIC	76.90%	8.30%	0.59	3.03E-27
TRIP13	ProIC	51.90%	1.90%	0.32	9.43E-27
MND1	ProIC	34.60%	0.20%	0.26	1.15E-26
CIT	ProIC	48.10%	1.40%	0.32	1.42E-26
USP1	ProIC	94.20%	33.30%	0.75	1.94E-26
CDCA2	ProIC	50.00%	1.70%	0.31	2.05E-26
NCAPH	ProIC	38.50%	0.40%	0.27	2.43E-26
ADAM12	ProIC	67.30%	8.10%	0.74	5.71E-26
NDC80	ProIC	46.20%	1.20%	0.40	1.17E-25
ORC6	ProIC	53.80%	2.50%	0.36	2.98E-25
DUT	ProIC	84.60%	63.50%	0.84	3.12E-25
SIVA1	ProIC	96.20%	69.00%	0.72	1.07E-23
MZT1	ProIC	84.60%	22.40%	0.71	1.33E-23
CDC45	ProIC	30.80%	0.20%	0.25	1.39E-23
KNSTRN	ProIC	50.00%	2.20%	0.47	3.21E-23
NCAPG2	ProIC	51.90%	2.50%	0.34	3.31E-23

TMPO	ProIC	71.20%	8.30%	0.59	4.39E-23
TUBB6	ProIC	96.20%	55.50%	0.78	5.63E-23
AURKA	ProIC	48.10%	1.90%	0.42	1.79E-22
EZH2	ProIC	78.80%	12.50%	0.59	2.05E-22
SGOL2	ProIC	55.80%	4.10%	0.53	2.25E-22
FAM111B	ProIC	34.60%	0.50%	0.43	2.38E-22
RANBP1	ProIC	92.30%	58.70%	0.70	4.29E-22
DRAP1	ProIC	92.30%	71.50%	0.73	8.02E-22
CENPK	ProIC	50.00%	2.40%	0.37	8.98E-22
DNAJC9	ProIC	80.80%	14.30%	0.60	1.39E-21
FEN1	ProIC	59.60%	5.80%	0.50	1.81E-21
STRA13	ProIC	90.40%	46.50%	0.71	3.86E-21
WHSC1	ProIC	53.80%	3.80%	0.33	1.64E-20
GPC1	ProIC	65.40%	7.20%	0.40	1.77E-20
ZWILCH	ProIC	61.50%	6.10%	0.35	1.97E-20
WDR34	ProIC	80.80%	22.60%	0.65	2.39E-20
RAD51	ProIC	34.60%	0.60%	0.26	2.62E-20
HN1	ProIC	86.50%	35.40%	0.72	3.49E-20
GAS2L3	ProIC	50.00%	3.30%	0.37	4.24E-20
GINS2	ProIC	38.50%	1.10%	0.38	5.10E-20
SMS	ProIC	92.30%	38.10%	0.78	1.07E-19
SMC2	ProIC	73.10%	14.50%	0.67	1.12E-19
NRM	ProIC	71.20%	10.70%	0.44	2.17E-19
LSM4	ProIC	94.20%	42.10%	0.68	4.87E-19
ANP32B	ProIC	94.20%	61.30%	0.73	6.88E-19
IL11	ProIC	57.70%	7.00%	0.84	1.18E-18
TIMELESS	ProIC	40.40%	1.50%	0.27	1.29E-18
CKS2	ProIC	98.10%	53.70%	0.82	1.54E-18
MAD2L2	ProIC	78.80%	27.50%	0.65	1.70E-18
HELLS	ProIC	55.80%	5.60%	0.52	2.82E-18
RAN	ProIC	98.10%	86.60%	0.68	3.21E-18
SPAG5	ProIC	42.30%	2.00%	0.25	3.25E-18
PTMS	ProIC	98.10%	52.00%	0.70	5.72E-18
MIS18BP1	ProIC	80.80%	18.40%	0.38	9.39E-18
TAGLN2	ProIC	100.00%	96.10%	0.67	1.60E-17
CENPB	ProIC	63.50%	9.20%	0.30	1.61E-17
CEP89	ProIC	61.50%	10.60%	0.25	2.06E-17
RHNO1	ProIC	53.80%	5.50%	0.29	2.86E-17
POLR3K	ProIC	71.20%	15.30%	0.27	4.03E-17
NT5DC2	ProIC	67.30%	12.40%	0.28	5.84E-17
MCM7	ProIC	65.40%	17.30%	0.56	6.69E-17

LIG1	ProIC	42.30%	2.50%	0.28	7.87E-17
MAZ	ProIC	88.50%	27.10%	0.58	9.00E-17
NCAPD3	ProIC	55.80%	6.70%	0.28	1.04E-16
PIN1	ProIC	92.30%	52.80%	0.62	1.54E-16
ITGB1BP1	ProIC	88.50%	59.00%	0.67	1.84E-16
IDH2	ProIC	78.80%	20.10%	0.55	3.57E-16
ANAPC11	ProIC	94.20%	75.40%	0.62	4.79E-16
TUBG1	ProIC	71.20%	13.90%	0.50	5.36E-16
FBXO5	ProIC	55.80%	6.20%	0.37	7.14E-16
TPGS2	ProIC	80.80%	24.60%	0.55	1.06E-15
DBI	ProIC	100.00%	83.80%	0.65	1.43E-15
LRR1	ProIC	53.80%	5.90%	0.31	1.59E-15
COL6A3	ProIC	82.70%	73.90%	0.82	3.67E-15
C19orf48	ProIC	65.40%	10.70%	0.39	4.05E-15
MCM4	ProIC	46.20%	4.00%	0.30	4.39E-15
SYNE2	ProIC	73.10%	16.90%	0.43	9.02E-15
NUDT1	ProIC	80.80%	29.70%	0.60	9.84E-15
BCL2L12	ProIC	71.20%	14.30%	0.44	1.54E-14
BRCA2	ProIC	42.30%	3.40%	0.26	2.23E-14
LMO7	ProIC	50.00%	5.30%	0.32	2.75E-14
XPO1	ProIC	75.00%	18.30%	0.34	2.96E-14
CSE1L	ProIC	61.50%	11.10%	0.30	4.13E-14
ANAPC15	ProIC	73.10%	19.00%	0.26	4.29E-14
SPDL1	ProIC	44.20%	3.70%	0.28	4.70E-14
REEP4	ProIC	50.00%	5.60%	0.29	4.79E-14
FAM101B	ProIC	55.80%	7.80%	0.30	4.93E-14
LOXL2	ProIC	75.00%	19.00%	0.72	5.91E-14
HMGN1	ProIC	96.20%	60.70%	0.64	6.28E-14
RP11-620J15.3	ProIC	59.60%	9.40%	0.32	6.73E-14
PRKCDBP	ProIC	92.30%	77.80%	0.77	1.67E-13
DSN1	ProIC	50.00%	6.30%	0.25	1.87E-13
BOLA3	ProIC	84.60%	34.40%	0.59	2.00E-13
SAE1	ProIC	73.10%	18.90%	0.52	2.09E-13
VANGL1	ProIC	51.90%	6.80%	0.27	3.42E-13
CLN6	ProIC	55.80%	8.10%	0.33	4.61E-13
CLIC1	ProIC	100.00%	94.80%	0.64	7.02E-13
CFL1	ProIC	100.00%	98.40%	0.56	7.62E-13
COMMD4	ProIC	90.40%	32.40%	0.45	1.86E-12
PLP2	ProIC	98.10%	79.40%	0.77	2.36E-12
EIF4EBP1	ProIC	78.80%	25.50%	0.49	2.40E-12
ACTN4	ProIC	86.50%	51.50%	0.65	3.20E-12

ANP32E	ProIC	90.40%	39.20%	0.59	3.26E-12
ATXN2L	ProIC	71.20%	19.00%	0.32	3.59E-12
HINT1	ProIC	98.10%	90.70%	0.55	3.69E-12
COL6A1	ProIC	84.60%	81.70%	0.80	3.88E-12
BCL7C	ProIC	76.90%	21.60%	0.45	4.19E-12
CD97	ProIC	84.60%	34.10%	0.59	4.44E-12
FAM83D	ProIC	38.50%	2.90%	0.26	4.60E-12
ACOT7	ProIC	73.10%	17.90%	0.48	5.34E-12
ITGA5	ProIC	80.80%	36.00%	0.68	6.03E-12
APITD1	ProIC	51.90%	7.30%	0.27	6.17E-12
HIST1H1E	ProIC	38.50%	2.90%	0.27	7.66E-12
CDC25B	ProIC	50.00%	7.40%	0.26	8.15E-12
TPI1	ProIC	98.10%	93.80%	0.59	1.06E-11
ZDHHC12	ProIC	69.20%	16.40%	0.36	1.08E-11
TPM4	ProIC	96.20%	80.60%	0.71	1.44E-11
CBX5	ProIC	88.50%	37.00%	0.55	1.51E-11
GAS2L1	ProIC	67.30%	15.60%	0.33	2.20E-11
RAD21	ProIC	90.40%	39.50%	0.55	2.26E-11
EMC9	ProIC	57.70%	11.50%	0.44	2.32E-11
ARPC2	ProIC	98.10%	91.70%	0.55	2.56E-11
PRRC2A	ProIC	75.00%	22.20%	0.30	2.72E-11
FADS1	ProIC	65.40%	14.70%	0.36	4.54E-11
NRP2	ProIC	82.70%	33.50%	0.67	4.56E-11
KIF5B	ProIC	94.20%	71.00%	0.59	4.66E-11
KIAA1524	ProIC	40.40%	3.90%	0.26	5.01E-11
NUDT15	ProIC	61.50%	13.80%	0.26	5.09E-11
TNFRSF12A	ProIC	94.20%	63.90%	0.91	6.82E-11
PDS5A	ProIC	75.00%	21.90%	0.32	7.05E-11
C11orf24	ProIC	73.10%	21.50%	0.31	7.71E-11
PMM1	ProIC	78.80%	25.10%	0.32	8.44E-11
PHGDH	ProIC	53.80%	9.60%	0.29	9.78E-11
ATAD5	ProIC	32.70%	2.00%	0.26	9.93E-11
RCC1	ProIC	67.30%	18.10%	0.27	1.07E-10
CCDC88A	ProIC	82.70%	28.60%	0.55	1.20E-10
CDCA4	ProIC	48.10%	6.80%	0.37	1.36E-10
SNRPG	ProIC	100.00%	80.30%	0.52	1.45E-10
HIST1H1A	ProIC	25.00%	1.20%	0.30	1.52E-10
LINC00152	ProIC	98.10%	48.00%	0.50	1.53E-10
CBFB	ProIC	63.50%	15.90%	0.26	2.18E-10
CKLF	ProIC	90.40%	36.80%	0.48	2.76E-10
IKBIP	ProIC	80.80%	42.50%	0.57	3.95E-10

RPA3	ProIC	86.50%	37.10%	0.49	3.99E-10
MZT2A	ProIC	80.80%	27.70%	0.47	4.10E-10
C12orf75	ProIC	63.50%	16.90%	0.65	4.61E-10
NAV2	ProIC	46.20%	6.40%	0.27	5.29E-10
BANF1	ProIC	90.40%	65.40%	0.55	5.40E-10
HDGF	ProIC	80.80%	28.20%	0.46	6.17E-10
COL5A1	ProIC	82.70%	39.00%	0.79	9.07E-10
SMC1A	ProIC	78.80%	26.40%	0.49	9.20E-10
SNRPB	ProIC	92.30%	76.10%	0.53	1.36E-09
COX20	ProIC	90.40%	50.60%	0.55	1.43E-09
SLAIN2	ProIC	76.90%	26.80%	0.27	1.93E-09
COL15A1	ProIC	76.90%	24.90%	0.65	2.47E-09
HP1BP3	ProIC	98.10%	61.80%	0.51	2.50E-09
BARD1	ProIC	40.40%	4.80%	0.26	3.30E-09
NCAPH2	ProIC	65.40%	17.00%	0.32	3.35E-09
CALM2	ProIC	100.00%	97.90%	0.59	3.55E-09
DCK	ProIC	57.70%	12.70%	0.31	3.88E-09
PSIP1	ProIC	65.40%	18.20%	0.28	4.48E-09
MNS1	ProIC	42.30%	5.30%	0.29	5.06E-09
MAP1B	ProIC	73.10%	27.60%	0.81	5.14E-09
CSPG4	ProIC	73.10%	22.60%	0.49	5.44E-09
RRM1	ProIC	63.50%	15.80%	0.42	5.47E-09
PGAM1	ProIC	98.10%	72.80%	0.61	6.41E-09
PTMA	ProIC	100.00%	99.90%	0.50	8.72E-09
RASSF1	ProIC	78.80%	29.60%	0.47	8.78E-09
COL5A3	ProIC	38.50%	6.10%	0.50	8.79E-09
NRAS	ProIC	61.50%	16.10%	0.26	1.00E-08
COL5A2	ProIC	82.70%	52.80%	0.78	1.14E-08
NDUFAF3	ProIC	94.20%	65.40%	0.51	1.33E-08
PPIA	ProIC	100.00%	98.00%	0.46	1.55E-08
COPS3	ProIC	84.60%	34.80%	0.28	1.78E-08
AP2S1	ProIC	96.20%	67.70%	0.53	1.85E-08
MIIP	ProIC	61.50%	15.90%	0.29	2.05E-08
ENO1	ProIC	100.00%	95.60%	0.61	2.08E-08
CDK5RAP2	ProIC	57.70%	14.00%	0.28	2.55E-08
COL1A1	ProIC	88.50%	69.10%	1.35	2.78E-08
AURKAIP1	ProIC	92.30%	58.00%	0.49	2.95E-08
CARHSP1	ProIC	82.70%	42.00%	0.57	3.24E-08
SLC25A5	ProIC	98.10%	80.80%	0.54	3.52E-08
RNF26	ProIC	53.80%	11.20%	0.31	6.34E-08
DCXR	ProIC	75.00%	26.20%	0.44	6.94E-08

KIF22	ProIC	78.80%	31.50%	0.47	8.38E-08
WSB2	ProIC	59.60%	15.30%	0.28	8.59E-08
DAG1	ProIC	69.20%	21.60%	0.31	1.29E-07
SUN2	ProIC	78.80%	29.20%	0.35	1.39E-07
ICMT	ProIC	57.70%	14.60%	0.28	1.42E-07
ITGB3BP	ProIC	65.40%	20.40%	0.26	1.49E-07
PARP1	ProIC	73.10%	29.30%	0.37	1.57E-07
RUVBL2	ProIC	75.00%	27.90%	0.42	1.74E-07
H2AFY	ProIC	88.50%	44.30%	0.47	1.76E-07
RPSA	ProIC	100.00%	97.70%	0.48	1.77E-07
MCM5	ProIC	42.30%	7.90%	0.41	1.81E-07
ST3GAL4	ProIC	61.50%	17.50%	0.31	1.99E-07
POLD2	ProIC	86.50%	41.00%	0.46	2.02E-07
DDX39A	ProIC	76.90%	32.50%	0.44	2.12E-07
GNB1	ProIC	90.40%	43.60%	0.43	2.27E-07
DIAPH1	ProIC	82.70%	33.40%	0.35	2.71E-07
AKT1	ProIC	59.60%	16.00%	0.33	2.80E-07
LSM5	ProIC	94.20%	55.40%	0.45	2.80E-07
NAP1L1	ProIC	98.10%	87.80%	0.48	2.87E-07
CEBPG	ProIC	78.80%	30.40%	0.33	3.26E-07
TMEM18	ProIC	75.00%	27.40%	0.30	3.37E-07
CBX1	ProIC	80.80%	31.70%	0.38	3.94E-07
TPM3	ProIC	98.10%	72.50%	0.54	4.12E-07
MYH9	ProIC	88.50%	43.50%	0.46	4.19E-07
TOR1A	ProIC	71.20%	25.30%	0.26	4.23E-07
CORO1C	ProIC	76.90%	29.30%	0.34	4.46E-07
VIM	ProIC	100.00%	100.00%	0.55	4.64E-07
FAM111A	ProIC	48.10%	10.90%	0.40	4.97E-07
RUSC1	ProIC	59.60%	16.90%	0.28	5.33E-07
RFC2	ProIC	53.80%	12.50%	0.29	6.61E-07
GMNN	ProIC	55.80%	15.40%	0.42	7.49E-07
C9orf142	ProIC	76.90%	28.80%	0.37	7.50E-07
BAZ1B	ProIC	71.20%	26.90%	0.25	8.12E-07
PTP4A2	ProIC	92.30%	46.20%	0.31	8.60E-07
NUCB2	ProIC	92.30%	69.00%	0.59	8.69E-07
MRPL37	ProIC	75.00%	27.20%	0.32	8.75E-07
EXOSC8	ProIC	73.10%	25.70%	0.33	9.78E-07
CCP110	ProIC	44.20%	8.30%	0.27	9.82E-07
HINT2	ProIC	88.50%	42.30%	0.39	1.14E-06
NIPA2	ProIC	71.20%	24.60%	0.31	1.19E-06
GPSM2	ProIC	63.50%	20.40%	0.25	1.27E-06

RAD23A	ProIC	90.40%	67.10%	0.48	1.33E-06
GAMT	ProIC	57.70%	15.50%	0.32	1.38E-06
SPARC	ProIC	92.30%	83.20%	0.84	1.51E-06
UCP2	ProIC	57.70%	17.20%	0.35	1.61E-06
UBE2S	ProIC	84.60%	40.20%	0.53	1.66E-06
UBALD2	ProIC	53.80%	14.60%	0.38	1.99E-06
LSM2	ProIC	84.60%	39.60%	0.31	2.18E-06
PPDPF	ProIC	96.20%	91.10%	0.49	2.36E-06
BUB3	ProIC	82.70%	40.70%	0.45	2.84E-06
CLEC11A	ProIC	59.60%	19.40%	0.45	2.91E-06
FHL2	ProIC	71.20%	33.50%	0.63	3.28E-06
PPP1CA	ProIC	94.20%	56.90%	0.43	3.29E-06
HMGXB4	ProIC	69.20%	25.30%	0.26	3.54E-06
TEX30	ProIC	51.90%	12.10%	0.29	3.73E-06
YWHAZ	ProIC	100.00%	74.10%	0.43	3.75E-06
RHOC	ProIC	88.50%	80.10%	0.50	4.74E-06
COL4A1	ProIC	63.50%	30.40%	0.86	5.93E-06
KHDRBS1	ProIC	76.90%	31.10%	0.35	6.83E-06
NUDCD2	ProIC	80.80%	36.10%	0.30	7.24E-06
FLNA	ProIC	94.20%	57.70%	0.49	8.36E-06
SNRPD1	ProIC	88.50%	56.70%	0.46	9.18E-06
CKAP4	ProIC	75.00%	31.80%	0.29	9.61E-06
ARL6IP4	ProIC	98.10%	79.80%	0.45	9.66E-06
AGPAT2	ProIC	76.90%	31.40%	0.31	9.84E-06
BDP1	ProIC	82.70%	37.90%	0.27	1.02E-05
ACTN1	ProIC	86.50%	43.10%	0.42	1.06E-05
RABL5	ProIC	73.10%	28.50%	0.29	1.06E-05
SGCB	ProIC	80.80%	42.90%	0.48	1.12E-05
CHTOP	ProIC	73.10%	29.60%	0.25	1.24E-05
SPATS2L	ProIC	75.00%	29.60%	0.34	1.25E-05
TFPI2	ProIC	21.20%	1.30%	0.47	1.29E-05
SNF8	ProIC	82.70%	37.50%	0.33	1.35E-05
CAPZA1	ProIC	82.70%	38.10%	0.31	1.49E-05
SZRD1	ProIC	78.80%	35.10%	0.28	1.54E-05
G3BP1	ProIC	84.60%	44.60%	0.28	1.60E-05
CALM3	ProIC	86.50%	49.10%	0.44	1.81E-05
MSC	ProIC	61.50%	20.00%	0.47	1.90E-05
SLC52A2	ProIC	76.90%	32.90%	0.26	2.06E-05
BTG3	ProIC	94.20%	54.40%	0.42	2.12E-05
MAP4K4	ProIC	57.70%	16.80%	0.33	2.44E-05
MRPL51	ProIC	92.30%	75.50%	0.43	2.56E-05

ELP5	ProIC	69.20%	26.80%	0.26	2.59E-05
MSN	ProIC	96.20%	69.80%	0.47	2.94E-05
TGFBI	ProIC	92.30%	64.90%	0.72	3.09E-05
LOX	ProIC	48.10%	13.50%	0.36	3.13E-05
RBM17	ProIC	84.60%	40.90%	0.29	3.60E-05
PRADC1	ProIC	67.30%	24.50%	0.28	3.86E-05
CSF1	ProIC	61.50%	28.90%	0.51	3.95E-05
PRKDC	ProIC	76.90%	33.70%	0.26	4.28E-05
COL3A1	ProIC	92.30%	75.00%	0.84	4.74E-05
ILF3	ProIC	88.50%	45.60%	0.28	5.33E-05
CALU	ProIC	92.30%	76.10%	0.48	5.41E-05
UBA2	ProIC	82.70%	40.10%	0.25	5.48E-05
COL4A2	ProIC	67.30%	34.30%	0.66	5.76E-05
GNG5	ProIC	100.00%	81.90%	0.36	5.85E-05
ARPC5	ProIC	98.10%	74.40%	0.40	6.34E-05
SSRP1	ProIC	78.80%	35.50%	0.33	6.43E-05
KPNA2	ProIC	92.30%	52.40%	0.34	6.56E-05
PALLD	ProIC	71.20%	28.00%	0.38	6.59E-05
TUBB4B	ProIC	100.00%	89.50%	0.56	7.55E-05
ARL6IP1	ProIC	82.70%	66.10%	0.49	7.81E-05
COX5A	ProIC	96.20%	66.60%	0.39	7.84E-05
CKAP5	ProIC	44.20%	9.80%	0.26	8.00E-05
CD82	ProIC	73.10%	34.50%	0.54	9.78E-05
RPL26L1	ProIC	76.90%	34.80%	0.26	9.79E-05
EHD2	ProIC	75.00%	31.90%	0.31	1.11E-04
WBP11	ProIC	80.80%	37.30%	0.31	1.12E-04
SMC3	ProIC	73.10%	38.70%	0.43	1.13E-04
FAM96A	ProIC	65.40%	25.20%	0.26	1.36E-04
MAPKAPK2	ProIC	71.20%	29.20%	0.27	1.60E-04
PSME2	ProIC	96.20%	58.80%	0.34	1.62E-04
NDUFS6	ProIC	98.10%	71.10%	0.38	1.95E-04
CMC2	ProIC	71.20%	30.20%	0.39	2.09E-04
ANKRD11	ProIC	84.60%	43.20%	0.29	2.29E-04
ITGB1	ProIC	94.20%	86.90%	0.45	2.33E-04
S100A16	ProIC	65.40%	47.50%	0.51	2.74E-04
CALM1	ProIC	100.00%	96.80%	0.44	2.79E-04
ACTR2	ProIC	90.40%	51.20%	0.26	2.83E-04
PTN	ProIC	59.60%	20.30%	0.46	2.91E-04
SUMO3	ProIC	94.20%	61.00%	0.39	3.08E-04
COL18A1	ProIC	46.20%	26.60%	0.58	3.22E-04
DCTN3	ProIC	84.60%	63.70%	0.42	3.33E-04

LRRC59	ProIC	82.70%	53.50%	0.45	3.47E-04
PFKL	ProIC	78.80%	36.90%	0.29	4.04E-04
SNRPF	ProIC	98.10%	69.20%	0.36	4.15E-04
MRPL27	ProIC	82.70%	43.80%	0.39	4.20E-04
CRNDE	ProIC	59.60%	21.50%	0.26	4.37E-04
PLOD1	ProIC	75.00%	33.90%	0.26	4.59E-04
CACYBP	ProIC	82.70%	50.10%	0.40	4.71E-04
TXNDC17	ProIC	90.40%	51.00%	0.34	4.80E-04
YWHAQ	ProIC	100.00%	84.40%	0.31	4.84E-04
ADAM15	ProIC	71.20%	30.90%	0.27	4.97E-04
EMD	ProIC	71.20%	31.00%	0.27	5.34E-04
MEA1	ProIC	76.90%	39.30%	0.38	5.87E-04
LMO4	ProIC	82.70%	53.60%	0.48	5.99E-04
PLEC	ProIC	84.60%	44.00%	0.31	6.08E-04
DOK5	ProIC	40.40%	9.90%	0.29	6.47E-04
C1QTNF2	ProIC	71.20%	29.90%	0.35	6.99E-04
RALY	ProIC	84.60%	45.40%	0.33	8.46E-04
MYO1C	ProIC	73.10%	32.20%	0.36	8.62E-04
ARPP19	ProIC	76.90%	36.10%	0.32	8.71E-04
FLOT1	ProIC	96.20%	61.70%	0.32	9.76E-04
SMTN	ProIC	61.50%	23.60%	0.37	1.01E-03
RAC1	ProIC	92.30%	56.00%	0.34	1.10E-03
CEP350	ProIC	63.50%	25.00%	0.26	1.18E-03
PPM1G	ProIC	78.80%	39.20%	0.27	1.38E-03
EIF4E2	ProIC	76.90%	36.40%	0.27	1.49E-03
MRPL18	ProIC	86.50%	49.30%	0.26	1.62E-03
PRDX3	ProIC	88.50%	50.10%	0.26	1.63E-03
SYNE1	ProIC	75.00%	35.00%	0.37	1.66E-03
HMG20B	ProIC	75.00%	38.20%	0.38	2.14E-03
FIBP	ProIC	82.70%	44.00%	0.31	2.15E-03
PXDN	ProIC	65.40%	26.20%	0.31	2.24E-03
BIRC3	ProIC	61.50%	23.20%	0.47	2.28E-03
EZR	ProIC	94.20%	59.90%	0.38	2.41E-03
MCM3	ProIC	44.20%	12.40%	0.32	2.42E-03
NAP1L4	ProIC	73.10%	33.50%	0.26	2.42E-03
MIR4435-1HG	ProIC	82.70%	43.10%	0.38	3.09E-03
RPS26	ProIC	100.00%	93.70%	0.36	3.34E-03
MRPL52	ProIC	88.50%	50.90%	0.26	3.35E-03
H3F3A	ProIC	100.00%	98.30%	0.31	4.07E-03
ARPC1B	ProIC	98.10%	86.50%	0.41	4.15E-03
LMNA	ProIC	100.00%	98.60%	0.42	4.32E-03

RAB32	ProIC	73.10%	34.50%	0.26	4.44E-03
ADAMTS2	ProIC	63.50%	26.90%	0.30	4.49E-03
NAA10	ProIC	82.70%	43.50%	0.29	4.51E-03
UQCR10	ProIC	94.20%	61.90%	0.33	4.54E-03
PTS	ProIC	80.80%	41.30%	0.31	4.55E-03
RBX1	ProIC	98.10%	80.80%	0.33	4.66E-03
CBX3	ProIC	88.50%	55.50%	0.39	5.27E-03
DGKI	ProIC	34.60%	7.00%	0.33	5.92E-03
SCCPDH	ProIC	69.20%	30.40%	0.30	6.39E-03
CKB	ProIC	59.60%	25.80%	0.51	7.04E-03
MRPL34	ProIC	84.60%	47.00%	0.27	7.07E-03
LMF2	ProIC	78.80%	40.90%	0.26	7.23E-03
KPNB1	ProIC	88.50%	51.70%	0.31	7.44E-03
RAB13	ProIC	94.20%	63.20%	0.35	9.52E-03
ARL4A	ProIC	84.60%	47.30%	0.38	1.01E-02
MYL6	ProIC	100.00%	99.10%	0.32	1.07E-02
CMIP	ProIC	57.70%	21.60%	0.28	1.15E-02
LIF	ProIC	46.20%	14.00%	0.53	1.21E-02
RAB31	ProIC	88.50%	52.80%	0.34	1.23E-02
RPLP1	ProIC	100.00%	99.80%	0.29	1.23E-02
PCBP1	ProIC	96.20%	65.70%	0.28	1.26E-02
PMVK	ProIC	86.50%	50.00%	0.26	1.32E-02
CDH13	ProIC	65.40%	29.40%	0.26	1.34E-02
LSM3	ProIC	92.30%	62.30%	0.35	1.40E-02
CRIP2	ProIC	96.20%	75.10%	0.43	1.46E-02
ILVBL	ProIC	55.80%	22.00%	0.27	1.75E-02
MTCH2	ProIC	69.20%	32.50%	0.31	1.76E-02
TMSB10	ProIC	98.10%	98.00%	0.42	2.06E-02
S100A3	ProIC	59.60%	24.70%	0.25	2.11E-02
HMGA1	ProIC	51.90%	22.00%	0.38	2.16E-02
FBXW5	ProIC	71.20%	34.00%	0.25	2.27E-02
CTNNAL1	ProIC	80.80%	45.10%	0.37	2.53E-02
CTSZ	ProIC	80.80%	45.40%	0.54	2.60E-02
PDCD5	ProIC	94.20%	66.00%	0.33	2.67E-02
CAPG	ProIC	82.70%	63.30%	0.55	2.74E-02
RRAD	ProIC	34.60%	9.90%	0.37	2.89E-02
STOML2	ProIC	84.60%	50.20%	0.25	3.37E-02
MLF2	ProIC	92.30%	62.50%	0.31	3.38E-02
CD44	ProIC	100.00%	90.20%	0.37	3.84E-02
HNRNPAB	ProIC	73.10%	36.30%	0.27	4.05E-02
SLC16A3	ProIC	65.40%	31.70%	0.47	4.22E-02

ARHGDIB	ProIC	51.90%	21.40%	0.33	4.62E-02
PSMD7	ProIC	88.50%	54.80%	0.26	5.05E-02
DYNLT1	ProIC	80.80%	53.70%	0.38	5.64E-02
PSMC3	ProIC	88.50%	61.90%	0.35	6.08E-02
COX7A2	ProIC	98.10%	93.00%	0.34	6.36E-02
KLF6	ProIC	92.30%	80.30%	0.59	6.41E-02
TIMM10	ProIC	73.10%	38.90%	0.34	6.66E-02
GSTO1	ProIC	96.20%	70.10%	0.31	6.69E-02
CLIC4	ProIC	84.60%	53.70%	0.33	7.28E-02
KIAA0040	ProIC	61.50%	27.40%	0.26	8.05E-02
NDUFS8	ProIC	90.40%	59.50%	0.32	9.47E-02
TPM2	ProIC	80.80%	45.30%	0.31	9.49E-02
CA5B	ProIC	57.70%	24.00%	0.25	9.83E-02
SPAG9	ProIC	92.30%	61.50%	0.25	1.03E-01
DESI2	ProIC	65.40%	30.80%	0.27	1.11E-01
CNIH4	ProIC	80.80%	49.80%	0.35	1.12E-01
GTF3A	ProIC	90.40%	59.00%	0.29	1.20E-01
DGCR6L	ProIC	71.20%	35.70%	0.28	1.29E-01
ABL2	ProIC	90.40%	66.20%	0.33	1.41E-01
IGFBP3	ProIC	21.20%	6.20%	0.51	1.42E-01
ANKRD28	ProIC	90.40%	58.90%	0.34	1.58E-01
RFC1	ProIC	67.30%	33.30%	0.29	1.70E-01
VPS29	ProIC	92.30%	62.70%	0.29	1.79E-01
FDPS	ProIC	78.80%	44.10%	0.26	1.83E-01
CD59	ProIC	94.20%	82.20%	0.45	2.04E-01
GPAA1	ProIC	90.40%	59.80%	0.29	2.21E-01
BAX	ProIC	67.30%	42.00%	0.26	2.23E-01
RBBP7	ProIC	78.80%	45.20%	0.31	2.39E-01
TMCO3	ProIC	75.00%	42.90%	0.32	2.97E-01
ANXA5	ProIC	100.00%	94.80%	0.35	3.43E-01
LRRFIP1	ProIC	92.30%	64.40%	0.33	3.46E-01
TGFB1I1	ProIC	69.20%	38.20%	0.30	3.72E-01
PHLDA2	ProIC	86.50%	55.50%	0.36	3.77E-01
PCBP2	ProIC	96.20%	83.70%	0.30	3.83E-01
ZFP91	ProIC	55.80%	24.10%	0.26	3.92E-01
TP53I13	ProIC	63.50%	30.30%	0.28	3.92E-01
CCND1	ProIC	71.20%	48.70%	0.52	4.17E-01
MRPL23	ProIC	88.50%	57.80%	0.28	4.39E-01
SUMO2	ProIC	100.00%	92.40%	0.29	5.05E-01
TRAPPC1	ProIC	76.90%	47.00%	0.26	5.22E-01
ECM1	ProIC	88.50%	57.70%	0.25	5.27E-01

RBP4	ProIC	42.30%	14.30%	0.32	5.52E-01
PTTG1IP	ProIC	88.50%	61.90%	0.29	7.34E-01
C19orf43	ProIC	92.30%	76.50%	0.33	7.94E-01
MPC2	ProIC	88.50%	63.40%	0.30	8.69E-01
GNAI2	ProIC	86.50%	56.10%	0.25	8.85E-01
ISLR	ProIC	75.00%	72.10%	0.46	9.09E-01
PA2G4	ProIC	92.30%	67.70%	0.30	1.00E+00
ATP5J2	ProIC	96.20%	83.70%	0.28	1.00E+00
ISOC2	ProIC	69.20%	39.80%	0.32	1.00E+00
TCEB2	ProIC	98.10%	91.00%	0.28	1.00E+00
MRPL41	ProIC	82.70%	57.80%	0.33	1.00E+00
CCDC85B	ProIC	88.50%	69.40%	0.35	1.00E+00
HSPB11	ProIC	73.10%	42.10%	0.26	1.00E+00
S100A10	ProIC	100.00%	99.30%	0.42	1.00E+00
MRP63	ProIC	82.70%	53.50%	0.28	1.00E+00
PHPT1	ProIC	92.30%	72.90%	0.31	1.00E+00
ANPEP	ProIC	69.20%	38.30%	0.30	1.00E+00
RPL28	ProIC	100.00%	99.70%	0.28	1.00E+00
PLAUR	ProIC	80.80%	60.00%	0.36	1.00E+00
UQCC2	ProIC	73.10%	42.90%	0.27	1.00E+00
NQO2	ProIC	75.00%	44.40%	0.25	1.00E+00
ACTA2	ProIC	46.20%	18.70%	0.28	1.00E+00
FAM180A	ProIC	63.50%	33.10%	0.31	1.00E+00
KDM6B	ProIC	76.90%	55.60%	0.27	1.00E+00
UQCRH	ProIC	98.10%	84.00%	0.25	1.00E+00
COTL1	ProIC	42.30%	17.10%	0.29	1.00E+00
RBMX	ProIC	90.40%	65.80%	0.27	1.00E+00
DKK3	ProIC	82.70%	56.00%	0.32	1.00E+00
CTSB	ProIC	98.10%	81.20%	0.32	1.00E+00
C12orf57	ProIC	94.20%	83.40%	0.32	1.00E+00
H2AFJ	ProIC	90.40%	68.10%	0.29	1.00E+00
TM4SF1	ProIC	78.80%	50.80%	0.32	1.00E+00
MIF	ProIC	92.30%	71.00%	0.29	1.00E+00
UBE2D2	ProIC	88.50%	63.90%	0.27	1.00E+00
ARHGDIA	ProIC	90.40%	66.80%	0.27	1.00E+00
NDUFA4L2	ProIC	88.50%	66.20%	0.49	1.00E+00
COL6A2	ProIC	88.50%	86.30%	0.37	1.00E+00
CSTB	ProIC	90.40%	82.20%	0.39	1.00E+00
UQCRFS1	ProIC	80.80%	54.10%	0.26	1.00E+00
PTHLH	ProIC	46.20%	20.80%	0.25	1.00E+00
VKORC1	ProIC	98.10%	82.40%	0.28	1.00E+00

ELN	ProIC	63.50%	36.70%	0.35	1.00E+00
COL1A2	ProIC	90.40%	87.00%	0.53	1.00E+00
RCAN1	ProIC	76.90%	52.20%	0.38	1.00E+00
NOTCH3	ProIC	25.00%	7.90%	0.30	1.00E+00
CDA	ProIC	48.10%	24.20%	0.26	1.00E+00
ACTB	ProIC	100.00%	99.90%	0.25	1.00E+00
MFGE8	ProIC	84.60%	67.40%	0.30	1.00E+00
HAS2	ProIC	26.90%	11.00%	0.28	1.00E+00
RGS5	ProIC	25.00%	7.70%	0.68	1.00E+00
CIB1	ProIC	90.40%	73.20%	0.26	1.00E+00
KCNMA1	ProIC	57.70%	34.00%	0.34	1.00E+00
CXCL2	ProIC	82.70%	59.90%	0.39	1.00E+00
NT5E	ProIC	71.20%	47.40%	0.26	1.00E+00
MT2A	ProIC	100.00%	95.20%	0.40	1.00E+00
SEP7	ProIC	90.40%	83.10%	0.28	1.00E+00
PRG4	SIF	99.70%	92.40%	2.00	0.00E+00
CSN1S1	SIF	15.50%	10.00%	1.95	0.00E+00
INHBA	SIF	82.50%	41.40%	1.76	0.00E+00
HTRA1	SIF	97.30%	76.40%	1.76	0.00E+00
CLU	SIF	99.80%	91.60%	1.67	0.00E+00
DEFB1	SIF	82.90%	20.30%	1.55	0.00E+00
CRTAC1	SIF	99.60%	82.20%	1.40	0.00E+00
CD55	SIF	94.60%	73.90%	1.39	0.00E+00
FN1	SIF	99.90%	94.50%	1.38	0.00E+00
TIMP3	SIF	96.50%	72.50%	1.37	0.00E+00
TNFAIP6	SIF	91.30%	69.70%	1.27	0.00E+00
ERRFI1	SIF	95.10%	65.70%	1.14	0.00E+00
TWISTNB	SIF	69.00%	38.20%	1.14	0.00E+00
HBEGF	SIF	85.40%	42.20%	1.11	0.00E+00
GFPT2	SIF	87.90%	46.70%	1.07	0.00E+00
MT2A	SIF	99.80%	93.30%	1.04	0.00E+00
AK1	SIF	97.10%	67.90%	0.99	0.00E+00
IGFBP5	SIF	76.90%	40.20%	0.94	0.00E+00
C2orf40	SIF	90.10%	52.20%	0.91	0.00E+00
MT1X	SIF	93.80%	72.40%	0.87	0.00E+00
NDUFA4L2	SIF	89.00%	56.50%	0.87	0.00E+00
PROCR	SIF	82.50%	43.40%	0.85	0.00E+00
PCOLCE2	SIF	95.60%	58.70%	0.80	0.00E+00
HAS1	SIF	87.00%	54.40%	0.78	0.00E+00
SLC7A2	SIF	42.10%	12.00%	0.78	0.00E+00
SMIM14	SIF	92.10%	61.90%	0.77	0.00E+00

CRLF1	SIF	71.30%	38.90%	0.77	0.00E+00
VKORC1	SIF	94.30%	77.40%	0.75	0.00E+00
CDO1	SIF	85.70%	56.50%	0.73	0.00E+00
PTGES	SIF	55.90%	27.40%	0.72	0.00E+00
GPX3	SIF	96.10%	72.90%	0.71	0.00E+00
SLC39A14	SIF	82.00%	46.40%	0.71	0.00E+00
UGP2	SIF	92.90%	68.40%	0.68	0.00E+00
HHIP	SIF	39.50%	3.70%	0.68	0.00E+00
TMEM196	SIF	56.00%	18.90%	0.67	0.00E+00
ITGB8	SIF	64.50%	19.70%	0.66	0.00E+00
CAPS	SIF	74.60%	25.50%	0.65	0.00E+00
UAP1	SIF	93.60%	69.20%	0.64	0.00E+00
HSD3B7	SIF	83.20%	44.10%	0.58	0.00E+00
ARL2	SIF	91.60%	64.90%	0.57	0.00E+00
PLEKHA1	SIF	74.50%	36.70%	0.57	0.00E+00
SH3BGRL3	SIF	98.40%	92.90%	0.56	0.00E+00
MT1F	SIF	54.70%	19.10%	0.56	0.00E+00
C10orf105	SIF	55.60%	11.00%	0.54	0.00E+00
F5	SIF	58.80%	19.10%	0.53	0.00E+00
CD151	SIF	96.50%	80.30%	0.52	0.00E+00
CD164	SIF	84.80%	71.40%	0.52	0.00E+00
SOD1	SIF	98.20%	92.80%	0.49	0.00E+00
ALDOA	SIF	98.30%	94.30%	0.47	0.00E+00
LDHA	SIF	99.30%	95.00%	0.46	0.00E+00
BTC	SIF	48.10%	10.10%	0.45	0.00E+00
SEMA3A	SIF	32.30%	4.00%	0.43	0.00E+00
PVRL4	SIF	31.50%	3.60%	0.42	0.00E+00
SLC2A12	SIF	43.80%	8.70%	0.40	0.00E+00
GPR64	SIF	43.70%	10.10%	0.39	0.00E+00
HHIP-AS1	SIF	29.30%	2.50%	0.31	0.00E+00
RCAN1	SIF	73.30%	43.20%	0.75	3.07241175005999e-313
BCAT1	SIF	76.30%	39.50%	0.46	9.17E-307
SOX5	SIF	58.90%	21.90%	0.41	2.20E-306
DLX4	SIF	54.50%	18.30%	0.45	1.67E-305
SLC29A1	SIF	68.20%	30.90%	0.49	5.24E-302
PRELP	SIF	88.40%	72.10%	0.95	8.72E-302
MGP	SIF	99.90%	91.50%	0.36	5.83E-300
TPD52L1	SIF	67.10%	30.90%	0.51	4.77E-298
ADAMTS6	SIF	41.50%	9.50%	0.41	3.15E-295
SEMA3E	SIF	49.20%	14.90%	0.43	2.62E-294
ZNF385B	SIF	44.00%	12.50%	0.42	4.49E-294

RP11-680F20.6	SIF	36.80%	7.50%	0.38	1.80E-293
LUM	SIF	99.90%	89.60%	0.26	2.49E-291
RGCC	SIF	77.80%	45.60%	0.58	5.39E-290
PLP2	SIF	93.20%	73.60%	0.51	1.06E-287
ITPR3	SIF	44.60%	12.50%	0.39	3.44E-287
SEMA3C	SIF	73.80%	38.00%	0.48	1.27E-286
CD9	SIF	99.50%	94.00%	0.56	5.18E-284
SELM	SIF	96.90%	84.00%	0.40	1.03E-282
SORBS2	SIF	60.60%	24.20%	0.43	2.24E-282
GABRA4	SIF	26.50%	2.80%	0.29	9.84E-277
SCARA3	SIF	58.90%	23.90%	0.45	4.34E-272
PFKP	SIF	84.40%	57.10%	0.53	3.48E-271
THBS4	SIF	76.60%	41.50%	0.52	2.88E-270
NUPR1	SIF	96.70%	80.60%	0.47	1.91E-264
ENPP1	SIF	66.40%	31.00%	0.52	5.13E-261
UGDH	SIF	90.80%	65.30%	0.47	2.29E-260
REXO2	SIF	94.60%	74.40%	0.43	1.67E-258
MMP3	SIF	40.90%	17.90%	1.55	1.42E-256
C11orf96	SIF	62.80%	31.70%	0.57	2.43E-255
MT1G	SIF	41.40%	11.90%	0.63	3.89E-255
TNXB	SIF	93.50%	65.40%	0.42	3.60E-253
FABP3	SIF	41.90%	15.60%	1.03	4.46E-253
ADAMTS5	SIF	40.60%	13.40%	0.43	1.77E-251
CHPF	SIF	57.60%	25.00%	0.41	2.11E-251
DNASE1L3	SIF	31.80%	6.60%	0.49	1.31E-250
NTN4	SIF	52.50%	21.00%	0.40	9.79E-249
ANK3	SIF	27.60%	4.10%	0.26	8.61E-245
TREM1	SIF	65.50%	38.30%	0.58	3.19E-243
CRYAB	SIF	88.60%	61.30%	0.54	1.76E-238
CREB5	SIF	73.80%	41.80%	0.43	2.02E-238
MT1E	SIF	91.00%	66.50%	0.56	3.78E-231
ITM2B	SIF	99.90%	99.50%	0.39	1.30E-226
DYSF	SIF	26.40%	4.50%	0.34	1.01E-224
TPPP3	SIF	95.60%	79.90%	0.58	1.07E-222
CAV1	SIF	96.20%	74.10%	0.34	7.18E-220
GPR1	SIF	23.30%	4.40%	0.50	7.81E-220
AMTN	SIF	21.00%	5.60%	1.16	5.55E-219
VCAM1	SIF	61.80%	35.50%	0.78	2.30E-215
SAT1	SIF	98.40%	94.00%	0.34	4.34E-215
TMEM100	SIF	65.50%	33.40%	0.44	5.96E-215
COX7A1	SIF	87.10%	63.40%	0.40	3.88E-212

ANGPTL2	SIF	96.00%	74.70%	0.38	6.52E-208
RABAC1	SIF	95.50%	85.80%	0.40	2.29E-207
FAM134B	SIF	74.30%	44.40%	0.39	3.32E-206
BMP2	SIF	30.30%	11.30%	0.46	5.54E-205
SEC61G	SIF	95.20%	87.00%	0.35	1.01E-203
MT1M	SIF	81.00%	52.70%	0.56	2.86E-203
SEMA5A	SIF	29.70%	6.60%	0.33	5.14E-202
EMP3	SIF	98.40%	92.90%	0.50	2.34E-198
TNFRSF11B	SIF	42.90%	33.60%	0.71	1.68E-194
FXYD5	SIF	90.50%	79.00%	0.43	8.23E-193
SLC9A3R1	SIF	48.50%	19.80%	0.30	8.26E-192
ACKR3	SIF	74.00%	43.00%	0.39	2.50E-191
GPRC5A	SIF	84.30%	56.40%	0.36	4.29E-191
COX7A2	SIF	96.30%	91.50%	0.30	1.28E-190
CDON	SIF	57.50%	27.40%	0.32	3.32E-190
FXYD6	SIF	57.00%	26.40%	0.32	4.29E-189
SLC2A1	SIF	39.90%	13.90%	0.27	2.11E-188
SGCA	SIF	45.10%	17.20%	0.31	1.24E-185
FMOD	SIF	77.90%	57.00%	0.62	4.49E-185
SEC11C	SIF	57.20%	27.30%	0.29	2.47E-182
NDP	SIF	28.60%	7.00%	0.26	1.50E-178
VASN	SIF	83.50%	58.00%	0.40	8.47E-176
BZW1	SIF	91.70%	78.20%	0.35	1.26E-174
S100A3	SIF	43.10%	17.00%	0.33	4.28E-174
NDFIP1	SIF	92.00%	77.40%	0.36	1.44E-173
GULP1	SIF	50.40%	22.50%	0.29	1.63E-173
HTRA4	SIF	19.30%	2.80%	0.30	1.66E-171
GUK1	SIF	95.20%	88.80%	0.31	5.85E-171
DIRC3	SIF	16.00%	1.50%	0.33	3.77E-170
CSGALNACT1	SIF	52.60%	24.60%	0.31	1.57E-168
SPARCL1	SIF	88.10%	71.80%	0.62	2.48E-165
GABARAPL1	SIF	75.30%	51.70%	0.35	9.43E-163
COX17	SIF	81.50%	61.20%	0.34	1.00E-162
INO80C	SIF	47.70%	22.40%	0.28	6.26E-157
OST4	SIF	97.20%	92.20%	0.27	6.32E-156
BTG2	SIF	71.60%	53.50%	0.44	6.41E-156
SBDS	SIF	92.90%	79.20%	0.32	1.00E-154
PPDPF	SIF	95.50%	89.30%	0.32	2.75E-154
SOD3	SIF	43.40%	18.10%	0.36	9.49E-152
ISLR	SIF	88.40%	65.00%	0.30	2.38E-151
CTGF	SIF	90.50%	68.30%	0.32	1.42E-150

CIB1	SIF	84.90%	68.30%	0.31	4.86E-149
APLP2	SIF	94.50%	84.90%	0.33	5.94E-144
RERG	SIF	70.90%	44.10%	0.28	5.31E-141
SERPINA1	SIF	27.40%	8.00%	0.37	1.45E-139
FAM180A	SIF	51.20%	25.50%	0.29	9.48E-139
HMGCS1	SIF	30.00%	20.50%	0.27	5.83E-137
VIMP	SIF	89.70%	72.10%	0.29	2.92E-136
CUX1	SIF	62.10%	36.50%	0.26	1.43E-133
NDUFA4	SIF	97.10%	91.20%	0.27	3.09E-133
ANKH	SIF	76.80%	52.70%	0.33	9.26E-133
TIMP1	SIF	99.40%	95.30%	0.42	1.31E-132
MRPL17	SIF	53.20%	29.30%	0.27	3.14E-132
SSR4	SIF	94.10%	87.50%	0.28	5.99E-132
RGS16	SIF	74.20%	50.60%	0.45	1.34E-131
TSPAN15	SIF	42.10%	19.20%	0.25	1.95E-128
NUCB2	SIF	82.10%	63.50%	0.33	4.57E-128
PGAM1	SIF	83.30%	68.50%	0.34	6.30E-128
MINOS1	SIF	93.90%	84.00%	0.27	6.61E-128
DSTN	SIF	99.20%	95.40%	0.29	9.50E-127
GAPDH	SIF	99.20%	99.40%	0.32	1.07E-120
PENK	SIF	11.70%	5.40%	1.02	1.03E-119
PDLIM5	SIF	67.00%	43.50%	0.26	1.16E-117
TIMP2	SIF	95.00%	81.90%	0.27	1.07E-116
CYB5R3	SIF	94.40%	80.30%	0.25	2.75E-114
ITGB5	SIF	62.10%	41.20%	0.30	7.73E-114
ITGAV	SIF	63.70%	40.40%	0.26	1.35E-113
C19orf10	SIF	89.40%	77.20%	0.29	1.13E-112
ANGPTL4	SIF	80.10%	59.70%	0.41	3.84E-112
AXL	SIF	71.60%	49.80%	0.31	3.14E-111
PRDX1	SIF	98.30%	95.10%	0.27	4.35E-111
TMED3	SIF	69.70%	49.60%	0.27	4.80E-111
BLOC1S2	SIF	65.90%	43.90%	0.26	9.02E-110
CD82	SIF	50.70%	27.80%	0.25	3.38E-109
TMED9	SIF	91.60%	79.10%	0.26	5.70E-109
CRIP1	SIF	93.10%	83.10%	0.37	2.06E-106
CFI	SIF	65.40%	41.70%	0.27	5.88E-106
PMP22	SIF	95.40%	84.60%	0.27	6.22E-106
KDELR3	SIF	61.90%	38.70%	0.26	1.16E-105
CYSTM1	SIF	81.60%	64.70%	0.25	7.94E-104
PHLDA2	SIF	70.70%	49.10%	0.31	4.81E-103
AKR1B1	SIF	73.60%	56.40%	0.32	7.85E-102

APP	SIF	87.50%	75.30%	0.26	1.53E-101
CADM3	SIF	38.40%	18.00%	0.30	1.96E-101
SOX9	SIF	39.30%	20.10%	0.28	2.45E-101
C10orf54	SIF	80.50%	60.80%	0.25	2.35E-95
P4HB	SIF	92.70%	81.40%	0.25	4.39E-88
XBP1	SIF	86.50%	72.50%	0.27	2.00E-87
PRDX4	SIF	83.90%	68.60%	0.25	1.29E-86
CXCL1	SIF	55.10%	42.50%	0.80	5.73E-86
CILP	SIF	43.80%	34.30%	0.60	8.72E-85
KCNMA1	SIF	47.80%	28.20%	0.26	4.16E-82
ITGBL1	SIF	31.00%	15.10%	0.25	8.28E-81
HSPB1	SIF	97.10%	93.40%	0.31	2.05E-76
SERPINE2	SIF	45.10%	30.50%	0.47	6.11E-74
CCL20	SIF	20.60%	10.30%	1.00	3.07E-70
CDA	SIF	35.40%	19.60%	0.28	4.39E-69
PTPRS	SIF	47.40%	31.40%	0.26	3.28E-58
C8orf4	SIF	17.70%	9.90%	0.26	4.02E-32
CXCL14	SIF	10.60%	6.20%	0.60	5.36E-18
WISP2	SSF	84.20%	37.50%	1.88	0.00E+00
IGF1	SSF	72.30%	15.70%	1.88	0.00E+00
CXCL12	SSF	71.80%	25.10%	1.74	0.00E+00
COL1A1	SSF	93.60%	47.50%	1.73	0.00E+00
COL3A1	SSF	93.40%	58.70%	1.69	0.00E+00
MMP2	SSF	88.60%	35.50%	1.56	0.00E+00
MFAP5	SSF	54.10%	10.70%	1.53	0.00E+00
SERPINF1	SSF	79.40%	44.60%	1.44	0.00E+00
DPT	SSF	97.40%	57.50%	1.42	0.00E+00
COL14A1	SSF	86.90%	23.30%	1.36	0.00E+00
COL1A2	SSF	99.70%	75.60%	1.28	0.00E+00
VCAN	SSF	91.30%	48.40%	1.24	0.00E+00
SFRP4	SSF	48.40%	10.80%	1.22	0.00E+00
EFEMP1	SSF	82.70%	48.20%	1.20	0.00E+00
ASPN	SSF	96.20%	58.40%	1.19	0.00E+00
C1R	SSF	97.80%	66.90%	1.17	0.00E+00
C1S	SSF	97.80%	68.80%	1.13	0.00E+00
CFH	SSF	87.70%	44.40%	1.13	0.00E+00
AEBP1	SSF	83.70%	31.70%	1.05	0.00E+00
PDGFRL	SSF	78.10%	30.20%	1.04	0.00E+00
DCN	SSF	99.90%	84.60%	1.00	0.00E+00
FSTL1	SSF	97.70%	67.20%	1.00	0.00E+00
IGFBP4	SSF	79.90%	55.10%	0.99	0.00E+00

IGFBP6	SSF	92.30%	68.50%	0.99	0.00E+00
PPAP2A	SSF	84.80%	42.30%	0.98	0.00E+00
FHL1	SSF	92.20%	62.40%	0.98	0.00E+00
CRABP2	SSF	55.40%	17.60%	0.96	0.00E+00
COL6A2	SSF	99.60%	74.50%	0.96	0.00E+00
COL6A1	SSF	97.50%	67.60%	0.92	0.00E+00
CP	SSF	55.90%	15.00%	0.91	0.00E+00
SCG2	SSF	31.00%	3.70%	0.91	0.00E+00
SPON2	SSF	51.10%	8.70%	0.88	0.00E+00
COL6A3	SSF	91.20%	58.40%	0.88	0.00E+00
SERPING1	SSF	96.70%	72.90%	0.82	0.00E+00
THY1	SSF	82.50%	36.40%	0.81	0.00E+00
MXRA8	SSF	81.80%	42.60%	0.80	0.00E+00
BGN	SSF	97.40%	70.00%	0.80	0.00E+00
PLTP	SSF	85.00%	56.20%	0.79	0.00E+00
SPARC	SSF	95.30%	72.50%	0.76	0.00E+00
PLA2G2A	SSF	98.90%	84.50%	0.76	0.00E+00
ECM2	SSF	73.40%	27.60%	0.75	0.00E+00
CTSK	SSF	86.70%	54.10%	0.74	0.00E+00
S100A13	SSF	94.20%	67.00%	0.74	0.00E+00
GSN	SSF	98.20%	86.90%	0.74	0.00E+00
FBLN2	SSF	52.50%	14.50%	0.73	0.00E+00
PCOLCE	SSF	98.50%	68.80%	0.73	0.00E+00
FBN1	SSF	71.30%	32.30%	0.73	0.00E+00
PLAC9	SSF	97.50%	71.20%	0.72	0.00E+00
PRSS23	SSF	90.60%	64.30%	0.72	0.00E+00
C1QTNF3	SSF	62.50%	26.30%	0.72	0.00E+00
SFRP1	SSF	44.50%	15.80%	0.72	0.00E+00
MXRA5	SSF	63.90%	21.90%	0.72	0.00E+00
COMP	SSF	91.30%	64.80%	0.72	0.00E+00
OLFML3	SSF	52.80%	8.90%	0.71	0.00E+00
FNDC1	SSF	44.10%	5.30%	0.71	0.00E+00
SMOC2	SSF	57.00%	17.60%	0.69	0.00E+00
COL5A2	SSF	69.10%	38.60%	0.68	0.00E+00
TUBB2B	SSF	49.60%	12.80%	0.67	0.00E+00
CADM1	SSF	43.70%	9.10%	0.66	0.00E+00
TUBA1A	SSF	89.10%	70.20%	0.66	0.00E+00
SSPN	SSF	75.00%	32.80%	0.66	0.00E+00
LHFP	SSF	82.50%	49.20%	0.66	0.00E+00
ANGPTL1	SSF	72.50%	38.70%	0.65	0.00E+00
EPHX1	SSF	74.90%	42.50%	0.65	0.00E+00

SRPX	SSF	43.60%	6.30%	0.64	0.00E+00
CNN3	SSF	87.30%	50.80%	0.62	0.00E+00
AKR1C1	SSF	78.30%	42.90%	0.62	0.00E+00
ITM2C	SSF	64.60%	29.60%	0.62	0.00E+00
FGF7	SSF	32.40%	4.50%	0.62	0.00E+00
FBLN1	SSF	46.60%	11.60%	0.61	0.00E+00
NNMT	SSF	98.70%	76.30%	0.60	0.00E+00
PODN	SSF	36.70%	3.50%	0.60	0.00E+00
ELN	SSF	54.20%	21.40%	0.59	0.00E+00
CCDC80	SSF	97.50%	68.60%	0.58	0.00E+00
AKR1C2	SSF	73.20%	40.30%	0.57	0.00E+00
LUM	SSF	99.90%	86.30%	0.57	0.00E+00
LIMA1	SSF	87.10%	57.60%	0.56	0.00E+00
FAM180B	SSF	39.70%	5.10%	0.56	0.00E+00
SDC2	SSF	91.60%	65.50%	0.56	0.00E+00
THBS3	SSF	69.60%	35.00%	0.55	0.00E+00
PDGFRA	SSF	69.80%	36.10%	0.54	0.00E+00
ECM1	SSF	73.00%	44.30%	0.54	0.00E+00
CPQ	SSF	82.50%	52.50%	0.54	0.00E+00
SERPINH1	SSF	76.90%	45.40%	0.52	0.00E+00
TSHZ2	SSF	68.00%	30.90%	0.51	0.00E+00
LGALS1	SSF	99.40%	95.40%	0.50	0.00E+00
ANGPTL5	SSF	46.70%	13.80%	0.49	0.00E+00
ANTXR1	SSF	56.10%	24.40%	0.47	0.00E+00
VIM	SSF	100.00%	100.00%	0.47	0.00E+00
IFITM3	SSF	98.90%	87.90%	0.46	0.00E+00
THBS2	SSF	43.10%	11.10%	0.46	0.00E+00
TMEM98	SSF	56.80%	22.50%	0.45	0.00E+00
FBLN5	SSF	36.40%	6.50%	0.44	0.00E+00
CDH11	SSF	37.80%	7.10%	0.41	0.00E+00
PPIB	SSF	98.20%	88.00%	0.39	0.00E+00
AKR1C3	SSF	52.80%	18.40%	0.37	0.00E+00
MGP	SSF	99.80%	88.90%	0.37	0.00E+00
TMEM119	SSF	30.90%	3.80%	0.36	0.00E+00
MYL9	SSF	86.60%	46.30%	0.36	0.00E+00
LAPTM4A	SSF	99.30%	91.90%	0.35	0.00E+00
CALD1	SSF	97.70%	72.00%	0.29	0.00E+00
MAP1B	SSF	43.00%	14.20%	0.25	0.00E+00
PDGFRB	SSF	57.20%	23.10%	0.47	1.77473073609811e-317
SFRP2	SSF	27.70%	4.30%	1.16	0.00E+00
EID1	SSF	97.50%	84.50%	0.37	0.00E+00

AHNAK2	SSF	43.70%	13.20%	0.46	4.62E-308
COL8A1	SSF	45.20%	15.90%	0.52	1.12E-307
PRRX1	SSF	82.40%	52.10%	0.45	1.67E-301
MEG3	SSF	80.80%	47.80%	0.42	7.43E-287
ANGPTL2	SSF	94.60%	69.10%	0.29	4.88E-285
MDK	SSF	34.40%	8.20%	0.52	1.63E-284
EFEMP2	SSF	70.60%	40.60%	0.41	2.01E-281
PTGFR	SSF	30.60%	6.90%	0.42	3.00E-279
GPNMB	SSF	84.40%	57.40%	0.56	1.12E-276
MGST1	SSF	66.90%	40.20%	0.53	2.86E-276
FGFBP2	SSF	60.80%	30.40%	0.99	8.57E-276
LGALS3	SSF	99.20%	94.70%	0.37	5.13E-267
CYP1B1	SSF	89.40%	68.30%	0.59	3.37E-265
CRISPLD1	SSF	52.70%	22.50%	0.44	8.76E-260
SGCE	SSF	43.00%	16.20%	0.43	9.18E-257
JUNB	SSF	98.90%	97.80%	0.62	1.56E-256
CTHRC1	SSF	30.50%	7.40%	0.55	7.59E-256
CYBRD1	SSF	79.70%	55.20%	0.41	4.07E-254
ADD3	SSF	50.40%	23.60%	0.41	7.84E-254
OGN	SSF	73.30%	41.20%	0.46	1.20E-253
EPB41L2	SSF	78.10%	51.80%	0.42	1.34E-251
ID3	SSF	86.90%	62.80%	0.57	2.24E-251
RARRES1	SSF	28.40%	5.70%	0.56	6.24E-247
LTBP2	SSF	50.80%	23.30%	0.48	1.70E-246
ISLR	SSF	87.20%	58.50%	0.32	1.01E-243
CFD	SSF	86.40%	81.80%	1.17	5.07E-238
COL5A1	SSF	53.30%	26.70%	0.52	5.78E-238
EBF1	SSF	57.70%	29.90%	0.44	1.34E-235
ISM1	SSF	26.10%	4.80%	0.28	6.47E-233
PPIC	SSF	87.40%	64.90%	0.38	1.06E-231
ANK2	SSF	46.50%	19.70%	0.35	5.79E-229
PTRF	SSF	91.10%	69.20%	0.36	9.93E-229
BICC1	SSF	34.90%	11.10%	0.33	1.22E-228
NUCKS1	SSF	94.80%	79.70%	0.35	1.76E-228
ABI3BP	SSF	84.80%	56.90%	0.36	6.67E-226
NFIX	SSF	54.10%	28.00%	0.36	2.17E-223
PTN	SSF	32.40%	9.90%	0.62	7.18E-219
NFIB	SSF	66.50%	40.00%	0.40	6.26E-218
PDLIM3	SSF	69.30%	41.70%	0.39	2.81E-217
ADIRF	SSF	95.90%	82.30%	0.34	1.30E-216
CD248	SSF	38.10%	13.70%	0.38	3.29E-212

SERTAD4	SSF	30.80%	8.20%	0.31	1.59E-211
LSP1	SSF	83.80%	57.50%	0.33	3.94E-211
NFIC	SSF	87.40%	66.50%	0.34	3.69E-208
ANXA2	SSF	99.60%	97.80%	0.30	5.07E-208
RCN3	SSF	80.50%	54.00%	0.35	3.18E-207
SSR2	SSF	90.40%	75.60%	0.32	4.20E-207
GEM	SSF	85.90%	66.20%	0.59	4.39E-207
DDAH2	SSF	86.20%	69.20%	0.37	3.44E-205
IFITM2	SSF	93.60%	82.90%	0.40	1.53E-201
TUBB	SSF	89.90%	77.30%	0.35	1.14E-197
AKAP12	SSF	33.90%	10.60%	0.38	3.23E-195
LMNA	SSF	99.60%	97.80%	0.25	4.37E-194
DPP4	SSF	26.90%	6.40%	0.30	2.83E-193
P4HA2	SSF	63.50%	39.40%	0.35	2.15E-190
C1orf21	SSF	60.80%	35.60%	0.34	1.39E-189
MYC	SSF	77.10%	55.30%	0.55	3.35E-189
CERCAM	SSF	66.30%	40.40%	0.37	7.50E-188
SLPI	SSF	18.70%	3.10%	0.58	2.21E-187
SPOCK1	SSF	30.40%	10.30%	0.29	2.24E-184
MRC2	SSF	64.40%	40.50%	0.36	3.65E-182
TNC	SSF	69.90%	57.60%	0.60	9.96E-182
DIO2	SSF	31.70%	10.10%	0.40	2.22E-181
FKBP9	SSF	54.30%	29.90%	0.35	2.66E-181
OSR2	SSF	63.70%	39.90%	0.45	9.26E-181
FHL2	SSF	47.10%	21.70%	0.33	2.92E-178
CYP27A1	SSF	35.60%	14.20%	0.27	6.74E-173
IL11RA	SSF	34.40%	13.90%	0.27	2.51E-172
TUBB2A	SSF	79.70%	61.90%	0.41	3.88E-170
CHI3L2	SSF	43.20%	21.50%	0.72	8.16E-170
PPFIBP1	SSF	51.30%	26.70%	0.30	2.25E-169
RRBP1	SSF	89.90%	72.30%	0.30	1.39E-168
LGALS3BP	SSF	63.00%	43.60%	0.48	1.44E-167
CHI3L1	SSF	34.40%	16.20%	1.17	1.97E-166
LTBP1	SSF	58.20%	33.30%	0.35	3.10E-165
CES1	SSF	34.90%	15.60%	0.39	4.91E-164
GLIS3	SSF	47.70%	25.20%	0.35	1.57E-162
EMP2	SSF	84.30%	63.20%	0.30	1.88E-162
RGS3	SSF	45.70%	26.20%	0.37	4.03E-161
NOV	SSF	30.50%	11.30%	0.48	4.05E-161
MFGE8	SSF	78.30%	57.80%	0.46	3.91E-160
PLXDC1	SSF	40.40%	17.10%	0.28	6.88E-160

C3	SSF	17.80%	3.00%	0.30	2.84E-158
GLT8D2	SSF	57.60%	35.30%	0.31	7.49E-158
SSC5D	SSF	49.00%	27.40%	0.29	4.30E-156
PLEKHA5	SSF	29.80%	10.50%	0.26	2.06E-154
C2	SSF	54.40%	32.10%	0.41	2.06E-154
TSPAN4	SSF	74.00%	54.10%	0.33	1.12E-150
C1QTNF2	SSF	41.40%	19.90%	0.32	1.21E-146
MRGPRF	SSF	50.50%	28.00%	0.31	1.00E-145
S100A16	SSF	59.50%	37.00%	0.33	3.12E-145
PAMR1	SSF	20.70%	4.90%	0.25	3.74E-145
SERTAD4-AS1	SSF	39.20%	17.40%	0.26	3.80E-145
TTC3	SSF	74.00%	54.30%	0.30	3.83E-145
RBMS3	SSF	40.40%	20.00%	0.28	1.28E-144
CYR61	SSF	76.40%	54.80%	0.42	5.10E-144
EGR1	SSF	89.50%	74.40%	0.36	2.70E-142
DBN1	SSF	37.20%	19.80%	0.30	8.36E-142
RAB34	SSF	75.60%	55.00%	0.28	8.85E-142
EDIL3	SSF	45.30%	24.40%	0.30	2.87E-139
EEA1	SSF	67.40%	48.20%	0.30	6.92E-139
PDLIM2	SSF	62.20%	40.70%	0.28	2.52E-138
ADH5	SSF	49.10%	29.00%	0.29	5.11E-138
ACTG1	SSF	99.80%	98.50%	0.25	8.28E-138
ENPP2	SSF	23.80%	7.00%	0.26	9.31E-138
NGFRAP1	SSF	76.30%	54.70%	0.29	3.54E-136
LRRN4CL	SSF	46.70%	26.50%	0.29	4.04E-136
FAP	SSF	51.20%	30.90%	0.34	1.39E-134
DHRS3	SSF	31.10%	12.70%	0.28	1.62E-134
LY6E	SSF	83.60%	64.30%	0.30	2.93E-134
GNG12	SSF	57.90%	36.90%	0.27	5.94E-134
SRI	SSF	80.40%	62.90%	0.28	6.69E-134
SCPEP1	SSF	60.40%	40.50%	0.30	7.56E-134
FGFR1	SSF	66.50%	46.60%	0.30	1.31E-133
SNAI2	SSF	36.20%	16.80%	0.35	6.95E-133
SNX9	SSF	65.10%	46.30%	0.30	5.27E-132
PPAP2B	SSF	74.90%	53.50%	0.38	1.23E-131
PAM	SSF	75.40%	53.60%	0.27	1.56E-130
C5orf15	SSF	59.70%	38.90%	0.27	3.21E-128
ITGB1	SSF	92.70%	81.80%	0.25	5.10E-128
PRKCDBP	SSF	87.50%	69.20%	0.26	3.36E-127
MATN2	SSF	38.30%	20.30%	0.33	1.80E-126
RARRES3	SSF	36.40%	16.80%	0.27	1.72E-125

ENAH	SSF	52.60%	31.10%	0.28	2.00E-125
DKK3	SSF	67.20%	46.30%	0.44	1.98E-124
A4GALT	SSF	49.50%	29.90%	0.27	2.20E-121
ESD	SSF	79.20%	63.10%	0.26	3.05E-119
ZFP36L1	SSF	94.50%	86.80%	0.30	7.15E-117
NOVA1	SSF	48.60%	27.90%	0.28	1.67E-116
MFAP4	SSF	70.10%	48.10%	0.32	2.00E-116
NFIA	SSF	64.10%	45.50%	0.29	2.73E-116
FXYD1	SSF	72.50%	51.30%	0.26	2.89E-116
CD47	SSF	79.20%	63.20%	0.27	3.39E-116
ZNF106	SSF	54.40%	35.90%	0.27	4.80E-116
SCRG1	SSF	51.70%	30.70%	0.46	5.82E-116
CRISPLD2	SSF	41.50%	21.50%	0.29	2.45E-114
NUCB1	SSF	83.30%	67.10%	0.25	3.23E-114
CD99	SSF	98.20%	94.90%	0.26	1.71E-113
MAP1A	SSF	68.40%	47.30%	0.27	3.50E-113
MEDAG	SSF	61.70%	40.80%	0.30	5.44E-113
CTGF	SSF	85.10%	66.00%	0.31	7.81E-113
CALU	SSF	84.40%	68.90%	0.27	1.09E-112
GOLIM4	SSF	74.60%	58.20%	0.30	5.89E-112
FAM3C	SSF	71.70%	55.20%	0.27	6.65E-111
PLS3	SSF	65.80%	46.10%	0.27	1.09E-110
ABHD2	SSF	51.00%	36.40%	0.28	2.38E-110
SEP11	SSF	52.50%	32.10%	0.25	7.47E-109
RARRES2	SSF	21.40%	7.00%	0.34	7.51E-107
ZBTB20	SSF	56.10%	38.00%	0.25	1.05E-106
TAX1BP3	SSF	63.10%	46.30%	0.25	1.57E-106
OMD	SSF	27.20%	11.60%	0.26	3.04E-105
CD70	SSF	25.30%	10.00%	0.26	3.92E-105
PLAU	SSF	45.90%	25.20%	0.30	5.39E-104
RASD1	SSF	46.10%	30.70%	0.46	9.25E-103
UBC	SSF	99.70%	99.50%	0.26	1.25E-102
OLFML2B	SSF	38.80%	20.90%	0.26	3.84E-102
GAS1	SSF	38.50%	20.30%	0.27	3.08E-101
HEXA	SSF	70.00%	52.70%	0.25	6.17E-100
TMEM176A	SSF	37.60%	26.20%	0.43	3.54E-99
SOX4	SSF	72.70%	54.70%	0.31	1.98E-98
STEAP1	SSF	45.80%	27.60%	0.26	2.74E-98
FAM46A	SSF	73.00%	55.40%	0.33	2.29E-97
LBH	SSF	66.20%	47.00%	0.28	2.83E-96
GAP43	SSF	23.40%	9.60%	0.25	3.54E-93

ARID5B	SSF	86.10%	70.30%	0.26	4.94E-90
IRF1	SSF	78.70%	63.80%	0.32	3.28E-89
RBP4	SSF	21.30%	8.40%	0.29	3.23E-88
PTGDS	SSF	27.30%	16.00%	0.91	9.39E-88
MMP14	SSF	50.90%	35.80%	0.27	1.40E-86
KCNQ1OT1	SSF	78.10%	62.00%	0.27	2.08E-83
GGT5	SSF	22.20%	8.70%	0.33	5.43E-82
SCARA5	SSF	51.60%	33.30%	0.28	1.91E-81
SOCS3	SSF	77.70%	65.50%	0.31	3.28E-81
CTSL	SSF	85.60%	73.60%	0.26	8.74E-79
IL6	SSF	42.80%	30.30%	0.66	9.38E-77
LTBP4	SSF	51.90%	35.50%	0.28	6.03E-75
FOSB	SSF	89.90%	81.80%	0.26	1.43E-66
ID1	SSF	57.00%	42.20%	0.31	8.79E-66
NTRK2	SSF	38.40%	25.50%	0.28	4.55E-65
FOS	SSF	96.60%	91.10%	0.28	6.32E-63
GADD45B	SSF	94.70%	90.30%	0.26	3.04E-62
ZFP36	SSF	94.10%	90.90%	0.27	6.79E-58
GDF15	SSF	14.90%	6.70%	0.28	1.83E-51
TMEM176B	SSF	40.00%	30.80%	0.31	1.97E-43
TNFRSF12A	SSF	67.80%	60.70%	0.25	7.97E-36

Supplementary Table S1B. List of differentially expressed genes in osteoarthritic HLA-DRA+ synoviocytes.

Gene	Putative cell type of the cluster	The percentage of cells expressing the gene in the cluster	The percentage of cells expressing the gene in the other clusters	Log fold-change of the average expression between the two groups	Adjusted p-value (Bonferroni correction)
SEPP1	IR-Mϕ	97.30%	58.10%	1.42	7.33E-112
AIF1	IR-Mϕ	99.00%	92.50%	0.83	1.69E-103
MTRNR2L2	IR-Mϕ	96.60%	66.30%	1.26	5.08E-103
FOLR2	IR-Mϕ	95.60%	57.00%	1.08	6.66E-101
RNASE1	IR-Mϕ	98.60%	69.00%	1.11	3.29E-94
TXNIP	IR-Mϕ	85.70%	33.80%	1.15	4.42E-94
C1QC	IR-Mϕ	98.60%	66.50%	0.93	4.34E-93
PDK4	IR-Mϕ	70.10%	18.20%	1.25	3.71E-91
C1QA	IR-Mϕ	98.80%	72.30%	0.97	1.40E-89
MAF	IR-Mϕ	77.20%	29.40%	0.97	7.81E-85
TGFBI	IR-Mϕ	97.50%	83.20%	0.86	6.91E-83
FOS	IR-Mϕ	98.00%	78.20%	0.79	3.32E-81
DAB2	IR-Mϕ	91.70%	57.90%	0.94	5.23E-78
MTRNR2L1	IR-Mϕ	73.30%	26.80%	1.18	3.72E-76
TYROBP	IR-Mϕ	100.00%	97.40%	0.51	9.92E-76
NPC2	IR-Mϕ	99.50%	97.40%	0.62	1.39E-75
MARCO	IR-Mϕ	91.20%	57.30%	0.98	1.27E-70
MAFB	IR-Mϕ	90.70%	59.40%	0.86	3.93E-68
CTSB	IR-Mϕ	97.50%	81.50%	0.74	2.52E-66
CYBB	IR-Mϕ	89.00%	57.00%	0.79	3.36E-65
LYVE1	IR-Mϕ	74.40%	31.20%	1.10	2.68E-64
CD68	IR-Mϕ	94.90%	80.30%	0.70	1.26E-63
VAMP8	IR-Mϕ	94.60%	81.20%	0.64	7.69E-63
HPGDS	IR-Mϕ	62.60%	23.40%	0.91	1.86E-61
SLC40A1	IR-Mϕ	66.00%	24.00%	1.00	1.73E-60
S100A4	IR-Mϕ	100.00%	99.90%	0.51	1.49E-58
STAB1	IR-Mϕ	80.10%	42.80%	0.82	1.53E-58
CFD	IR-Mϕ	96.90%	81.00%	0.64	3.30E-58
C1QB	IR-Mϕ	96.40%	68.30%	0.81	5.70E-58
BLVRB	IR-Mϕ	84.20%	58.20%	0.75	1.12E-57
HSPA1B	IR-Mϕ	76.70%	37.30%	0.83	2.70E-57
SPI1	IR-Mϕ	75.60%	43.40%	0.79	4.23E-57

FCGRT	IR-Mϕ	95.20%	88.90%	0.66	1.82E-56
MTRNR2L12	IR-Mϕ	83.90%	53.50%	0.77	2.98E-56
CTSD	IR-Mϕ	92.90%	69.40%	0.74	1.07E-55
MTRNR2L8	IR-Mϕ	83.20%	48.00%	0.73	8.24E-55
CD99	IR-Mϕ	97.30%	91.50%	0.61	1.08E-54
EMB	IR-Mϕ	76.20%	42.70%	0.78	1.42E-53
LGMN	IR-Mϕ	85.20%	59.40%	0.88	4.05E-52
DUSP1	IR-Mϕ	87.90%	72.60%	0.81	1.98E-51
HSPB1	IR-Mϕ	94.10%	74.50%	0.68	5.89E-51
GPR34	IR-Mϕ	59.30%	21.00%	0.75	1.16E-50
ARPC3	IR-Mϕ	97.50%	93.00%	0.48	6.82E-50
FRMD4B	IR-Mϕ	75.40%	40.90%	0.64	1.17E-49
CYBA	IR-Mϕ	99.50%	97.00%	0.45	1.34E-49
LAPTM5	IR-Mϕ	98.00%	95.10%	0.49	4.53E-48
ITM2B	IR-Mϕ	99.70%	97.50%	0.44	1.20E-47
PMP22	IR-Mϕ	79.60%	47.70%	0.71	1.41E-47
PYCARD	IR-Mϕ	81.70%	56.60%	0.61	1.90E-47
ACTB	IR-Mϕ	100.00%	99.90%	0.44	2.69E-47
COLEC12	IR-Mϕ	65.90%	29.70%	0.67	3.16E-47
PLTP	IR-Mϕ	78.60%	48.30%	0.88	9.79E-47
LILRB5	IR-Mϕ	67.90%	31.60%	0.65	1.36E-46
F13A1	IR-Mϕ	73.00%	40.10%	0.96	3.26E-46
HSPA1A	IR-Mϕ	83.70%	59.50%	0.85	4.10E-46
TSC22D3	IR-Mϕ	87.30%	62.30%	0.60	4.23E-46
ARHGAP18	IR-Mϕ	75.00%	43.70%	0.62	2.59E-45
CD14	IR-Mϕ	93.50%	71.90%	0.61	1.72E-44
TSPO	IR-Mϕ	97.10%	92.00%	0.48	5.34E-44
JUN	IR-Mϕ	92.50%	79.10%	0.64	2.31E-42
RBPJ	IR-Mϕ	89.30%	72.40%	0.56	2.37E-42
VSIG4	IR-Mϕ	90.30%	75.40%	0.63	5.03E-42
ME1	IR-Mϕ	47.20%	13.70%	0.58	5.53E-41
SNX2	IR-Mϕ	71.60%	43.70%	0.60	7.75E-41
CSF1R	IR-Mϕ	72.80%	41.70%	0.60	2.72E-40
NCF1	IR-Mϕ	74.40%	43.60%	0.79	4.37E-40
ADAP2	IR-Mϕ	53.30%	20.50%	0.61	5.66E-39
RHOB	IR-Mϕ	67.60%	35.20%	0.66	1.09E-38
BLVRA	IR-Mϕ	60.60%	28.60%	0.60	1.46E-38
CTSZ	IR-Mϕ	94.60%	83.70%	0.58	1.52E-38
C4orf48	IR-Mϕ	46.00%	15.20%	0.61	9.42E-38
FCER1G	IR-Mϕ	99.20%	96.10%	0.40	3.09E-37
ALOX5AP	IR-Mϕ	87.30%	67.90%	0.55	3.35E-37

ALDH1A1	IR-Mϕ	55.00%	21.30%	0.64	4.65E-37
MAMDC2	IR-Mϕ	72.30%	40.40%	0.59	1.06E-36
LTC4S	IR-Mϕ	56.70%	24.60%	0.63	2.70E-36
S100A6	IR-Mϕ	100.00%	99.70%	0.45	4.93E-36
MS4A7	IR-Mϕ	79.10%	55.00%	0.59	5.19E-36
CTSS	IR-Mϕ	95.90%	92.30%	0.48	1.96E-35
PSAP	IR-Mϕ	99.20%	97.30%	0.43	2.28E-35
YBX1	IR-Mϕ	95.80%	88.90%	0.49	3.37E-35
COMT	IR-Mϕ	84.20%	65.00%	0.51	2.11E-34
CD163	IR-Mϕ	79.10%	50.30%	0.51	5.77E-34
SNX6	IR-Mϕ	66.00%	39.60%	0.58	1.20E-33
HNMT	IR-Mϕ	69.80%	43.60%	0.57	6.63E-33
RHOA	IR-Mϕ	93.90%	91.90%	0.41	7.80E-33
MS4A4A	IR-Mϕ	67.70%	39.10%	0.58	9.40E-33
PLD3	IR-Mϕ	75.70%	56.30%	0.65	2.45E-32
ANXA4	IR-Mϕ	73.00%	47.50%	0.60	2.62E-32
ZFP36L2	IR-Mϕ	78.10%	50.60%	0.53	4.28E-32
RNF130	IR-Mϕ	85.90%	72.40%	0.48	6.33E-32
TMEM176B	IR-Mϕ	66.90%	37.40%	0.65	8.29E-32
HSP90AA1	IR-Mϕ	98.00%	93.30%	0.48	1.18E-31
GIMAP4	IR-Mϕ	36.30%	9.20%	0.45	4.55E-31
PEPD	IR-Mϕ	67.10%	44.50%	0.65	5.32E-31
MEF2C	IR-Mϕ	68.90%	42.70%	0.55	1.27E-30
MS4A6A	IR-Mϕ	95.90%	86.70%	0.43	4.88E-30
GRN	IR-Mϕ	93.50%	88.00%	0.46	7.59E-30
ACP5	IR-Mϕ	65.40%	38.10%	0.62	2.39E-29
SH3BGRL	IR-Mϕ	90.30%	79.90%	0.43	1.22E-28
MPEG1	IR-Mϕ	54.70%	27.00%	0.53	2.00E-28
ARPC4	IR-Mϕ	72.50%	51.40%	0.51	2.21E-28
MT-ND2	IR-Mϕ	99.70%	100.00%	0.32	4.21E-28
CST3	IR-Mϕ	100.00%	99.20%	0.36	1.15E-27
CREG1	IR-Mϕ	64.30%	39.60%	0.55	1.36E-27
NUPR1	IR-Mϕ	68.80%	40.90%	0.58	2.22E-27
GPNMB	IR-Mϕ	77.40%	51.20%	0.49	4.56E-27
TNS1	IR-Mϕ	62.00%	35.40%	0.49	4.17E-26
ZFP36L1	IR-Mϕ	93.20%	83.60%	0.44	9.60E-26
KLF2	IR-Mϕ	44.80%	18.50%	0.53	1.43E-25
CHCHD10	IR-Mϕ	39.60%	13.80%	0.45	2.81E-25
HSP90B1	IR-Mϕ	83.70%	71.10%	0.54	3.58E-25
C2	IR-Mϕ	50.30%	24.10%	0.52	1.69E-24
ARHGDIB	IR-Mϕ	84.70%	72.30%	0.42	2.01E-24

FTL	IR-Mϕ	100.00%	100.00%	0.30	2.56E-24
SCN9A	IR-Mϕ	33.10%	9.80%	0.44	2.63E-24
UCP2	IR-Mϕ	61.00%	36.80%	0.48	2.83E-24
ACTG1	IR-Mϕ	98.80%	96.30%	0.31	7.37E-24
GIMAP7	IR-Mϕ	28.20%	6.50%	0.42	7.43E-24
UNC93B1	IR-Mϕ	45.20%	19.50%	0.43	1.14E-23
CAPZA2	IR-Mϕ	78.10%	63.60%	0.45	1.29E-23
SIGLEC1	IR-Mϕ	32.60%	9.50%	0.43	1.77E-23
DNAJB1	IR-Mϕ	80.80%	64.50%	0.61	1.98E-23
KCTD12	IR-Mϕ	73.00%	50.70%	0.45	2.96E-23
TMEM37	IR-Mϕ	24.60%	4.80%	0.32	3.37E-23
GLUL	IR-Mϕ	90.50%	79.40%	0.36	4.25E-23
LAMTOR4	IR-Mϕ	85.90%	74.80%	0.38	9.20E-23
SERPINF1	IR-Mϕ	72.00%	51.60%	0.43	1.02E-22
CAP1	IR-Mϕ	83.90%	72.30%	0.42	1.58E-22
ATP5D	IR-Mϕ	51.10%	27.20%	0.47	2.75E-22
LAMP1	IR-Mϕ	65.90%	44.60%	0.51	3.38E-22
MSR1	IR-Mϕ	75.60%	56.30%	0.46	9.31E-22
MCOLN1	IR-Mϕ	45.50%	21.40%	0.45	1.05E-21
CYTH4	IR-Mϕ	36.70%	13.40%	0.43	1.52E-21
ARRB2	IR-Mϕ	68.40%	48.50%	0.44	1.54E-21
TCEAL4	IR-Mϕ	57.00%	34.10%	0.48	2.01E-21
CALM2	IR-Mϕ	96.90%	93.90%	0.37	2.22E-21
GMFG	IR-Mϕ	82.50%	72.90%	0.43	3.44E-21
CALM3	IR-Mϕ	53.30%	30.20%	0.48	4.12E-21
GAS6	IR-Mϕ	50.10%	25.30%	0.49	5.11E-21
ATP5I	IR-Mϕ	82.30%	69.40%	0.39	6.19E-21
SLCO2B1	IR-Mϕ	48.20%	22.60%	0.42	9.06E-21
FOSB	IR-Mϕ	85.70%	78.50%	0.47	1.74E-20
P2RY14	IR-Mϕ	34.00%	12.40%	0.52	6.20E-20
ATP5H	IR-Mϕ	79.50%	66.70%	0.39	7.32E-20
C10orf118	IR-Mϕ	46.50%	24.10%	0.51	1.28E-19
EMP1	IR-Mϕ	88.30%	76.30%	0.43	1.58E-19
MT-ND5	IR-Mϕ	98.60%	98.70%	0.33	2.46E-19
AP2S1	IR-Mϕ	72.70%	56.60%	0.45	3.88E-19
RIN2	IR-Mϕ	41.90%	19.60%	0.45	6.82E-19
DAAM1	IR-Mϕ	48.90%	26.50%	0.42	1.41E-18
MAN1A1	IR-Mϕ	53.70%	31.40%	0.48	2.68E-18
KLF4	IR-Mϕ	82.70%	63.60%	0.32	4.08E-18
PTPN6	IR-Mϕ	39.90%	18.60%	0.41	5.42E-18
RENBP	IR-Mϕ	40.20%	18.20%	0.41	5.90E-18

GNAS	IR-Mϕ	85.10%	74.30%	0.40	7.89E-18
GNAI2	IR-Mϕ	79.50%	65.50%	0.37	9.22E-18
DHRS7	IR-Mϕ	63.80%	44.00%	0.40	9.99E-18
PFN1	IR-Mϕ	98.30%	98.30%	0.30	1.13E-17
ITGAM	IR-Mϕ	57.00%	35.50%	0.46	1.26E-17
RAB13	IR-Mϕ	65.00%	46.20%	0.39	2.08E-17
MT-ND3	IR-Mϕ	99.80%	99.70%	0.26	2.31E-17
MT-ND4L	IR-Mϕ	88.60%	81.40%	0.38	3.32E-17
GNG11	IR-Mϕ	42.10%	20.50%	0.44	3.48E-17
TIMD4	IR-Mϕ	32.90%	12.50%	0.45	6.78E-17
ECM1	IR-Mϕ	52.10%	29.90%	0.39	8.41E-17
PARVB	IR-Mϕ	44.50%	21.80%	0.34	1.10E-16
ATPIF1	IR-Mϕ	72.70%	55.20%	0.35	1.41E-16
NCF4	IR-Mϕ	49.40%	28.90%	0.41	1.41E-16
CTD-2337J16.1	IR-Mϕ	36.80%	15.90%	0.41	1.58E-16
S100A10	IR-Mϕ	99.50%	99.50%	0.37	2.61E-16
IFI16	IR-Mϕ	65.20%	45.70%	0.40	3.97E-16
7SK.2	IR-Mϕ	33.30%	14.20%	0.58	8.68E-16
RNF150	IR-Mϕ	21.20%	4.90%	0.26	9.99E-16
HSBP1	IR-Mϕ	69.30%	52.00%	0.38	1.09E-15
SLA	IR-Mϕ	33.10%	13.40%	0.35	1.11E-15
COX6B1	IR-Mϕ	90.30%	86.30%	0.30	1.27E-15
RP11-108M9.4	IR-Mϕ	26.10%	8.50%	0.33	2.20E-15
COX5B	IR-Mϕ	86.20%	76.30%	0.31	2.33E-15
NUCKS1	IR-Mϕ	70.80%	53.00%	0.35	4.34E-15
AP1B1	IR-Mϕ	54.70%	35.00%	0.38	5.21E-15
SERPINB6	IR-Mϕ	52.30%	32.50%	0.37	5.52E-15
SLC48A1	IR-Mϕ	28.50%	10.30%	0.32	5.85E-15
MFSD1	IR-Mϕ	72.30%	56.40%	0.33	8.00E-15
PET100	IR-Mϕ	69.10%	52.90%	0.36	1.01E-14
CANX	IR-Mϕ	73.70%	62.20%	0.36	1.06E-14
LAMP2	IR-Mϕ	64.50%	46.80%	0.36	1.14E-14
CMKLR1	IR-Mϕ	28.20%	10.20%	0.32	1.42E-14
DOK2	IR-Mϕ	60.80%	40.80%	0.38	1.46E-14
FAM105A	IR-Mϕ	43.80%	23.60%	0.38	1.46E-14
TMEM230	IR-Mϕ	59.30%	40.40%	0.36	1.57E-14
PRSS36	IR-Mϕ	20.20%	4.90%	0.27	1.72E-14
C3AR1	IR-Mϕ	56.40%	35.60%	0.39	1.78E-14
C21orf33	IR-Mϕ	31.60%	13.20%	0.35	2.01E-14
PLEC	IR-Mϕ	62.50%	44.00%	0.42	2.14E-14

UQCR11.1	IR-Mϕ	79.30%	73.50%	0.34	2.47E-14
TSPAN4	IR-Mϕ	57.60%	38.80%	0.42	2.48E-14
ASAH1	IR-Mϕ	67.10%	52.10%	0.41	2.53E-14
TNFAIP8L2	IR-Mϕ	20.40%	5.30%	0.27	2.73E-14
SRSF7	IR-Mϕ	65.70%	47.50%	0.35	3.61E-14
THEMIS2	IR-Mϕ	36.50%	17.30%	0.34	3.95E-14
GIMAP1	IR-Mϕ	23.80%	7.50%	0.32	4.64E-14
SYF2	IR-Mϕ	63.50%	48.90%	0.44	5.13E-14
NPL	IR-Mϕ	29.50%	11.60%	0.30	5.91E-14
HMGB1	IR-Mϕ	94.20%	91.10%	0.29	6.52E-14
OSTF1	IR-Mϕ	55.50%	38.20%	0.36	7.50E-14
FUCA1	IR-Mϕ	35.70%	16.60%	0.39	8.44E-14
TMBIM1	IR-Mϕ	55.90%	37.20%	0.37	9.07E-14
SRSF3	IR-Mϕ	78.60%	68.50%	0.34	1.23E-13
RCSD1	IR-Mϕ	38.40%	18.70%	0.32	1.29E-13
1-Mar	IR-Mϕ	38.20%	18.70%	0.33	1.30E-13
SELPLG	IR-Mϕ	32.10%	13.90%	0.34	1.39E-13
TRA2B	IR-Mϕ	76.70%	65.30%	0.42	1.96E-13
RGS10	IR-Mϕ	81.80%	72.10%	0.33	2.45E-13
YWHAH	IR-Mϕ	46.30%	26.70%	0.36	2.52E-13
HES1	IR-Mϕ	48.00%	27.90%	0.46	2.55E-13
C6orf1	IR-Mϕ	28.50%	11.40%	0.31	2.91E-13
TMEM219	IR-Mϕ	61.00%	44.00%	0.33	2.95E-13
NTAN1	IR-Mϕ	42.30%	23.00%	0.34	3.66E-13
PLA2G15	IR-Mϕ	25.60%	9.20%	0.31	4.18E-13
GATM	IR-Mϕ	36.70%	17.40%	0.32	4.85E-13
PTMS	IR-Mϕ	52.60%	34.20%	0.37	6.66E-13
ARHGAP30	IR-Mϕ	42.40%	23.20%	0.35	8.46E-13
TCN2	IR-Mϕ	42.30%	23.40%	0.38	8.62E-13
SESN1	IR-Mϕ	22.10%	7.00%	0.33	1.09E-12
BST2	IR-Mϕ	72.50%	61.50%	0.30	1.10E-12
FPR3	IR-Mϕ	64.20%	46.70%	0.38	1.13E-12
CNBP	IR-Mϕ	72.20%	59.50%	0.34	1.53E-12
GYPC	IR-Mϕ	54.70%	36.90%	0.39	1.57E-12
ZRSR2	IR-Mϕ	23.80%	8.10%	0.29	2.11E-12
ATP6V0E1	IR-Mϕ	85.10%	75.70%	0.29	2.68E-12
COMMD3	IR-Mϕ	38.90%	20.40%	0.32	3.32E-12
IFI30	IR-Mϕ	49.40%	29.70%	0.34	4.46E-12
HEXIM1	IR-Mϕ	67.60%	53.80%	0.42	7.75E-12
DSC2	IR-Mϕ	25.00%	9.20%	0.27	8.43E-12
CALR	IR-Mϕ	86.10%	81.30%	0.37	8.62E-12

FKBP5	IR-Mϕ	45.50%	27.50%	0.36	8.68E-12
NEU1	IR-Mϕ	63.00%	45.70%	0.31	9.04E-12
TCEB2	IR-Mϕ	85.60%	77.30%	0.27	9.77E-12
NDUFB1	IR-Mϕ	73.90%	65.00%	0.33	1.01E-11
RGL1	IR-Mϕ	33.60%	16.00%	0.33	1.03E-11
MERTK	IR-Mϕ	27.80%	11.20%	0.29	1.03E-11
PTPN18	IR-Mϕ	35.30%	17.80%	0.34	1.29E-11
GIMAP2	IR-Mϕ	20.00%	5.90%	0.27	1.40E-11
MYL12A	IR-Mϕ	91.30%	87.60%	0.25	1.56E-11
PPT1	IR-Mϕ	76.40%	64.10%	0.32	1.63E-11
EPHX1	IR-Mϕ	44.10%	26.20%	0.36	2.10E-11
SNCA	IR-Mϕ	43.10%	25.80%	0.36	2.24E-11
FCGR2B	IR-Mϕ	78.10%	63.20%	0.36	2.32E-11
HOXB7	IR-Mϕ	18.70%	5.20%	0.25	2.42E-11
ATP5G2	IR-Mϕ	85.40%	80.30%	0.27	2.97E-11
COX6A1	IR-Mϕ	83.00%	77.00%	0.30	3.37E-11
UQCRQ	IR-Mϕ	77.10%	69.30%	0.29	5.37E-11
A2M	IR-Mϕ	52.80%	35.00%	0.39	5.51E-11
TGFBR2	IR-Mϕ	40.10%	23.00%	0.32	5.84E-11
ITSN1	IR-Mϕ	23.80%	8.60%	0.28	6.75E-11
TPM3	IR-Mϕ	87.90%	82.20%	0.28	7.15E-11
CD59	IR-Mϕ	67.10%	54.10%	0.38	8.61E-11
GAA	IR-Mϕ	50.80%	33.50%	0.32	1.10E-10
SEC62	IR-Mϕ	77.60%	66.70%	0.30	1.37E-10
TMEM160	IR-Mϕ	45.00%	27.00%	0.30	1.49E-10
CRYL1	IR-Mϕ	39.40%	22.70%	0.34	1.54E-10
MNDA	IR-Mϕ	56.00%	38.80%	0.34	2.09E-10
CLEC12A	IR-Mϕ	40.70%	23.10%	0.33	2.65E-10
ARPC1B	IR-Mϕ	90.00%	86.70%	0.25	2.84E-10
ATP6V0B	IR-Mϕ	88.10%	84.90%	0.30	3.27E-10
IFI27L2	IR-Mϕ	64.90%	52.10%	0.34	3.60E-10
FXYD6	IR-Mϕ	31.90%	15.90%	0.37	5.37E-10
SAP18	IR-Mϕ	90.30%	87.40%	0.31	5.76E-10
PLXND1	IR-Mϕ	39.20%	22.20%	0.32	6.03E-10
TMEM176A	IR-Mϕ	44.80%	27.20%	0.37	6.21E-10
TMED9	IR-Mϕ	64.50%	51.00%	0.33	7.37E-10
FAM96A	IR-Mϕ	43.80%	27.00%	0.31	7.94E-10
RPS27L	IR-Mϕ	77.60%	68.10%	0.30	8.25E-10
CCR1	IR-Mϕ	33.60%	17.00%	0.31	8.49E-10
DYNC1I2	IR-Mϕ	55.30%	39.90%	0.34	8.65E-10
CIR1	IR-Mϕ	34.30%	17.50%	0.27	9.11E-10

CD4	IR-Mϕ	62.00%	45.50%	0.31	9.42E-10
CTSC	IR-Mϕ	52.10%	36.90%	0.36	1.31E-09
RNF13	IR-Mϕ	62.30%	47.50%	0.34	1.64E-09
FYB	IR-Mϕ	51.60%	35.00%	0.33	1.72E-09
TMEM179B	IR-Mϕ	45.50%	28.50%	0.31	1.75E-09
TMEM14C	IR-Mϕ	69.30%	60.10%	0.33	1.76E-09
SCP2	IR-Mϕ	64.00%	49.90%	0.28	1.77E-09
TTC7A	IR-Mϕ	28.40%	13.20%	0.28	1.82E-09
LAMTOR1	IR-Mϕ	54.50%	39.50%	0.31	2.18E-09
EVI2B	IR-Mϕ	60.10%	45.40%	0.33	2.25E-09
MYADM	IR-Mϕ	76.70%	63.50%	0.27	2.67E-09
RGS19	IR-Mϕ	40.10%	23.00%	0.29	2.72E-09
PEBP1	IR-Mϕ	71.30%	61.80%	0.30	3.19E-09
VAT1	IR-Mϕ	42.30%	25.80%	0.32	3.39E-09
RAB11A	IR-Mϕ	51.10%	35.20%	0.29	3.55E-09
SYNGR2	IR-Mϕ	54.20%	38.80%	0.30	3.56E-09
SLC9A9	IR-Mϕ	30.10%	14.20%	0.26	4.46E-09
DUSP23	IR-Mϕ	50.40%	34.80%	0.33	4.68E-09
COCH	IR-Mϕ	20.00%	7.10%	0.32	5.77E-09
TTC1	IR-Mϕ	38.90%	22.30%	0.27	5.88E-09
COLGALT1	IR-Mϕ	35.50%	20.10%	0.32	7.36E-09
NDUFB7	IR-Mϕ	66.20%	54.30%	0.34	7.77E-09
EVI2A	IR-Mϕ	50.30%	36.40%	0.36	1.02E-08
CSK	IR-Mϕ	26.80%	12.30%	0.27	1.04E-08
HEXB	IR-Mϕ	71.10%	63.10%	0.28	1.07E-08
ADAM9	IR-Mϕ	47.40%	31.10%	0.29	1.23E-08
BRI3	IR-Mϕ	45.00%	29.40%	0.33	1.24E-08
CRIP1	IR-Mϕ	87.90%	79.90%	0.28	1.26E-08
LAMTOR2	IR-Mϕ	58.10%	44.80%	0.31	1.35E-08
LSM4	IR-Mϕ	46.70%	31.20%	0.31	1.67E-08
HEBP1	IR-Mϕ	33.60%	18.70%	0.29	2.48E-08
ARL6IP4	IR-Mϕ	76.20%	71.70%	0.27	2.57E-08
ABCA6	IR-Mϕ	15.40%	4.50%	0.25	2.92E-08
PLXNB2	IR-Mϕ	38.70%	22.50%	0.30	3.07E-08
DPYSL3	IR-Mϕ	44.70%	29.50%	0.34	3.53E-08
NISCH	IR-Mϕ	30.40%	15.60%	0.29	3.56E-08
GAS7	IR-Mϕ	47.50%	31.70%	0.29	4.10E-08
STX7	IR-Mϕ	41.80%	26.60%	0.29	4.24E-08
SNX3	IR-Mϕ	71.00%	62.80%	0.25	4.52E-08
WASH4P	IR-Mϕ	35.80%	20.50%	0.27	5.32E-08
PPDPF	IR-Mϕ	80.10%	71.40%	0.26	6.52E-08

SLC7A8	IR-Mϕ	27.20%	13.00%	0.29	6.92E-08
NDUFA13	IR-Mϕ	70.60%	61.40%	0.31	7.57E-08
SLC7A7	IR-Mϕ	41.60%	26.10%	0.26	8.64E-08
FRMD4A	IR-Mϕ	22.20%	9.40%	0.26	8.76E-08
VPS28	IR-Mϕ	60.30%	49.00%	0.29	8.88E-08
PRDX5	IR-Mϕ	70.60%	61.90%	0.27	9.38E-08
CISD3	IR-Mϕ	32.60%	18.20%	0.30	9.78E-08
IDH1	IR-Mϕ	34.00%	18.70%	0.25	1.01E-07
NDUFA3	IR-Mϕ	44.70%	30.50%	0.33	1.02E-07
TM9SF2	IR-Mϕ	41.90%	28.00%	0.33	1.03E-07
AES	IR-Mϕ	47.40%	33.80%	0.32	1.15E-07
BLOC1S1	IR-Mϕ	67.10%	57.20%	0.28	1.15E-07
ST13	IR-Mϕ	72.20%	64.30%	0.28	1.23E-07
ISCU	IR-Mϕ	60.10%	45.70%	0.28	1.41E-07
SAMHD1	IR-Mϕ	58.60%	44.50%	0.30	1.57E-07
AP2A2	IR-Mϕ	46.50%	30.80%	0.30	1.57E-07
MT-ATP8	IR-Mϕ	42.10%	26.70%	0.29	1.59E-07
SSB	IR-Mϕ	52.50%	39.70%	0.29	1.60E-07
DYNLRB1	IR-Mϕ	64.30%	52.30%	0.27	1.74E-07
CCDC85B	IR-Mϕ	53.00%	38.60%	0.29	1.76E-07
PDIA6	IR-Mϕ	58.70%	47.40%	0.33	1.77E-07
RAB5C	IR-Mϕ	73.00%	63.70%	0.27	2.06E-07
SLC35F6	IR-Mϕ	24.30%	11.10%	0.25	2.22E-07
RP11-290F20.3	IR-Mϕ	33.40%	18.30%	0.29	2.36E-07
CNPY3	IR-Mϕ	43.50%	29.80%	0.30	2.53E-07
CLTA	IR-Mϕ	67.60%	56.50%	0.29	2.68E-07
NRP1	IR-Mϕ	38.50%	22.70%	0.27	2.79E-07
GSTT1	IR-Mϕ	28.20%	14.20%	0.26	2.97E-07
PDIA3	IR-Mϕ	79.30%	73.80%	0.29	3.10E-07
NUP214	IR-Mϕ	37.90%	23.90%	0.31	3.45E-07
ANAPC11	IR-Mϕ	64.20%	52.30%	0.27	3.66E-07
FCGBP	IR-Mϕ	14.80%	4.40%	0.32	3.68E-07
KLF9	IR-Mϕ	41.60%	27.10%	0.29	4.76E-07
APH1A	IR-Mϕ	50.80%	37.70%	0.28	5.57E-07
EID1	IR-Mϕ	68.90%	57.30%	0.27	6.40E-07
LFNG	IR-Mϕ	22.40%	9.90%	0.25	6.40E-07
FCGR2A	IR-Mϕ	83.20%	77.30%	0.29	6.72E-07
FCGR3A	IR-Mϕ	53.50%	39.60%	0.45	7.57E-07
MGST2	IR-Mϕ	36.50%	22.50%	0.28	8.45E-07
LINC00998	IR-Mϕ	42.60%	28.40%	0.26	9.59E-07
PPCS	IR-Mϕ	35.00%	21.30%	0.28	1.04E-06

PSME1	IR-Mϕ	74.40%	65.50%	0.27	1.06E-06
LRP1	IR-Mϕ	60.40%	47.20%	0.27	1.23E-06
AKR1B1	IR-Mϕ	55.90%	43.50%	0.29	1.28E-06
SUMF2	IR-Mϕ	41.10%	26.20%	0.26	1.30E-06
XIST	IR-Mϕ	69.90%	52.80%	0.29	1.35E-06
BANF1	IR-Mϕ	54.20%	42.10%	0.29	1.45E-06
BMP2K	IR-Mϕ	37.50%	23.10%	0.27	1.46E-06
LAIR1	IR-Mϕ	34.80%	20.40%	0.26	1.58E-06
VAMP3	IR-Mϕ	47.40%	34.80%	0.32	1.65E-06
TRMT1	IR-Mϕ	37.90%	23.70%	0.28	1.84E-06
TRAPPC1	IR-Mϕ	47.50%	35.10%	0.29	1.92E-06
ATP5C1	IR-Mϕ	63.30%	53.90%	0.26	1.98E-06
RAB31	IR-Mϕ	67.60%	58.50%	0.26	2.35E-06
CD302	IR-Mϕ	45.70%	32.40%	0.32	2.39E-06
GNB2	IR-Mϕ	58.70%	47.40%	0.26	2.39E-06
TREM2	IR-Mϕ	37.40%	22.80%	0.31	2.89E-06
KANK2	IR-Mϕ	25.60%	13.00%	0.26	3.12E-06
CMTM3	IR-Mϕ	37.50%	24.90%	0.34	3.17E-06
CITED2	IR-Mϕ	41.80%	27.60%	0.36	4.23E-06
DPYSL2	IR-Mϕ	65.00%	56.10%	0.28	4.29E-06
SNHG12	IR-Mϕ	41.80%	27.90%	0.34	4.73E-06
ZYX	IR-Mϕ	46.30%	32.40%	0.26	5.40E-06
WDR83OS	IR-Mϕ	65.00%	57.40%	0.27	5.78E-06
RFC1	IR-Mϕ	26.70%	14.30%	0.28	6.96E-06
LSM3	IR-Mϕ	52.30%	39.50%	0.26	8.06E-06
NBEAL1	IR-Mϕ	73.30%	70.50%	0.27	9.09E-06
CHURC1	IR-Mϕ	44.10%	31.20%	0.28	1.03E-05
WASF2	IR-Mϕ	59.60%	47.50%	0.27	1.05E-05
MGAT4A	IR-Mϕ	31.40%	18.10%	0.26	1.06E-05
UACA	IR-Mϕ	21.70%	10.50%	0.29	1.08E-05
TKT	IR-Mϕ	67.10%	57.40%	0.26	1.09E-05
PRR13	IR-Mϕ	56.50%	45.50%	0.27	1.10E-05
DBNL	IR-Mϕ	42.40%	29.20%	0.26	1.16E-05
CRTAP	IR-Mϕ	52.80%	40.50%	0.27	1.28E-05
CHMP3	IR-Mϕ	47.90%	35.50%	0.28	1.34E-05
BCAP31	IR-Mϕ	56.70%	46.10%	0.28	1.56E-05
GLUD1	IR-Mϕ	53.70%	42.60%	0.29	1.90E-05
NDUFA12	IR-Mϕ	57.40%	46.60%	0.27	2.09E-05
EPS15	IR-Mϕ	30.90%	17.90%	0.25	2.10E-05
VPS29	IR-Mϕ	63.30%	56.40%	0.27	2.14E-05
EVI5	IR-Mϕ	35.50%	21.90%	0.26	2.28E-05

HNRNPUL1	IR-Mϕ	46.90%	35.40%	0.30	2.38E-05
AKR1A1	IR-Mϕ	58.40%	48.10%	0.27	2.88E-05
SMIM4	IR-Mϕ	44.30%	31.70%	0.25	3.84E-05
CLTC	IR-Mϕ	50.40%	37.70%	0.26	4.55E-05
NUB1	IR-Mϕ	23.80%	12.40%	0.25	4.61E-05
SCAMP2	IR-Mϕ	40.60%	28.00%	0.27	5.00E-05
CBX3	IR-Mϕ	54.80%	43.00%	0.25	5.27E-05
WLS	IR-Mϕ	28.20%	16.10%	0.28	5.30E-05
CAPNS1	IR-Mϕ	51.30%	40.30%	0.27	1.03E-04
KIAA1598	IR-Mϕ	38.90%	26.20%	0.26	1.05E-04
EMC10	IR-Mϕ	43.60%	31.70%	0.25	1.09E-04
PTTG1IP	IR-Mϕ	53.30%	43.20%	0.28	1.10E-04
MTIF3	IR-Mϕ	34.80%	22.60%	0.27	1.10E-04
SLC20A1	IR-Mϕ	42.30%	29.30%	0.32	1.32E-04
TYMP	IR-Mϕ	54.50%	43.40%	0.30	1.50E-04
STOM	IR-Mϕ	44.10%	31.90%	0.27	1.64E-04
PDIA4	IR-Mϕ	36.70%	25.30%	0.28	2.39E-04
NCF2	IR-Mϕ	45.30%	33.50%	0.26	2.45E-04
CORO1B	IR-Mϕ	43.60%	32.30%	0.26	2.59E-04
ATP5F1	IR-Mϕ	55.70%	45.90%	0.26	2.81E-04
KRT10	IR-Mϕ	40.60%	28.60%	0.26	3.15E-04
NSA2	IR-Mϕ	54.80%	45.40%	0.27	3.64E-04
PPIG	IR-Mϕ	50.40%	40.30%	0.28	6.49E-04
CTSA	IR-Mϕ	49.10%	38.10%	0.27	7.75E-04
CPM	IR-Mϕ	50.60%	38.80%	0.25	8.12E-04
DRAM2	IR-Mϕ	42.60%	31.20%	0.25	1.09E-03
GADD45G	IR-Mϕ	30.70%	19.00%	0.29	1.19E-03
LARP7	IR-Mϕ	40.10%	29.00%	0.28	1.46E-03
SP100	IR-Mϕ	44.00%	33.00%	0.25	2.02E-03
C1orf63	IR-Mϕ	60.80%	52.30%	0.28	2.43E-03
PRMT2	IR-Mϕ	47.00%	37.50%	0.26	9.95E-03
HSPH1	IR-Mϕ	53.70%	44.50%	0.29	1.10E-02
TNFRSF1A	IR-Mϕ	45.00%	35.50%	0.26	1.62E-02
LY96	IR-Mϕ	56.00%	50.30%	0.26	3.01E-02
ADIRF	IR-Mϕ	59.30%	48.60%	0.28	3.65E-02
PHPT1	IR-Mϕ	53.30%	47.10%	0.25	6.54E-02
RN7SL1	IR-Mϕ	21.70%	13.70%	0.41	1.10E-01
ALDH2	IR-Mϕ	63.70%	58.10%	0.26	1.39E-01
CD36	IR-Mϕ	25.80%	18.60%	0.26	1.00E+00
HSPA6	IR-Mϕ	28.50%	24.10%	0.48	1.00E+00
CCL3	I-Mϕ	98.30%	70.30%	1.72	1.60E-83

SOD2	I-Mϕ	99.70%	79.70%	1.23	6.49E-83
CCL4	I-Mϕ	95.70%	60.00%	2.02	2.15E-82
IER3	I-Mϕ	100.00%	92.20%	0.87	1.70E-78
CXCL1	I-Mϕ	86.40%	42.90%	1.74	4.95E-74
CXCL3	I-Mϕ	99.40%	70.80%	1.09	1.68E-71
CXCL2	I-Mϕ	99.70%	68.30%	1.07	1.16E-69
CCL3L1	I-Mϕ	71.90%	23.70%	1.49	7.44E-69
FNIP2	I-Mϕ	75.90%	34.20%	0.90	1.97E-56
BCL2A1	I-Mϕ	84.60%	36.30%	0.93	1.90E-55
PHLDA1	I-Mϕ	82.30%	40.90%	0.93	1.15E-53
ICAM1	I-Mϕ	84.30%	48.20%	0.78	1.03E-51
IL1B	I-Mϕ	90.40%	52.20%	0.91	1.69E-46
PLAUR	I-Mϕ	96.50%	72.10%	0.80	2.49E-45
NFKB1	I-Mϕ	80.90%	42.90%	0.72	2.91E-45
S100A11	I-Mϕ	99.70%	99.30%	0.53	3.35E-45
TUBA1C	I-Mϕ	84.10%	57.70%	0.73	3.10E-44
THBD	I-Mϕ	69.60%	33.30%	0.93	5.47E-44
TNFAIP3	I-Mϕ	95.10%	63.70%	0.68	2.77E-43
RP11-701P16.5	I-Mϕ	46.10%	12.10%	1.01	3.52E-42
CD44	I-Mϕ	98.30%	90.90%	0.57	3.72E-42
PLEK	I-Mϕ	90.40%	59.50%	0.70	5.24E-42
IL8	I-Mϕ	99.70%	81.60%	0.62	1.44E-40
GLRX	I-Mϕ	87.00%	67.70%	0.83	6.78E-37
CCL2	I-Mϕ	85.80%	58.60%	1.28	7.18E-37
PIM3	I-Mϕ	67.50%	31.30%	0.59	3.42E-36
SPP1	I-Mϕ	33.00%	6.20%	2.00	7.59E-36
CTSL	I-Mϕ	86.10%	62.30%	0.78	2.19E-35
CFLAR	I-Mϕ	87.50%	59.90%	0.55	2.79E-34
ETS2	I-Mϕ	83.50%	53.20%	0.58	1.60E-32
TNF	I-Mϕ	79.40%	53.80%	1.16	3.23E-32
MMP9	I-Mϕ	45.20%	14.60%	0.82	6.60E-32
PDE4DIP	I-Mϕ	80.00%	52.00%	0.64	2.68E-31
PELI1	I-Mϕ	69.00%	37.80%	0.67	1.69E-30
TXN	I-Mϕ	98.60%	90.40%	0.59	3.13E-30
ABL2	I-Mϕ	84.30%	52.70%	0.54	8.93E-30
MARCKS	I-Mϕ	72.20%	42.50%	0.67	2.87E-29
MIR155HG	I-Mϕ	58.00%	23.00%	0.61	7.73E-29
IL1A	I-Mϕ	53.60%	21.90%	0.65	3.21E-28
CEBPB	I-Mϕ	81.20%	56.10%	0.69	8.99E-28
GPR183	I-Mϕ	92.80%	63.20%	0.50	1.59E-27
CCL20	I-Mϕ	34.50%	9.00%	1.21	7.20E-27

ATP13A3	I-Mϕ	60.60%	31.10%	0.55	2.07E-26
FTH1	I-Mϕ	100.00%	100.00%	0.42	5.29E-26
TNFAIP2	I-Mϕ	87.00%	65.20%	0.58	5.39E-26
WTAP	I-Mϕ	73.30%	45.80%	0.60	5.04E-25
C5AR1	I-Mϕ	73.60%	48.50%	0.58	7.72E-25
ZC3H12C	I-Mϕ	34.20%	9.90%	0.44	9.36E-25
IL1RN	I-Mϕ	49.00%	21.20%	1.05	1.63E-23
RBM47	I-Mϕ	68.10%	43.10%	0.55	2.63E-23
KANK1	I-Mϕ	27.50%	6.40%	0.45	6.09E-23
SERTAD2	I-Mϕ	58.80%	32.20%	0.54	1.38E-22
PLIN2	I-Mϕ	66.70%	41.20%	0.75	1.80E-22
CCRL2	I-Mϕ	40.00%	14.90%	0.53	1.88E-22
SDCBP	I-Mϕ	96.80%	87.50%	0.34	2.09E-22
IL6	I-Mϕ	44.90%	18.80%	0.92	3.45E-22
EIF4E	I-Mϕ	85.80%	58.70%	0.60	4.90E-22
DSE	I-Mϕ	67.20%	39.40%	0.49	7.95E-22
ARID5B	I-Mϕ	57.40%	28.90%	0.53	8.16E-22
CSTB	I-Mϕ	94.50%	88.70%	0.61	1.09E-21
ATP6V1F	I-Mϕ	88.10%	73.90%	0.42	1.32E-21
CYP1B1	I-Mϕ	73.90%	44.90%	0.57	9.84E-21
F3	I-Mϕ	28.10%	7.50%	0.48	9.84E-21
PLAU	I-Mϕ	65.50%	40.70%	0.67	3.00E-20
FTL	I-Mϕ	100.00%	100.00%	0.46	5.97E-20
CXCL5	I-Mϕ	27.80%	7.20%	0.67	6.86E-20
KLF6	I-Mϕ	98.30%	87.70%	0.39	7.78E-20
OTUD1	I-Mϕ	47.20%	22.10%	0.53	1.11E-19
ABCA1	I-Mϕ	52.80%	27.80%	0.50	3.40E-19
BIRC3	I-Mϕ	68.70%	38.80%	0.50	2.01E-18
PNRC1	I-Mϕ	90.70%	75.10%	0.39	3.75E-18
MAP2K3	I-Mϕ	82.90%	58.90%	0.37	6.76E-18
MFSD2A	I-Mϕ	44.90%	20.50%	0.40	8.53E-18
TNFAIP8	I-Mϕ	70.10%	43.10%	0.37	2.19E-17
NFE2L2	I-Mϕ	92.50%	70.60%	0.40	2.78E-17
GPR84	I-Mϕ	29.60%	9.40%	0.37	3.97E-17
RPS26	I-Mϕ	89.60%	83.70%	0.40	1.28E-16
SRGN	I-Mϕ	98.60%	91.60%	0.31	1.51E-16
SLC16A10	I-Mϕ	40.60%	18.40%	0.50	1.88E-16
INSIG1	I-Mϕ	87.50%	66.00%	0.35	2.17E-16
PTGER2	I-Mϕ	42.30%	18.60%	0.40	2.42E-16
KLHL21	I-Mϕ	44.60%	22.20%	0.45	6.50E-16
CDKN1A	I-Mϕ	94.20%	73.50%	0.39	9.70E-16

ACSL1	I-Mϕ	41.70%	19.30%	0.40	1.11E-15
CCL4L2	I-Mϕ	27.00%	8.40%	0.44	1.68E-15
NINJ1	I-Mϕ	78.30%	58.40%	0.44	6.08E-15
DOT1L	I-Mϕ	30.10%	11.10%	0.32	2.69E-14
MMP19	I-Mϕ	37.40%	16.20%	0.44	5.18E-14
NRP2	I-Mϕ	65.50%	42.10%	0.40	5.31E-14
EHD1	I-Mϕ	63.20%	40.10%	0.42	8.05E-14
TNFAIP6	I-Mϕ	47.20%	22.70%	0.28	9.95E-14
HIF1A	I-Mϕ	60.00%	37.10%	0.40	1.01E-13
RASGRP3	I-Mϕ	40.00%	19.30%	0.44	1.13E-13
ARL8B	I-Mϕ	58.60%	36.60%	0.39	1.66E-13
KMO	I-Mϕ	25.50%	8.30%	0.36	2.38E-13
KYNU	I-Mϕ	66.40%	42.90%	0.35	3.15E-13
LCP2	I-Mϕ	53.90%	31.90%	0.39	3.44E-13
MT-ATP6	I-Mϕ	100.00%	99.70%	0.26	4.29E-13
FAM107B	I-Mϕ	50.70%	28.10%	0.35	5.47E-13
HIVEP2	I-Mϕ	36.20%	16.50%	0.37	6.38E-13
MAFF	I-Mϕ	53.00%	30.50%	0.33	1.72E-12
PRDX1	I-Mϕ	94.80%	87.30%	0.31	3.23E-12
FNDC3B	I-Mϕ	46.10%	25.60%	0.36	3.75E-12
SDC4	I-Mϕ	32.20%	13.40%	0.29	6.51E-12
MESDC1	I-Mϕ	27.00%	10.30%	0.32	1.02E-11
CD109	I-Mϕ	34.20%	15.50%	0.35	1.11E-11
RASGEF1B	I-Mϕ	71.30%	48.60%	0.32	1.19E-11
NAMPT	I-Mϕ	73.90%	54.90%	0.36	1.20E-11
KIF1B	I-Mϕ	52.50%	31.80%	0.38	1.26E-11
CPEB4	I-Mϕ	63.20%	45.20%	0.45	1.26E-11
FABP5	I-Mϕ	74.20%	53.50%	0.39	1.65E-11
AQP9	I-Mϕ	13.60%	2.60%	0.32	1.82E-11
SLC16A6	I-Mϕ	17.40%	4.40%	0.26	2.32E-11
SAT1	I-Mϕ	99.40%	98.00%	0.35	2.40E-11
TPRA1	I-Mϕ	40.30%	21.00%	0.34	2.50E-11
ATP6V1B2	I-Mϕ	59.40%	40.30%	0.36	2.93E-11
LINC00152	I-Mϕ	52.80%	32.20%	0.58	3.31E-11
TANK	I-Mϕ	61.20%	40.30%	0.32	3.42E-11
POMP	I-Mϕ	84.90%	71.90%	0.32	5.87E-11
MAPK6	I-Mϕ	51.30%	32.70%	0.37	1.44E-10
FEM1B	I-Mϕ	28.70%	12.40%	0.40	1.68E-10
TLR2	I-Mϕ	39.70%	20.80%	0.34	5.37E-10
MFHAS1	I-Mϕ	30.40%	13.20%	0.28	5.48E-10
NUMB	I-Mϕ	48.70%	30.40%	0.39	5.78E-10

OLR1	I-Mϕ	47.20%	24.30%	0.29	6.68E-10
SMS	I-Mϕ	57.70%	41.20%	0.43	9.13E-10
ZC3H12A	I-Mϕ	61.70%	40.10%	0.26	1.03E-09
METRNL	I-Mϕ	48.70%	29.00%	0.33	1.26E-09
USP12	I-Mϕ	35.40%	17.30%	0.31	2.21E-09
MAP3K8	I-Mϕ	72.80%	54.80%	0.32	2.88E-09
SLC11A2	I-Mϕ	30.40%	13.70%	0.28	3.72E-09
DENND5A	I-Mϕ	43.50%	25.20%	0.33	3.91E-09
ELL2	I-Mϕ	46.10%	28.50%	0.38	3.93E-09
ATP6V1H	I-Mϕ	44.60%	25.90%	0.29	4.61E-09
B4GALT5	I-Mϕ	30.40%	14.10%	0.30	4.63E-09
SLC43A3	I-Mϕ	48.40%	30.80%	0.37	6.98E-09
TXNRD1	I-Mϕ	56.50%	34.90%	0.29	7.72E-09
PTPN1	I-Mϕ	51.00%	32.10%	0.39	8.45E-09
LUCAT1	I-Mϕ	53.00%	33.40%	0.39	1.08E-08
ZNF267	I-Mϕ	58.60%	38.00%	0.29	1.33E-08
RALGDS	I-Mϕ	47.80%	28.90%	0.30	1.54E-08
LIMS1	I-Mϕ	63.20%	49.20%	0.37	2.12E-08
CSGALNACT2	I-Mϕ	44.90%	27.50%	0.33	2.32E-08
LITAF	I-Mϕ	73.00%	60.90%	0.37	2.84E-08
TRAF1	I-Mϕ	43.20%	23.50%	0.41	3.34E-08
PTGS2	I-Mϕ	53.60%	35.70%	0.56	3.60E-08
TSC22D1	I-Mϕ	42.00%	23.90%	0.52	4.55E-08
MAPKAPK2	I-Mϕ	43.20%	26.70%	0.34	5.28E-08
DRAM1	I-Mϕ	30.10%	14.70%	0.29	5.71E-08
CLIC4	I-Mϕ	47.00%	31.40%	0.33	5.86E-08
KDM6B	I-Mϕ	81.20%	62.50%	0.27	5.88E-08
LPXN	I-Mϕ	53.00%	34.70%	0.29	8.60E-08
DUSP5	I-Mϕ	34.50%	18.10%	0.36	9.41E-08
MAFK	I-Mϕ	25.80%	11.50%	0.28	1.18E-07
ZBTB43	I-Mϕ	41.70%	24.20%	0.33	1.19E-07
STK40	I-Mϕ	32.50%	16.50%	0.28	1.23E-07
PRDM1	I-Mϕ	40.60%	23.20%	0.31	3.11E-07
IL10	I-Mϕ	44.60%	27.10%	0.59	3.60E-07
ABI1	I-Mϕ	49.60%	34.10%	0.29	5.38E-07
CDC42EP3	I-Mϕ	51.60%	33.80%	0.29	5.82E-07
ZBTB38	I-Mϕ	47.20%	30.80%	0.32	6.39E-07
BTG1	I-Mϕ	84.30%	72.10%	0.30	1.35E-06
TNIP3	I-Mϕ	17.40%	6.20%	0.55	1.47E-06
PIK3R5	I-Mϕ	37.70%	22.00%	0.27	1.85E-06
EPB41L3	I-Mϕ	47.80%	32.30%	0.28	1.91E-06

SNX10	I-Mϕ	48.70%	34.00%	0.41	2.41E-06
CLN8	I-Mϕ	46.10%	29.60%	0.27	2.48E-06
FOXO3	I-Mϕ	47.80%	31.30%	0.29	3.37E-06
SAV1	I-Mϕ	34.50%	19.70%	0.28	5.48E-06
BIRC2	I-Mϕ	47.80%	31.60%	0.25	5.63E-06
FOSL2	I-Mϕ	42.60%	26.20%	0.26	6.26E-06
LHFPL2	I-Mϕ	51.00%	35.00%	0.30	7.17E-06
PDGFB	I-Mϕ	39.10%	23.30%	0.28	8.74E-06
PDE4B	I-Mϕ	49.30%	31.30%	0.26	9.03E-06
CD93	I-Mϕ	21.40%	9.20%	0.27	1.05E-05
NOTCH2	I-Mϕ	41.40%	25.00%	0.25	1.31E-05
EREG	I-Mϕ	31.00%	16.00%	0.70	1.33E-05
TIPARP	I-Mϕ	62.00%	44.30%	0.26	1.78E-05
NSMAF	I-Mϕ	38.60%	23.20%	0.33	1.78E-05
MIR22HG	I-Mϕ	69.60%	52.30%	0.27	2.24E-05
GCLM	I-Mϕ	31.60%	17.50%	0.26	2.52E-05
SPHK1	I-Mϕ	30.70%	17.20%	0.26	1.01E-04
B3GNT5	I-Mϕ	41.40%	26.90%	0.27	1.27E-04
MMP14	I-Mϕ	47.00%	31.40%	0.26	1.51E-04
ICAM4	I-Mϕ	23.50%	11.80%	0.29	1.92E-04
SLC7A5	I-Mϕ	28.10%	15.20%	0.26	2.27E-04
RYBP	I-Mϕ	47.20%	32.60%	0.25	4.48E-04
TNIP1	I-Mϕ	44.90%	30.10%	0.26	1.19E-03
DNTTIP2	I-Mϕ	57.10%	42.90%	0.27	1.67E-03
BACH1	I-Mϕ	42.00%	29.10%	0.26	2.15E-03
SNAPC1	I-Mϕ	32.20%	20.50%	0.52	3.71E-03
NRIP1	I-Mϕ	33.90%	22.40%	0.28	4.43E-03
ANPEP	I-Mϕ	40.60%	29.00%	0.31	5.23E-03
BTG3	I-Mϕ	46.70%	35.40%	0.28	5.71E-03
QKI	I-Mϕ	59.70%	48.10%	0.27	6.74E-03
GNA13	I-Mϕ	38.60%	27.30%	0.25	2.06E-02
MIR29A	I-Mϕ	33.60%	22.70%	0.26	1.26E-01
THAP2	I-Mϕ	30.40%	21.00%	0.26	7.52E-01
CCL3L3	I-Mϕ	28.10%	19.80%	0.46	1.00E+00
CCL8	I-Mϕ	13.30%	7.00%	0.25	1.00E+00
CCL18	I-Mϕ	21.20%	13.70%	0.35	1.00E+00
RND3	I-Mϕ	31.00%	24.20%	0.30	1.00E+00
PFKFB3	I-Mϕ	38.60%	32.00%	0.26	1.00E+00
FCER1A	DC	86.90%	9.50%	2.45	6.79E-174
IL1R2	DC	74.10%	6.90%	1.72	2.13E-143
CD1C	DC	53.10%	1.50%	1.41	1.38E-119

CLEC10A	DC	80.30%	16.90%	1.36	1.52E-114
LGALS2	DC	60.60%	5.30%	1.07	6.43E-109
HLA-DPB1	DC	100.00%	90.50%	1.14	2.23E-97
AMICA1	DC	58.10%	6.20%	0.83	3.07E-96
HLA-DQA1	DC	96.60%	61.50%	1.43	1.73E-93
HLA-DQB1	DC	98.40%	75.40%	1.34	4.22E-93
HLA-DRA	DC	100.00%	96.80%	1.07	3.34E-91
HLA-DPA1	DC	100.00%	92.70%	1.09	2.77E-90
AGPAT9	DC	51.90%	5.00%	0.90	7.63E-87
CCR7	DC	47.80%	4.40%	1.60	5.75E-82
RHOF	DC	55.30%	8.40%	0.94	1.28E-77
CD48	DC	51.20%	7.20%	0.73	8.02E-74
G0S2	DC	65.60%	16.90%	2.13	1.49E-72
EIF1	DC	100.00%	99.40%	0.58	1.76E-69
SRGN	DC	98.80%	91.70%	1.04	2.56E-69
HLA-DRB5	DC	99.70%	91.00%	0.99	7.90E-69
REL	DC	93.80%	66.00%	1.07	7.66E-68
CFP	DC	55.60%	10.60%	0.70	6.00E-64
C15orf48	DC	67.50%	21.80%	1.51	3.49E-62
CCL22	DC	32.20%	1.40%	1.21	8.60E-62
RILPL2	DC	70.60%	24.70%	0.87	7.66E-61
RPL3	DC	100.00%	99.60%	0.50	6.00E-60
CYTIP	DC	73.80%	29.30%	0.97	7.67E-59
RPS2	DC	100.00%	99.70%	0.51	5.03E-56
CD1E	DC	25.90%	0.40%	0.49	5.78E-56
GPR183	DC	92.80%	63.90%	1.00	2.41E-55
STX11	DC	74.10%	34.90%	0.90	1.66E-53
RPL18A	DC	100.00%	99.60%	0.44	4.96E-53
CST7	DC	26.60%	0.90%	0.60	1.84E-52
HLA-DRB1	DC	100.00%	94.40%	0.69	5.05E-52
SERPINB9	DC	74.40%	34.00%	1.01	1.09E-51
LSP1	DC	78.10%	33.70%	0.81	4.87E-51
RPS27	DC	100.00%	100.00%	0.40	7.13E-51
RPL10	DC	100.00%	100.00%	0.37	3.09E-50
TRAF1	DC	60.90%	18.50%	0.81	1.59E-49
EEF1A1	DC	100.00%	100.00%	0.35	7.91E-49
RP6-91H8.3	DC	28.80%	1.90%	0.48	4.39E-48
PHACTR1	DC	63.10%	20.90%	0.80	5.06E-48
HLA-DQA2	DC	77.20%	38.00%	1.25	5.65E-48
PTGIR	DC	40.90%	6.90%	0.64	6.08E-48
RPS9	DC	100.00%	99.90%	0.37	6.22E-48

RPS19	DC	99.70%	99.70%	0.45	1.32E-47
PABPC1	DC	99.10%	96.50%	0.52	3.48E-47
IL8	DC	94.10%	83.80%	1.06	2.41E-46
LIMD2	DC	29.10%	2.40%	0.40	3.76E-46
RPL11	DC	100.00%	99.80%	0.39	8.66E-46
STK4	DC	67.80%	29.00%	0.79	2.48E-45
PLAUR	DC	94.70%	73.30%	0.77	1.45E-44
RPS3	DC	100.00%	99.40%	0.41	5.06E-44
RPS18	DC	100.00%	99.90%	0.46	6.45E-44
PDE4B	DC	67.50%	26.10%	0.66	1.23E-43
MARCKSL1	DC	46.90%	10.90%	0.61	2.79E-43
TNFAIP8	DC	77.50%	41.50%	0.95	1.05E-42
INSIG1	DC	88.10%	66.40%	0.96	1.10E-42
RPL28	DC	100.00%	99.70%	0.39	4.11E-42
DUSP4	DC	49.70%	14.90%	1.01	5.61E-41
CXCR4	DC	78.10%	42.00%	1.00	7.02E-41
RPS7	DC	99.70%	98.80%	0.44	4.94E-40
CD52	DC	36.90%	6.70%	0.66	8.28E-40
PLD4	DC	23.80%	1.50%	0.37	1.76E-39
BIRC3	DC	75.00%	37.50%	0.84	2.28E-39
CD74	DC	100.00%	99.20%	0.50	4.03E-39
RPL6	DC	100.00%	99.10%	0.40	4.04E-39
RPS3A	DC	99.70%	99.90%	0.38	2.77E-38
ETV3	DC	54.10%	18.30%	0.57	6.96E-38
RPL4	DC	99.40%	93.70%	0.49	2.51E-37
RPS16	DC	99.70%	99.70%	0.36	1.58E-36
RPL41	DC	100.00%	100.00%	0.30	1.63E-36
TMEM120B	DC	29.10%	4.00%	0.46	4.89E-36
RPL13A	DC	100.00%	100.00%	0.31	1.30E-35
RPLP0	DC	99.40%	96.00%	0.42	1.32E-35
CSF2RA	DC	65.90%	30.90%	0.68	1.38E-35
RPS6	DC	100.00%	100.00%	0.36	1.93E-35
CD55	DC	92.20%	75.30%	0.62	2.85E-35
PKIB	DC	33.40%	6.30%	0.50	7.84E-35
H3F3A	DC	99.10%	97.10%	0.45	1.18E-34
RPL34	DC	99.70%	100.00%	0.37	2.47E-34
IL4I1	DC	28.40%	4.10%	0.56	2.49E-34
SLC7A11	DC	32.20%	5.70%	0.71	4.18E-34
IL1B	DC	85.00%	54.80%	1.09	4.54E-34
RPL26	DC	99.70%	99.80%	0.33	5.73E-34
DDX5	DC	99.10%	96.00%	0.52	2.23E-33

RPL37	DC	100.00%	99.70%	0.34	2.39E-33
SRSF2	DC	89.40%	68.00%	0.57	3.52E-33
RGS1	DC	91.90%	70.00%	0.77	3.89E-33
HLA-A	DC	99.10%	94.50%	0.47	6.68E-33
RPS13	DC	99.70%	99.80%	0.33	1.53E-31
H3F3B	DC	100.00%	98.80%	0.47	2.06E-31
CREM	DC	65.00%	36.50%	0.94	1.58E-30
EZR	DC	85.90%	62.60%	0.66	1.65E-30
ADAM8	DC	35.30%	8.20%	0.45	5.06E-30
RPL13	DC	100.00%	100.00%	0.29	8.47E-30
MIR29A	DC	50.00%	17.90%	0.51	1.63E-29
ADAM19	DC	22.80%	2.70%	0.43	2.44E-29
RPS8	DC	99.40%	99.80%	0.36	2.59E-29
MIR181A1HG	DC	25.60%	3.70%	0.36	3.00E-29
CCND2	DC	37.20%	9.90%	0.46	3.78E-29
RPS5	DC	99.40%	98.10%	0.34	4.80E-29
CYCS	DC	88.40%	65.00%	0.58	7.87E-29
RPL18	DC	99.70%	99.00%	0.33	9.87E-29
RPL39	DC	99.70%	99.70%	0.30	1.46E-28
RALA	DC	55.00%	23.40%	0.65	2.05E-28
ANKRD28	DC	73.40%	45.10%	0.74	2.31E-28
RP11-138A9.2	DC	32.50%	7.20%	0.44	3.07E-28
NFKBID	DC	66.90%	32.90%	0.53	5.10E-28
RPL10A	DC	99.70%	98.90%	0.35	6.61E-28
AREG	DC	22.80%	2.90%	0.36	7.94E-28
CXCR3	DC	18.10%	1.30%	0.39	8.46E-28
SAT1	DC	98.80%	98.30%	0.61	1.22E-27
RPS24	DC	100.00%	99.90%	0.30	1.38E-27
ICAM3	DC	24.40%	3.70%	0.37	4.61E-27
CD69	DC	26.60%	4.60%	0.42	5.09E-27
IL7R	DC	29.10%	5.90%	0.61	7.23E-27
BCL2A1	DC	72.50%	41.20%	0.84	7.36E-27
ISG20	DC	45.30%	16.10%	0.59	1.31E-26
NLRP3	DC	57.50%	25.50%	0.64	2.47E-26
AKAP13	DC	85.30%	64.90%	0.54	3.31E-26
HLA-DQB2	DC	20.00%	2.20%	0.29	3.85E-26
RPS23	DC	99.40%	100.00%	0.31	3.88E-26
DAPP1	DC	31.20%	7.30%	0.46	6.55E-26
GPBP1	DC	76.20%	50.30%	0.53	7.65E-26
PMAIP1	DC	73.40%	42.40%	0.60	1.30E-25
BAZ1A	DC	82.80%	57.90%	0.56	1.37E-25

EREG	DC	41.20%	13.20%	0.82	1.74E-25
PTPRE	DC	70.30%	39.10%	0.60	2.20E-25
RGS2	DC	75.60%	50.10%	1.04	2.26E-25
CPVL	DC	86.20%	64.40%	0.65	6.06E-25
CD86	DC	64.40%	33.50%	0.55	6.53E-25
TXNRD1	DC	61.90%	33.80%	0.71	6.59E-25
SLC38A1	DC	19.10%	2.10%	0.26	7.16E-25
PPIF	DC	55.90%	25.20%	0.62	7.85E-25
RPL30	DC	99.70%	99.50%	0.29	1.15E-24
RPS15A	DC	100.00%	99.90%	0.28	1.34E-24
OLR1	DC	51.90%	23.50%	0.77	3.07E-24
RPL5	DC	99.40%	98.60%	0.30	3.22E-24
RPL7	DC	99.70%	99.50%	0.29	4.62E-24
RPL21	DC	100.00%	100.00%	0.26	7.14E-24
MAP3K8	DC	77.50%	53.70%	0.53	7.81E-24
CDKN1A	DC	93.80%	74.10%	0.46	2.37E-23
MCOLN2	DC	29.70%	7.10%	0.36	4.05E-23
PRMT10	DC	26.20%	5.50%	0.50	4.15E-23
TES	DC	53.10%	24.80%	0.54	5.09E-23
HLA-DMA	DC	96.60%	86.10%	0.40	5.77E-23
SNHG15	DC	47.80%	19.80%	0.52	1.03E-22
IL2RG	DC	22.80%	3.90%	0.28	2.41E-22
BTG1	DC	86.20%	71.80%	0.73	9.63E-22
RPS4X	DC	99.70%	99.60%	0.28	1.18E-21
RPL9	DC	99.70%	99.70%	0.27	1.80E-21
PPP1R15A	DC	98.40%	88.60%	0.65	2.02E-21
KYNU	DC	68.10%	42.90%	0.63	2.84E-21
HMGA1	DC	59.70%	31.10%	0.46	2.91E-21
FAU	DC	100.00%	99.80%	0.25	3.04E-21
BID	DC	64.10%	36.40%	0.54	3.47E-21
LUCAT1	DC	60.90%	31.40%	0.52	3.80E-21
RPL32	DC	99.70%	99.90%	0.26	4.74E-21
MIR142	DC	36.20%	11.90%	0.42	7.56E-21
LINC00936	DC	56.60%	30.00%	0.68	8.01E-21
SAMSN1	DC	42.50%	17.50%	0.74	1.36E-20
RPL23A	DC	99.70%	99.40%	0.29	2.16E-20
RPSA	DC	98.40%	94.90%	0.33	3.49E-20
GNB2L1	DC	98.80%	97.40%	0.30	4.09E-20
EEF2	DC	95.90%	89.10%	0.35	4.26E-20
NFKB2	DC	54.70%	26.00%	0.42	5.23E-20
H2AFZ	DC	93.40%	85.50%	0.56	9.16E-20

ADAM28	DC	22.20%	4.20%	0.31	1.06E-19
DDIT4	DC	70.60%	46.90%	0.63	1.61E-19
SLC25A6	DC	95.00%	87.80%	0.40	1.74E-19
RUNX3	DC	33.10%	10.30%	0.38	1.89E-19
CD83	DC	95.00%	80.90%	0.59	1.99E-19
HLA-B	DC	99.70%	99.00%	0.28	2.93E-19
NFAT5	DC	57.50%	28.30%	0.38	2.94E-19
C12orf75	DC	16.90%	2.30%	0.28	5.45E-19
MIR22HG	DC	77.20%	50.40%	0.46	6.10E-19
IL23A	DC	21.60%	4.20%	0.39	6.91E-19
FAM49A	DC	50.60%	24.10%	0.45	7.01E-19
SIPA1L1	DC	46.20%	19.70%	0.40	8.36E-19
CXCL16	DC	73.40%	48.80%	0.52	1.07E-18
VEGFA	DC	48.40%	21.50%	0.46	1.64E-18
RP11-58E21.3	DC	19.40%	3.40%	0.28	2.07E-18
AC016831.7	DC	24.70%	5.80%	0.31	2.53E-18
RPL35A	DC	99.40%	99.80%	0.27	2.55E-18
RPL15	DC	100.00%	100.00%	0.25	2.71E-18
TMEM123	DC	78.80%	56.40%	0.48	3.69E-18
MTHFD2	DC	57.50%	32.30%	0.51	5.14E-18
HCST	DC	80.00%	53.30%	0.42	6.26E-18
EEF1B2	DC	97.20%	93.00%	0.33	8.99E-18
RPL31	DC	99.70%	99.60%	0.26	1.51E-17
ID2	DC	91.90%	82.40%	0.56	1.97E-17
EIF4A1	DC	99.40%	95.60%	0.34	2.02E-17
NAP1L1	DC	91.90%	76.70%	0.45	3.13E-17
PTGER4	DC	40.90%	16.30%	0.44	3.20E-17
PID1	DC	39.70%	15.60%	0.42	4.34E-17
MAP2K3	DC	80.60%	60.20%	0.45	6.21E-17
NAMPT	DC	75.90%	54.70%	0.46	6.36E-17
LST1	DC	89.70%	73.20%	0.35	9.05E-17
LITAF	DC	80.00%	59.10%	0.44	1.64E-16
NPM1	DC	96.60%	89.50%	0.40	1.76E-16
BASP1	DC	41.90%	18.10%	0.46	2.02E-16
TCHH	DC	31.20%	10.50%	0.59	2.48E-16
RPL36A	DC	98.40%	92.50%	0.30	4.08E-16
TRAF4	DC	21.60%	4.90%	0.27	4.40E-16
FNBP1	DC	60.90%	34.60%	0.43	6.59E-16
THAP2	DC	42.50%	17.50%	0.38	9.03E-16
EHD1	DC	64.40%	40.30%	0.50	9.52E-16
ATF4	DC	94.10%	82.80%	0.36	9.63E-16

NFKBIA	DC	97.80%	93.10%	0.48	2.85E-15
NFE2L2	DC	85.90%	73.10%	0.39	3.15E-15
LPXN	DC	58.10%	33.60%	0.41	4.64E-15
RASSF5	DC	35.00%	13.60%	0.35	4.95E-15
EIF3L	DC	74.10%	55.20%	0.40	6.33E-15
RPS10	DC	97.50%	96.30%	0.28	6.41E-15
ARL4C	DC	48.10%	23.00%	0.47	9.18E-15
TACSTD2	DC	10.90%	0.90%	0.45	9.76E-15
NR4A3	DC	45.30%	21.30%	0.43	1.02E-14
TNFRSF1B	DC	59.10%	34.80%	0.46	1.03E-14
TGIF1	DC	59.10%	35.90%	0.44	1.29E-14
ETF1	DC	67.20%	42.00%	0.38	1.69E-14
RP11-138A9.1	DC	37.50%	16.00%	0.38	1.83E-14
CFLAR	DC	83.40%	61.80%	0.41	2.46E-14
SDCBP	DC	93.40%	88.80%	0.45	3.33E-14
HNRNPA0	DC	75.90%	58.20%	0.40	6.28E-14
MIR155HG	DC	49.40%	26.40%	0.68	7.05E-14
MXD1	DC	42.50%	20.60%	0.56	7.23E-14
SKIL	DC	68.10%	46.60%	0.44	9.67E-14
IFRD1	DC	65.60%	41.70%	0.46	1.11E-13
SPAG9	DC	70.60%	50.40%	0.42	1.37E-13
ATP2B1	DC	83.40%	68.30%	0.43	1.37E-13
CHD2	DC	56.60%	34.10%	0.43	2.16E-13
SERPINB1	DC	70.30%	52.20%	0.68	5.03E-13
ATP1B1	DC	44.10%	21.50%	0.44	6.24E-13
BTF3	DC	97.20%	92.60%	0.26	6.64E-13
DNTTIP2	DC	63.10%	41.40%	0.38	9.49E-13
SLC25A5	DC	85.00%	73.20%	0.36	1.02E-12
SYAP1	DC	55.00%	32.60%	0.41	1.92E-12
C10orf128	DC	27.80%	9.70%	0.29	2.11E-12
TIPARP	DC	66.90%	43.20%	0.38	2.18E-12
SLC2A3	DC	43.10%	20.80%	0.38	2.34E-12
NFKBIZ	DC	75.90%	55.90%	0.41	3.30E-12
HNRNPC	DC	91.20%	79.10%	0.30	3.46E-12
UBE2D3	DC	90.30%	77.60%	0.32	3.76E-12
FCN1	DC	16.20%	3.40%	0.35	3.89E-12
GK	DC	29.70%	11.20%	0.31	5.18E-12
SQSTM1	DC	94.70%	84.10%	0.40	8.29E-12
ATP1B3	DC	77.20%	62.00%	0.52	9.47E-12
CD58	DC	58.40%	38.20%	0.37	1.15E-11
SLC3A2	DC	76.60%	67.90%	0.55	1.64E-11

REV3L	DC	42.80%	21.60%	0.39	1.76E-11
TNIP1	DC	50.60%	28.70%	0.40	1.81E-11
JARID2	DC	35.00%	15.00%	0.33	2.22E-11
ZNF331	DC	36.20%	16.70%	0.58	2.44E-11
RBM8A	DC	83.40%	66.70%	0.33	2.54E-11
NDRG2	DC	29.10%	10.90%	0.26	3.17E-11
CHMP1B	DC	70.30%	54.00%	0.53	3.25E-11
ZC3H12A	DC	61.90%	40.60%	0.42	5.06E-11
PELO	DC	25.00%	8.70%	0.28	5.52E-11
CDC42EP3	DC	54.40%	33.30%	0.44	7.94E-11
IL10	DC	47.20%	26.70%	0.59	1.09E-10
ACSL5	DC	34.70%	14.80%	0.26	1.12E-10
SLC1A5	DC	40.30%	20.80%	0.35	1.45E-10
JUNB	DC	99.10%	95.20%	0.50	1.49E-10
PHLDA2	DC	67.20%	51.30%	0.68	1.55E-10
SERPINA1	DC	30.30%	12.00%	0.26	1.81E-10
NEDD9	DC	30.30%	12.50%	0.30	2.16E-10
CASC7	DC	31.90%	13.60%	0.28	2.46E-10
SDPR	DC	17.20%	4.30%	0.27	2.62E-10
CXCL3	DC	87.20%	75.20%	0.64	2.67E-10
VASP	DC	76.20%	58.10%	0.35	3.27E-10
BZW1	DC	73.80%	53.00%	0.32	3.93E-10
TCOF1	DC	35.90%	17.50%	0.50	5.41E-10
LINC00152	DC	54.70%	32.10%	0.29	6.09E-10
DNAJB6	DC	82.80%	67.40%	0.40	6.43E-10
MAFF	DC	52.50%	31.20%	0.39	7.92E-10
FOSL2	DC	46.90%	25.30%	0.32	1.13E-09
MCL1	DC	90.60%	79.20%	0.38	1.15E-09
IRF7	DC	33.80%	15.60%	0.36	1.39E-09
EIF3F	DC	76.90%	57.00%	0.31	1.44E-09
RAB8B	DC	41.60%	21.00%	0.29	2.01E-09
LYZ	DC	96.60%	90.00%	0.49	2.07E-09
MIR24-2	DC	45.30%	24.00%	0.34	2.17E-09
NFKB1	DC	68.10%	47.70%	0.39	2.34E-09
GLTSCR2	DC	86.90%	72.10%	0.28	2.38E-09
KDM6B	DC	83.10%	62.40%	0.30	2.60E-09
PLEK	DC	77.80%	64.10%	0.38	2.67E-09
GLA	DC	52.50%	32.30%	0.39	2.70E-09
SUB1	DC	82.50%	69.60%	0.33	3.07E-09
RNF24	DC	29.10%	12.60%	0.34	3.73E-09
PVRL2	DC	40.30%	20.70%	0.29	3.85E-09

ZFP36	DC	94.10%	89.20%	0.58	5.24E-09
GNA15	DC	52.50%	32.90%	0.35	5.75E-09
CMTM6	DC	74.40%	57.80%	0.38	7.08E-09
ARL8B	DC	58.10%	37.30%	0.33	1.53E-08
ARL5B	DC	36.90%	19.10%	0.39	1.98E-08
ATP1A1	DC	76.90%	60.80%	0.34	2.20E-08
CA2	DC	20.90%	7.40%	0.32	2.96E-08
KCNN4	DC	34.10%	16.30%	0.27	3.29E-08
HNRNPA2B1	DC	97.80%	92.00%	0.29	4.01E-08
TUBB4B	DC	86.20%	78.20%	0.42	5.02E-08
CSRNP1	DC	63.40%	42.20%	0.34	5.15E-08
XBP1	DC	66.20%	47.60%	0.32	7.74E-08
SETD5-AS1	DC	49.40%	29.60%	0.32	9.17E-08
PLEKHF2	DC	27.20%	11.80%	0.27	1.05E-07
TARS	DC	35.00%	18.80%	0.34	1.11E-07
SPHK1	DC	34.10%	16.50%	0.25	1.12E-07
EIF4E	DC	77.80%	61.80%	0.32	1.96E-07
TET2	DC	41.60%	21.90%	0.26	2.13E-07
EIF5	DC	86.60%	74.70%	0.31	2.75E-07
IL10RA	DC	54.40%	34.40%	0.28	2.82E-07
RBM23	DC	37.80%	20.00%	0.25	3.10E-07
MBP	DC	53.40%	35.30%	0.33	3.47E-07
LMNA	DC	97.50%	96.90%	0.34	4.53E-07
MAFG	DC	49.40%	30.00%	0.34	4.87E-07
PIM3	DC	56.20%	35.60%	0.33	6.37E-07
AKIRIN2	DC	44.40%	26.90%	0.34	6.66E-07
RHEB	DC	72.50%	57.40%	0.28	1.19E-06
SFR1	DC	27.50%	13.10%	0.33	1.67E-06
PTGS2	DC	53.80%	36.10%	0.39	1.96E-06
RBM39	DC	83.10%	70.40%	0.28	2.10E-06
CLEC5A	DC	31.60%	15.70%	0.31	2.36E-06
USP53	DC	48.80%	31.20%	0.30	2.93E-06
IL1A	DC	43.80%	25.70%	0.38	3.56E-06
CORO1A	DC	66.90%	48.50%	0.26	3.72E-06
TANK	DC	60.00%	41.20%	0.26	3.86E-06
TOP1	DC	57.20%	39.80%	0.29	4.45E-06
ZBTB43	DC	41.90%	24.60%	0.27	4.94E-06
AXL	DC	36.20%	19.60%	0.30	5.14E-06
CD40	DC	37.50%	21.80%	0.37	5.47E-06
GTF2B	DC	51.90%	33.00%	0.27	6.71E-06
PPP1R15B	DC	47.80%	30.20%	0.27	6.73E-06

EIF4A3	DC	71.90%	60.80%	0.49	1.02E-05
MYO1G	DC	41.20%	24.50%	0.26	1.41E-05
HCAR2	DC	23.10%	10.40%	0.32	2.07E-05
PTTG1	DC	33.10%	17.90%	0.29	2.13E-05
SOCS3	DC	66.90%	48.90%	0.34	2.17E-05
VMP1	DC	62.20%	45.10%	0.33	2.23E-05
CKLF	DC	56.90%	40.80%	0.32	3.34E-05
DNAJB4	DC	34.10%	18.70%	0.29	3.56E-05
ARID4B	DC	56.60%	40.10%	0.29	3.77E-05
LPAR6	DC	37.20%	22.00%	0.39	5.41E-05
SRSF5	DC	83.80%	73.60%	0.31	6.76E-05
UBE2S	DC	49.70%	32.60%	0.26	6.98E-05
SOX4	DC	37.20%	21.40%	0.40	8.20E-05
OGFRL1	DC	43.40%	26.50%	0.25	8.37E-05
JMJD1C	DC	66.20%	51.60%	0.37	9.84E-05
HSPA8	DC	92.50%	89.80%	0.37	1.05E-04
CLEC7A	DC	65.00%	47.50%	0.28	1.24E-04
IRF8	DC	62.50%	48.90%	0.45	1.24E-04
AHR	DC	66.60%	51.50%	0.28	1.28E-04
CTA-29F11.1	DC	32.20%	18.00%	0.26	1.45E-04
CSNK1D	DC	42.50%	26.70%	0.26	1.77E-04
KPNA2	DC	50.90%	34.00%	0.29	1.82E-04
ZNF267	DC	55.00%	39.60%	0.29	2.98E-04
SFPQ	DC	77.20%	65.70%	0.28	4.40E-04
CXCL2	DC	84.10%	73.90%	0.38	5.45E-04
ATP13A3	DC	51.60%	34.60%	0.26	8.03E-04
BTG2	DC	58.80%	43.30%	0.31	1.01E-03
DNAJA1	DC	89.40%	82.00%	0.40	1.05E-03
CHD1	DC	48.80%	33.50%	0.25	1.45E-03
CCNL1	DC	77.80%	64.20%	0.28	1.52E-03
PNRC1	DC	88.10%	76.20%	0.33	1.87E-03
PPP1CB	DC	64.40%	50.60%	0.27	2.28E-03
DDX3X	DC	81.20%	73.80%	0.27	4.29E-03
PTGER2	DC	34.70%	21.60%	0.30	4.82E-03
CLK1	DC	65.00%	53.10%	0.30	5.04E-03
YPEL5	DC	59.40%	48.20%	0.37	6.74E-03
TIMP1	DC	90.30%	82.00%	0.34	1.81E-02
RP11-386I14.4	DC	26.90%	15.50%	0.32	1.85E-02
B4GALT1	DC	45.00%	32.60%	0.37	1.94E-02
MAP1LC3B	DC	81.60%	72.60%	0.29	2.18E-02
YME1L1	DC	57.20%	44.90%	0.29	3.16E-02

PRDM1	DC	37.50%	24.50%	0.26	4.55E-02
ZFAND2A	DC	34.40%	22.20%	0.27	5.96E-02
FABP5	DC	68.40%	55.70%	0.33	7.29E-02
RAB11FIP1	DC	35.00%	23.50%	0.27	1.11E-01
ITGA5	DC	43.40%	32.20%	0.25	1.12E-01
RIT1	DC	48.80%	36.00%	0.30	1.14E-01
BHLHE40	DC	59.70%	47.60%	0.29	1.74E-01
RAB9A	DC	29.10%	19.00%	0.39	1.88E-01
RNASET2	DC	73.10%	67.70%	0.27	2.02E-01
FILIP1L	DC	60.30%	50.80%	0.33	6.64E-01
CKS2	DC	52.50%	46.20%	0.41	8.59E-01
RGCC	DC	62.80%	53.40%	0.26	1.00E+00
ATF3	DC	71.60%	61.00%	0.38	1.00E+00
WSB1	DC	72.50%	67.10%	0.27	1.00E+00
ILF3-AS1	DC	18.40%	11.10%	0.27	1.00E+00
VMO1	DC	40.30%	30.30%	0.26	1.00E+00
NR4A2	DC	73.40%	66.50%	0.43	1.00E+00
NDUFV2	DC	56.90%	51.00%	0.27	1.00E+00
TSHZ2	iFIB	59.80%	1.60%	0.70	1.97E-120
SSPN	iFIB	60.80%	1.90%	0.70	3.41E-117
MEG3	iFIB	70.10%	3.90%	1.22	7.87E-110
CALD1	iFIB	90.70%	10.30%	1.43	8.58E-107
COL14A1	iFIB	62.90%	3.10%	1.25	3.76E-105
PRRX1	iFIB	68.00%	3.80%	0.83	5.58E-104
CNN3	iFIB	71.10%	4.70%	0.82	1.54E-101
MFAP4	iFIB	73.20%	5.30%	1.04	2.27E-100
ANGPTL2	iFIB	91.80%	12.20%	1.52	6.86E-98
NFIB	iFIB	50.50%	1.50%	0.59	6.22E-97
EBF1	iFIB	54.60%	2.10%	0.53	1.63E-95
MEDAG	iFIB	60.80%	3.20%	0.68	1.12E-93
LAMA4	iFIB	54.60%	2.40%	0.55	7.03E-91
ISLR	iFIB	80.40%	8.40%	1.18	1.06E-88
TPD52L1	iFIB	52.60%	2.20%	0.55	1.40E-88
LHFP	iFIB	74.20%	6.50%	0.85	3.64E-88
MFGE8	iFIB	74.20%	6.80%	1.11	1.74E-87
FKBP10	iFIB	63.90%	4.30%	0.66	3.10E-87
RP11-572C15.6	iFIB	48.50%	1.80%	0.54	3.54E-85
MAP1A	iFIB	69.10%	5.50%	0.73	1.16E-84
CCDC80	iFIB	89.70%	14.40%	1.60	1.01E-82
C1R	iFIB	91.80%	16.10%	1.88	2.21E-82
LIMA1	iFIB	84.50%	11.00%	1.04	3.08E-82

MXRA8	iFIB	66.00%	5.20%	0.87	3.43E-82
CAV1	iFIB	81.40%	10.20%	1.27	3.54E-82
ANGPTL1	iFIB	50.50%	2.30%	0.59	4.78E-82
PCOLCE	iFIB	91.80%	16.40%	1.68	1.15E-81
GLT8D2	iFIB	45.40%	1.60%	0.44	3.23E-81
FOXC1	iFIB	48.50%	2.10%	0.51	9.17E-81
C1QTNF3	iFIB	52.60%	2.80%	0.76	1.42E-80
RERG	iFIB	53.60%	2.90%	0.60	2.55E-80
ECM2	iFIB	48.50%	2.10%	0.61	2.69E-80
MT1M	iFIB	70.10%	6.60%	1.12	4.05E-80
EFEMP2	iFIB	51.50%	2.60%	0.54	6.94E-80
COL6A2	iFIB	93.80%	19.90%	1.92	8.80E-80
FXYD1	iFIB	66.00%	5.40%	0.60	4.89E-79
FSTL1	iFIB	85.60%	13.70%	1.52	9.47E-79
DDR2	iFIB	54.60%	3.20%	0.61	4.01E-78
CFI	iFIB	54.60%	3.20%	0.61	4.50E-78
PLAC9	iFIB	91.80%	17.20%	1.69	3.76E-77
ABI3BP	iFIB	76.30%	8.80%	0.91	2.15E-76
COL5A2	iFIB	58.80%	4.20%	0.76	2.41E-76
ANGPTL4	iFIB	76.30%	9.40%	1.26	5.40E-76
TNXB	iFIB	81.40%	12.10%	1.33	1.28E-75
AEBP1	iFIB	58.80%	4.70%	0.83	8.88E-74
FBN1	iFIB	54.60%	3.70%	0.71	1.21E-73
VASN	iFIB	67.00%	6.50%	0.89	1.68E-73
CRYAB	iFIB	66.00%	6.50%	0.92	8.46E-73
SCARA5	iFIB	48.50%	2.70%	0.68	1.80E-72
COL6A1	iFIB	88.70%	17.00%	1.49	4.15E-72
NT5E	iFIB	50.50%	2.90%	0.45	1.16E-71
NNMT	iFIB	93.80%	23.10%	1.80	2.27E-71
TMEM45A	iFIB	53.60%	3.50%	0.46	2.47E-71
CYR61	iFIB	69.10%	7.70%	1.04	4.06E-71
PDLIM4	iFIB	63.90%	5.80%	0.63	5.34E-71
PDGFRA	iFIB	54.60%	3.90%	0.63	7.07E-71
PDLIM3	iFIB	50.50%	3.00%	0.52	8.69E-71
PDGFRL	iFIB	60.80%	5.40%	0.77	1.26E-70
COL1A2	iFIB	99.00%	29.70%	2.26	4.49E-70
LRRN4CL	iFIB	37.10%	1.10%	0.35	3.20E-69
COL6A3	iFIB	84.50%	14.10%	1.32	3.34E-69
DLC1	iFIB	44.30%	2.10%	0.37	5.02E-68
PTPRS	iFIB	46.40%	2.60%	0.77	9.07E-68
IFITM1	iFIB	37.10%	1.20%	0.42	1.49E-67

PDGFD	iFIB	43.30%	2.10%	0.38	1.60E-67
CTGF	iFIB	86.60%	15.50%	1.46	1.87E-67
ENAH	iFIB	57.70%	4.70%	0.50	2.18E-67
CRISPLD1	iFIB	41.20%	1.80%	0.42	5.72E-66
NTN4	iFIB	39.20%	1.60%	0.38	4.39E-65
MRGPRF	iFIB	35.10%	1.00%	0.34	5.16E-65
MRC2	iFIB	51.50%	3.80%	0.53	1.92E-64
ENPP1	iFIB	49.50%	3.50%	0.64	4.63E-64
THBS3	iFIB	57.70%	5.20%	0.57	1.29E-63
CFH	iFIB	64.90%	7.70%	1.11	7.56E-63
HSPB8	iFIB	46.40%	2.90%	0.52	8.63E-63
C1S	iFIB	87.60%	20.80%	1.65	1.01E-62
COX7A1	iFIB	71.10%	9.30%	0.79	1.16E-62
MT1E	iFIB	82.50%	14.90%	1.37	2.24E-62
SNED1	iFIB	39.20%	1.70%	0.36	2.53E-62
FAM134B	iFIB	56.70%	5.10%	0.54	3.44E-61
WWTR1	iFIB	45.40%	2.80%	0.36	3.85E-61
TMEM100	iFIB	44.30%	2.70%	0.51	4.63E-61
FILIP1	iFIB	25.80%	0.20%	0.28	6.39E-61
COL8A1	iFIB	35.10%	1.30%	0.41	1.19E-60
THBS4	iFIB	59.80%	6.30%	0.78	5.19E-60
SPARCL1	iFIB	86.60%	19.70%	1.78	8.72E-60
FGF10	iFIB	35.10%	1.30%	0.35	2.08E-59
NOVA1	iFIB	37.10%	1.70%	0.44	3.32E-59
EFEMP1	iFIB	77.30%	13.30%	1.32	4.37E-59
OSR2	iFIB	53.60%	4.80%	0.62	6.04E-59
FBLN2	iFIB	44.30%	2.80%	0.50	8.36E-59
PCOLCE2	iFIB	71.10%	11.30%	1.30	1.04E-58
GULP1	iFIB	33.00%	1.10%	0.29	1.51E-58
PAM	iFIB	75.30%	10.60%	0.61	2.01E-58
SPTBN1	iFIB	50.50%	4.00%	0.40	3.10E-58
C1orf21	iFIB	46.40%	3.30%	0.43	3.57E-58
SEMA3C	iFIB	54.60%	5.00%	0.59	5.17E-58
PLOD2	iFIB	41.20%	2.40%	0.35	7.17E-58
FBLN1	iFIB	37.10%	1.70%	0.49	1.46E-57
CRLF1	iFIB	53.60%	5.10%	0.77	1.72E-57
WISP2	iFIB	74.20%	12.90%	1.64	2.01E-57
S1PR3	iFIB	38.10%	1.90%	0.40	2.04E-57
NGFRAP1	iFIB	61.90%	7.30%	0.69	2.34E-57
MAP1B	iFIB	33.00%	1.20%	0.47	3.53E-57
OGN	iFIB	58.80%	6.70%	1.02	4.76E-57

CERCAM	iFIB	47.40%	3.60%	0.45	8.34E-57
GFPT2	iFIB	66.00%	8.60%	0.81	2.32E-56
ABLIM1	iFIB	34.00%	1.30%	0.30	4.06E-56
MGST1	iFIB	55.70%	5.80%	0.64	6.64E-56
PPAP2A	iFIB	64.90%	8.90%	0.85	1.03E-55
STEAP4	iFIB	40.20%	2.40%	0.60	1.10E-55
HAS1	iFIB	76.30%	12.50%	1.17	1.55E-55
THY1	iFIB	57.70%	6.70%	0.94	1.74E-55
FHL1	iFIB	79.40%	15.80%	1.29	2.11E-55
RCN3	iFIB	68.00%	9.40%	0.70	2.20E-55
CTTN	iFIB	53.60%	5.00%	0.52	2.30E-55
AKR1C1	iFIB	59.80%	6.80%	0.64	5.04E-55
GPRC5A	iFIB	74.20%	12.40%	0.91	7.42E-55
TMEM98	iFIB	34.00%	1.40%	0.31	8.09E-55
LARP6	iFIB	35.10%	1.60%	0.30	1.32E-54
C9orf3	iFIB	40.20%	2.40%	0.37	1.90E-54
MYL9	iFIB	69.10%	10.40%	0.91	1.94E-54
ADAMTS1	iFIB	49.50%	4.30%	0.60	2.12E-54
LTBP4	iFIB	56.70%	6.30%	0.73	2.66E-54
MGP	iFIB	99.00%	58.70%	2.46	2.73E-54
SMOC2	iFIB	36.10%	1.80%	0.47	5.54E-54
BGN	iFIB	87.60%	26.50%	1.84	7.00E-54
NBL1	iFIB	69.10%	10.30%	0.84	1.46E-53
DPT	iFIB	82.50%	19.80%	1.75	7.29E-53
LUM	iFIB	100.00%	59.80%	2.38	8.54E-53
SGCE	iFIB	33.00%	1.40%	0.36	8.94E-53
ZNF385D	iFIB	33.00%	1.40%	0.31	1.02E-52
ELN	iFIB	38.10%	2.30%	0.45	2.43E-52
DCN	iFIB	97.90%	56.40%	2.63	5.10E-52
GPX8	iFIB	45.40%	3.60%	0.38	7.46E-52
IGFBP5	iFIB	56.70%	6.90%	1.36	1.17E-51
GJA1	iFIB	43.30%	3.20%	0.41	1.26E-51
CD248	iFIB	34.00%	1.70%	0.38	1.93E-51
CDO1	iFIB	56.70%	7.00%	0.92	4.38E-51
SFRP1	iFIB	36.10%	2.10%	0.56	4.55E-51
C11orf96	iFIB	49.50%	4.90%	0.61	5.48E-51
P4HA2	iFIB	57.70%	6.50%	0.40	6.13E-51
ERRFI1	iFIB	90.70%	23.80%	1.41	9.36E-51
PDPN	iFIB	54.60%	5.90%	0.49	1.33E-50
IGFBP6	iFIB	88.70%	24.80%	1.93	2.88E-50
MT1A	iFIB	55.70%	6.90%	1.07	3.03E-50

A4GALT	iFIB	36.10%	2.10%	0.34	3.29E-50
SGCD	iFIB	30.90%	1.30%	0.27	3.46E-50
ANKRD35	iFIB	30.90%	1.30%	0.27	3.65E-50
CP	iFIB	34.00%	1.70%	0.57	4.08E-50
C2orf40	iFIB	67.00%	10.70%	1.04	4.43E-50
PPAP2B	iFIB	67.00%	10.30%	0.90	1.36E-49
FMOD	iFIB	66.00%	10.40%	1.01	1.43E-49
CDON	iFIB	41.20%	3.00%	0.34	1.57E-49
GALNT15	iFIB	34.00%	1.80%	0.39	3.21E-49
NTRK2	iFIB	32.00%	1.50%	0.44	3.79E-49
FAP	iFIB	38.10%	2.50%	0.36	5.94E-49
PTRF	iFIB	79.40%	16.50%	0.94	8.00E-49
SNAI2	iFIB	32.00%	1.50%	0.38	8.74E-49
MXRA5	iFIB	42.30%	3.40%	0.52	8.86E-49
F5	iFIB	34.00%	1.80%	0.38	9.70E-49
SPON2	iFIB	35.10%	2.10%	0.63	2.28E-48
TMEM196	iFIB	33.00%	1.70%	0.46	2.92E-48
PLA2G2A	iFIB	97.90%	58.30%	3.05	4.23E-48
ASPN	iFIB	80.40%	21.90%	2.12	6.86E-48
LBH	iFIB	62.90%	9.00%	0.62	8.05E-48
DKK3	iFIB	52.60%	6.30%	0.95	9.19E-48
LTBP1	iFIB	43.30%	3.60%	0.44	9.76E-48
RBMS3	iFIB	36.10%	2.20%	0.25	2.10E-47
AUTS2	iFIB	36.10%	2.30%	0.37	4.30E-47
ACKR3	iFIB	50.50%	5.50%	0.59	6.48E-47
S100A16	iFIB	39.20%	2.80%	0.32	1.01E-46
STEAP1	iFIB	35.10%	2.10%	0.33	1.31E-46
FAM114A1	iFIB	60.80%	8.40%	0.55	2.05E-46
SERPING1	iFIB	90.70%	31.80%	1.17	2.80E-46
PLS3	iFIB	50.50%	5.40%	0.37	3.58E-46
RBFOX2	iFIB	34.00%	2.00%	0.26	3.91E-46
PFN2	iFIB	34.00%	2.00%	0.26	4.98E-46
DBN1	iFIB	30.90%	1.50%	0.30	5.29E-46
STEAP2	iFIB	26.80%	0.90%	0.28	8.46E-46
SELM	iFIB	96.90%	37.40%	1.06	1.07E-45
PCDH9	iFIB	27.80%	1.10%	0.27	1.72E-45
AK1	iFIB	79.40%	19.40%	1.13	7.47E-45
KDELR3	iFIB	45.40%	4.60%	0.45	2.43E-44
ANK2	iFIB	41.20%	3.60%	0.38	3.61E-44
COL4A1	iFIB	34.00%	2.10%	0.29	4.52E-44
FAM180B	iFIB	27.80%	1.20%	0.26	7.51E-44

EFNA5	iFIB	24.70%	0.80%	0.28	7.71E-44
SEMA3E	iFIB	24.70%	0.80%	0.26	7.71E-44
AKR1C2	iFIB	49.50%	5.80%	0.67	8.79E-44
PDGFRB	iFIB	39.20%	3.20%	0.38	1.13E-43
PROCR	iFIB	52.60%	6.90%	0.63	2.42E-43
FAT1	iFIB	26.80%	1.10%	0.25	4.44E-43
PTGES	iFIB	37.10%	2.80%	0.40	5.47E-43
TIMP3	iFIB	85.60%	25.60%	1.46	5.61E-43
COL12A1	iFIB	30.90%	1.70%	0.30	1.23E-42
TNC	iFIB	68.00%	13.90%	1.13	1.58E-42
SLC39A14	iFIB	55.70%	8.00%	0.62	2.92E-42
SDC2	iFIB	80.40%	20.40%	0.93	2.95E-42
CRABP2	iFIB	40.20%	3.70%	0.69	4.00E-42
THBS2	iFIB	33.00%	2.10%	0.34	4.01E-42
TNFAIP6	iFIB	84.50%	24.60%	1.44	1.60E-41
FHL2	iFIB	32.00%	2.10%	0.32	2.46E-41
FN1	iFIB	100.00%	79.70%	2.03	4.66E-41
RAMP2	iFIB	35.10%	2.50%	0.26	5.46E-41
PKIG	iFIB	44.30%	4.70%	0.37	9.07E-41
SCARA3	iFIB	37.10%	3.10%	0.32	3.13E-40
CADM3	iFIB	28.90%	1.60%	0.27	4.25E-40
FNDC1	iFIB	28.90%	1.60%	0.33	4.78E-40
FAM180A	iFIB	27.80%	1.40%	0.32	4.89E-40
SRPX	iFIB	25.80%	1.10%	0.28	5.37E-40
NFIX	iFIB	47.40%	5.40%	0.38	8.64E-40
COL5A1	iFIB	37.10%	3.20%	0.49	9.25E-40
VCAM1	iFIB	43.30%	4.70%	0.73	1.08E-39
HSD3B7	iFIB	66.00%	12.90%	0.72	1.37E-39
UGDH	iFIB	67.00%	14.00%	0.89	1.60E-39
SSC5D	iFIB	40.20%	3.90%	0.36	1.98E-39
PRELP	iFIB	80.40%	23.70%	1.63	3.96E-39
MT2A	iFIB	99.00%	71.50%	1.79	8.17E-39
C16orf45	iFIB	35.10%	2.80%	0.31	1.78E-38
SMARCA1	iFIB	29.90%	1.90%	0.30	1.90E-38
CXCL12	iFIB	60.80%	11.20%	1.26	2.68E-38
PTN	iFIB	20.60%	0.60%	0.35	3.27E-38
GLI3	iFIB	25.80%	1.30%	0.26	5.79E-38
SPARC	iFIB	88.70%	29.80%	1.32	7.46E-38
MMP2	iFIB	71.10%	17.90%	1.19	7.75E-38
SULF1	iFIB	33.00%	2.50%	0.30	2.16E-37
PRKCDBP	iFIB	83.50%	23.10%	0.80	2.27E-37

EMP2	iFIB	72.20%	16.30%	0.68	2.41E-37
AQP1	iFIB	80.40%	24.40%	1.24	4.38E-37
COL1A1	iFIB	67.00%	16.30%	2.33	5.91E-37
CSGALNACT1	iFIB	32.00%	2.40%	0.28	7.44E-37
DEFB1	iFIB	50.50%	7.50%	0.83	8.95E-37
LY6E	iFIB	74.20%	17.40%	0.78	3.77E-36
FKBP7	iFIB	36.10%	3.30%	0.26	5.51E-36
TCEA3	iFIB	33.00%	2.70%	0.27	5.82E-36
SERPINH1	iFIB	61.90%	11.10%	0.49	6.42E-36
MFAP5	iFIB	37.10%	3.80%	1.16	1.34E-35
COL4A2	iFIB	34.00%	2.90%	0.38	1.34E-35
MYC	iFIB	78.40%	21.80%	0.92	1.58E-35
GAS1	iFIB	26.80%	1.60%	0.27	1.95E-35
ASS1	iFIB	26.80%	1.70%	0.29	7.86E-35
CRTAC1	iFIB	94.80%	52.50%	2.16	1.03E-34
LPAR1	iFIB	40.20%	4.50%	0.34	2.29E-34
LTBP3	iFIB	44.30%	5.80%	0.42	1.37E-33
GAP43	iFIB	20.60%	0.80%	0.30	2.26E-33
CLU	iFIB	99.00%	67.60%	1.91	2.43E-33
SOX9	iFIB	23.70%	1.30%	0.28	5.80E-33
ITGB8	iFIB	45.40%	6.20%	0.42	1.34E-32
TPM2	iFIB	51.50%	8.40%	0.49	1.67E-32
SGCB	iFIB	37.10%	4.00%	0.27	2.11E-32
ID3	iFIB	82.50%	25.70%	0.96	2.18E-32
PRG4	iFIB	97.90%	76.60%	2.41	2.53E-32
GPX3	iFIB	88.70%	34.70%	1.05	3.77E-32
KCNE4	iFIB	24.70%	1.50%	0.33	5.20E-32
CHST12	iFIB	44.30%	6.00%	0.36	6.02E-32
IGFBP4	iFIB	81.40%	24.50%	1.05	8.98E-32
GEM	iFIB	86.60%	30.50%	1.19	1.85E-31
TNFRSF11B	iFIB	37.10%	4.40%	0.45	2.38E-31
CRISPLD2	iFIB	43.30%	5.80%	0.42	2.50E-31
FGFBP2	iFIB	47.40%	8.10%	1.39	2.61E-31
PTHLH	iFIB	24.70%	1.60%	0.32	3.98E-31
MYL6B	iFIB	50.50%	8.00%	0.32	5.32E-31
HSPG2	iFIB	40.20%	5.00%	0.33	6.47E-31
PLEKHA4	iFIB	39.20%	4.80%	0.31	9.91E-31
INHBA	iFIB	68.00%	16.10%	1.29	1.29E-30
NDUFA4L2	iFIB	64.90%	18.30%	1.52	1.35E-30
SFRP4	iFIB	32.00%	3.20%	0.81	1.56E-30
RGS3	iFIB	36.10%	4.00%	0.34	1.56E-30

ITM2C	iFIB	49.50%	8.10%	0.47	1.57E-30
PARVA	iFIB	37.10%	4.30%	0.27	2.02E-30
GOLM1	iFIB	40.20%	5.20%	0.36	2.21E-30
CNPY4	iFIB	35.10%	3.80%	0.25	3.40E-30
SOX4	iFIB	78.40%	21.00%	0.63	4.30E-30
ARID5B	iFIB	91.80%	31.80%	0.82	4.41E-30
RCN1	iFIB	51.50%	8.60%	0.35	6.97E-30
LGALS3BP	iFIB	67.00%	16.10%	0.61	1.91E-29
TGFBR3	iFIB	48.50%	8.00%	0.45	2.30E-29
CYB5R3	iFIB	90.70%	37.90%	0.77	2.52E-29
BDH2	iFIB	52.60%	8.90%	0.32	3.38E-29
SEMA5A	iFIB	17.50%	0.60%	0.31	1.05E-28
WBP5	iFIB	84.50%	25.80%	0.63	1.71E-28
GGT5	iFIB	18.60%	0.80%	0.36	2.20E-28
COL3A1	iFIB	78.40%	29.10%	2.16	2.34E-28
DIO2	iFIB	26.80%	2.20%	0.35	2.66E-28
CAPS	iFIB	35.10%	4.30%	0.42	4.36E-28
TPPP3	iFIB	89.70%	46.80%	1.17	6.51E-28
CES1	iFIB	34.00%	3.90%	0.30	8.65E-28
SCRG1	iFIB	39.20%	5.70%	0.64	9.28E-28
TM4SF1	iFIB	51.50%	9.80%	0.48	1.05E-27
ADD3	iFIB	42.30%	6.20%	0.30	1.41E-27
ST6GALNAC6	iFIB	47.40%	8.00%	0.34	5.17E-27
CDA	iFIB	23.70%	1.70%	0.28	5.19E-27
SCG2	iFIB	18.60%	0.90%	0.29	6.36E-27
PRSS23	iFIB	76.30%	26.40%	1.03	1.98E-26
SFRP2	iFIB	24.70%	2.10%	0.76	3.14E-26
UAP1	iFIB	82.50%	29.30%	0.79	4.36E-26
SORBS2	iFIB	24.70%	2.10%	0.28	5.19E-26
COMP	iFIB	84.50%	38.40%	2.06	5.27E-26
P4HA3	iFIB	27.80%	2.70%	0.25	6.18E-26
PPA1	iFIB	72.20%	19.30%	0.48	8.47E-26
PTPRG	iFIB	29.90%	3.20%	0.25	8.72E-26
LDHA	iFIB	99.00%	77.90%	0.79	1.05E-25
MAGED1	iFIB	38.10%	5.40%	0.25	1.46E-25
COL18A1	iFIB	32.00%	3.70%	0.25	2.06E-25
FKBP9	iFIB	47.40%	8.40%	0.33	2.48E-25
VKORC1	iFIB	88.70%	35.50%	0.73	2.56E-25
ADIRF	iFIB	94.80%	50.00%	0.92	2.64E-25
IFT57	iFIB	50.50%	9.60%	0.36	2.70E-25
LAPTM4A	iFIB	97.90%	77.00%	0.80	3.33E-25

IFITM3	iFIB	95.90%	66.10%	0.92	5.83E-25
NUCB2	iFIB	67.00%	16.40%	0.42	9.16E-25
DUSP14	iFIB	49.50%	9.50%	0.38	1.39E-24
PPIC	iFIB	78.40%	25.50%	0.59	1.92E-24
COPZ2	iFIB	40.20%	6.20%	0.25	2.71E-24
AHI1	iFIB	74.20%	19.80%	0.51	3.00E-24
REXO2	iFIB	77.30%	28.90%	0.75	3.73E-24
ANTXR1	iFIB	43.30%	7.30%	0.36	5.29E-24
CHPF	iFIB	29.90%	3.50%	0.26	8.63E-24
SERPINE2	iFIB	30.90%	3.90%	0.43	1.21E-23
OLFML3	iFIB	36.10%	5.30%	0.35	1.33E-23
SMIM14	iFIB	72.20%	21.90%	0.59	1.98E-23
S100A13	iFIB	77.30%	30.20%	0.79	2.13E-23
C8orf4	iFIB	21.60%	1.70%	0.44	3.80E-23
IL1R1	iFIB	44.30%	7.70%	0.30	4.90E-23
PDLIM2	iFIB	54.60%	12.30%	0.37	7.02E-22
CILP	iFIB	39.20%	7.00%	0.71	8.81E-22
CBR3	iFIB	52.60%	11.40%	0.38	9.22E-22
CTHRC1	iFIB	20.60%	1.70%	0.39	1.24E-21
EPB41L2	iFIB	75.30%	23.80%	0.50	2.48E-21
IFI27	iFIB	74.20%	23.10%	0.67	2.94E-21
PFKP	iFIB	67.00%	19.70%	0.56	5.08E-21
PDGFA	iFIB	30.90%	4.20%	0.25	9.12E-21
ZBTB20	iFIB	55.70%	12.60%	0.27	3.58E-20
IGF1	iFIB	55.70%	15.90%	1.19	3.88E-20
C12orf57	iFIB	81.40%	36.10%	0.65	4.07E-20
RGS16	iFIB	61.90%	18.50%	0.73	8.14E-20
HTRA1	iFIB	91.80%	52.60%	1.14	9.41E-20
VAMP5	iFIB	64.90%	17.60%	0.36	1.15E-19
VCAN	iFIB	75.30%	30.50%	0.93	1.75E-19
NAV1	iFIB	44.30%	8.90%	0.29	1.79E-19
MT1X	iFIB	83.50%	39.40%	1.16	1.98E-19
RORA	iFIB	43.30%	8.80%	0.32	5.18E-19
NFIA	iFIB	66.00%	18.90%	0.41	5.53E-19
PRDX4	iFIB	68.00%	22.30%	0.47	7.46E-19
ARL2	iFIB	84.50%	32.50%	0.53	8.00E-19
TIMP1	iFIB	99.00%	82.80%	0.79	8.10E-19
SBDS	iFIB	84.50%	40.50%	0.64	9.95E-19
TMED3	iFIB	59.80%	16.00%	0.34	1.06E-18
MAP7D3	iFIB	43.30%	8.80%	0.27	1.42E-18
C1GALT1C1	iFIB	45.40%	9.90%	0.27	4.71E-18

TIMP2	iFIB	95.90%	54.00%	0.62	7.72E-18
PLAGL1	iFIB	37.10%	6.90%	0.25	1.34E-17
AIG1	iFIB	51.50%	12.30%	0.26	1.89E-17
CYBRD1	iFIB	77.30%	28.80%	0.47	2.74E-17
MATN2	iFIB	24.70%	3.20%	0.26	3.71E-17
EID1	iFIB	93.80%	59.90%	0.61	4.19E-17
PMEPA1	iFIB	61.90%	18.10%	0.39	4.69E-17
CD151	iFIB	87.60%	49.50%	0.68	5.15E-17
NCOA7	iFIB	45.40%	10.20%	0.28	5.63E-17
FYN	iFIB	40.20%	8.40%	0.28	7.48E-17
CADM1	iFIB	25.80%	3.60%	0.30	7.53E-17
NFIC	iFIB	79.40%	32.40%	0.56	1.41E-16
BPGM	iFIB	39.20%	8.10%	0.30	1.41E-16
RRAS	iFIB	46.40%	10.70%	0.27	2.22E-16
FAM20C	iFIB	38.10%	7.70%	0.28	2.24E-16
FAM127A	iFIB	58.80%	16.60%	0.34	2.30E-16
PPFIBP1	iFIB	41.20%	8.90%	0.28	2.51E-16
TWISTNB	iFIB	57.70%	16.70%	0.60	3.43E-16
CREB3L2	iFIB	55.70%	15.20%	0.35	4.10E-16
RARRES2	iFIB	15.50%	1.20%	0.25	4.94E-16
TUBB2B	iFIB	28.90%	4.70%	0.33	5.43E-16
FAM3C	iFIB	68.00%	21.70%	0.39	6.97E-16
SLC39A7	iFIB	68.00%	23.00%	0.39	1.95E-15
PDLIM7	iFIB	53.60%	14.70%	0.33	2.76E-15
AP3S1	iFIB	80.40%	32.40%	0.44	2.85E-15
C1GALT1	iFIB	37.10%	7.90%	0.32	4.29E-15
TAGLN	iFIB	47.40%	13.40%	0.99	6.61E-15
CHI3L2	iFIB	26.80%	4.40%	0.74	7.20E-15
PRDX2	iFIB	72.20%	26.30%	0.39	1.09E-14
VAMP2	iFIB	75.30%	30.50%	0.47	1.47E-14
COPS8	iFIB	55.70%	16.20%	0.32	2.01E-14
GPAA1	iFIB	66.00%	22.30%	0.37	2.18E-14
LRP1	iFIB	95.90%	49.60%	0.52	2.41E-14
CHI3L1	iFIB	28.90%	5.30%	0.83	2.45E-14
ID1	iFIB	44.30%	11.40%	0.47	3.68E-14
ERLEC1	iFIB	63.90%	20.20%	0.31	4.08E-14
CTSK	iFIB	77.30%	28.90%	0.39	4.19E-14
PSD3	iFIB	56.70%	16.30%	0.29	5.31E-14
AKR1C3	iFIB	38.10%	8.60%	0.29	5.54E-14
SLC25A37	iFIB	50.50%	13.80%	0.38	5.61E-14
LRP10	iFIB	68.00%	24.50%	0.40	6.11E-14

S100A8	iFIB	43.30%	11.50%	0.83	7.78E-14
RRBP1	iFIB	86.60%	39.60%	0.46	8.07E-14
PROS1	iFIB	47.40%	12.50%	0.28	9.27E-14
C19orf10	iFIB	83.50%	42.90%	0.48	1.67E-13
LAMB2	iFIB	41.20%	10.40%	0.33	2.21E-13
RND3	iFIB	68.00%	22.70%	0.35	2.53E-13
CCPG1	iFIB	77.30%	29.80%	0.42	2.54E-13
AXL	iFIB	59.80%	20.80%	0.49	3.61E-13
CYP1B1	iFIB	90.70%	49.30%	0.65	5.13E-13
IFI6	iFIB	62.90%	22.40%	0.43	5.65E-13
SLC29A1	iFIB	39.20%	9.60%	0.31	6.34E-13
C1orf122	iFIB	63.90%	21.90%	0.35	8.82E-13
NDUFC1	iFIB	78.40%	32.10%	0.37	9.90E-13
OPTN	iFIB	48.50%	13.70%	0.28	1.91E-12
GABARAPL2	iFIB	93.80%	56.80%	0.52	1.94E-12
FGFR1	iFIB	63.90%	23.70%	0.39	2.18E-12
FERMT2	iFIB	53.60%	16.10%	0.26	3.28E-12
KDELR2	iFIB	78.40%	36.80%	0.46	4.00E-12
PLEKHA1	iFIB	47.40%	13.40%	0.32	4.19E-12
HBEGF	iFIB	69.10%	28.70%	0.63	5.35E-12
CLEC3B	iFIB	44.30%	11.80%	0.26	5.62E-12
TXNDC15	iFIB	52.60%	15.90%	0.26	5.95E-12
RHOBTB3	iFIB	29.90%	6.10%	0.25	1.15E-11
IGFBP7	iFIB	66.00%	28.70%	0.73	1.27E-11
CD63	iFIB	100.00%	98.70%	0.37	1.38E-11
CPQ	iFIB	60.80%	22.50%	0.35	1.44E-11
VGLL4	iFIB	42.30%	11.50%	0.27	2.14E-11
FAM46A	iFIB	60.80%	24.50%	0.62	2.26E-11
DST	iFIB	81.40%	35.80%	0.40	2.33E-11
LTBP2	iFIB	39.20%	10.30%	0.31	2.56E-11
CD9	iFIB	95.90%	81.70%	0.77	2.95E-11
RARRES1	iFIB	16.50%	2.10%	0.41	3.10E-11
NDRG1	iFIB	71.10%	26.40%	0.29	3.16E-11
SVIL	iFIB	37.10%	9.60%	0.31	3.61E-11
CUX1	iFIB	60.80%	21.10%	0.31	3.95E-11
OAT	iFIB	64.90%	24.60%	0.35	4.07E-11
TTC3	iFIB	73.20%	29.40%	0.35	7.45E-11
TCF4	iFIB	74.20%	29.90%	0.37	7.65E-11
ZNF503	iFIB	50.50%	15.90%	0.31	7.92E-11
GLIS3	iFIB	43.30%	12.40%	0.29	8.79E-11
ANKH	iFIB	74.20%	33.00%	0.44	1.00E-10

TMEM59	iFIB	93.80%	68.40%	0.43	1.05E-10
PPP1CC	iFIB	55.70%	19.10%	0.35	1.08E-10
GSN	iFIB	93.80%	78.40%	0.59	2.26E-10
RABAC1	iFIB	93.80%	60.20%	0.46	3.03E-10
CALU	iFIB	79.40%	36.20%	0.41	4.15E-10
DSTN	iFIB	96.90%	83.70%	0.50	4.46E-10
CTNNAL1	iFIB	38.10%	10.30%	0.26	4.67E-10
NUPR1	iFIB	92.80%	49.90%	0.47	4.79E-10
SRI	iFIB	71.10%	31.10%	0.39	5.40E-10
MAGED2	iFIB	60.80%	21.90%	0.31	5.63E-10
APP	iFIB	83.50%	39.60%	0.47	8.90E-10
TPM1	iFIB	53.60%	17.80%	0.31	1.03E-09
SLC25A36	iFIB	42.30%	12.60%	0.26	1.09E-09
MINOS1	iFIB	93.80%	66.10%	0.42	1.12E-09
KCNMA1	iFIB	49.50%	15.70%	0.39	1.47E-09
SEC61A1	iFIB	46.40%	14.40%	0.25	1.89E-09
TFG	iFIB	67.00%	27.00%	0.31	2.26E-09
SEPW1	iFIB	85.60%	41.40%	0.38	3.12E-09
RPS4Y1	iFIB	54.60%	20.90%	0.48	4.10E-09
RPL3	iFIB	100.00%	99.70%	0.29	5.25E-09
MRPL32	iFIB	55.70%	20.20%	0.28	7.37E-09
PTGDS	iFIB	24.70%	5.30%	0.75	7.52E-09
P4HB	iFIB	85.60%	48.90%	0.41	7.73E-09
KCNQ1OT1	iFIB	86.60%	42.70%	0.44	7.84E-09
RBBP6	iFIB	81.40%	39.30%	0.40	8.40E-09
TFPI	iFIB	55.70%	19.60%	0.28	8.88E-09
MPG	iFIB	56.70%	21.50%	0.31	1.05E-08
EEF2	iFIB	99.00%	90.10%	0.33	1.08E-08
CD276	iFIB	47.40%	15.90%	0.25	1.26E-08
PPP3CA	iFIB	61.90%	26.80%	0.39	1.27E-08
SERTAD1	iFIB	90.70%	54.70%	0.45	1.56E-08
SLPI	iFIB	15.50%	2.20%	0.64	1.61E-08
SOCS3	iFIB	86.60%	50.60%	0.50	2.56E-08
LEPROT	iFIB	76.30%	36.10%	0.34	2.80E-08
GINM1	iFIB	59.80%	22.50%	0.28	3.18E-08
YIF1A	iFIB	57.70%	21.60%	0.29	3.85E-08
IRF1	iFIB	74.20%	39.00%	0.46	4.24E-08
ARL6IP5	iFIB	95.90%	66.80%	0.37	4.56E-08
EGR1	iFIB	91.80%	56.40%	0.37	4.62E-08
H2AFJ	iFIB	74.20%	35.50%	0.39	5.55E-08
EPHX1	iFIB	72.20%	31.00%	0.34	6.27E-08

NUCKS1	iFIB	90.70%	58.40%	0.42	7.16E-08
UGP2	iFIB	81.40%	45.00%	0.47	1.07E-07
COPS6	iFIB	71.10%	30.20%	0.27	1.67E-07
DYNLT1	iFIB	59.80%	23.40%	0.25	2.33E-07
ITM2B	iFIB	100.00%	98.30%	0.35	2.72E-07
JUNB	iFIB	100.00%	95.80%	0.45	3.40E-07
IL6ST	iFIB	66.00%	28.60%	0.35	3.44E-07
PPIB	iFIB	93.80%	71.60%	0.40	3.62E-07
TNFRSF12A	iFIB	69.10%	32.80%	0.52	5.11E-07
ARL4D	iFIB	55.70%	23.40%	0.34	5.38E-07
MIR4435-1HG	iFIB	53.60%	20.30%	0.28	5.73E-07
PXDC1	iFIB	55.70%	21.30%	0.26	8.95E-07
H3F3B	iFIB	100.00%	99.00%	0.27	1.00E-06
LGALS3	iFIB	100.00%	90.20%	0.35	1.09E-06
CCND1	iFIB	52.60%	21.50%	0.36	1.22E-06
CD81	iFIB	87.60%	50.00%	0.32	1.67E-06
COL15A1	iFIB	14.40%	2.40%	0.35	1.85E-06
SPTSSA	iFIB	67.00%	30.50%	0.29	2.56E-06
SKP1	iFIB	96.90%	75.50%	0.34	2.62E-06
RBBP7	iFIB	56.70%	22.80%	0.26	3.05E-06
SERPINF1	iFIB	87.60%	58.30%	0.66	3.06E-06
LMO4	iFIB	63.90%	28.70%	0.32	3.52E-06
CFD	iFIB	95.90%	87.30%	0.93	3.60E-06
SOD1	iFIB	94.80%	77.30%	0.37	4.16E-06
CAMLG	iFIB	71.10%	33.30%	0.28	6.96E-06
OST4	iFIB	97.90%	77.00%	0.35	7.05E-06
RPL10A	iFIB	100.00%	99.10%	0.26	7.65E-06
CHCHD2	iFIB	96.90%	85.60%	0.32	1.10E-05
DAD1	iFIB	93.80%	60.30%	0.33	1.17E-05
APOE	iFIB	37.10%	12.70%	0.35	1.25E-05
RCAN1	iFIB	59.80%	26.10%	0.29	1.91E-05
ISCU	iFIB	86.60%	49.30%	0.29	1.97E-05
VIMP	iFIB	80.40%	43.50%	0.31	3.32E-05
SIVA1	iFIB	72.20%	34.40%	0.25	3.51E-05
MLF2	iFIB	68.00%	31.60%	0.25	3.90E-05
PSMB5	iFIB	68.00%	33.90%	0.26	4.08E-05
IL6	iFIB	52.60%	23.40%	0.43	4.29E-05
MORF4L2	iFIB	85.60%	44.10%	0.29	4.42E-05
MMP3	iFIB	27.80%	8.50%	0.52	4.49E-05
ATRAID	iFIB	83.50%	49.30%	0.30	4.61E-05
MRPL51	iFIB	81.40%	44.40%	0.27	4.62E-05

GUK1	iFIB	92.80%	71.40%	0.35	4.99E-05
ELL2	iFIB	64.90%	30.50%	0.28	5.73E-05
SSR2	iFIB	86.60%	53.90%	0.29	6.63E-05
GOLGA4	iFIB	87.60%	46.30%	0.29	9.48E-05
HCFC1R1	iFIB	63.90%	31.30%	0.29	1.04E-04
CREB5	iFIB	54.60%	24.90%	0.28	1.30E-04
CRIP2	iFIB	75.30%	44.20%	0.36	1.66E-04
HDLBP	iFIB	73.20%	38.90%	0.29	1.83E-04
HNRNPA0	iFIB	94.80%	59.90%	0.29	1.95E-04
AKAP9	iFIB	83.50%	43.60%	0.26	2.06E-04
BSG	iFIB	88.70%	58.30%	0.32	2.43E-04
CYSTM1	iFIB	77.30%	41.30%	0.28	3.19E-04
C4orf3	iFIB	88.70%	55.00%	0.33	3.29E-04
PEBP1	iFIB	91.80%	63.90%	0.29	3.85E-04
TPI1	iFIB	97.90%	81.90%	0.29	4.39E-04
TUBB2A	iFIB	77.30%	44.80%	0.27	4.79E-04
ERGIC3	iFIB	78.40%	44.50%	0.26	5.11E-04
EMC4	iFIB	56.70%	27.20%	0.26	6.03E-04
TMEM258	iFIB	92.80%	68.40%	0.26	6.90E-04
ITGB1	iFIB	88.70%	61.30%	0.31	8.26E-04
LRPAP1	iFIB	72.20%	41.70%	0.27	8.76E-04
PIGT	iFIB	54.60%	26.50%	0.29	1.40E-03
EMC7	iFIB	63.90%	33.20%	0.25	1.57E-03
RAB2A	iFIB	80.40%	48.50%	0.25	1.96E-03
OSTC	iFIB	72.20%	40.80%	0.27	2.09E-03
GOLIM4	iFIB	72.20%	38.10%	0.31	2.12E-03
MGST3	iFIB	89.70%	74.00%	0.29	2.17E-03
NDFIP1	iFIB	88.70%	58.20%	0.30	2.69E-03
KDELR1	iFIB	75.30%	41.90%	0.26	2.79E-03
YWHAQ	iFIB	84.50%	54.90%	0.30	3.83E-03
IFITM2	iFIB	92.80%	65.60%	0.33	5.16E-03
MAT2A	iFIB	76.30%	45.10%	0.29	5.46E-03
RAN	iFIB	90.70%	64.90%	0.26	8.93E-03
DYNLL1	iFIB	99.00%	87.70%	0.25	9.98E-03
SEC61G	iFIB	95.90%	69.20%	0.28	1.61E-02
TMED9	iFIB	85.60%	54.60%	0.26	1.87E-02
SHFM1	iFIB	92.80%	65.00%	0.25	1.87E-02
7-Sep	iFIB	86.60%	54.10%	0.25	2.03E-02
GPX4	iFIB	96.90%	87.60%	0.26	2.14E-02
PPDPF	iFIB	93.80%	73.70%	0.29	2.70E-02
JTB	iFIB	81.40%	56.60%	0.27	3.11E-02

ALDOA	iFIB	99.00%	87.30%	0.26	4.34E-02
APOC1	iFIB	25.80%	10.30%	0.49	3.19E-01
RPS26	iFIB	99.00%	84.10%	0.31	1.00E+00
PLP2	iFIB	74.20%	55.20%	0.28	1.00E+00
MZB1	B cell	100.00%	0.50%	4.09	3.83E-191
TNFRSF17	B cell	69.20%	0.10%	0.86	8.22E-182
RP11-16E12.2	B cell	61.50%	0.00%	1.09	2.18E-179
POU2AF1	B cell	61.50%	0.00%	0.72	2.18E-179
CD79A	B cell	100.00%	0.70%	2.61	9.67E-174
FCRL5	B cell	69.20%	0.10%	1.04	1.19E-164
SPAG4	B cell	84.60%	0.40%	1.68	2.43E-159
ZBTB32	B cell	53.80%	0.00%	0.96	1.47E-156
DERL3	B cell	100.00%	0.90%	3.37	7.43E-153
SMPDL3B	B cell	61.50%	0.10%	0.69	1.27E-142
FAM92B	B cell	53.80%	0.10%	0.83	1.44E-136
CD27	B cell	76.90%	0.50%	1.44	7.40E-131
IGJ	B cell	84.60%	0.70%	4.56	6.69E-129
CPNE5	B cell	46.20%	0.10%	0.48	3.83E-114
RP11-685N3.1	B cell	38.50%	0.00%	0.72	5.48E-111
APOBEC3B	B cell	61.50%	0.40%	0.89	9.73E-101
RP11-290F5.1	B cell	53.80%	0.30%	0.70	8.60E-98
C4orf26	B cell	38.50%	0.10%	0.71	1.03E-91
AC104699.1	B cell	38.50%	0.10%	0.28	1.03E-91
AIM2	B cell	53.80%	0.40%	0.53	1.37E-89
TAS1R3	B cell	30.80%	0.00%	0.39	3.07E-88
RASSF6	B cell	38.50%	0.10%	0.43	4.87E-78
RP11-81H14.2	B cell	38.50%	0.10%	0.32	6.07E-78
SDC1	B cell	53.80%	0.50%	0.62	3.17E-76
ZBP1	B cell	53.80%	0.50%	0.60	4.32E-76
ESR2	B cell	30.80%	0.10%	0.49	8.26E-70
GNB3	B cell	30.80%	0.10%	0.42	9.35E-70
C2orf88	B cell	38.50%	0.20%	0.38	6.64E-68
CTB-43E15.4	B cell	23.10%	0.00%	0.48	1.64E-65
KB-1980E6.3	B cell	23.10%	0.00%	0.37	1.64E-65
CD38	B cell	76.90%	1.80%	0.87	1.77E-63
PTP4A3	B cell	53.80%	0.80%	1.47	1.17E-58
IGLL5	B cell	100.00%	4.20%	4.94	1.07E-54
RALGPS2	B cell	61.50%	1.30%	0.53	6.07E-54
ZNF215	B cell	38.50%	0.40%	0.35	2.63E-53
LMTK3	B cell	30.80%	0.20%	0.44	7.64E-49
IGLL1	B cell	30.80%	0.20%	0.29	1.69E-48

E2F5	B cell	23.10%	0.10%	0.25	2.71E-48
KIAA0125	B cell	23.10%	0.10%	0.31	3.83E-48
RP11-446N19.1	B cell	38.50%	0.40%	0.40	4.69E-48
HLA-DOB	B cell	61.50%	1.60%	0.91	3.11E-45
PTPRCAP	B cell	69.20%	2.10%	0.96	1.11E-44
RP11-94L15.2	B cell	38.50%	0.50%	0.48	1.05E-43
HBD	B cell	15.40%	0.00%	0.51	8.62E-43
SMIM22	B cell	15.40%	0.00%	0.25	8.62E-43
CD79B	B cell	46.20%	0.90%	0.57	1.09E-41
FKBP11	B cell	100.00%	6.10%	2.58	3.44E-40
PAIP2B	B cell	38.50%	0.60%	0.40	5.34E-40
ZNF165	B cell	53.80%	1.40%	0.84	3.89E-39
SERPINI1	B cell	53.80%	1.50%	0.83	7.57E-38
JSRP1	B cell	30.80%	0.40%	0.55	6.48E-37
MCTP2	B cell	30.80%	0.40%	0.29	6.94E-37
GZMB	B cell	38.50%	0.70%	0.59	8.99E-37
RAB30	B cell	61.50%	2.40%	1.26	9.49E-33
TSPAN13	B cell	53.80%	1.70%	0.52	9.92E-33
BIK	B cell	61.50%	2.40%	0.80	2.19E-32
HIST1H1C	B cell	84.60%	4.80%	1.22	2.40E-32
RP11-1070N10.3	B cell	38.50%	0.80%	0.87	4.13E-32
PYCR1	B cell	53.80%	1.80%	0.59	4.57E-32
PNOC	B cell	23.10%	0.20%	0.30	5.53E-31
CLDN14	B cell	23.10%	0.20%	0.27	7.53E-31
GNG7	B cell	76.90%	4.20%	0.85	1.12E-29
HIST1H2AE	B cell	30.80%	0.50%	0.91	1.56E-29
GPR160	B cell	53.80%	2.10%	0.42	3.96E-27
BRSK1	B cell	46.20%	1.50%	0.51	4.83E-27
QPRT	B cell	69.20%	3.70%	0.83	7.09E-27
RHOH	B cell	76.90%	5.30%	1.24	1.17E-24
TEX9	B cell	30.80%	0.70%	0.43	1.85E-24
ICAM3	B cell	92.30%	7.80%	1.09	2.09E-24
RP11-532F12.5	B cell	30.80%	0.70%	0.26	2.35E-24
ITM2C	B cell	100.00%	10.20%	1.83	2.82E-24
SYNE2	B cell	53.80%	2.40%	0.93	1.59E-23
TMEM156	B cell	38.50%	1.20%	0.53	3.35E-23
AQP3	B cell	61.50%	3.40%	0.81	1.71E-22
SYTL1	B cell	38.50%	1.30%	0.45	3.29E-22
OCIAD2	B cell	61.50%	3.40%	0.48	4.63E-22
PERP	B cell	53.80%	2.70%	0.55	3.11E-21

CTH	B cell	38.50%	1.30%	0.50	4.82E-21
CHKB-AS1	B cell	30.80%	0.80%	0.27	1.68E-20
ATAD3C	B cell	38.50%	1.40%	0.34	1.67E-19
AC074289.1	B cell	61.50%	3.80%	0.53	2.69E-19
MEI1	B cell	53.80%	3.00%	0.45	2.79E-19
HIST1H2BJ	B cell	38.50%	1.50%	0.75	4.41E-19
KCNN3	B cell	30.80%	0.90%	0.39	5.41E-19
HIST2H2BE	B cell	53.80%	3.10%	0.89	2.24E-18
SYNGR1	B cell	46.20%	2.20%	0.33	2.38E-18
DNAAF1	B cell	38.50%	1.60%	1.81	3.02E-18
LY9	B cell	38.50%	1.60%	0.93	9.48E-18
AC017002.1	B cell	23.10%	0.50%	0.50	3.72E-17
RP1-313I6.12	B cell	84.60%	9.50%	1.27	1.08E-16
SLC38A5	B cell	30.80%	1.00%	0.33	1.31E-16
CERCAM	B cell	69.20%	6.10%	0.75	5.40E-16
CHPF	B cell	61.50%	4.80%	0.60	2.07E-15
TOR3A	B cell	92.30%	12.70%	1.10	2.64E-14
PIM2	B cell	84.60%	11.30%	1.52	2.97E-14
COBLL1	B cell	38.50%	2.00%	0.40	1.08E-13
TPD52	B cell	53.80%	3.90%	0.48	1.37E-13
ASNS	B cell	53.80%	4.30%	0.65	6.80E-13
PPAPDC1B	B cell	76.90%	9.50%	1.06	1.59E-12
HSH2D	B cell	23.10%	0.70%	0.26	3.79E-12
SLC38A1	B cell	61.50%	5.60%	0.47	4.84E-12
HIST1H2BK	B cell	92.30%	15.20%	1.20	1.18E-11
B4GALT7	B cell	76.90%	8.90%	0.55	1.28E-11
GLCCI1	B cell	46.20%	3.40%	0.44	3.14E-11
C3orf52	B cell	23.10%	0.80%	0.28	3.67E-11
IL2RG	B cell	69.20%	7.80%	0.57	4.27E-11
ERLEC1	B cell	100.00%	22.60%	1.45	6.89E-11
PRDX4	B cell	100.00%	24.80%	1.80	7.74E-11
ORMDL3	B cell	61.50%	6.10%	0.40	1.72E-10
ISG20	B cell	100.00%	22.20%	1.78	2.67E-10
SEC11C	B cell	100.00%	27.20%	2.11	3.00E-10
ST6GALNAC4	B cell	69.20%	8.70%	0.74	4.34E-10
RAB39B	B cell	23.10%	0.90%	0.26	5.00E-10
RNF122	B cell	30.80%	1.60%	0.35	6.27E-10
FAM46C	B cell	76.90%	11.20%	1.09	9.47E-10
YIPF2	B cell	61.50%	6.60%	0.50	1.01E-09
CTHRC1	B cell	38.50%	2.70%	0.52	1.53E-09
TAPBPL	B cell	69.20%	8.70%	0.74	1.57E-09

BLNK	B cell	76.90%	11.00%	0.77	2.63E-09
SRPRB	B cell	84.60%	14.70%	0.92	3.63E-09
LMF1	B cell	46.20%	4.00%	0.38	4.80E-09
TOR2A	B cell	38.50%	2.80%	0.35	6.36E-09
WDYHV1	B cell	38.50%	2.90%	0.39	7.93E-09
TUBA4A	B cell	76.90%	11.60%	0.94	8.34E-09
SDF2L1	B cell	100.00%	27.20%	1.46	1.36E-08
MGAT2	B cell	84.60%	14.40%	0.68	1.47E-08
TNFRSF18	B cell	46.20%	4.30%	0.64	1.64E-08
HMCES	B cell	69.20%	9.50%	0.66	1.89E-08
KLF13	B cell	84.60%	15.40%	0.91	1.95E-08
GLTPD1	B cell	38.50%	3.00%	0.26	3.89E-08
LMAN1	B cell	100.00%	27.30%	1.03	4.74E-08
MDK	B cell	38.50%	3.10%	0.46	5.48E-08
MTERFD2	B cell	61.50%	7.30%	0.36	7.58E-08
DHRS9	B cell	53.80%	6.20%	0.95	9.78E-08
CAPN10-AS1	B cell	30.80%	2.00%	0.39	9.79E-08
TRABD	B cell	84.60%	15.50%	0.67	3.55E-07
CXXC5	B cell	69.20%	10.50%	0.61	4.52E-07
LRWD1	B cell	38.50%	3.30%	0.32	5.88E-07
JMJD4	B cell	30.80%	2.10%	0.29	6.42E-07
CADM1	B cell	46.20%	4.80%	0.38	6.64E-07
NUCB2	B cell	84.60%	19.40%	1.23	7.29E-07
ISOC1	B cell	61.50%	8.40%	0.55	7.74E-07
HIST1H2BC	B cell	15.40%	0.50%	0.26	8.49E-07
STAP1	B cell	15.40%	0.50%	0.57	9.40E-07
TIFA	B cell	69.20%	11.40%	0.74	1.03E-06
EDEM2	B cell	69.20%	11.00%	0.63	1.04E-06
P2RX1	B cell	23.10%	1.30%	0.33	1.08E-06
CHAC1	B cell	46.20%	5.10%	0.70	1.30E-06
SPATS2	B cell	61.50%	9.00%	0.62	1.75E-06
NUDT22	B cell	69.20%	10.60%	0.44	2.69E-06
ABHD5	B cell	84.60%	18.60%	1.42	2.93E-06
PARM1	B cell	23.10%	1.30%	0.35	4.12E-06
PYCRL	B cell	23.10%	1.30%	0.27	4.49E-06
PPP1CC	B cell	92.30%	21.00%	0.71	5.08E-06
XBP1	B cell	100.00%	51.50%	2.19	6.74E-06
SSR3	B cell	100.00%	49.70%	1.34	6.95E-06
SYTL3	B cell	61.50%	9.00%	0.43	7.22E-06
SSR4	B cell	100.00%	73.30%	3.21	7.63E-06
RNF34	B cell	53.80%	6.70%	0.28	8.04E-06

SMOX	B cell	53.80%	7.40%	0.80	8.69E-06
NPPC	B cell	15.40%	0.60%	0.50	9.80E-06
LINC-PINT	B cell	84.60%	17.20%	0.56	1.06E-05
FKBP2	B cell	100.00%	56.30%	1.65	1.07E-05
LINC00152	B cell	100.00%	36.80%	1.97	1.17E-05
HLA-C	B cell	100.00%	97.00%	2.17	1.19E-05
CAV1	B cell	76.90%	14.70%	0.77	1.33E-05
LINC00649	B cell	30.80%	2.50%	0.50	1.40E-05
SYVN1	B cell	53.80%	7.40%	0.43	1.69E-05
CHST2	B cell	38.50%	3.90%	0.48	2.02E-05
ANKRD37	B cell	76.90%	17.60%	1.38	2.28E-05
GADD45A	B cell	92.30%	31.50%	1.61	2.37E-05
SCYL1	B cell	69.20%	12.70%	0.65	2.57E-05
APOBEC3A	B cell	23.10%	1.50%	0.54	2.63E-05
GGH	B cell	53.80%	7.50%	0.41	2.72E-05
DNAJB9	B cell	92.30%	31.30%	1.32	2.75E-05
SLC1A4	B cell	46.20%	5.60%	0.38	3.59E-05
SELM	B cell	100.00%	41.10%	1.09	4.37E-05
ZEB1	B cell	46.20%	5.60%	0.25	4.55E-05
SPCS3	B cell	100.00%	33.20%	1.19	4.82E-05
KLHL6	B cell	46.20%	5.70%	0.43	5.00E-05
HLA-B	B cell	100.00%	99.20%	1.20	6.05E-05
TXNDC15	B cell	76.90%	17.90%	0.98	6.07E-05
ICAM2	B cell	46.20%	5.90%	0.49	6.95E-05
IFNAR2	B cell	69.20%	12.70%	0.51	7.27E-05
SPP1	B cell	69.20%	12.40%	1.27	7.42E-05
HERPUD1	B cell	100.00%	71.20%	2.37	8.53E-05
DERL1	B cell	84.60%	22.30%	0.81	9.47E-05
RRBP1	B cell	100.00%	42.40%	1.19	1.24E-04
OGFOD3	B cell	53.80%	8.70%	0.66	1.32E-04
NME1	B cell	84.60%	23.30%	0.93	1.42E-04
SLC38A10	B cell	69.20%	14.40%	0.65	1.46E-04
HIPK2	B cell	53.80%	8.20%	0.42	1.54E-04
SSTR2	B cell	23.10%	1.60%	0.37	1.84E-04
PTPN7	B cell	38.50%	4.40%	0.43	2.03E-04
ZNF593	B cell	76.90%	16.20%	0.52	2.17E-04
SERP1	B cell	100.00%	73.60%	1.14	2.24E-04
CDC37L1	B cell	53.80%	8.50%	0.77	2.51E-04
ARID5B	B cell	100.00%	35.50%	1.26	2.74E-04
EFCAB4A	B cell	38.50%	4.40%	0.27	3.02E-04
YIF1A	B cell	92.30%	23.50%	0.64	3.05E-04

APOBEC3F	B cell	23.10%	1.70%	0.39	3.44E-04
PSAT1	B cell	15.40%	0.70%	0.28	3.60E-04
HSD17B8	B cell	46.20%	6.30%	0.38	3.83E-04
HIST1H4H	B cell	23.10%	1.70%	0.32	4.02E-04
TMEM59	B cell	100.00%	69.90%	1.24	4.18E-04
SEC24A	B cell	69.20%	13.50%	0.62	4.62E-04
DHDDS	B cell	46.20%	6.10%	0.28	4.75E-04
RABAC1	B cell	100.00%	62.30%	1.20	6.07E-04
NT5C3A	B cell	61.50%	11.00%	0.39	7.52E-04
GAMT	B cell	46.20%	6.50%	0.32	7.65E-04
UBE2J1	B cell	69.20%	16.10%	0.79	1.10E-03
PIGP	B cell	76.90%	17.80%	0.49	1.30E-03
FNDC3A	B cell	84.60%	22.30%	0.73	1.38E-03
TMEM208	B cell	92.30%	28.20%	0.73	1.42E-03
CDKN1B	B cell	61.50%	11.90%	0.54	1.51E-03
SPCS1	B cell	100.00%	50.40%	1.10	1.53E-03
SPCS2	B cell	100.00%	56.30%	1.16	2.34E-03
ANKRD39	B cell	38.50%	5.00%	0.33	2.43E-03
SEL1L	B cell	69.20%	16.10%	0.73	3.44E-03
ISCA1	B cell	76.90%	20.50%	0.67	3.81E-03
PHGDH	B cell	23.10%	2.00%	0.29	3.94E-03
FAM136A	B cell	53.80%	9.50%	0.42	4.04E-03
RPLP0	B cell	100.00%	96.70%	1.17	4.10E-03
TM7SF2	B cell	23.10%	2.00%	0.29	4.72E-03
LARP1B	B cell	53.80%	9.50%	0.36	4.78E-03
STXBP6	B cell	23.10%	2.00%	0.40	4.86E-03
CDR1	B cell	23.10%	2.00%	0.26	4.86E-03
PPP3CC	B cell	53.80%	9.30%	0.48	5.25E-03
RPS26	B cell	100.00%	85.00%	1.17	5.56E-03
MANF	B cell	84.60%	32.10%	1.17	6.16E-03
SLAMF7	B cell	38.50%	5.60%	0.53	6.32E-03
CLEC2D	B cell	30.80%	3.60%	0.37	7.14E-03
CKAP4	B cell	46.20%	7.50%	0.32	7.32E-03
CYTIP	B cell	92.30%	39.20%	1.03	9.35E-03
TMEM214	B cell	46.20%	7.50%	0.33	1.05E-02
SIL1	B cell	69.20%	17.70%	0.89	1.06E-02
GNL3	B cell	76.90%	22.90%	0.76	1.14E-02
MRPS31	B cell	46.20%	8.40%	0.72	1.29E-02
RPL3	B cell	100.00%	99.70%	1.19	1.35E-02
UBALD2	B cell	76.90%	19.80%	0.72	1.40E-02
TMEM258	B cell	100.00%	69.80%	1.00	1.55E-02

GFPT1	B cell	53.80%	10.30%	0.43	1.73E-02
SELK	B cell	100.00%	69.20%	1.14	1.76E-02
ERGIC2	B cell	69.20%	18.30%	0.71	1.90E-02
ABHD14A	B cell	53.80%	10.30%	0.46	2.06E-02
CHID1	B cell	92.30%	28.60%	0.65	2.13E-02
ARID5A	B cell	84.60%	31.90%	0.69	2.24E-02
ARF4	B cell	100.00%	50.20%	0.83	2.28E-02
ALG5	B cell	76.90%	21.80%	0.65	2.36E-02
YKT6	B cell	61.50%	12.90%	0.40	2.44E-02
NEDD4L	B cell	38.50%	6.30%	0.72	2.62E-02
TMED4	B cell	84.60%	27.10%	0.71	2.64E-02
PNKP	B cell	61.50%	13.50%	0.40	2.94E-02
ANKRD44	B cell	46.20%	7.80%	0.27	3.17E-02
RPLP1	B cell	100.00%	100.00%	0.92	3.38E-02
RPL7A	B cell	100.00%	99.00%	1.02	3.45E-02
MRPL3	B cell	69.20%	16.70%	0.44	3.49E-02
NRROS	B cell	53.80%	10.30%	0.36	3.53E-02
DESI1	B cell	53.80%	10.40%	0.28	4.26E-02
DDOST	B cell	84.60%	27.80%	0.69	4.28E-02
SENP2	B cell	38.50%	5.90%	0.33	4.36E-02
OST4	B cell	100.00%	78.30%	0.88	4.47E-02
PPA1	B cell	76.90%	22.50%	0.64	4.74E-02
LMAN2	B cell	100.00%	39.60%	0.90	5.28E-02
C16orf54	B cell	30.80%	4.10%	0.34	5.34E-02
PRDM1	B cell	84.60%	27.00%	1.10	5.74E-02
EEF2	B cell	100.00%	90.60%	0.87	6.06E-02
HN1	B cell	84.60%	24.50%	0.49	6.12E-02
UBQLN2	B cell	46.20%	8.10%	0.27	6.39E-02
EXOSC1	B cell	46.20%	8.40%	0.30	6.48E-02
TMEM134	B cell	69.20%	17.90%	0.57	6.49E-02
SSR2	B cell	100.00%	55.80%	0.91	6.66E-02
CUTA	B cell	92.30%	55.70%	0.94	7.23E-02
IARS	B cell	53.80%	11.30%	0.34	7.76E-02
KDELR1	B cell	100.00%	43.70%	0.66	8.33E-02
ATF4	B cell	100.00%	85.30%	0.87	8.74E-02
TAPSAR1	B cell	46.20%	8.70%	0.34	9.60E-02
UFM1	B cell	92.30%	37.50%	0.84	1.00E-01
VIMP	B cell	84.60%	45.70%	0.90	1.08E-01
CLU	B cell	100.00%	69.60%	0.62	1.12E-01
PRELP	B cell	84.60%	27.20%	0.43	1.13E-01
CTNNAL1	B cell	53.80%	11.90%	0.44	1.25E-01

RPN2	B cell	92.30%	41.70%	0.79	1.28E-01
LRRC59	B cell	84.60%	28.50%	0.49	1.30E-01
SEC61B	B cell	92.30%	73.10%	1.10	1.35E-01
BCAR3	B cell	46.20%	9.50%	0.54	1.42E-01
P2RY10	B cell	15.40%	1.20%	0.28	1.49E-01
MRPL17	B cell	69.20%	17.60%	0.37	1.54E-01
CREB3	B cell	69.20%	18.50%	0.41	1.58E-01
SEC61A1	B cell	61.50%	16.20%	0.50	1.68E-01
ERGIC3	B cell	92.30%	46.50%	0.87	1.74E-01
CNPY2	B cell	84.60%	30.00%	0.64	1.79E-01
VKORC1	B cell	92.30%	38.80%	0.76	1.97E-01
SURF1	B cell	92.30%	29.60%	0.47	2.17E-01
RPS6KB2	B cell	61.50%	15.40%	0.39	2.19E-01
EEF1D	B cell	100.00%	94.80%	0.82	2.28E-01
ST6GAL1	B cell	53.80%	12.90%	0.52	2.30E-01
RPL36AL	B cell	100.00%	93.30%	0.73	2.40E-01
NDUFA4L2	B cell	76.90%	21.10%	0.29	2.48E-01
C19orf10	B cell	84.60%	45.40%	1.19	2.53E-01
TRIM27	B cell	46.20%	9.10%	0.29	2.57E-01
KRTCAP2	B cell	92.30%	42.00%	0.73	2.71E-01
APOBEC3C	B cell	69.20%	18.90%	0.42	2.77E-01
ARSA	B cell	46.20%	9.30%	0.35	2.79E-01
HAGH	B cell	69.20%	19.80%	0.48	3.13E-01
SRPR	B cell	61.50%	18.50%	0.63	3.31E-01
CALCOCO1	B cell	46.20%	9.20%	0.26	3.43E-01
METTL1	B cell	30.80%	4.70%	0.28	3.54E-01
RPS4X	B cell	100.00%	99.60%	0.85	3.60E-01
ARFGAP1	B cell	38.50%	6.80%	0.25	3.74E-01
NCLN	B cell	38.50%	7.30%	0.44	3.78E-01
NUS1	B cell	61.50%	14.80%	0.38	4.36E-01
EEF1B2	B cell	100.00%	93.90%	0.81	4.39E-01
RPS19BP1	B cell	92.30%	38.00%	0.68	4.46E-01
TTPAL	B cell	23.10%	2.90%	0.42	4.63E-01
IFI27L1	B cell	38.50%	7.40%	0.39	4.98E-01
MTUS1	B cell	30.80%	4.90%	0.30	4.99E-01
NDUFB11	B cell	100.00%	64.90%	0.71	5.08E-01
HTRA2	B cell	46.20%	10.70%	0.48	5.22E-01
SLC25A6	B cell	100.00%	89.40%	1.07	5.38E-01
SAMSN1	B cell	76.90%	22.90%	0.42	5.48E-01
CCNC	B cell	61.50%	15.80%	0.34	5.49E-01
IGF1	B cell	61.50%	18.40%	0.68	5.52E-01

WDR45	B cell	61.50%	19.40%	0.70	5.64E-01
UBALD1	B cell	46.20%	9.50%	0.28	5.75E-01
PGM3	B cell	38.50%	7.80%	0.47	5.75E-01
ANKRD28	B cell	92.30%	51.40%	0.95	5.78E-01
PRDX2	B cell	84.60%	29.00%	0.55	5.82E-01
FBXW7	B cell	46.20%	9.50%	0.27	5.86E-01
APOBEC3G	B cell	38.50%	7.50%	0.37	6.22E-01
C12orf57	B cell	100.00%	38.80%	0.78	6.45E-01
MGMT	B cell	69.20%	21.90%	0.56	6.53E-01
MCFD2	B cell	61.50%	15.70%	0.31	7.40E-01
MPG	B cell	76.90%	23.50%	0.47	7.54E-01
RP11-138A9.2	B cell	53.80%	12.70%	0.34	7.68E-01
SELT	B cell	100.00%	57.40%	0.66	8.08E-01
RPS5	B cell	100.00%	98.40%	0.72	9.14E-01
MSI2	B cell	38.50%	7.50%	0.35	9.18E-01
PARP16	B cell	23.10%	3.00%	0.30	9.21E-01
MPZL1	B cell	53.80%	13.00%	0.30	9.29E-01
SCO2	B cell	53.80%	14.50%	0.44	9.41E-01
KIAA0895L	B cell	15.40%	1.40%	0.26	9.69E-01
MIR4435-1HG	B cell	61.50%	22.30%	1.10	9.82E-01
P4HB	B cell	100.00%	51.00%	0.62	1.00E+00
CHMP7	B cell	38.50%	7.50%	0.28	1.00E+00
AIG1	B cell	53.80%	14.70%	0.43	1.00E+00
CCDC69	B cell	38.50%	7.50%	0.28	1.00E+00
CSN1S1	B cell	38.50%	7.30%	0.31	1.00E+00
PRADC1	B cell	46.20%	10.40%	0.29	1.00E+00
BTG2	B cell	92.30%	46.50%	0.83	1.00E+00
GORASP2	B cell	53.80%	13.00%	0.28	1.00E+00
SERPINB9	B cell	92.30%	43.00%	0.62	1.00E+00
RPS19	B cell	100.00%	99.70%	0.67	1.00E+00
MTHFS	B cell	38.50%	7.90%	0.36	1.00E+00
ZNF331	B cell	69.20%	20.80%	0.37	1.00E+00
RPL4	B cell	100.00%	95.00%	0.73	1.00E+00
AC006129.4	B cell	23.10%	3.10%	1.40	1.00E+00
DPM2	B cell	61.50%	16.10%	0.26	1.00E+00
UROS	B cell	53.80%	13.50%	0.31	1.00E+00
TPT1	B cell	100.00%	99.90%	0.73	1.00E+00
LGALSL	B cell	30.80%	5.40%	0.28	1.00E+00
CRELD2	B cell	53.80%	13.80%	0.33	1.00E+00
RPL8	B cell	100.00%	99.70%	0.55	1.00E+00
PIK3CG	B cell	30.80%	5.30%	0.28	1.00E+00

PHPT1	B cell	100.00%	49.30%	0.63	1.00E+00
C10orf54	B cell	84.60%	35.60%	0.54	1.00E+00
NDUFS8	B cell	84.60%	37.30%	0.62	1.00E+00
MZT2B	B cell	92.30%	38.90%	0.52	1.00E+00
CISD2	B cell	76.90%	27.10%	0.53	1.00E+00
TMEM57	B cell	53.80%	14.10%	0.30	1.00E+00
ALG3	B cell	53.80%	13.90%	0.30	1.00E+00
EDF1	B cell	100.00%	78.30%	0.58	1.00E+00
B4GALT3	B cell	30.80%	5.60%	0.26	1.00E+00
MRPL53	B cell	30.80%	5.50%	0.27	1.00E+00
MCEE	B cell	30.80%	5.80%	0.38	1.00E+00
SRM	B cell	30.80%	5.60%	0.26	1.00E+00
PELI1	B cell	84.60%	45.30%	1.19	1.00E+00
MRPL34	B cell	84.60%	29.90%	0.41	1.00E+00
TMED9	B cell	92.30%	56.50%	0.60	1.00E+00
FAM3C	B cell	76.90%	24.50%	0.33	1.00E+00
TRAM1	B cell	92.30%	44.90%	0.57	1.00E+00
APOA1BP	B cell	53.80%	14.20%	0.42	1.00E+00
SSSCA1	B cell	46.20%	11.00%	0.25	1.00E+00
RPL41	B cell	100.00%	100.00%	0.50	1.00E+00
DCXR	B cell	61.50%	20.10%	0.42	1.00E+00
AURKAIP1	B cell	92.30%	41.30%	0.48	1.00E+00
THAP2	B cell	61.50%	23.00%	1.21	1.00E+00
RPS11	B cell	100.00%	99.60%	0.55	1.00E+00
TRAF3	B cell	38.50%	8.30%	0.27	1.00E+00
FASTK	B cell	53.80%	14.70%	0.30	1.00E+00
SUB1	B cell	100.00%	72.40%	0.74	1.00E+00
ZNF706	B cell	92.30%	58.50%	0.56	1.00E+00
MRPL2	B cell	46.20%	11.80%	0.29	1.00E+00
TMEM256	B cell	84.60%	41.10%	0.59	1.00E+00
EIF2AK3	B cell	38.50%	8.70%	0.37	1.00E+00
WDR83	B cell	30.80%	6.00%	0.25	1.00E+00
MTHFD2	B cell	84.60%	37.70%	0.57	1.00E+00
AMTN	B cell	23.10%	3.60%	0.25	1.00E+00
SHMT2	B cell	53.80%	15.30%	0.34	1.00E+00
ARMC9	B cell	23.10%	3.70%	0.33	1.00E+00
TSC22D3	B cell	100.00%	72.80%	0.85	1.00E+00
TRAM2	B cell	30.80%	6.40%	0.71	1.00E+00
IFT20	B cell	61.50%	20.10%	0.48	1.00E+00
NMRK1	B cell	46.20%	11.70%	0.26	1.00E+00
GNL1	B cell	53.80%	16.10%	0.36	1.00E+00

IFT57	B cell	46.20%	12.20%	0.32	1.00E+00
GALNT2	B cell	46.20%	11.90%	0.33	1.00E+00
TNIP1	B cell	84.60%	33.40%	0.48	1.00E+00
TMEM19	B cell	46.20%	12.10%	0.30	1.00E+00
ELL2	B cell	76.90%	32.50%	0.53	1.00E+00
TMED10	B cell	92.30%	46.90%	0.44	1.00E+00
NGLY1	B cell	46.20%	12.20%	0.29	1.00E+00
SEC24D	B cell	53.80%	15.30%	0.28	1.00E+00
RPSA	B cell	100.00%	95.70%	0.67	1.00E+00
ODF2L	B cell	30.80%	6.20%	0.27	1.00E+00
NDUFV3	B cell	53.80%	16.70%	0.47	1.00E+00
HM13	B cell	92.30%	35.90%	0.45	1.00E+00
COPE	B cell	92.30%	70.90%	0.57	1.00E+00
COMMD3	B cell	76.90%	27.90%	0.38	1.00E+00
GTF3A	B cell	84.60%	40.50%	0.65	1.00E+00
RPS14	B cell	100.00%	99.90%	0.63	1.00E+00
RNF126	B cell	46.20%	12.70%	0.32	1.00E+00
BEX5	B cell	15.40%	1.90%	0.44	1.00E+00
EIF2S2	B cell	92.30%	55.10%	0.50	1.00E+00
A1BG	B cell	61.50%	19.30%	0.29	1.00E+00
WARS	B cell	46.20%	13.20%	0.45	1.00E+00
CCM2	B cell	46.20%	12.50%	0.32	1.00E+00
RPL10	B cell	100.00%	100.00%	0.61	1.00E+00
ARMCX3	B cell	69.20%	26.00%	0.44	1.00E+00
IDI1	B cell	76.90%	36.90%	0.67	1.00E+00
SIAE	B cell	15.40%	1.90%	0.28	1.00E+00
MRPS33	B cell	53.80%	16.40%	0.34	1.00E+00
ATG13	B cell	30.80%	6.60%	0.26	1.00E+00
ERP29	B cell	100.00%	56.60%	0.41	1.00E+00
TMEM45A	B cell	30.80%	6.80%	0.35	1.00E+00
PDIA4	B cell	84.60%	29.70%	0.26	1.00E+00
ITGA6	B cell	30.80%	6.70%	0.29	1.00E+00
ANXA6	B cell	53.80%	16.10%	0.29	1.00E+00
KLHDC2	B cell	46.20%	12.60%	0.27	1.00E+00
SQSTM1	B cell	100.00%	86.50%	0.93	1.00E+00
DNAJC4	B cell	53.80%	18.80%	0.59	1.00E+00
RPL23A	B cell	100.00%	99.50%	0.52	1.00E+00
PREB	B cell	38.50%	10.30%	0.39	1.00E+00
WHSC1L1	B cell	61.50%	21.40%	0.44	1.00E+00
STK16	B cell	38.50%	9.80%	0.29	1.00E+00
RPL18	B cell	100.00%	99.20%	0.60	1.00E+00

HDAC9	B cell	38.50%	9.50%	0.33	1.00E+00
SEC61G	B cell	92.30%	70.90%	0.56	1.00E+00
INSIG2	B cell	38.50%	9.80%	0.33	1.00E+00
IGSF8	B cell	38.50%	9.90%	0.29	1.00E+00
CCDC22	B cell	30.80%	6.60%	0.29	1.00E+00
C12orf23	B cell	38.50%	10.30%	0.38	1.00E+00
CDK4	B cell	61.50%	20.40%	0.34	1.00E+00
SEC13	B cell	69.20%	24.10%	0.29	1.00E+00
HAX1	B cell	76.90%	29.80%	0.43	1.00E+00
NHP2L1	B cell	84.60%	56.00%	0.60	1.00E+00
ACP1	B cell	76.90%	28.80%	0.39	1.00E+00
MAN1A2	B cell	46.20%	14.50%	0.43	1.00E+00
MALAT1	B cell	100.00%	100.00%	0.58	1.00E+00
CYBA	B cell	100.00%	98.10%	0.98	1.00E+00
CCDC57	B cell	38.50%	10.10%	0.29	1.00E+00
DDIT4	B cell	84.60%	52.20%	0.86	1.00E+00
ARFGAP3	B cell	76.90%	32.30%	0.42	1.00E+00
ATRAID	B cell	92.30%	51.30%	0.46	1.00E+00
OSTC	B cell	84.60%	42.60%	0.58	1.00E+00
RP11-138A9.1	B cell	61.50%	20.70%	0.28	1.00E+00
C19orf38	B cell	23.10%	4.30%	0.28	1.00E+00
RPL13A	B cell	100.00%	100.00%	0.52	1.00E+00
PRELID1	B cell	84.60%	41.10%	0.53	1.00E+00
RPL35	B cell	100.00%	99.60%	0.40	1.00E+00
CCPG1	B cell	76.90%	32.80%	0.43	1.00E+00
DNAJC3	B cell	76.90%	35.60%	0.48	1.00E+00
CIRBP	B cell	100.00%	75.30%	0.56	1.00E+00
RPS18	B cell	100.00%	99.90%	0.58	1.00E+00
ITPR3	B cell	23.10%	4.60%	0.31	1.00E+00
PTPN1	B cell	84.60%	36.40%	0.36	1.00E+00
UAP1	B cell	84.60%	32.60%	0.26	1.00E+00
GCHFR	B cell	46.20%	14.90%	0.37	1.00E+00
TRIB1	B cell	61.50%	32.40%	1.00	1.00E+00
LY96	B cell	92.30%	52.40%	0.43	1.00E+00
MRPS11	B cell	46.20%	13.90%	0.29	1.00E+00
RAB27A	B cell	30.80%	7.50%	0.26	1.00E+00
MIR155HG	B cell	69.20%	31.50%	0.75	1.00E+00
UFC1	B cell	92.30%	49.00%	0.47	1.00E+00
RPL18A	B cell	100.00%	99.70%	0.59	1.00E+00
NXT1	B cell	53.80%	18.10%	0.29	1.00E+00
SCFD1	B cell	53.80%	20.00%	0.40	1.00E+00

TMED3	B cell	53.80%	18.80%	0.35	1.00E+00
RPS3	B cell	100.00%	99.60%	0.41	1.00E+00
RPL5	B cell	100.00%	98.70%	0.61	1.00E+00
RPL14	B cell	100.00%	99.10%	0.39	1.00E+00
TMEM243	B cell	61.50%	25.50%	0.52	1.00E+00
MAGED2	B cell	61.50%	24.40%	0.45	1.00E+00
PSENEN	B cell	84.60%	39.70%	0.34	1.00E+00
MARS	B cell	38.50%	11.30%	0.29	1.00E+00
RPS8	B cell	100.00%	99.70%	0.48	1.00E+00
NDUFA3	B cell	76.90%	36.20%	0.42	1.00E+00
TLK1	B cell	38.50%	11.00%	0.25	1.00E+00
PQBP1	B cell	69.20%	28.40%	0.32	1.00E+00
PPIB	B cell	92.30%	73.00%	0.54	1.00E+00
RNASEH2C	B cell	69.20%	26.10%	0.29	1.00E+00
MZT2A	B cell	46.20%	15.80%	0.32	1.00E+00
SRA1	B cell	61.50%	22.40%	0.29	1.00E+00
GUSB	B cell	69.20%	27.70%	0.42	1.00E+00
MRPL52	B cell	84.60%	40.60%	0.47	1.00E+00
STX5	B cell	61.50%	25.20%	0.39	1.00E+00
JTB	B cell	84.60%	58.10%	0.59	1.00E+00
PSMD2	B cell	69.20%	27.20%	0.33	1.00E+00
SLC7A5	B cell	53.80%	18.10%	0.32	1.00E+00
BHLHE41	B cell	38.50%	11.40%	0.47	1.00E+00
COA1	B cell	38.50%	11.50%	0.25	1.00E+00
VOPP1	B cell	76.90%	37.00%	0.60	1.00E+00
FAM207A	B cell	30.80%	8.40%	0.30	1.00E+00
IFI35	B cell	46.20%	15.20%	0.27	1.00E+00
AKAP9	B cell	76.90%	46.10%	0.49	1.00E+00
C1orf122	B cell	61.50%	24.50%	0.31	1.00E+00
MRPL55	B cell	61.50%	24.00%	0.36	1.00E+00
EHMT1	B cell	38.50%	11.30%	0.28	1.00E+00
NANS	B cell	84.60%	44.40%	0.45	1.00E+00
RPL27A	B cell	100.00%	99.90%	0.35	1.00E+00
PCGF5	B cell	53.80%	21.40%	0.47	1.00E+00
RPL24	B cell	100.00%	98.60%	0.43	1.00E+00
NOSIP	B cell	61.50%	22.90%	0.29	1.00E+00
RPL15	B cell	100.00%	100.00%	0.38	1.00E+00
RPS28	B cell	100.00%	100.00%	0.50	1.00E+00
ATP5A1	B cell	92.30%	54.80%	0.35	1.00E+00
C7orf50	B cell	53.80%	22.30%	0.41	1.00E+00
BSG	B cell	100.00%	60.10%	0.36	1.00E+00

DNAJC10	B cell	46.20%	17.00%	0.31	1.00E+00
GOLGA2	B cell	53.80%	18.90%	0.26	1.00E+00
DAPP1	B cell	38.50%	12.70%	0.39	1.00E+00
DPP7	B cell	84.60%	58.00%	0.64	1.00E+00
C12orf44	B cell	69.20%	28.80%	0.38	1.00E+00
SARS	B cell	69.20%	30.30%	0.30	1.00E+00
EMC4	B cell	69.20%	28.90%	0.29	1.00E+00
PDE4B	B cell	76.90%	35.50%	0.39	1.00E+00
RPL35A	B cell	100.00%	99.70%	0.39	1.00E+00
RPL13	B cell	100.00%	100.00%	0.35	1.00E+00
OPTN	B cell	46.20%	15.90%	0.32	1.00E+00
NARF	B cell	46.20%	16.80%	0.61	1.00E+00
FNBP1	B cell	76.90%	40.40%	0.48	1.00E+00
HDLBP	B cell	84.60%	40.90%	0.41	1.00E+00
RNF7	B cell	84.60%	37.30%	0.30	1.00E+00
GTF2E1	B cell	15.40%	2.70%	0.93	1.00E+00
UQCRH	B cell	100.00%	72.30%	0.53	1.00E+00
PSMB8	B cell	61.50%	27.30%	0.36	1.00E+00
RPL6	B cell	100.00%	99.30%	0.43	1.00E+00
PDIA6	B cell	76.90%	52.00%	0.58	1.00E+00
PRMT1	B cell	69.20%	29.10%	0.28	1.00E+00
CLPTM1L	B cell	38.50%	12.60%	0.31	1.00E+00
RPL22L1	B cell	84.60%	44.60%	0.46	1.00E+00
ASCC3	B cell	30.80%	9.00%	0.25	1.00E+00
DOK3	B cell	23.10%	5.70%	0.28	1.00E+00
ANKRD36	B cell	46.20%	16.30%	0.37	1.00E+00
RAC2	B cell	53.80%	20.80%	0.26	1.00E+00
MRPS18B	B cell	46.20%	16.30%	0.29	1.00E+00
GYPC	B cell	92.30%	44.10%	0.30	1.00E+00
DAD1	B cell	100.00%	62.30%	0.37	1.00E+00
SPSB3	B cell	53.80%	20.90%	0.28	1.00E+00
ZBTB38	B cell	69.20%	34.60%	0.58	1.00E+00
RBM33	B cell	38.50%	12.50%	0.37	1.00E+00
TPST2	B cell	46.20%	18.80%	0.37	1.00E+00
RPL10A	B cell	100.00%	99.10%	0.44	1.00E+00
RSL24D1	B cell	84.60%	54.80%	0.44	1.00E+00
NSMCE2	B cell	38.50%	13.20%	0.28	1.00E+00
THEMIS2	B cell	61.50%	25.20%	0.28	1.00E+00
SEC63	B cell	61.50%	27.40%	0.48	1.00E+00
RPL9	B cell	100.00%	99.70%	0.34	1.00E+00
RPLP2	B cell	100.00%	100.00%	0.34	1.00E+00

XRN1	B cell	38.50%	13.70%	0.33	1.00E+00
ISCU	B cell	92.30%	51.50%	0.42	1.00E+00
YIPF3	B cell	69.20%	30.00%	0.29	1.00E+00
GLG1	B cell	46.20%	17.90%	0.43	1.00E+00
MPC2	B cell	84.60%	44.10%	0.35	1.00E+00
TMEM141	B cell	46.20%	17.50%	0.31	1.00E+00
TAGAP	B cell	53.80%	21.90%	0.37	1.00E+00
RP11-386I14.4	B cell	46.20%	17.90%	0.34	1.00E+00
LDHB	B cell	53.80%	26.70%	0.57	1.00E+00
TOP1	B cell	76.90%	43.50%	0.39	1.00E+00
UBE2G1	B cell	46.20%	17.40%	0.27	1.00E+00
MAGEH1	B cell	30.80%	9.50%	0.26	1.00E+00
GLRX	B cell	92.30%	72.40%	0.53	1.00E+00

Supplementary Table S2. Evaluation of existing bulk gene expression data from OA and control synovial for key markers of pathogenic subsets in OA based on scRNA-seq.

Dataset<br>Reference	HLA-DRA	HLA-DQA1	HLA-DQA2	OLR1	TLR2	Source of control synovium	Comments
**GSE1919**<br>Ungethuem, U. et al. Molecular signatures and new candidates to target the pathogenesis of rheumatoid arthritis. Physiological genomics 42a, 267-282, doi:10.1152/physiolgenomics.00004.2010 (2010). PMID: 20858714	No difference (p > 0.05)<br>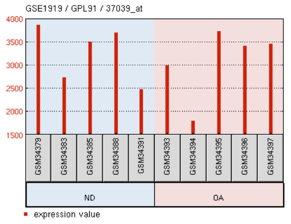	High in OA, low in ND<br>logFC=1.72, p=0.023<br>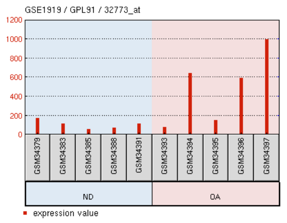	No data	High in OA, low in ND<br>LogFC=2.13, p=0.0047<br>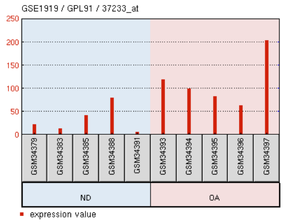	No difference (p > 0.05)<br>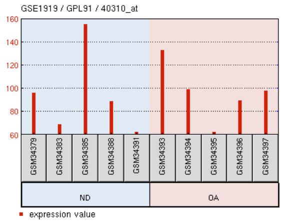	72 synovia from a tissue bank of fatal accident victims	Time from death to acquisition and storage not indicated; Individuals had “no or slightly inflamed synovial membranes”; according to these data, relative to normal donors, some canonical cytokines, such as TNFalpha were downregulated in rheumatoid arthritis (RA)
**GSE41038**<br>Thomas, G. P. *et al.* Expression profiling in spondyloarthropathy synovial biopsies highlights changes in expression of inflammatory genes in conjunction with tissue remodelling genes. *BMC Musculoskelet Disord* **14**, 354, doi:10.1186/1471-2474-14-354 (2013). PMID: 24330574	No difference (p > 0.05)<br>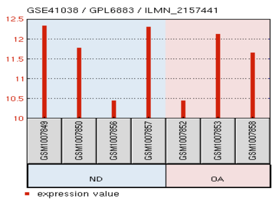	No data	No difference (p > 0.05)<br>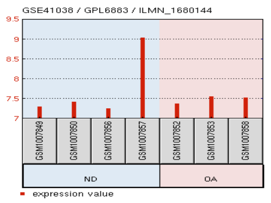	No difference (p > 0.05)<br>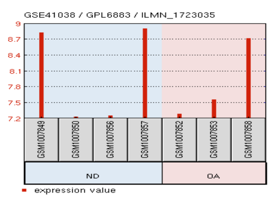	No difference (p > 0.05)<br>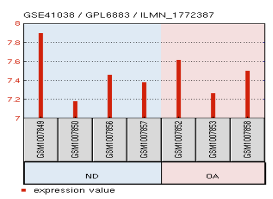	4 normal controls from a synovial tissue bank	Means of acquisition by arthroscopic; clinical indication for arthroscopy not specified; no report of macroscopic investigation of cartilage of controls; not specified whether tissues were from a non-arthritic/non-trauma individual or from a macroscopically normal region of synovium of a patient with indication for arthroscopic procedure
**GSE55457**<br>Woetzel, D. *et al.* Identification of rheumatoid arthritis and osteoarthritis patients by transcriptome-based rule set generation. *Arthritis Res Ther* **16**, R84, doi:10.1186/ar4526 (2014). PMID: 24690414	No difference (p > 0.05)<br>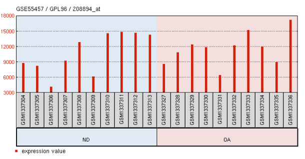	High in OA, low in ND<br>logFC=2.88, p=0.014<br>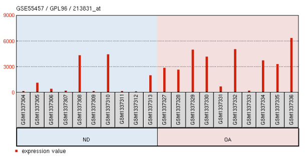	High in OA, low in ND<br>logFC=0.97, p=0.042<br>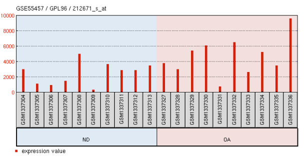	No difference (p > 0.05)<br>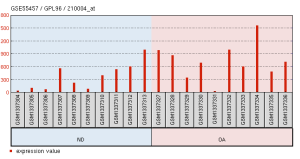	No difference (p > 0.05)<br>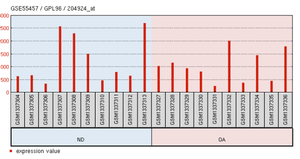	10 normal donors and 10 OA	Synovial membrane samples were obtained from postmortem joints (n=15) or traumatic joint injury cases (n = 5); Time from death or injury to acquisition and storage not indicated
**GSE55235**<br>Woetzel, D. *et al.* Identification of rheumatoid arthritis and osteoarthritis patients by transcriptome-based rule set generation. *Arthritis Res Ther* **16**, R84, doi:10.1186/ar4526 (2014). PMID: 24690414	High in OA, low in ND<br>logFC=1.56, p= 1.92E-03<br>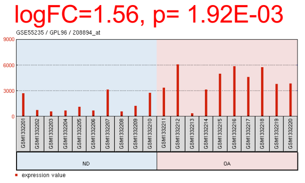	High in OA, low in ND<br>logFC=2.57, p= 9.68E-05<br>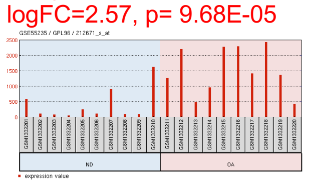	High in OA, low in ND<br>logFC=2.57, p= 9.68E-05<br>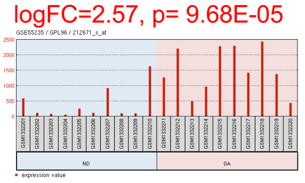	High in OA, low in ND<br>LogFC=2.33, p=1.89E-06<br>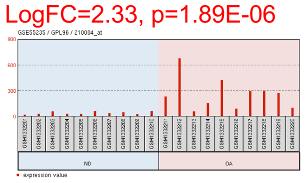	No difference (p > 0.05)<br>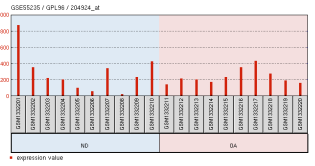	10 normal donors and 10 OA	As described above in table, datasets used each have major confounds with respect to the condition of normal or control synovium
Data for 896 differentially expressed genes at http://onlinelibrary.wiley.com/doi/10.1002/art.38315 see **ART_38315_sm_SupplTable.docx**<br>Lambert, C. *et al.* Gene expression pattern of cells from inflamed and normal areas of osteoarthritis synovial membrane. *Arthritis Rheumatol* **66**, 960-968, doi:10.1002/art.38315 (2014). PMID: 24757147	inflamed vs matched non-inflamed synovia<br>FC = 3.25 to 4.51	inflamed vs matched non-inflamed synovia<br>FC = 2.71	No data	No difference	No difference	12 knee OA synovial specimens from knee replacement comparing inflamed vs normal synovia regions within each patient	The first to identify different expression patterns between 2 areas of the synovial membrane from the same patient (inflamed vs normal or reactive by Ayral criteria); gene expression performed after 7 days’ cell culture

Profile graphs show expression in normal donor highlighted in blue, expression in OA highlighted in red. OA=osteoarthritic

Supplementary Table S3. List of differentially expressed genes in osteoarthritic chondrocytes from non-damaged and damaged cartilages.

Gene	The percentage of cells expressing the gene in MT	The percentage of cells expressing the gene in oLT	Log fold-change of the average expression between the two groups	Adjusted p-value (Bonferroni correction)
S100A4	0.909	37.50%	209.05%	0
COL1A1	0.271	2.00%	168.95%	0
COL1A2	0.825	26.70%	164.82%	0
COL3A1	0.966	61.50%	153.62%	0
TMSB4X	0.877	68.20%	150.40%	0
HTRA1	0.985	67.20%	148.73%	0
FN1	1	99.80%	133.98%	0
OGN	0.945	69.20%	129.69%	0
CRTAC1	0.923	59.50%	129.47%	0
TNFRSF11B	0.896	69.90%	121.62%	0
TGFBI	0.673	15.70%	119.77%	0
ASPN	0.802	43.80%	118.75%	0
TNFAIP6	0.383	5.50%	114.13%	0
SERPINE2	0.855	40.90%	107.60%	0
COL6A3	0.811	35.30%	102.24%	0
MT1G	0.883	62.50%	101.15%	0
CD55	0.85	59.70%	98.80%	0
TNC	0.737	31.80%	88.87%	0
TPPP3	0.47	10.90%	88.34%	0
COMP	1	99.80%	86.58%	0
CRLF1	0.503	4.30%	82.48%	0
OMD	0.838	49.90%	78.05%	0
PTGES	0.614	17.10%	77.19%	0
TSPAN2	0.883	55.60%	76.56%	0
COL5A1	0.792	30.20%	74.67%	0
COL6A1	0.904	75.40%	73.71%	0
THY1	0.457	5.00%	72.09%	0
SERPINE1	0.659	21.50%	71.36%	0
S100A6	1	99.90%	71.28%	0
VCAN	0.487	10.00%	70.15%	0
CILP	0.971	84.30%	70.04%	0
ABI3BP	0.713	29.10%	67.46%	0

COL6A2	0.941	81.60%	67.40%	0
IL11	0.143	0.30%	66.45%	0
TAGLN2	0.94	66.00%	65.45%	0
AKR1C2	0.692	25.10%	64.55%	0
PRSS23	0.449	13.70%	64.20%	0
AQP1	0.217	1.70%	61.55%	0
COL15A1	0.606	16.20%	59.82%	0
S100A10	0.999	98.80%	58.98%	0
CILP2	0.856	66.90%	57.41%	0
SMOC1	0.721	27.10%	57.10%	0
CAPS	0.642	19.80%	56.31%	0
NGF	0.467	5.20%	55.99%	0
DKK3	0.639	24.30%	55.97%	0
LUM	0.999	99.80%	55.66%	0
GAS1	0.642	23.50%	55.56%	0
ANKH	0.882	60.50%	53.55%	0
AK1	0.962	77.10%	53.19%	0
SPARC	0.995	95.50%	52.12%	0
SRPX2	0.767	40.20%	51.65%	0
COL5A2	0.831	49.90%	51.51%	0
ANGPTL2	0.984	82.30%	51.39%	0
FSTL1	0.811	51.70%	51.36%	0
PRELP	0.998	99.60%	51.13%	0
MT1F	0.816	57.40%	50.13%	0
DPT	0.518	24.30%	47.14%	0
CD151	0.927	74.90%	47.02%	0
UGP2	0.948	79.70%	46.70%	0
ADAMTS6	0.639	22.40%	46.63%	0
SPP1	0.505	23.40%	46.51%	0
NT5E	0.668	31.40%	46.32%	0
ATP5E	0.99	92.40%	45.05%	0
NBL1	0.3	5.80%	43.87%	0
PART1	0.628	27.70%	43.62%	0
SH3BGRL3	0.99	94.40%	42.64%	0
EMP3	0.975	87.90%	42.39%	0
TNFRSF12A	0.865	55.90%	42.20%	0
PDLIM7	0.537	13.80%	42.13%	0
NOVA1	0.564	24.20%	41.97%	0
FMOD	0.992	99.20%	41.14%	0
CAV1	0.884	66.90%	40.82%	0
CLIC3	0.308	3.50%	40.67%	0

IGFBP7	0.812	39.30%	40.56%	0
LOXL2	0.457	10.40%	40.56%	0
OSTC	0.929	75.30%	40.02%	0
DNER	0.412	4.20%	39.90%	0
IGFBP4	0.385	5.00%	39.69%	0
PID1	0.809	51.00%	39.42%	0
TREM1	0.202	0.50%	38.32%	0
UROC1	0.388	9.00%	38.31%	0
FNIP2	0.574	22.20%	38.07%	0
PFKP	0.834	55.20%	38.04%	0
PMP22	0.958	83.70%	38.02%	0
TIMP2	0.829	61.40%	37.87%	0
MXRA5	0.389	10.50%	37.75%	0
TNXB	0.464	19.60%	37.73%	0
S100A11	0.988	94.10%	37.65%	0
PLP2	0.957	80.60%	36.67%	0
CAPG	0.751	48.20%	36.43%	0
CDA	0.529	21.60%	36.09%	0
ITGB5	0.462	15.30%	35.95%	0
RPL28	1	99.90%	35.91%	0
CD68	0.616	26.10%	35.78%	0
TM4SF1	0.784	48.20%	35.68%	0
AEBP1	0.921	76.70%	35.58%	0
PLAUR	0.511	18.70%	35.42%	0
CCND1	0.279	5.60%	34.96%	0
HCFC1R1	0.784	49.30%	34.80%	0
GAPDH	1	100.00%	34.62%	0
THBS3	0.533	17.70%	33.95%	0
PRKCDBP	0.877	66.90%	33.90%	0
CRIP1	0.496	22.30%	33.40%	0
GNG11	0.709	37.90%	33.27%	0
SLC7A2	0.507	24.90%	33.18%	0
PROCR	0.675	38.50%	32.87%	0
PAPPA	0.216	4.00%	32.81%	0
COX17	0.861	63.00%	32.62%	0
RABAC1	0.993	93.30%	32.49%	0
OAZ1	0.999	99.00%	31.27%	0
LOX	0.539	24.70%	31.20%	0
NDUFB2	0.925	73.90%	31.12%	0
C7orf73	0.898	68.00%	30.87%	0
YIF1A	0.82	54.10%	30.84%	0

VKORC1	0.992	93.40%	30.27%	0
LOXL3	0.536	19.90%	29.96%	0
RPLP0	1	99.90%	29.26%	0
FAP	0.466	16.40%	29.19%	0
ECM1	0.266	5.90%	29.10%	0
PPIC	0.87	61.50%	28.79%	0
S100A2	0.168	2.10%	28.44%	0
ANK3	0.529	20.80%	27.93%	0
LTBP1	0.531	26.30%	27.38%	0
ADAMTS5	0.254	4.80%	27.29%	0
NDUFS8	0.61	31.50%	26.83%	0
PGAM1	0.968	88.00%	26.74%	0
CNIH1	0.804	55.60%	26.70%	0
C10orf105	0.371	6.60%	26.67%	0
CDON	0.624	28.70%	26.46%	0
RPS12	1	100.00%	26.30%	0
HSD3B7	0.272	4.90%	26.27%	0
COX7B	0.919	74.10%	26.26%	0
NDUFA11	0.959	83.80%	26.21%	0
RAB13	0.868	66.10%	26.04%	0
DOK1	0.548	25.70%	25.99%	0
MRC2	0.698	38.30%	25.89%	0
TMEM167A	0.787	52.00%	25.86%	0
ATOX1	0.832	57.70%	25.72%	0
RPS24	1	99.80%	25.56%	0
MPG	0.745	44.80%	25.46%	0
REXO2	0.943	79.60%	25.40%	0
SEC61G	0.991	94.10%	25.38%	0
DIXDC1	0.716	42.50%	25.26%	0
TRPS1	0.702	41.80%	25.21%	0
RP11-572C15.6	0.47	22.50%	25.21%	0
GRN	0.794	56.60%	25.15%	0
LTBP2	0.537	24.50%	25.06%	0
SGK1	0.609	35.70%	27.85%	3.24E-300
RCAN1	0.791	57.00%	34.85%	7.46E-296
CFD	0.111	0.80%	36.78%	3.14E-290
TNNT3	0.448	23.70%	49.71%	1.96E-287
CXCL14	0.132	1.80%	42.93%	3.33E-283
SMOC2	0.933	87.00%	32.26%	3.66E-280
SLC39A14	0.826	65.40%	26.37%	4.12E-278
ANXA2	0.995	96.50%	28.59%	1.72E-275

COL2A1	0.964	88.60%	62.15%	2.18E-272
ADAMTS1	0.29	11.40%	25.31%	3.70E-269
MT1H	0.38	18.00%	25.55%	2.16E-256
ARPC1B	0.731	51.70%	28.95%	8.82E-252
AMTN	0.179	4.80%	28.89%	1.50E-245
CAPZB	0.746	53.60%	28.69%	1.52E-243
INHBA	0.79	60.60%	40.43%	4.56E-227
MSMP	0.126	2.70%	39.99%	1.46E-201
CCDC80	0.928	79.90%	33.00%	1.20E-197
MYL6	0.997	97.40%	25.49%	4.31E-195
SMIM14	0.783	59.60%	25.05%	1.13E-194
CKB	0.455	27.80%	29.42%	8.83E-184
PFN1	0.935	83.40%	29.89%	2.51E-183
TIMP1	0.999	99.10%	37.10%	1.00E-174
ENPP1	0.787	62.40%	29.00%	7.98E-164
AQP3	0.436	27.90%	41.26%	1.34E-151
C2orf40	0.997	100.00%	48.22%	2.96E-133
MGST3	0.875	74.80%	26.34%	3.05E-119
MYL9	0.374	22.80%	41.20%	4.51E-113
TMSB10	0.848	71.70%	49.77%	5.47E-113
ISLR	0.803	69.30%	35.02%	5.93E-104
CALD1	0.852	71.60%	27.72%	6.40E-78
TIMP3	0.607	49.30%	46.62%	8.86E-70
LGALS1	0.946	90.50%	62.70%	1.07E-65
RGS2	0.522	40.90%	25.55%	6.18E-54
PRG4	0.403	33.60%	40.04%	3.53E-05
TAGLN	0.198	21.30%	53.37%	1
CFH1	0.884	98.40%	-160.02%	0
FRZB1	0.604	96.70%	-156.28%	0
APOD1	0.628	83.70%	-128.51%	0
CHI3L11	0.692	83.10%	-120.40%	0
JUN	0.875	95.50%	-119.77%	0
CRISPLD11	0.325	62.40%	-119.05%	0
FOSB1	0.738	92.70%	-112.31%	0
MMP31	0.412	64.40%	-111.73%	0
CHRDL21	0.062	39.70%	-109.12%	0
ZFP361	0.919	97.80%	-108.13%	0
FOS1	0.958	99.00%	-101.13%	0
SOD31	0.884	93.80%	-94.24%	0
MEG31	0.342	64.00%	-86.07%	0
JUNB1	0.988	99.50%	-85.71%	0

HAPLN11	0.92	96.80%	-85.28%	0
MIR24-21	0.536	78.20%	-83.61%	0
GPX31	0.984	99.10%	-79.28%	0
GEM1	0.706	84.40%	-79.09%	0
GDF101	0.45	75.50%	-77.67%	0
STC21	0.298	52.30%	-76.65%	0
KLF41	0.775	87.30%	-76.54%	0
IER21	0.84	87.80%	-72.43%	0
RBP41	0.606	77.10%	-70.95%	0
PPP1R15A1	0.67	78.80%	-69.95%	0
RASD11	0.505	72.30%	-69.77%	0
SOCS31	0.763	82.30%	-67.36%	0
ATF31	0.466	65.50%	-66.94%	0
BTG21	0.651	76.80%	-66.40%	0
DDIT41	0.4	59.60%	-65.22%	0
SLPI1	0.106	30.80%	-62.44%	0
COL9A31	0.838	92.90%	-62.05%	0
NR4A11	0.49	65.20%	-61.11%	0
RARRES21	0.191	47.60%	-60.05%	0
ACAN1	0.936	98.60%	-59.60%	0
CDKN1A1	0.777	86.70%	-59.50%	0
SRSF71	0.83	86.40%	-59.17%	0
TSC22D11	0.946	98.90%	-58.45%	0
CCNL11	0.827	89.10%	-57.18%	0
SLC14A11	0.24	53.60%	-56.32%	0
EGR11	0.883	93.00%	-55.94%	0
NDRG21	0.552	75.80%	-55.69%	0
BTG11	0.896	93.70%	-55.21%	0
MALAT11	1	100.00%	-54.96%	0
SERTAD11	0.763	86.30%	-54.76%	0
WWP21	0.783	85.40%	-54.16%	0
CYTL11	0.834	91.30%	-53.98%	0
DDX51	0.986	98.60%	-53.76%	0
ADM1	0.353	56.50%	-52.73%	0
GPC61	0.636	79.20%	-52.33%	0
PNRC11	0.702	79.80%	-52.17%	0
TUBB4B1	0.808	86.50%	-51.25%	0
JUND1	0.734	82.10%	-50.63%	0
SPINT21	0.839	94.00%	-50.18%	0
HSPA1B1	0.572	73.20%	-49.94%	0
UBC1	0.996	99.80%	-49.93%	0

NR4A21	0.533	66.90%	-49.57%	0
CSRNP11	0.618	75.00%	-48.89%	0
KLF101	0.502	64.00%	-48.30%	0
SRSF51	0.975	98.50%	-48.15%	0
SNHG81	0.892	94.30%	-48.13%	0
HSPA1A1	0.719	82.40%	-48.05%	0
S100B1	0.872	96.90%	-46.92%	0
PLAC91	0.967	97.10%	-44.77%	0
MYADM1	0.859	85.30%	-44.63%	0
WSB11	0.734	83.20%	-43.81%	0
HLA-C1	0.986	98.60%	-42.46%	0
SETD5-AS11	0.613	72.10%	-41.77%	0
RHOBTB31	0.442	59.80%	-41.74%	0
SERPING11	0.851	86.50%	-41.01%	0
HLA-B1	0.969	97.30%	-40.83%	0
HLA-E1	0.805	85.60%	-40.29%	0
TOB11	0.55	64.10%	-40.06%	0
METTL7A1	0.396	57.70%	-39.75%	0
EIF4A21	0.958	96.10%	-39.54%	0
EDIL31	0.732	76.60%	-39.24%	0
SRSF31	0.956	95.90%	-38.94%	0
COL11A21	0.671	78.50%	-38.82%	0
HMGB21	0.439	61.10%	-38.47%	0
CNN31	0.832	86.50%	-37.96%	0
H3F3B1	1	99.90%	-37.16%	0
VIT1	0.041	30.10%	-36.91%	0
TSPYL21	0.39	56.80%	-36.57%	0
PPP1R101	0.51	62.10%	-35.78%	0
C1S1	0.803	87.30%	-35.50%	0
SCIN1	0.416	55.80%	-34.69%	0
CIRBP1	0.99	99.10%	-34.33%	0
RBM391	0.907	91.20%	-33.00%	0
PNISR1	0.829	85.50%	-32.72%	0
AC005152.31	0.119	31.30%	-32.66%	0
RP11-983P16.41	0.465	60.20%	-32.46%	0
HLA-A1	0.991	98.60%	-31.17%	0
TRA2B1	0.823	83.40%	-31.06%	0
TCEAL21	0.708	80.20%	-30.86%	0
HNRNPA2B11	0.975	97.10%	-30.34%	0
BEX41	0.63	72.70%	-30.28%	0
HSP90AB11	0.996	99.50%	-30.03%	0

B2M1	1	99.90%	-26.72%	0
CNBP1	0.95	94.50%	-26.70%	0
GSN1	0.935	91.80%	-25.61%	0
SSR31	0.963	94.80%	-30.69%	7.84E-301
MCL11	0.837	84.80%	-33.30%	4.46E-294
PLA2G2A1	0.979	98.90%	-36.67%	1.20E-291
NAMPT1	0.348	49.40%	-35.56%	8.41E-286
NCL1	0.886	88.20%	-27.29%	3.96E-283
LAG31	0.411	53.60%	-36.34%	1.46E-281
NGFRAP11	0.904	88.00%	-26.80%	2.51E-275
CTA-29F11.11	0.292	45.70%	-30.59%	9.35E-273
BOC1	0.318	46.00%	-32.55%	3.67E-270
MAOB1	0.378	51.70%	-31.55%	2.06E-269
TIPARP1	0.502	60.80%	-39.55%	1.29E-264
ITM2A1	0.63	72.50%	-38.78%	1.68E-263
SDC21	0.958	94.70%	-29.94%	6.98E-263
EPB41L4A-AS11	0.783	80.90%	-27.61%	4.64E-259
TPD52L11	0.756	77.80%	-39.67%	4.23E-254
C2orf821	0.892	97.50%	-37.74%	1.34E-253
PLA2G161	0.294	44.30%	-28.72%	2.11E-251
EPS81	0.532	62.10%	-29.70%	6.91E-250
EIF51	0.944	93.60%	-28.69%	8.38E-240
HNRNPU1	0.651	69.30%	-27.00%	1.57E-239
VASN1	0.666	70.90%	-31.42%	2.05E-237
MIA1	0.931	97.40%	-35.76%	8.02E-236
ICAM11	0.434	55.80%	-62.17%	2.31E-235
ARL6IP11	0.527	61.20%	-29.22%	1.09E-234
CRIM11	0.324	45.50%	-28.34%	5.09E-231
ELL21	0.485	58.10%	-30.88%	1.45E-230
CD141	0.364	49.50%	-39.76%	1.15E-228
CLK11	0.68	72.20%	-29.51%	1.19E-228
KCNQ1OT11	0.538	63.20%	-41.20%	2.51E-227
NFATC11	0.495	58.10%	-34.69%	8.19E-226
PDCD41	0.446	54.60%	-26.26%	1.86E-225
C1orf631	0.598	66.10%	-29.67%	5.83E-225
FBLN71	0.194	34.20%	-26.35%	6.69E-225
RBMX1	0.792	79.70%	-25.41%	7.19E-224
VGLL41	0.632	68.20%	-29.26%	3.89E-222
COL9A11	0.142	30.00%	-28.60%	5.62E-219
MAFF1	0.201	34.30%	-27.66%	6.38E-215
XBP11	0.942	92.50%	-25.42%	2.42E-213

ILF3-AS11	0.334	46.40%	-26.80%	1.14E-212
SLC40A11	0.252	39.40%	-35.13%	9.82E-212
PIK3R11	0.297	43.20%	-37.78%	1.83E-209
PNN1	0.649	69.10%	-25.14%	4.74E-209
PPP1R3C1	0.651	70.80%	-46.16%	3.41E-207
SQSTM11	0.92	91.00%	-30.29%	1.64E-205
SAA11	0.031	13.90%	-81.34%	7.90E-205
IFRD11	0.59	65.00%	-28.59%	4.06E-202
TXNIP1	0.432	53.30%	-43.50%	1.18E-200
HES11	0.421	53.80%	-49.32%	1.19E-194
SDF41	0.472	53.60%	-26.77%	2.18E-188
TUBB2A1	0.633	69.60%	-28.51%	8.91E-187
DNAJA11	0.756	75.60%	-30.95%	7.33E-186
GTF2B1	0.426	52.10%	-29.67%	1.29E-183
KLF21	0.419	52.30%	-31.80%	1.97E-180
PCF111	0.399	49.80%	-26.88%	5.28E-178
MTRNR2L21	0.39	49.90%	-39.62%	5.84E-178
SFPQ1	0.598	63.80%	-26.02%	5.13E-174
LIMCH11	0.574	62.00%	-25.29%	3.95E-172
ID11	0.658	70.70%	-42.80%	7.04E-167
RHOB1	0.757	77.00%	-36.23%	1.51E-166
PHLDA11	0.716	73.70%	-38.61%	7.49E-161
FABP51	0.227	34.60%	-30.72%	5.37E-159
STEAP41	0.044	14.20%	-28.54%	5.07E-157
CP1	0.551	62.50%	-37.27%	7.15E-157
ID31	0.688	72.90%	-38.71%	3.90E-156
DDIT31	0.72	74.30%	-33.97%	9.94E-156
RGS31	0.793	80.10%	-32.19%	5.47E-154
HEXIM11	0.505	56.80%	-31.25%	2.76E-151
BHLHE411	0.553	61.40%	-28.40%	6.21E-151
EIF4A31	0.418	49.80%	-26.24%	1.91E-148
GMDS1	0.584	62.40%	-25.16%	1.66E-147
EIF4A11	0.963	94.40%	-25.40%	3.79E-146
SERTAD4-AS11	0.629	63.80%	-31.49%	7.02E-145
SERPINI11	0.305	40.50%	-31.84%	1.17E-144
BRD21	0.701	71.50%	-25.20%	7.11E-140
MST41	0.541	58.20%	-29.43%	3.28E-139
MAP2K61	0.317	41.20%	-26.14%	5.68E-132
CHAD1	0.822	87.70%	-28.35%	6.97E-130
TUBB2B1	0.569	65.00%	-27.98%	4.31E-127
GADD45B1	0.899	91.90%	-41.52%	1.07E-126

SNAI21	0.558	58.80%	-33.69%	4.95E-125
IRF11	0.456	52.70%	-46.77%	3.21E-117
COL9A21	0.67	68.70%	-35.80%	1.15E-116
FXYD11	0.759	71.70%	-32.36%	4.15E-107
IDI11	0.555	58.70%	-27.00%	6.20E-106
DKK11	0.121	22.00%	-30.71%	2.84E-102
GREM11	0.191	29.20%	-38.64%	5.71E-99
PRR41	0.239	32.30%	-28.92%	1.36E-97
MAFB1	0.236	32.10%	-31.10%	2.68E-89
LDLR1	0.348	41.30%	-26.18%	3.97E-89
SLC25A371	0.806	79.60%	-32.84%	2.88E-87
GLUL1	0.528	56.90%	-25.70%	8.88E-81
CST31	0.997	99.60%	-26.46%	2.08E-79
SAT11	0.965	94.50%	-25.18%	5.68E-78
BHLHE401	0.792	76.40%	-29.60%	1.88E-76
RGS161	0.729	73.60%	-53.61%	7.85E-69
NFKBIA1	0.867	87.30%	-44.38%	1.52E-64
RAMP11	0.224	28.90%	-32.11%	1.48E-57
ANKRD371	0.443	46.10%	-25.14%	6.15E-54
HMOX11	0.342	39.90%	-66.77%	2.92E-53
FGFBP21	0.951	95.50%	-37.53%	4.20E-50
EMP11	0.756	71.70%	-41.43%	1.11E-49
SCRG11	0.986	99.60%	-25.17%	2.65E-42
COL10A11	0.116	17.60%	-78.75%	5.81E-42
G0S21	0.159	21.70%	-25.61%	3.12E-41
HMGCS11	0.371	40.60%	-29.31%	1.51E-39
PDK41	0.219	27.50%	-25.45%	2.74E-39
TF1	0.516	51.80%	-55.30%	9.42E-20
RND11	0.185	21.40%	-25.79%	4.95E-13
IBSP1	0.133	16.70%	-48.68%	5.90E-10
CHI3L21	0.877	85.50%	-48.43%	6.72E-06

Supplementary Table S4. List of differentially expressed genes in osteoarthritic chondrocytes.

Gene	Putative cell type of the cluster	The percentage of cells expressing the gene in the cluster	The percentage of cells expressing the gene in the other clusters	Log fold-change of the average expression between the two groups	Adjusted p-value (Bonferroni correction)
MMP3	HomC	71.50%	45.80%	1.452	0.00E+00
CHI3L1	HomC	88.50%	71.40%	1.366	0.00E+00
CFH	HomC	99.40%	91.40%	1.249	0.00E+00
ZFP36	HomC	98.60%	93.50%	0.965	0.00E+00
GPX3	HomC	99.30%	98.60%	0.868	0.00E+00
PPP1R15A	HomC	81.90%	69.60%	0.789	0.00E+00
GEM	HomC	86.40%	74.40%	0.789	0.00E+00
ICAM1	HomC	61.30%	45.10%	0.777	0.00E+00
SERTAD1	HomC	89.70%	78.10%	0.727	0.00E+00
FOSB	HomC	90.90%	81.20%	0.684	0.00E+00
KLF4	HomC	86.70%	81.20%	0.670	0.00E+00
JUN	HomC	95.90%	90.00%	0.663	0.00E+00
SOCS3	HomC	84.70%	77.20%	0.660	0.00E+00
RPS4Y1	HomC	59.10%	21.40%	0.657	0.00E+00
TUBB4B	HomC	88.50%	81.80%	0.646	0.00E+00
DDX5	HomC	98.80%	98.50%	0.642	0.00E+00
SLC25A37	HomC	85.10%	77.60%	0.628	0.00E+00
SNHG8	HomC	96.00%	90.20%	0.618	0.00E+00
CCNL1	HomC	91.30%	83.90%	0.614	0.00E+00
TSC22D1	HomC	99.00%	96.00%	0.612	0.00E+00
BTG1	HomC	94.90%	90.40%	0.610	0.00E+00
MALAT1	HomC	100.00%	100.00%	0.610	0.00E+00
HSPA1B	HomC	74.60%	62.10%	0.602	0.00E+00
UBC	HomC	99.90%	99.60%	0.596	0.00E+00
SOD3	HomC	92.30%	91.00%	0.556	0.00E+00
MIR24-2	HomC	76.50%	63.00%	0.552	0.00E+00
CDKN1A	HomC	88.70%	79.80%	0.513	0.00E+00
CSRNP1	HomC	77.80%	65.00%	0.504	0.00E+00
JUNB	HomC	99.70%	98.90%	0.502	0.00E+00
SRSF3	HomC	96.70%	95.30%	0.500	0.00E+00
DDIT3	HomC	77.00%	71.50%	0.480	0.00E+00

NNMT	HomC	97.60%	94.90%	0.476	0.00E+00
EIF4A1	HomC	95.60%	95.10%	0.471	0.00E+00
SRSF5	HomC	98.80%	97.70%	0.458	0.00E+00
PLA2G2A	HomC	99.30%	98.10%	0.452	0.00E+00
DNAJB1	HomC	79.20%	72.70%	0.449	0.00E+00
SRSF7	HomC	86.90%	83.90%	0.448	0.00E+00
EIF5	HomC	94.20%	93.90%	0.442	0.00E+00
PNRC1	HomC	78.70%	74.10%	0.433	0.00E+00
HMGB2	HomC	63.40%	48.70%	0.426	0.00E+00
DDX3Y	HomC	38.30%	13.70%	0.420	0.00E+00
EIF4A2	HomC	96.50%	95.70%	0.420	0.00E+00
CNN3	HomC	86.90%	84.10%	0.411	0.00E+00
SQSTM1	HomC	91.00%	91.70%	0.407	0.00E+00
IFITM2	HomC	85.30%	80.90%	0.407	0.00E+00
SETD5-AS1	HomC	72.90%	64.60%	0.398	0.00E+00
CIRBP	HomC	99.30%	98.90%	0.385	0.00E+00
HLA-E	HomC	85.10%	82.40%	0.374	0.00E+00
C4BPA	HomC	24.90%	3.40%	0.371	0.00E+00
WSB1	HomC	81.90%	77.40%	0.364	0.00E+00
PLA2G16	HomC	49.00%	32.20%	0.354	0.00E+00
EIF1	HomC	100.00%	100.00%	0.353	0.00E+00
RBM39	HomC	91.00%	91.00%	0.338	0.00E+00
H3F3B	HomC	99.90%	100.00%	0.336	0.00E+00
C1S	HomC	87.50%	82.60%	0.333	0.00E+00
XBP1	HomC	92.50%	93.60%	0.322	0.00E+00
FRZB	HomC	97.80%	72.40%	0.312	0.00E+00
HNRNPA2B1	HomC	96.50%	97.60%	0.310	0.00E+00
ZFAS1	HomC	97.20%	97.40%	0.292	0.00E+00
HSP90AB1	HomC	99.50%	99.60%	0.288	0.00E+00
CNBP	HomC	94.10%	95.00%	0.272	0.00E+00
HNRNPA1	HomC	99.60%	99.60%	0.266	0.00E+00
SAA1	HomC	18.40%	4.60%	1.070	4.53E-303
HNRNPDL	HomC	90.10%	91.50%	0.285	1.05E-302
DDIT4	HomC	61.50%	45.80%	0.707	4.00E-301
NDRG2	HomC	73.60%	63.30%	0.338	6.68E-298
ADM	HomC	58.40%	41.70%	0.456	9.47E-293
HSP90AA1	HomC	98.10%	98.50%	0.305	2.51E-286
SRSF2	HomC	83.60%	84.10%	0.331	1.41E-283
ADIRF	HomC	93.50%	91.00%	0.346	9.06E-282
C1orf63	HomC	67.70%	61.10%	0.359	7.65E-281
SBDS	HomC	87.60%	88.80%	0.310	2.22E-280

HSPA1A	HomC	83.00%	75.30%	0.576	1.60E-277
CD14	HomC	53.30%	39.10%	0.467	2.89E-277
FUS	HomC	89.40%	90.60%	0.302	6.03E-273
SERPING1	HomC	84.70%	86.40%	0.385	1.76E-270
IFITM1	HomC	37.60%	21.10%	0.290	1.00E-267
CLK1	HomC	72.90%	69.10%	0.333	7.48E-263
GBP2	HomC	55.20%	41.50%	0.366	1.99E-262
MT1X	HomC	99.50%	99.20%	0.365	8.82E-262
NCL	HomC	87.60%	88.80%	0.292	9.47E-259
HMOX1	HomC	48.10%	32.20%	0.979	2.62E-258
ATF3	HomC	66.60%	52.70%	0.423	7.49E-256
RBMX	HomC	79.40%	79.60%	0.295	5.05E-255
HEXIM1	HomC	59.70%	51.20%	0.414	5.72E-255
PPP1R10	HomC	62.80%	54.50%	0.355	2.61E-253
FOS	HomC	99.00%	96.90%	0.524	1.38E-250
HMGN2	HomC	87.60%	88.70%	0.265	8.80E-248
RSL24D1	HomC	86.40%	87.70%	0.266	8.99E-248
GADD45B	HomC	93.10%	90.00%	0.591	1.70E-244
HSPD1	HomC	80.90%	81.90%	0.289	3.73E-240
RARRES2	HomC	46.90%	29.30%	0.397	5.49E-238
SSR3	HomC	94.30%	96.00%	0.280	1.71E-237
MTRNR2L2	HomC	53.70%	40.90%	0.450	7.08E-237
KLF10	HomC	64.10%	54.90%	0.397	3.65E-233
MAFB	HomC	38.90%	23.30%	0.430	1.94E-231
MGP	HomC	100.00%	100.00%	0.273	2.75E-229
GDF10	HomC	71.50%	57.50%	0.387	1.63E-227
STEAP4	HomC	18.20%	5.90%	0.360	1.38E-224
GTF2B	HomC	54.40%	44.70%	0.347	6.21E-219
SLC14A1	HomC	51.90%	35.00%	0.350	1.10E-217
MYADM	HomC	83.40%	86.60%	0.419	1.24E-216
TSPYL2	HomC	56.20%	45.50%	0.308	3.89E-214
DNAJA1	HomC	75.30%	75.70%	0.363	6.28E-213
IFRD1	HomC	65.60%	60.70%	0.321	7.12E-212
MAFF	HomC	37.40%	23.50%	0.303	3.65E-207
JUND	HomC	78.90%	78.00%	0.353	6.64E-205
PNISR	HomC	83.70%	84.60%	0.254	1.10E-202
EPB41L4A-AS1	HomC	80.00%	79.60%	0.264	4.59E-202
ARL6IP1	HomC	61.40%	55.60%	0.310	5.01E-201
IRF1	HomC	56.60%	46.20%	0.579	2.23E-194
HERPUD1	HomC	90.10%	92.40%	0.303	9.65E-193
TIPARP	HomC	61.40%	53.50%	0.376	5.16E-192

LAG3	HomC	54.40%	45.10%	0.312	6.27E-192
SLC3A2	HomC	58.70%	52.90%	0.345	2.95E-191
CHI3L2	HomC	90.10%	84.70%	0.601	6.88E-188
IER2	HomC	88.20%	85.10%	0.332	8.48E-187
TRA2B	HomC	81.80%	83.50%	0.263	1.42E-186
BRD2	HomC	71.90%	70.40%	0.310	8.91E-184
H2AFX	HomC	39.80%	28.20%	0.323	5.35E-178
PCF11	HomC	51.40%	42.50%	0.296	1.47E-177
NR4A2	HomC	65.40%	58.80%	0.364	1.61E-176
HNRNPF	HomC	66.90%	65.80%	0.267	1.23E-175
OSMR	HomC	60.90%	56.50%	0.362	1.75E-170
MAOB	HomC	51.80%	42.60%	0.298	2.21E-170
HSPA8	HomC	89.00%	91.50%	0.341	4.27E-166
SLC40A1	HomC	41.00%	29.40%	0.348	7.80E-158
MT2A	HomC	99.80%	99.70%	0.315	3.38E-156
RGS3	HomC	79.90%	79.70%	0.342	1.03E-153
RSRC2	HomC	74.80%	76.50%	0.255	3.96E-153
MT1E	HomC	99.00%	98.90%	0.335	4.08E-151
BAG3	HomC	58.40%	54.20%	0.295	4.94E-149
PIK3R1	HomC	43.70%	34.10%	0.413	4.82E-148
NAMPT	HomC	48.40%	40.30%	0.319	3.27E-144
SFPQ	HomC	62.80%	61.70%	0.268	5.96E-143
CP	HomC	63.00%	57.40%	0.421	1.53E-139
ERRFI1	HomC	78.20%	76.50%	0.509	2.13E-137
MAP2K6	HomC	43.80%	33.70%	0.259	4.64E-136
PLSCR1	HomC	42.00%	33.60%	0.287	5.73E-135
LBH	HomC	51.60%	45.30%	0.365	1.66E-133
EGR1	HomC	92.30%	90.30%	0.261	2.26E-133
TXNIP	HomC	52.80%	46.90%	0.428	2.15E-131
RGS16	HomC	75.70%	72.20%	0.729	5.27E-131
TUBB2A	HomC	68.60%	66.00%	0.285	1.11E-130
ELL2	HomC	56.60%	52.50%	0.283	3.22E-130
COL12A1	HomC	42.90%	32.80%	0.280	3.85E-128
MIR22HG	HomC	54.00%	50.70%	0.306	1.26E-126
PHLDA1	HomC	73.30%	72.50%	0.370	1.51E-126
RASD1	HomC	69.50%	59.40%	0.298	1.34E-121
JMJD6	HomC	52.50%	48.00%	0.262	1.26E-119
NFKBIA	HomC	87.90%	86.60%	0.607	7.46E-119
AMD1	HomC	59.20%	58.30%	0.267	1.56E-112
BTG2	HomC	74.80%	70.10%	0.280	3.81E-112
SERPINB1	HomC	54.80%	52.30%	0.293	5.63E-112

TOB1	HomC	61.60%	59.30%	0.268	8.94E-111
MTRNR2L8	HomC	33.70%	24.70%	0.251	2.99E-108
ARID5A	HomC	49.10%	43.60%	0.286	7.51E-108
EIF4A3	HomC	49.40%	44.70%	0.250	4.23E-106
METTL7A	HomC	54.40%	47.40%	0.251	3.41E-103
MTRNR2L12	HomC	39.70%	32.50%	0.255	1.75E-99
HNRNPH1	HomC	63.50%	63.90%	0.254	6.42E-94
TUBB2B	HomC	64.60%	59.90%	0.286	7.21E-94
NR4A1	HomC	62.70%	55.80%	0.294	4.28E-93
STC2	HomC	49.10%	39.20%	0.295	4.61E-93
MEG3	HomC	56.80%	48.00%	0.318	6.60E-90
APOD	HomC	79.10%	72.30%	0.327	1.94E-89
ID3	HomC	73.30%	70.00%	0.310	5.15E-89
SOD2	HomC	83.60%	84.80%	0.522	7.33E-86
BAZ1A	HomC	41.00%	35.70%	0.256	1.97E-84
SNAPC1	HomC	55.50%	54.60%	0.259	2.46E-80
MYC	HomC	35.40%	28.20%	0.440	6.18E-77
KIAA0040	HomC	62.60%	64.60%	0.296	1.98E-76
WTAP	HomC	62.00%	64.30%	0.313	4.24E-76
GLUL	HomC	56.30%	54.50%	0.312	2.93E-75
TF	HomC	55.70%	49.80%	0.290	1.33E-70
ID1	HomC	69.90%	67.90%	0.320	5.64E-70
KCNQ1OT1	HomC	59.70%	58.70%	0.325	7.98E-70
SAT1	HomC	93.90%	96.10%	0.277	1.11E-69
RND1	HomC	25.10%	17.70%	0.407	3.74E-66
MT1M	HomC	80.80%	81.50%	0.397	5.37E-62
PDK4	HomC	28.80%	23.20%	0.325	3.28E-41
BHLHE40	HomC	73.60%	79.60%	0.276	2.31E-28
EMP1	HomC	68.20%	75.90%	0.496	6.24E-27
C10orf10	HomC	26.90%	23.50%	0.255	2.39E-23
MT1A	HomC	40.30%	40.70%	0.334	1.45E-20
CCL2	HomC	23.50%	19.60%	0.529	2.06E-19
HILPDA	HomC	33.80%	32.90%	0.289	1.69E-15
G0S2	HomC	21.00%	18.20%	0.336	2.28E-12
HSPA6	HomC	10.40%	7.70%	0.261	1.33E-10
IER3	HomC	84.40%	86.90%	0.346	4.36E-01
COL10A1	HTC	39.80%	12.20%	1.404	0.00E+00
CHRDL2	HTC	78.00%	19.00%	1.399	0.00E+00
FRZB	HTC	99.90%	78.50%	1.369	0.00E+00
CRISPLD1	HTC	96.50%	43.90%	1.359	0.00E+00
IBSP	HTC	29.50%	13.60%	1.186	9.70E-114

TF	HTC	73.80%	49.30%	1.088	4.40E-228
FGFBP2	HTC	100.00%	94.80%	1.025	0.00E+00
IER2	HTC	97.70%	84.90%	1.001	0.00E+00
PDPN	HTC	95.00%	73.50%	0.952	0.00E+00
HAPLN1	HTC	100.00%	94.10%	0.910	0.00E+00
RBP4	HTC	94.40%	67.10%	0.894	0.00E+00
STC2	HTC	76.80%	38.60%	0.871	0.00E+00
RASD1	HTC	88.80%	59.80%	0.870	0.00E+00
JUN	HTC	98.80%	91.20%	0.866	0.00E+00
CYTL1	HTC	99.60%	86.50%	0.861	0.00E+00
C2orf82	HTC	100.00%	93.10%	0.861	0.00E+00
SLPI	HTC	50.60%	18.60%	0.852	0.00E+00
JUNB	HTC	100.00%	99.10%	0.845	0.00E+00
APOD	HTC	93.90%	72.40%	0.817	0.00E+00
SCRG1	HTC	100.00%	99.10%	0.805	0.00E+00
FOS	HTC	100.00%	97.40%	0.796	0.00E+00
NR4A1	HTC	85.50%	55.00%	0.749	0.00E+00
RAMP1	HTC	61.60%	22.10%	0.744	0.00E+00
SPINT2	HTC	99.90%	88.40%	0.743	0.00E+00
ATF3	HTC	83.00%	54.30%	0.743	0.00E+00
CST3	HTC	99.90%	99.60%	0.741	0.00E+00
BTG2	HTC	89.70%	69.60%	0.737	0.00E+00
DKK1	HTC	49.50%	14.10%	0.734	0.00E+00
PLAC9	HTC	99.80%	96.60%	0.718	0.00E+00
CHAD	HTC	99.80%	83.60%	0.717	0.00E+00
FXYD1	HTC	95.00%	71.20%	0.708	0.00E+00
SERPINA5	HTC	98.00%	82.10%	0.699	0.00E+00
EGR1	HTC	98.70%	90.10%	0.689	0.00E+00
MIA	HTC	100.00%	95.00%	0.688	0.00E+00
FOSB	HTC	98.00%	82.80%	0.668	0.00E+00
TSPAN13	HTC	65.10%	30.00%	0.655	0.00E+00
SERPINA1	HTC	100.00%	95.70%	0.652	0.00E+00
MEG3	HTC	82.20%	47.40%	0.643	0.00E+00
ACAN	HTC	100.00%	96.00%	0.637	0.00E+00
S100B	HTC	99.90%	91.80%	0.634	0.00E+00
LECT1	HTC	73.00%	44.20%	0.628	1.82E-242
WWP2	HTC	97.50%	80.60%	0.620	0.00E+00
PPP1R3C	HTC	88.20%	66.10%	0.593	5.22E-276
ACTG1	HTC	100.00%	99.30%	0.578	0.00E+00
IGFBP6	HTC	82.70%	67.80%	0.557	8.28E-129
SLC14A1	HTC	77.90%	36.40%	0.552	0.00E+00

HES1	HTC	70.60%	46.20%	0.539	1.31E-185
SRSF7	HTC	94.70%	83.80%	0.539	1.65E-279
ITM2A	HTC	93.80%	65.50%	0.537	0.00E+00
GDF10	HTC	89.60%	59.00%	0.533	2.22E-292
SERPINI1	HTC	65.60%	32.90%	0.532	0.00E+00
S100A1	HTC	100.00%	87.70%	0.523	0.00E+00
SPTSSB	HTC	46.90%	15.30%	0.517	0.00E+00
HMGCS1	HTC	56.80%	37.10%	0.516	1.19E-120
TTLL7	HTC	73.50%	33.20%	0.514	0.00E+00
TPD52L1	HTC	89.60%	75.40%	0.511	9.19E-227
NDRG2	HTC	91.80%	63.90%	0.511	0.00E+00
DUSP1	HTC	91.80%	74.60%	0.510	2.72E-228
MDFI	HTC	93.10%	66.70%	0.507	0.00E+00
ACTB	HTC	100.00%	99.60%	0.505	0.00E+00
PHOSPHO1	HTC	57.30%	16.20%	0.503	0.00E+00
COL9A2	HTC	95.00%	64.90%	0.489	0.00E+00
FCGRT	HTC	94.10%	76.40%	0.481	0.00E+00
MFGE8	HTC	98.10%	93.30%	0.473	2.35E-207
IDI1	HTC	78.60%	54.90%	0.466	2.58E-222
EDIL3	HTC	91.00%	73.30%	0.457	1.40E-271
MRPS6	HTC	89.80%	65.40%	0.457	0.00E+00
LDLR	HTC	64.60%	35.50%	0.455	7.54E-255
KCNMA1	HTC	82.80%	62.60%	0.451	1.49E-176
COL9A3	HTC	99.80%	87.60%	0.450	9.12E-281
TPM4	HTC	80.80%	61.40%	0.448	7.26E-207
CRYAB	HTC	92.10%	80.10%	0.446	4.65E-130
SCIN	HTC	79.60%	46.20%	0.446	0.00E+00
MST4	HTC	84.40%	53.30%	0.446	0.00E+00
AC005152.3	HTC	52.70%	19.40%	0.445	0.00E+00
ITM2C	HTC	99.80%	92.60%	0.443	0.00E+00
RHOB	HTC	89.20%	75.00%	0.442	1.37E-149
BHLHE41	HTC	83.20%	56.00%	0.436	9.22E-262
CLEC3A	HTC	98.20%	70.30%	0.431	1.83E-281
SNAI2	HTC	75.70%	55.40%	0.427	1.02E-170
INSIG1	HTC	67.20%	46.50%	0.424	2.58E-130
MIR24-2	HTC	87.30%	65.20%	0.421	2.19E-190
HSPB1	HTC	97.50%	93.90%	0.418	5.43E-111
BHLHE40	HTC	88.30%	76.50%	0.410	3.97E-141
NR4A2	HTC	79.80%	58.80%	0.407	7.09E-165
FOXA3	HTC	68.70%	38.50%	0.405	3.10E-249
VIT	HTC	47.60%	15.40%	0.405	0.00E+00

WIF1	HTC	45.30%	21.00%	0.402	1.16E-177
CPE	HTC	56.60%	32.20%	0.401	3.48E-176
MEF2C	HTC	67.10%	39.70%	0.400	4.35E-221
NGFRAP1	HTC	97.90%	88.10%	0.398	0.00E+00
SDC2	HTC	99.30%	94.70%	0.397	1.54E-246
PLD3	HTC	97.20%	87.30%	0.393	0.00E+00
GREM1	HTC	44.20%	22.60%	0.392	4.82E-132
SERTAD4	HTC	65.10%	41.20%	0.390	8.30E-177
MCL1	HTC	94.60%	83.20%	0.388	1.42E-218
CD99	HTC	99.70%	96.70%	0.386	3.53E-294
KLF10	HTC	76.50%	55.80%	0.386	4.43E-161
METTL7A	HTC	76.40%	46.70%	0.385	1.50E-236
ADM	HTC	68.10%	44.80%	0.381	4.31E-134
TMEM59	HTC	99.80%	98.00%	0.381	0.00E+00
AP3S1	HTC	96.20%	84.50%	0.381	3.00E-250
AGR2	HTC	18.40%	4.10%	0.380	5.89E-200
ZFP36	HTC	99.60%	94.70%	0.377	7.09E-202
TOB1	HTC	78.80%	58.00%	0.377	1.48E-185
TUBB2B	HTC	81.70%	59.20%	0.371	2.15E-151
TIMP4	HTC	80.40%	62.30%	0.368	6.89E-155
GSN	HTC	98.90%	91.90%	0.366	1.18E-301
MSMO1	HTC	56.20%	34.80%	0.365	1.61E-133
NUCB2	HTC	98.90%	89.00%	0.365	1.15E-229
FBXO2	HTC	98.40%	85.00%	0.363	9.01E-265
CHID1	HTC	86.80%	65.80%	0.356	9.30E-248
SSR4	HTC	99.60%	96.40%	0.356	4.15E-231
COL11A2	HTC	96.00%	70.90%	0.354	1.67E-250
HLA-A	HTC	99.80%	98.70%	0.352	7.07E-257
SOD3	HTC	94.20%	91.10%	0.352	4.06E-72
PRDX4	HTC	98.30%	90.60%	0.351	3.69E-206
SLC29A1	HTC	88.50%	77.50%	0.346	7.14E-103
NEU1	HTC	61.30%	44.00%	0.340	2.70E-102
PDE4DIP	HTC	84.00%	64.50%	0.340	4.26E-206
CD59	HTC	99.00%	93.20%	0.337	2.00E-222
CYR61	HTC	96.00%	93.00%	0.336	7.78E-58
ADRB2	HTC	60.30%	37.50%	0.336	3.12E-146
GPC6	HTC	89.20%	70.40%	0.333	2.75E-200
WISP3	HTC	81.90%	61.50%	0.332	5.35E-164
CDKN1A	HTC	90.90%	81.80%	0.332	1.30E-129
SPOCK3	HTC	21.90%	3.10%	0.330	0.00E+00
NFATC1	HTC	72.80%	52.20%	0.328	3.16E-145

PEBP1	HTC	99.20%	95.50%	0.328	9.14E-277
EGR3	HTC	42.70%	22.70%	0.328	2.59E-131
CD83	HTC	33.60%	13.70%	0.325	8.44E-165
CYSTM1	HTC	78.10%	62.10%	0.324	3.59E-132
CD9	HTC	99.30%	94.50%	0.323	3.03E-228
HLA-C	HTC	99.90%	98.50%	0.322	2.37E-249
CTH	HTC	41.80%	16.20%	0.321	2.35E-240
LDLRAD4	HTC	76.90%	58.30%	0.319	1.65E-126
H3F3B	HTC	100.00%	100.00%	0.319	1.21E-170
ANKRD37	HTC	59.10%	43.80%	0.319	2.33E-79
TSPAN3	HTC	82.20%	61.20%	0.319	6.74E-194
EXTL1	HTC	49.70%	19.40%	0.315	3.68E-293
HEXB	HTC	82.00%	66.80%	0.315	1.24E-146
CFH	HTC	99.20%	93.40%	0.314	1.88E-156
FGFR1	HTC	82.60%	68.40%	0.313	2.12E-97
JUND	HTC	90.70%	76.90%	0.307	2.32E-142
SOX9	HTC	86.90%	69.00%	0.307	3.91E-125
GEM	HTC	90.50%	76.90%	0.304	2.50E-125
LGI4	HTC	46.30%	18.30%	0.304	1.60E-248
NR1D1	HTC	81.70%	65.40%	0.303	8.20E-105
FXYD6	HTC	97.30%	85.30%	0.303	4.15E-148
DLX5	HTC	64.20%	45.90%	0.302	8.42E-97
TSPAN6	HTC	90.00%	68.90%	0.299	2.26E-177
ZNF385B	HTC	51.80%	19.90%	0.296	0.00E+00
CTA-29F11.1	HTC	60.90%	35.90%	0.295	5.27E-157
SLC44A2	HTC	65.80%	41.80%	0.295	5.55E-167
TUBA1A	HTC	65.90%	59.90%	0.293	5.00E-23
DDIT4	HTC	68.80%	48.90%	0.292	1.33E-99
GLIPR1	HTC	81.50%	61.40%	0.287	1.53E-128
CYB5D2	HTC	69.80%	43.70%	0.286	5.24E-195
AQPEP	HTC	43.00%	16.00%	0.284	2.80E-273
RCN3	HTC	94.00%	84.80%	0.282	3.49E-134
HSPB6	HTC	60.00%	39.10%	0.281	3.65E-127
MFI2	HTC	74.60%	53.00%	0.276	7.20E-139
SERTAD4-AS1	HTC	77.30%	61.90%	0.276	5.92E-93
TMED10	HTC	98.20%	89.80%	0.275	4.61E-171
VGLL4	HTC	80.60%	64.40%	0.274	1.49E-119
CNN2	HTC	54.30%	28.60%	0.274	1.79E-181
TSPYL2	HTC	69.90%	46.70%	0.272	3.44E-148
RHOBTB3	HTC	69.80%	51.00%	0.268	2.93E-107
DAP	HTC	83.50%	66.40%	0.267	1.19E-139

ARL4D	HTC	42.60%	26.00%	0.267	1.99E-83
PHLDA1	HTC	80.10%	71.90%	0.267	1.86E-47
BOC	HTC	58.70%	37.60%	0.266	2.31E-127
RARRES2	HTC	50.10%	33.40%	0.266	2.15E-66
RP11-983P16.4	HTC	74.20%	52.00%	0.266	4.96E-145
CSRP1	HTC	79.50%	64.00%	0.265	1.31E-120
TMED4	HTC	83.70%	67.20%	0.261	3.25E-142
GABARAPL1	HTC	72.20%	57.40%	0.258	1.36E-90
B2M	HTC	100.00%	99.90%	0.257	1.71E-239
LRPAP1	HTC	89.90%	79.10%	0.257	6.16E-100
STK38L	HTC	79.90%	64.90%	0.256	3.09E-79
ATRAID	HTC	95.90%	89.10%	0.256	5.03E-129
PRPSAP1	HTC	63.30%	38.50%	0.256	3.49E-163
RPN2	HTC	91.30%	80.20%	0.256	1.14E-103
PDLIM5	HTC	80.60%	63.60%	0.254	1.03E-128
SCPEP1	HTC	71.70%	54.40%	0.253	8.65E-117
CD320	HTC	70.70%	51.80%	0.253	2.62E-122
A2M	HTC	58.60%	37.20%	0.251	2.64E-108
WSB1	HTC	91.90%	77.40%	0.250	8.65E-149
CLEC3A	PreHTC	98.90%	70.30%	1.001	0.00E+00
C2orf82	PreHTC	100.00%	93.10%	0.993	0.00E+00
CHRDL2	PreHTC	65.70%	20.60%	0.969	0.00E+00
FGFBP2	PreHTC	100.00%	94.80%	0.955	0.00E+00
CRISPLD1	PreHTC	90.20%	44.80%	0.918	0.00E+00
SCRG1	PreHTC	100.00%	99.10%	0.860	0.00E+00
CYTL1	PreHTC	99.40%	86.60%	0.833	0.00E+00
PDPN	PreHTC	94.80%	73.60%	0.753	0.00E+00
FRZB	PreHTC	99.80%	78.60%	0.718	0.00E+00
FXYD1	PreHTC	94.50%	71.30%	0.702	0.00E+00
PLAC9	PreHTC	99.80%	96.60%	0.692	0.00E+00
ITM2A	PreHTC	95.10%	65.50%	0.682	0.00E+00
HAPLN1	PreHTC	100.00%	94.10%	0.672	0.00E+00
S100B	PreHTC	100.00%	91.80%	0.662	0.00E+00
SPINT2	PreHTC	99.80%	88.40%	0.647	0.00E+00
CHAD	PreHTC	99.90%	83.70%	0.647	0.00E+00
SERPINA5	PreHTC	98.50%	82.10%	0.643	0.00E+00
ACAN	PreHTC	100.00%	96.00%	0.641	0.00E+00
S100A1	PreHTC	100.00%	87.70%	0.611	0.00E+00
WWP2	PreHTC	96.50%	80.70%	0.589	0.00E+00
MDFI	PreHTC	94.80%	66.60%	0.589	0.00E+00
COL9A2	PreHTC	95.00%	65.10%	0.586	0.00E+00

SERPINA1	PreHTC	100.00%	95.80%	0.557	0.00E+00
MIA	PreHTC	99.90%	95.00%	0.549	0.00E+00
DKK1	PreHTC	45.60%	14.60%	0.534	0.00E+00
PHOSPHO1	PreHTC	57.00%	16.40%	0.526	0.00E+00
MRPS6	PreHTC	90.50%	65.40%	0.517	0.00E+00
COL11A2	PreHTC	96.40%	71.00%	0.501	0.00E+00
WIF1	PreHTC	54.90%	20.10%	0.491	0.00E+00
MST4	PreHTC	86.60%	53.20%	0.489	0.00E+00
FBXO2	PreHTC	98.30%	85.00%	0.447	0
ITM2C	PreHTC	99.60%	92.60%	0.445	0
NGFRAP1	PreHTC	98.50%	88.10%	0.429	0
SSR4	PreHTC	99.70%	96.40%	0.425	0
SERF2	PreHTC	100.00%	99.80%	0.330	0
FTL	PreHTC	100.00%	100.00%	0.274	0
EXTL1	PreHTC	51.10%	19.30%	0.290	1.0517E-297
NUCB2	PreHTC	99.10%	89.00%	0.412	4.8112E-291
TSPAN13	PreHTC	61.90%	30.50%	0.548	5.1207E-289
APOD	PreHTC	94.80%	72.30%	0.625	6.688E-287
TMEM59	PreHTC	99.80%	98.00%	0.355	1.0054E-285
FXYD6	PreHTC	97.20%	85.40%	0.435	1.7538E-275
TTLL7	PreHTC	67.20%	34.00%	0.357	7.7183E-275
GREM1	PreHTC	51.20%	21.90%	0.791	4.5545E-271
TSPAN6	PreHTC	91.90%	68.70%	0.397	5.194E-271
RAMP1	PreHTC	53.80%	23.10%	0.528	1.7406E-268
SPTSSB	PreHTC	43.70%	15.80%	0.366	7.7836E-265
CPE	PreHTC	62.90%	31.70%	0.443	1.9611E-261
BHLHE41	PreHTC	83.80%	56.00%	0.454	5.7425E-261
COL9A1	PreHTC	49.20%	20.20%	0.497	1.5503E-258
PRDX4	PreHTC	98.80%	90.60%	0.406	1.2044E-257
DUSP1	PreHTC	92.70%	74.60%	0.538	6.918E-254
EDIL3	PreHTC	90.40%	73.50%	0.431	7.9572E-250
FCGRT	PreHTC	93.10%	76.60%	0.369	2.9226E-246
TMED10	PreHTC	98.80%	89.80%	0.337	1.3377E-244
FGFR1	PreHTC	88.90%	67.80%	0.461	1.9056E-240
TSPO	PreHTC	99.50%	94.30%	0.355	1.8256E-228
PEBP1	PreHTC	99.30%	95.50%	0.301	5.4806E-223
PCOLCE2	PreHTC	100.00%	96.70%	0.339	7.2149E-222
C2orf40	PreHTC	100.00%	99.80%	0.522	1.9157E-220
MFI2	PreHTC	79.30%	52.50%	0.354	1.6613E-216
STC2	PreHTC	70.70%	39.40%	0.459	2.8687E-215
COL9A3	PreHTC	99.60%	87.70%	0.431	3.78E-208

SPARC	PreHTC	100.00%	97.00%	0.354	4.79E-208
LECT1	PreHTC	73.00%	44.30%	0.598	5.06E-207
RBP4	PreHTC	88.00%	67.80%	0.602	8.54E-201
LDLRAD4	PreHTC	80.40%	58.00%	0.395	2.16E-198
RASD1	PreHTC	84.20%	60.40%	0.518	2.80E-194
COL10A1	PreHTC	34.30%	12.90%	0.983	9.19E-193
HLA-A	PreHTC	99.90%	98.70%	0.307	8.57E-191
GDF10	PreHTC	86.30%	59.40%	0.354	1.20E-186
DAP	PreHTC	86.50%	66.20%	0.302	1.05E-184
MFGE8	PreHTC	98.50%	93.20%	0.501	2.00E-181
FOS	PreHTC	99.80%	97.40%	0.329	1.39E-180
JUNB	PreHTC	99.60%	99.10%	0.329	4.90E-177
EGR1	PreHTC	97.20%	90.30%	0.402	9.54E-176
CILP	PreHTC	98.40%	89.10%	0.507	2.40E-175
CD99	PreHTC	99.60%	96.70%	0.282	4.53E-175
SLC44A2	PreHTC	67.50%	41.80%	0.289	2.82E-174
SERTAD4	PreHTC	66.00%	41.20%	0.346	2.77E-169
STK38L	PreHTC	85.50%	64.30%	0.356	2.45E-168
S100A13	PreHTC	99.80%	97.20%	0.260	5.64E-168
AP3S1	PreHTC	96.40%	84.50%	0.323	1.69E-166
SERPINI1	PreHTC	59.10%	33.70%	0.350	2.94E-166
TSPAN3	PreHTC	81.50%	61.40%	0.291	3.96E-165
GPC6	PreHTC	88.10%	70.60%	0.319	3.62E-164
CNPY2	PreHTC	93.40%	80.00%	0.301	1.04E-161
CHID1	PreHTC	84.00%	66.20%	0.294	7.74E-160
SLC14A1	PreHTC	67.20%	37.70%	0.278	5.39E-158
FOXA3	PreHTC	65.20%	39.00%	0.292	5.84E-158
GSN	PreHTC	98.50%	91.90%	0.251	6.14E-158
COL11A1	PreHTC	95.60%	77.10%	0.284	4.20E-157
CD59	PreHTC	99.00%	93.20%	0.296	2.52E-155
PLD3	PreHTC	97.30%	87.30%	0.271	6.95E-155
NR4A1	PreHTC	78.80%	55.80%	0.393	6.69E-154
NTAN1	PreHTC	60.40%	36.80%	0.293	6.82E-151
RNF130	PreHTC	79.80%	59.10%	0.276	5.75E-145
KRT10	PreHTC	94.10%	81.20%	0.293	1.26E-144
NDRG2	PreHTC	87.30%	64.50%	0.261	2.02E-144
SOX9	PreHTC	87.10%	69.00%	0.322	1.75E-143
HSPB6	PreHTC	62.80%	38.90%	0.264	1.38E-142
XIST	PreHTC	77.80%	58.50%	0.354	3.25E-140
CTA-29F11.1	PreHTC	61.30%	36.00%	0.264	4.59E-140
IER2	PreHTC	93.60%	85.30%	0.408	1.23E-138

TIMP4	PreHTC	80.50%	62.40%	0.343	7.59E-136
NDUFA1	PreHTC	98.00%	92.70%	0.267	9.96E-133
GLIPR1	PreHTC	81.70%	61.50%	0.306	9.44E-130
RHOB	PreHTC	88.80%	75.10%	0.351	1.56E-128
PDE4DIP	PreHTC	82.50%	64.70%	0.256	3.95E-128
IGFBP7	PreHTC	74.20%	56.10%	0.495	3.06E-126
SLC26A2	PreHTC	60.50%	40.20%	0.314	5.52E-123
ATRAID	PreHTC	96.50%	89.10%	0.259	1.98E-122
RHOBTB3	PreHTC	71.70%	50.90%	0.263	4.36E-122
LDLR	PreHTC	58.40%	36.30%	0.270	2.61E-121
PPP1R3C	PreHTC	85.10%	66.50%	0.291	4.56E-117
FOSB	PreHTC	96.20%	83.10%	0.277	8.95E-115
EGR3	PreHTC	41.20%	22.90%	0.359	3.00E-110
MEF2C	PreHTC	61.00%	40.50%	0.286	4.46E-109
CALM1	PreHTC	97.90%	93.70%	0.274	4.97E-109
RPN2	PreHTC	91.90%	80.20%	0.251	1.14E-108
WISP3	PreHTC	80.50%	61.70%	0.256	4.41E-107
SERTAD4-AS1	PreHTC	79.40%	61.70%	0.258	4.96E-103
NR1D1	PreHTC	83.30%	65.20%	0.253	1.22E-102
CRYAB	PreHTC	91.90%	80.20%	0.375	7.95E-102
RCN3	PreHTC	94.20%	84.80%	0.251	3.82E-98
SLC29A1	PreHTC	89.70%	77.40%	0.312	2.02E-96
INSIG1	PreHTC	65.50%	46.80%	0.340	2.17E-96
SNAI2	PreHTC	73.40%	55.80%	0.290	7.69E-95
CYSTM1	PreHTC	77.20%	62.30%	0.269	3.15E-94
COL2A1	PreHTC	98.20%	91.40%	0.279	5.03E-94
AGR2	PreHTC	14.40%	4.60%	0.365	8.39E-90
BTG2	PreHTC	82.20%	70.50%	0.373	1.35E-89
NRN1	PreHTC	94.20%	84.30%	0.259	2.15E-87
MEG3	PreHTC	66.40%	49.20%	0.353	3.17E-80
HMGCS1	PreHTC	54.00%	37.40%	0.341	6.88E-74
TF	PreHTC	66.70%	50.10%	0.439	1.14E-64
IBSP	PreHTC	26.00%	14.00%	0.967	3.36E-64
SLPI	PreHTC	35.10%	20.40%	0.317	1.48E-59
KCNMA1	PreHTC	76.90%	63.30%	0.269	6.26E-58
CST3	PreHTC	100.00%	99.60%	0.260	3.58E-56
SPP1	PreHTC	37.70%	35.10%	0.716	2.70E-06
COL2A1	RepC	99.80%	90.60%	1.345	0.00E+00
CLEC3A	RepC	98.60%	68.30%	1.167	0.00E+00
SPARC	RepC	99.90%	96.80%	0.902	0.00E+00
C2orf40	RepC	100.00%	99.80%	0.860	0.00E+00

CTHRC1	RepC	81.60%	48.90%	0.737	0.00E+00
CILP	RepC	99.20%	88.30%	0.683	0.00E+00
OGN	RepC	94.80%	77.70%	0.636	0.00E+00
CILP2	RepC	94.00%	71.60%	0.634	0.00E+00
TNFRSF11B	RepC	95.80%	75.40%	0.625	0.00E+00
OMD	RepC	87.10%	60.80%	0.622	0.00E+00
COL11A1	RepC	95.10%	75.90%	0.579	0.00E+00
COL3A1	RepC	96.10%	73.40%	0.572	0.00E+00
MATN3	RepC	80.10%	49.20%	0.543	0.00E+00
COMP	RepC	100.00%	99.90%	0.536	0.00E+00
COL9A2	RepC	92.60%	63.30%	0.465	0.00E+00
SMOC2	RepC	99.20%	88.00%	0.444	0.00E+00
C2orf82	RepC	99.90%	92.70%	0.432	0.00E+00
FMOD	RepC	100.00%	99.00%	0.414	0.00E+00
SERPINE2	RepC	90.70%	55.00%	0.413	0.00E+00
CHAD	RepC	99.10%	82.70%	0.409	0.00E+00
COL5A2	RepC	86.50%	60.50%	0.408	0.00E+00
PCOLCE2	RepC	100.00%	96.50%	0.401	0.00E+00
S100A1	RepC	99.50%	86.90%	0.379	0.00E+00
SCRG1	RepC	100.00%	99.00%	0.376	0.00E+00
SBSPON	RepC	71.90%	39.80%	0.354	0.00E+00
SRPX2	RepC	83.10%	51.30%	0.342	0.00E+00
DNER	RepC	44.70%	16.10%	0.322	0.00E+00
PRELP	RepC	100.00%	99.60%	0.441	1.82E-303
CA9	RepC	65.30%	34.40%	0.305	2.36E-303
FXYD6	RepC	97.80%	84.40%	0.320	1.69E-298
TSPAN2	RepC	92.20%	65.90%	0.333	2.44E-297
UROC1	RepC	43.80%	18.20%	0.325	9.83E-290
PID1	RepC	86.30%	60.10%	0.319	1.32E-289
IGF2	RepC	53.00%	25.10%	0.436	2.70E-285
ANGPTL2	RepC	99.20%	87.60%	0.337	2.55E-281
SERPINE1	RepC	65.70%	36.60%	0.468	6.93E-281
P4HA2	RepC	86.00%	60.70%	0.315	4.08E-271
NTRK2	RepC	64.80%	35.80%	0.291	5.25E-271
SERPINA5	RepC	97.50%	81.10%	0.311	5.59E-267
FIBIN	RepC	95.90%	81.00%	0.420	1.42E-264
LOX	RepC	61.70%	33.10%	0.294	6.19E-263
EPS8L2	RepC	77.30%	50.80%	0.288	1.57E-240
COL11A2	RepC	92.80%	69.80%	0.329	1.10E-238
LECT1	RepC	67.90%	43.10%	0.539	1.08E-231
S100A13	RepC	99.90%	97.00%	0.255	1.24E-230

STK38L	RepC	86.80%	62.50%	0.305	4.75E-226
FAM162A	RepC	96.20%	84.60%	0.279	9.15E-223
AQP3	RepC	55.70%	31.00%	0.501	3.22E-220
TNNT3	RepC	53.60%	29.20%	0.499	1.68E-218
PPIC	RepC	89.70%	69.60%	0.316	1.91E-218
ITM2A	RepC	88.40%	64.60%	0.304	1.79E-211
NRN1	RepC	94.50%	83.60%	0.334	5.64E-208
LDLRAD4	RepC	80.20%	56.50%	0.251	7.96E-191
CYTL1	RepC	96.20%	86.30%	0.414	8.47E-185
PAPSS2	RepC	92.00%	74.10%	0.251	2.69E-182
COL9A3	RepC	99.40%	86.90%	0.260	2.31E-149
ID2	RepC	97.30%	90.20%	0.275	9.38E-130
CKB	RepC	52.30%	32.50%	0.251	2.52E-119
GREM1	RepC	38.80%	22.10%	0.298	1.03E-108
RCAN1	RepC	80.00%	64.30%	0.305	6.45E-79
SPP1	RepC	47.50%	33.10%	0.328	9.60E-76
TNFRSF11B	PreFC	98.70%	75.90%	1.363	0.00E+00
IL11	PreFC	28.40%	3.60%	1.296	0.00E+00
OGN	PreFC	99.20%	77.90%	1.181	0.00E+00
COL3A1	PreFC	99.60%	74.00%	1.169	0.00E+00
SERPINE2	PreFC	97.60%	55.70%	1.119	0.00E+00
TNFAIP6	PreFC	56.20%	15.20%	1.043	0.00E+00
ABI3BP	PreFC	94.50%	41.50%	1.023	0.00E+00
CD55	PreFC	94.70%	67.70%	0.985	0.00E+00
ASPN	PreFC	89.00%	56.00%	0.954	0.00E+00
HTRA1	PreFC	99.80%	78.50%	0.950	0.00E+00
FN1	PreFC	100.00%	99.90%	0.842	0.00E+00
S100A4	PreFC	97.60%	56.20%	0.836	0.00E+00
TSPAN2	PreFC	98.50%	66.30%	0.833	0.00E+00
COMP	PreFC	100.00%	99.90%	0.819	0.00E+00
NGF	PreFC	76.50%	16.50%	0.812	0.00E+00
GAS1	PreFC	90.80%	34.90%	0.805	0.00E+00
SMOC1	PreFC	95.00%	40.60%	0.769	0.00E+00
PTGES	PreFC	84.10%	30.40%	0.768	0.00E+00
COL5A1	PreFC	95.10%	46.10%	0.751	0.00E+00
CILP2	PreFC	96.30%	72.30%	0.731	0.00E+00
TGFBI	PreFC	92.00%	31.40%	0.707	0.00E+00
SRPX2	PreFC	95.50%	51.10%	0.704	0.00E+00
S100A6	PreFC	100.00%	99.90%	0.685	0.00E+00
COL1A2	PreFC	93.10%	45.80%	0.647	0.00E+00
COL2A1	PreFC	99.60%	91.00%	0.634	0.00E+00

TNC	PreFC	93.20%	44.60%	0.626	0.00E+00
INHBA	PreFC	92.90%	65.50%	0.619	0.00E+00
COL15A1	PreFC	86.20%	29.10%	0.611	0.00E+00
S100A2	PreFC	28.00%	6.00%	0.608	0.00E+00
UGP2	PreFC	99.20%	84.60%	0.602	0.00E+00
PAPPA	PreFC	41.50%	7.80%	0.599	0.00E+00
OSTC	PreFC	99.10%	80.90%	0.593	0.00E+00
NT5E	PreFC	89.20%	41.50%	0.589	0.00E+00
CRLF1	PreFC	71.40%	18.40%	0.588	0.00E+00
COL6A3	PreFC	93.80%	50.40%	0.587	0.00E+00
SERPINE1	PreFC	83.20%	35.50%	0.584	0.00E+00
AMTN	PreFC	33.80%	7.50%	0.560	0.00E+00
AKR1C2	PreFC	82.10%	39.60%	0.549	0.00E+00
LOXL2	PreFC	72.20%	19.90%	0.549	0.00E+00
ANKH	PreFC	95.90%	69.60%	0.537	0.00E+00
CAPS	PreFC	84.10%	33.50%	0.523	0.00E+00
CRTAC1	PreFC	99.40%	70.60%	0.523	0
NOVA1	PreFC	73.90%	33.70%	0.513	0
VCAN	PreFC	70.30%	21.30%	0.512	0
CLIC3	PreFC	45.60%	11.60%	0.509	0
PART1	PreFC	79.50%	38.40%	0.499	0
CILP	PreFC	99.90%	88.70%	0.497	0
SLC7A2	PreFC	75.60%	31.10%	0.494	0
FMOD	PreFC	100.00%	99.10%	0.486	0
COL6A2	PreFC	99.30%	85.50%	0.486	0
DKK3	PreFC	82.80%	36.30%	0.486	0
DNER	PreFC	64.80%	14.60%	0.471	0
CTHRC1	PreFC	92.60%	48.90%	0.468	0
DIO2	PreFC	62.10%	18.20%	0.462	0
FSTL1	PreFC	94.50%	60.70%	0.461	0
TNFRSF12A	PreFC	94.60%	66.10%	0.460	0
COL6A1	PreFC	98.50%	79.90%	0.459	0
LOXL3	PreFC	78.60%	29.00%	0.459	0
EPYC	PreFC	31.20%	4.20%	0.450	0
UROC1	PreFC	57.50%	17.50%	0.447	0
PPIC	PreFC	96.50%	69.60%	0.407	0
COL5A2	PreFC	93.10%	60.70%	0.403	0
PID1	PreFC	93.70%	60.20%	0.403	0
PMP22	PreFC	99.00%	87.70%	0.403	0.00E+00
THY1	PreFC	67.50%	17.10%	0.401	0.00E+00
AK1	PreFC	98.70%	83.80%	0.397	0.00E+00

THBS3	PreFC	76.30%	27.70%	0.395	0.00E+00
CD151	PreFC	97.80%	80.70%	0.384	0.00E+00
ADAMTS6	PreFC	81.20%	35.30%	0.376	0.00E+00
PDLIM7	PreFC	75.20%	25.60%	0.375	0.00E+00
SEC61G	PreFC	99.80%	95.80%	0.373	0.00E+00
FNIP2	PreFC	73.80%	32.90%	0.365	0.00E+00
RPL28	PreFC	100.00%	99.90%	0.360	0.00E+00
IGFBP3	PreFC	28.20%	4.20%	0.358	0.00E+00
DOK1	PreFC	74.60%	33.80%	0.358	0.00E+00
OAZ1	PreFC	100.00%	99.30%	0.357	0.00E+00
C7orf73	PreFC	96.80%	75.00%	0.352	0.00E+00
LEPREL1	PreFC	50.60%	9.80%	0.339	0.00E+00
TMEM167A	PreFC	92.80%	59.90%	0.338	0.00E+00
GAPDH	PreFC	100.00%	100.00%	0.335	0.00E+00
GRN	PreFC	92.50%	63.20%	0.333	0.00E+00
CDON	PreFC	80.90%	38.60%	0.332	0.00E+00
YIF1A	PreFC	92.90%	62.90%	0.330	0.00E+00
CD68	PreFC	78.30%	36.90%	0.328	0.00E+00
ATP5E	PreFC	99.70%	94.70%	0.325	0.00E+00
GALNT15	PreFC	84.10%	45.00%	0.320	0.00E+00
ANK3	PreFC	73.90%	29.80%	0.317	0.00E+00
RABAC1	PreFC	99.90%	95.40%	0.313	0.00E+00
LTBP2	PreFC	73.80%	32.60%	0.313	0.00E+00
RPLP0	PreFC	100.00%	99.90%	0.312	0.00E+00
RPS12	PreFC	100.00%	100.00%	0.304	0.00E+00
FAP	PreFC	68.10%	24.60%	0.302	0.00E+00
BAALC	PreFC	63.60%	18.50%	0.301	0.00E+00
RPSA	PreFC	99.90%	99.20%	0.299	0.00E+00
IGFBP7	PreFC	92.80%	53.10%	0.293	0.00E+00
S100A10	PreFC	100.00%	99.20%	0.289	0.00E+00
SMIM5	PreFC	48.30%	13.60%	0.289	0.00E+00
UBAC2	PreFC	76.80%	39.80%	0.287	0.00E+00
WNT16	PreFC	13.00%	1.20%	0.285	0.00E+00
RP11-456H18.2	PreFC	53.10%	14.80%	0.284	0.00E+00
EVI2A	PreFC	58.40%	19.40%	0.274	0.00E+00
OST4	PreFC	99.90%	97.90%	0.274	0.00E+00
GPR64	PreFC	68.10%	27.30%	0.273	0.00E+00
RPS24	PreFC	100.00%	99.90%	0.270	0.00E+00
RPL27A	PreFC	100.00%	99.90%	0.270	0.00E+00
C10orf105	PreFC	59.00%	14.90%	0.267	0.00E+00
CENPP	PreFC	49.10%	12.20%	0.258	0.00E+00

CXCL14	PreFC	22.70%	4.70%	0.817	6.36E-298
LMAN1	PreFC	87.40%	55.10%	0.316	5.79E-295
PLAUR	PreFC	65.30%	28.70%	0.266	4.26E-294
AEBP1	PreFC	97.70%	81.60%	0.408	4.24E-292
RCAN1	PreFC	92.50%	63.30%	0.461	1.03E-288
PGAM1	PreFC	99.10%	91.00%	0.335	4.57E-283
SLC39A14	PreFC	94.10%	70.20%	0.330	2.84E-280
OMD	PreFC	92.20%	61.30%	0.375	5.47E-279
CDA	PreFC	65.80%	31.40%	0.268	1.18E-274
TM4SF1	PreFC	88.70%	57.90%	0.339	7.32E-272
TAGLN2	PreFC	97.30%	75.90%	0.288	1.70E-271
ERGIC3	PreFC	96.90%	78.80%	0.290	2.03E-263
C19orf10	PreFC	99.50%	90.50%	0.303	4.98E-260
LDHA	PreFC	100.00%	99.60%	0.276	4.83E-256
TRPS1	PreFC	84.10%	50.40%	0.285	3.08E-254
AQP3	PreFC	62.00%	31.30%	0.563	6.05E-243
SPARC	PreFC	100.00%	96.90%	0.501	8.78E-233
KDELR3	PreFC	87.00%	55.80%	0.287	1.54E-230
CLU	PreFC	100.00%	100.00%	0.329	2.28E-229
FNDC3B	PreFC	90.40%	61.30%	0.266	1.68E-228
TMED3	PreFC	93.20%	66.60%	0.267	3.69E-228
COX17	PreFC	93.60%	70.50%	0.318	5.22E-228
SMOC2	PreFC	99.60%	88.50%	0.338	2.43E-226
SEMA3D	PreFC	46.80%	19.70%	0.290	2.38E-223
MRPL33	PreFC	97.70%	85.70%	0.275	1.25E-222
KDELR2	PreFC	99.10%	90.00%	0.266	5.95E-221
SLC16A3	PreFC	93.00%	69.90%	0.300	8.27E-220
SBSPON	PreFC	73.40%	41.00%	0.333	8.50E-220
PRELP	PreFC	100.00%	99.60%	0.257	3.77E-214
PFKP	PreFC	92.10%	64.40%	0.268	3.59E-211
LUM	PreFC	100.00%	99.80%	0.343	3.62E-210
CKB	PreFC	60.70%	32.30%	0.460	4.36E-210
CAV1	PreFC	93.60%	74.10%	0.318	2.20E-206
UGDH	PreFC	86.90%	57.30%	0.251	1.55E-204
ATP1B1	PreFC	88.20%	58.50%	0.271	1.89E-202
P4HB	PreFC	99.90%	96.40%	0.278	2.42E-201
HCFC1R1	PreFC	88.20%	58.70%	0.256	3.60E-201
PCSK1N	PreFC	48.00%	21.10%	0.257	1.69E-199
DST	PreFC	96.60%	77.90%	0.262	1.24E-188
DPT	PreFC	63.20%	32.80%	0.296	9.27E-181
ID2	PreFC	98.70%	90.30%	0.347	1.15E-167

ANGPTL2	PreFC	99.40%	88.10%	0.278	8.30E-167
MATN3	PreFC	77.10%	51.00%	0.383	1.80E-151
S100A13	PreFC	99.90%	97.10%	0.266	5.03E-149
COL11A1	PreFC	95.20%	76.70%	0.272	2.80E-143
ENPP1	PreFC	88.80%	67.00%	0.324	2.26E-128
IGFBP5	PreFC	38.00%	20.20%	0.599	1.23E-99
SPP1	PreFC	55.60%	32.60%	0.755	2.15E-92
CCDC80	PreFC	95.30%	84.30%	0.318	3.88E-89
TIMP1	PreFC	100.00%	99.40%	0.468	2.08E-47
CHI3L2	RegC	98.70%	85.80%	1.498	0.00E+00
IFITM3	RegC	98.90%	96.80%	0.568	7.92E-228
CHI3L1	RegC	93.90%	76.10%	0.953	1.87E-207
VCAM1	RegC	46.90%	15.80%	0.882	7.46E-203
CLU	RegC	99.90%	100.00%	0.605	7.40E-189
NNMT	RegC	99.40%	95.60%	0.506	2.06E-172
TNC	RegC	83.20%	48.60%	0.520	9.23E-150
CRTAC1	RegC	95.10%	72.90%	0.285	1.17E-143
MALAT1	RegC	100.00%	100.00%	0.305	4.49E-124
IFITM2	RegC	91.40%	81.80%	0.475	8.93E-124
BTG1	RegC	96.40%	91.60%	0.497	2.12E-122
EFEMP1	RegC	77.10%	55.00%	0.475	3.69E-118
AEBP1	RegC	91.70%	83.10%	0.481	9.31E-93
C1R	RegC	78.80%	65.60%	0.294	6.33E-71
COL12A1	RegC	54.90%	35.10%	0.390	1.23E-70
OSMR	RegC	70.40%	57.30%	0.357	6.34E-67
ITGB8	RegC	28.20%	12.20%	0.265	4.64E-65
SOD2	RegC	90.20%	84.10%	0.469	4.86E-64
MAFB	RegC	46.50%	27.40%	0.356	9.71E-62
MT2A	RegC	99.90%	99.70%	0.361	1.55E-54
NTN1	RegC	32.90%	17.50%	0.336	4.78E-53
MT1M	RegC	86.70%	81.00%	0.487	2.54E-51
ANK3	RegC	50.40%	34.20%	0.341	1.06E-48
STEAP4	RegC	21.70%	9.20%	0.321	3.30E-46
EGFLAM	RegC	43.90%	29.30%	0.261	4.54E-41
GLRX	RegC	58.40%	47.10%	0.481	2.93E-40
SOCS3	RegC	86.30%	79.30%	0.282	2.97E-38
IRF1	RegC	62.90%	48.90%	0.291	1.70E-37
DIO2	RegC	36.10%	22.70%	0.323	4.70E-32
TCF4	RegC	66.10%	57.90%	0.259	1.93E-31
SLC39A14	RegC	77.80%	72.80%	0.271	7.41E-31
ERRFI1	RegC	80.90%	76.90%	0.307	1.32E-29

ARID5B	RegC	73.50%	63.30%	0.259	2.15E-29
CTGF	RegC	97.80%	97.70%	0.329	2.75E-29
ANGPTL4	RegC	72.00%	61.00%	0.362	1.67E-28
GLI3	RegC	52.70%	42.80%	0.265	1.02E-27
MT1X	RegC	99.80%	99.30%	0.274	1.05E-23
ZFP36L2	RegC	44.30%	34.80%	0.283	2.87E-22
MT1E	RegC	99.10%	98.90%	0.255	3.57E-21
IGFBP5	RegC	32.00%	21.80%	0.809	1.65E-20
NFKBIZ	RegC	57.30%	49.40%	0.254	3.75E-20
SLC40A1	RegC	42.10%	32.70%	0.255	1.53E-18
MT1A	RegC	48.30%	40.20%	0.314	4.37E-17
SCARA3	RegC	58.80%	56.70%	0.285	8.31E-12
HMOX1	RegC	44.70%	37.00%	0.501	1.47E-11
RGS16	RegC	76.00%	73.20%	0.283	4.26E-10
TIMP1	RegC	99.50%	99.50%	0.477	1.84E-07
CTSC	RegC	31.10%	27.70%	0.259	4.11E-03
COL1A1	FC	48.00%	6.70%	2.515	0.00E+00
TMSB4X	FC	95.80%	73.40%	2.336	0.00E+00
PRG4	FC	62.60%	31.80%	2.152	0.00E+00
S100A4	FC	95.00%	54.90%	1.925	0.00E+00
CRTAC1	FC	98.30%	69.60%	1.854	0.00E+00
HTRA1	FC	98.80%	77.80%	1.602	0.00E+00
COL1A2	FC	91.80%	44.00%	1.573	0.00E+00
TGFBI	FC	90.10%	29.10%	1.529	0.00E+00
TPPP3	FC	69.80%	19.00%	1.479	0.00E+00
TNFAIP6	FC	60.20%	12.70%	1.398	0.00E+00
DPT	FC	73.90%	29.60%	1.301	0.00E+00
TNC	FC	93.80%	42.40%	1.252	0.00E+00
ASPN	FC	90.90%	54.20%	1.251	0.00E+00
LGALS1	FC	95.10%	91.80%	1.240	0.00E+00
AQP1	FC	48.90%	3.50%	1.207	0.00E+00
COL6A3	FC	86.50%	49.90%	1.184	0.00E+00
COL6A1	FC	95.10%	79.70%	1.124	0.00E+00
TIMP3	FC	73.20%	50.90%	1.076	0.00E+00
COL6A2	FC	97.60%	85.20%	1.058	0.00E+00
PRSS23	FC	59.10%	21.80%	1.057	0.00E+00
CD55	FC	84.80%	68.30%	1.056	0.00E+00
SPARCL1	FC	21.70%	1.20%	1.050	0.00E+00
THY1	FC	67.70%	14.90%	1.040	0.00E+00
CRLF1	FC	64.40%	17.40%	1.011	0.00E+00
FN1	FC	99.90%	99.90%	0.963	0.00E+00

TNXB	FC	68.10%	24.80%	0.940	0.00E+00
NBL1	FC	58.20%	8.90%	0.923	0.00E+00
S100A10	FC	99.80%	99.20%	0.861	0.00E+00
TAGLN2	FC	97.10%	75.00%	0.848	0.00E+00
ISLR	FC	89.60%	71.30%	0.833	0.00E+00
COL3A1	FC	96.50%	73.40%	0.804	0.00E+00
CRIP1	FC	72.30%	27.50%	0.794	0.00E+00
PTGES	FC	67.20%	31.10%	0.787	0.00E+00
ABI3BP	FC	88.00%	40.40%	0.784	0.00E+00
CFD	FC	22.80%	2.10%	0.781	0.00E+00
S100A6	FC	100.00%	99.90%	0.767	0.00E+00
SH3BGRL3	FC	99.30%	95.90%	0.767	0.00E+00
CALD1	FC	91.70%	75.00%	0.762	0.00E+00
VCAN	FC	52.00%	22.50%	0.732	0.00E+00
S100A11	FC	99.20%	95.60%	0.728	0.00E+00
COL15A1	FC	74.90%	28.70%	0.715	0.00E+00
EMP3	FC	97.50%	91.10%	0.714	0.00E+00
CCND1	FC	54.60%	8.30%	0.713	0.00E+00
ECM1	FC	54.20%	7.90%	0.694	0.00E+00
PFN1	FC	97.00%	86.20%	0.683	0.00E+00
MXRA5	FC	55.60%	17.20%	0.683	0.00E+00
MMP2	FC	22.30%	3.90%	0.668	0.00E+00
FSTL1	FC	86.50%	60.70%	0.664	0.00E+00
MGST3	FC	92.90%	78.10%	0.661	0.00E+00
IGFBP4	FC	54.10%	13.50%	0.661	0.00E+00
ITGB5	FC	64.00%	22.50%	0.660	0.00E+00
TREM1	FC	37.10%	4.10%	0.651	0.00E+00
DKK3	FC	71.10%	36.40%	0.641	0.00E+00
CAPZB	FC	84.70%	58.90%	0.634	0.00E+00
ATP5E	FC	99.30%	94.60%	0.628	0.00E+00
CD151	FC	95.20%	80.40%	0.628	0.00E+00
MT-CO2	FC	94.50%	95.00%	0.606	0.00E+00
ARPC1B	FC	85.40%	56.70%	0.591	0.00E+00
CAPS	FC	71.60%	33.60%	0.591	0.00E+00
MT-ATP6	FC	94.40%	95.00%	0.589	0.00E+00
TIMP2	FC	86.30%	68.10%	0.588	0.00E+00
COL5A1	FC	80.10%	46.70%	0.585	0.00E+00
CAPG	FC	78.20%	56.80%	0.571	0.00E+00
FAP	FC	67.50%	22.90%	0.566	0.00E+00
HSD3B7	FC	47.70%	8.70%	0.562	0.00E+00
PLAUR	FC	65.90%	27.10%	0.561	0.00E+00

LUM	FC	99.60%	99.90%	0.559	0.00E+00
PRKCDBP	FC	91.10%	73.40%	0.559	0.00E+00
PDLIM7	FC	67.00%	25.00%	0.557	0.00E+00
CLIC1	FC	96.00%	84.70%	0.552	0.00E+00
TXN	FC	96.00%	85.70%	0.547	0.00E+00
ADAMTS6	FC	67.60%	35.80%	0.544	0.00E+00
PROCR	FC	72.30%	47.50%	0.538	0.00E+00
CRIP2	FC	91.70%	74.50%	0.536	0.00E+00
PPAP2B	FC	48.80%	17.60%	0.531	0.00E+00
ADAMTS5	FC	40.60%	9.00%	0.522	0.00E+00
MINOS1	FC	91.30%	73.90%	0.522	0.00E+00
IFITM3	FC	99.20%	96.50%	0.520	0.00E+00
PLP2	FC	95.80%	85.70%	0.514	0.00E+00
MT-CYB	FC	93.70%	94.80%	0.512	0.00E+00
SMOC1	FC	76.10%	41.70%	0.504	0.00E+00
MYL6	FC	99.70%	98.20%	0.503	0.00E+00
CRABP2	FC	23.20%	3.50%	0.501	0.00E+00
COL4A1	FC	16.20%	1.30%	0.500	0.00E+00
ANXA2	FC	99.20%	97.60%	0.498	0.00E+00
VCAM1	FC	41.10%	13.00%	0.485	0.00E+00
GJA1	FC	59.50%	20.70%	0.483	0.00E+00
AHNAK	FC	92.30%	78.10%	0.481	0.00E+00
SEPW1	FC	92.40%	74.00%	0.481	0.00E+00
POLR2L	FC	96.10%	85.80%	0.480	0.00E+00
NT5E	FC	75.60%	41.90%	0.480	0.00E+00
FAM134B	FC	72.70%	40.10%	0.475	0.00E+00
COL14A1	FC	25.50%	5.50%	0.473	0.00E+00
ABHD2	FC	54.10%	20.00%	0.467	0.00E+00
ANKH	FC	87.60%	70.00%	0.466	0.00E+00
CERCAM	FC	49.50%	17.20%	0.466	0.00E+00
TCF4	FC	80.60%	54.20%	0.456	0.00E+00
SERPINF1	FC	15.60%	1.10%	0.456	0.00E+00
PMP22	FC	97.00%	87.60%	0.452	0.00E+00
TMEM100	FC	48.90%	13.60%	0.449	0.00E+00
HCFC1R1	FC	84.00%	58.20%	0.448	0.00E+00
CD68	FC	69.00%	36.80%	0.446	0.00E+00
RPS27L	FC	96.60%	88.70%	0.443	0.00E+00
NDUFB2	FC	94.30%	79.90%	0.442	0.00E+00
MT-ND2	FC	94.50%	95.10%	0.441	0.00E+00
NDUFS8	FC	68.80%	40.10%	0.440	0.00E+00
THBS3	FC	65.50%	27.60%	0.440	0.00E+00

BLOC1S1	FC	66.60%	34.20%	0.437	0.00E+00
P4HA3	FC	52.30%	12.50%	0.432	0.00E+00
TMEM50A	FC	77.20%	50.30%	0.431	0.00E+00
MRC2	FC	76.10%	47.90%	0.418	0.00E+00
COL4A2	FC	19.70%	1.50%	0.412	0.00E+00
LTBP2	FC	62.30%	32.90%	0.411	0.00E+00
MAP1A	FC	43.10%	8.20%	0.410	0.00E+00
C10orf105	FC	49.60%	14.70%	0.406	0.00E+00
CD109	FC	68.50%	39.60%	0.399	0.00E+00
DPYSL2	FC	61.90%	29.60%	0.398	0.00E+00
FNIP2	FC	60.60%	33.60%	0.392	0.00E+00
AKR1C2	FC	69.80%	40.00%	0.392	0.00E+00
NDUFS5	FC	95.30%	88.50%	0.387	0.00E+00
F5	FC	44.20%	10.60%	0.380	0.00E+00
CNIH1	FC	83.20%	63.50%	0.380	0.00E+00
SORBS2	FC	43.90%	13.90%	0.378	0.00E+00
LTBP3	FC	71.20%	46.70%	0.377	0.00E+00
RAB31	FC	41.20%	11.60%	0.375	0.00E+00
PLEC	FC	56.10%	28.40%	0.375	0.00E+00
EZR	FC	44.30%	17.60%	0.366	0.00E+00
GPNMB	FC	19.30%	1.30%	0.366	0.00E+00
ACKR3	FC	44.40%	18.00%	0.357	0.00E+00
COX7B	FC	92.70%	80.00%	0.355	0.00E+00
ABL2	FC	43.70%	17.50%	0.348	0.00E+00
RPL28	FC	99.90%	99.90%	0.348	0.00E+00
HMGB1	FC	98.50%	96.00%	0.348	0.00E+00
ZEB2	FC	33.70%	2.30%	0.347	0.00E+00
MGST1	FC	30.70%	6.10%	0.344	0.00E+00
CD63	FC	100.00%	99.70%	0.343	0.00E+00
SEMA3C	FC	32.00%	6.50%	0.341	0.00E+00
CDH13	FC	32.50%	5.00%	0.331	0.00E+00
ENG	FC	53.80%	25.50%	0.331	0.00E+00
COL18A1	FC	20.50%	3.00%	0.329	0.00E+00
NDUFA11	FC	96.60%	87.80%	0.326	0.00E+00
COX6B1	FC	96.90%	89.40%	0.325	0.00E+00
PHACTR2	FC	49.90%	21.90%	0.308	0.00E+00
TAX1BP3	FC	47.30%	20.80%	0.305	0.00E+00
MAMDC2	FC	32.20%	3.10%	0.302	0.00E+00
PTPRD	FC	47.50%	17.70%	0.299	0.00E+00
COX4I1	FC	98.40%	94.90%	0.299	0.00E+00
TNS1	FC	50.40%	23.60%	0.298	0.00E+00

ANTXR1	FC	43.50%	18.30%	0.296	0.00E+00
PDGFRB	FC	26.00%	6.70%	0.293	0.00E+00
KCNN4	FC	29.80%	6.00%	0.291	0.00E+00
ANGPTL1	FC	32.90%	9.30%	0.287	0.00E+00
SSC5D	FC	39.70%	13.00%	0.286	0.00E+00
SULF1	FC	37.40%	10.60%	0.286	0.00E+00
COL8A1	FC	16.80%	2.10%	0.285	0.00E+00
ANPEP	FC	23.60%	0.80%	0.281	0.00E+00
S100A3	FC	33.90%	11.50%	0.267	0.00E+00
OAZ1	FC	99.70%	99.30%	0.266	0.00E+00
DYSF	FC	26.90%	7.40%	0.266	0.00E+00
ITGB8	FC	31.30%	9.60%	0.257	0.00E+00
PXDN	FC	44.30%	18.70%	0.256	0.00E+00
ODF3B	FC	38.90%	10.80%	0.251	0.00E+00
PDLIM1	FC	19.50%	4.10%	0.327	3.87E-303
LTBP1	FC	59.70%	34.20%	0.430	1.23E-302
NRP2	FC	55.20%	27.70%	0.323	2.44E-302
MVP	FC	57.30%	31.10%	0.313	5.16E-297
AKR1C1	FC	34.00%	12.10%	0.332	3.55E-296
CUX1	FC	55.00%	28.30%	0.269	1.22E-295
ATP6V0E1	FC	94.80%	84.40%	0.330	3.60E-295
DNAJC15	FC	74.40%	50.70%	0.325	2.22E-294
DEFB1	FC	20.10%	4.50%	0.434	3.69E-294
VIM	FC	100.00%	100.00%	0.456	5.14E-291
AXL	FC	52.00%	26.00%	0.328	4.33E-289
UQCRQ	FC	95.40%	86.80%	0.316	1.04E-288
NTN4	FC	40.70%	16.70%	0.263	3.26E-282
RPS26	FC	99.50%	97.70%	0.409	5.58E-282
GADD45GIP1	FC	89.50%	75.10%	0.315	7.29E-282
NAV1	FC	44.70%	20.50%	0.258	3.08E-279
C10orf54	FC	69.50%	44.60%	0.347	3.04E-278
ATL3	FC	46.20%	21.70%	0.263	4.15E-278
CD81	FC	75.10%	52.80%	0.423	6.45E-278
ZNHIT1	FC	83.40%	65.70%	0.334	1.85E-276
HSPG2	FC	59.10%	33.70%	0.372	3.45E-275
RABAC1	FC	99.40%	95.30%	0.340	9.15E-273
HHIP	FC	20.50%	5.00%	0.268	2.83E-272
RAB13	FC	87.80%	73.00%	0.345	1.51E-270
COX5B	FC	96.80%	91.20%	0.285	5.39E-269
PDGFRA	FC	48.60%	24.10%	0.319	1.20E-268
AK1	FC	94.20%	84.00%	0.410	2.54E-267

NDUFS6	FC	80.90%	61.70%	0.328	1.80E-266
NOP10	FC	87.00%	71.40%	0.326	3.04E-266
ADAMTS1	FC	38.10%	15.80%	0.447	1.49E-265
SLC7A2	FC	56.30%	32.70%	0.456	1.53E-265
FXYD5	FC	97.60%	93.30%	0.316	1.52E-263
IFI27	FC	31.70%	11.70%	0.575	5.79E-263
ATP5J2	FC	88.90%	74.50%	0.345	2.30E-261
PFKP	FC	83.30%	64.80%	0.400	2.41E-261
POSTN	FC	18.00%	3.90%	0.599	6.38E-261
IFITM2	FC	94.00%	80.20%	0.357	8.29E-261
AP2S1	FC	78.50%	59.70%	0.348	1.70E-257
IGFBP5	FC	41.70%	18.80%	0.724	1.43E-253
CYBA	FC	81.00%	64.40%	0.406	5.85E-253
ANKRD28	FC	66.10%	41.40%	0.325	8.27E-252
SIGIRR	FC	50.40%	25.90%	0.253	9.31E-252
HIGD2A	FC	64.00%	40.10%	0.293	2.2994E-250
APRT	FC	75.00%	54.20%	0.335	2.8629E-250
TMSB10	FC	87.60%	75.70%	0.984	4.0055E-249
ARPC3	FC	93.30%	82.80%	0.307	9.409E-249
PSMA7	FC	94.10%	85.80%	0.357	6.6333E-248
ACTA2	FC	11.50%	1.60%	1.053	2.1573E-245
ITGB1	FC	88.10%	75.00%	0.409	4.7927E-245
RBX1	FC	88.50%	74.90%	0.319	9.0451E-245
GPX1	FC	87.40%	74.00%	0.365	3.4611E-244
MYL12B	FC	94.10%	87.00%	0.367	3.7991E-244
DSTN	FC	99.80%	99.00%	0.317	8.2799E-244
REXO2	FC	93.60%	84.70%	0.318	3.6346E-243
ATP5H	FC	90.80%	78.50%	0.287	3.219E-241
PTMA	FC	99.90%	99.90%	0.280	2.6406E-240
ATP6AP2	FC	78.80%	60.00%	0.333	1.8974E-237
PSD3	FC	62.90%	39.50%	0.290	5.6642E-236
NDUFA13	FC	87.50%	71.50%	0.294	1.6852E-235
GYPC	FC	58.60%	35.30%	0.304	1.4346E-234
FKBP1A	FC	75.80%	56.90%	0.358	1.1672E-233
SLC39A14	FC	83.30%	71.10%	0.393	2.3647E-231
OGN	FC	94.30%	77.80%	0.342	4.9679E-231
CSTB	FC	92.80%	81.00%	0.356	3.0936E-230
IGFBP7	FC	79.70%	53.80%	0.561	1.8771E-228
C1R	FC	81.30%	63.40%	0.609	2.3967E-228
LTBP4	FC	58.60%	35.10%	0.273	1.3248E-224
DYNLL1	FC	97.80%	93.80%	0.295	4.8556E-224

ARPC5	FC	75.90%	56.80%	0.337	1.9425E-223
TCEB2	FC	97.50%	92.50%	0.277	3.3513E-219
MYH9	FC	40.90%	20.10%	0.313	9.1562E-219
YWHAB	FC	81.00%	64.70%	0.307	2.3685E-218
LMAN1	FC	75.60%	55.90%	0.296	2.4376E-218
PDLIM3	FC	43.50%	21.30%	0.298	9.9579E-218
IGF1	FC	11.60%	1.90%	0.494	8.3089E-215
PRDX1	FC	98.10%	95.10%	0.366	4.9405E-212
PART1	FC	62.70%	39.70%	0.397	1.7648E-210
FLNA	FC	49.00%	27.80%	0.434	2.8678E-209
SPTBN1	FC	51.30%	29.60%	0.276	6.4566E-207
ANXA4	FC	71.20%	51.70%	0.366	2.9558E-206
AKR1B1	FC	59.50%	37.80%	0.302	4.5637E-206
LAMB2	FC	67.00%	44.90%	0.283	4.6259E-206
PLTP	FC	30.30%	12.40%	0.269	7.8301E-206
CIB1	FC	84.00%	71.40%	0.321	4.603E-205
TIMP1	FC	99.80%	99.40%	0.606	1.7529E-204
APLP2	FC	85.90%	72.20%	0.296	2.5065E-202
PCOLCE	FC	90.80%	80.20%	0.504	4.98E-200
MPG	FC	75.10%	54.80%	0.261	1.20E-198
PSMB3	FC	82.00%	66.00%	0.280	5.52E-198
GALNT15	FC	67.80%	46.30%	0.320	8.22E-197
SMIM14	FC	79.60%	65.70%	0.407	2.14E-196
PSME1	FC	68.40%	48.30%	0.307	7.60E-196
USMG5	FC	95.90%	88.70%	0.256	8.42E-196
CST3	FC	99.90%	99.60%	0.260	2.95E-195
GFPT2	FC	47.70%	27.40%	0.460	1.66E-192
CAST	FC	73.20%	53.70%	0.286	1.74E-192
C9orf16	FC	77.80%	60.80%	0.296	9.89E-190
CAV1	FC	86.30%	74.60%	0.429	1.62E-188
RNH1	FC	83.30%	71.20%	0.315	9.10E-187
RAC1	FC	63.00%	42.30%	0.258	9.49E-187
DRAP1	FC	79.30%	63.30%	0.282	1.30E-186
ARL2	FC	71.90%	54.60%	0.357	5.81E-186
MT-CO3	FC	94.60%	95.10%	0.390	8.00E-186
CDON	FC	62.20%	40.20%	0.280	1.40E-183
ATOX1	FC	82.90%	66.40%	0.265	1.71E-183
PRELID1	FC	85.40%	72.40%	0.251	1.88E-183
CTSA	FC	63.10%	42.10%	0.258	9.41E-183
MRPL41	FC	75.50%	58.10%	0.267	1.01E-181
ITGAV	FC	58.30%	37.60%	0.260	1.87E-181

UGDH	FC	75.20%	58.10%	0.321	2.43E-180
NEAT1	FC	99.80%	99.50%	0.310	7.42E-180
MSN	FC	65.90%	47.20%	0.299	3.26E-179
CLU	FC	100.00%	100.00%	0.451	3.42E-179
CCND2	FC	54.50%	33.80%	0.261	3.67E-179
FAM127A	FC	80.20%	63.20%	0.257	4.67E-179
DIXDC1	FC	71.30%	52.40%	0.262	4.43E-178
TPM3	FC	74.30%	56.40%	0.276	6.13E-178
MT-ND3	FC	93.30%	93.50%	0.371	3.34E-176
GAS1	FC	62.20%	37.70%	0.307	4.16E-175
ARL6IP5	FC	95.90%	90.90%	0.281	8.17E-174
MMP14	FC	35.10%	16.80%	0.268	8.53E-174
TM4SF1	FC	78.00%	58.60%	0.316	8.19E-172
CD44	FC	80.20%	65.30%	0.364	2.35E-169
TRPS1	FC	70.70%	51.30%	0.271	1.66E-166
POMP	FC	91.50%	82.50%	0.265	3.26E-164
FAM114A1	FC	82.10%	67.80%	0.265	4.13E-164
RHOA	FC	94.10%	88.30%	0.298	5.09E-164
YIF1A	FC	80.50%	63.90%	0.262	5.27E-164
SDF2L1	FC	56.50%	36.90%	0.261	1.09E-163
RP11-572C15.6	FC	49.00%	30.50%	0.353	1.43E-162
FOXO1	FC	50.10%	31.30%	0.268	1.68E-162
SEP15	FC	78.50%	63.40%	0.253	2.35E-162
PSMD8	FC	80.40%	66.60%	0.251	1.12E-161
GNG11	FC	68.90%	49.50%	0.328	4.32E-159
IQGAP1	FC	72.20%	55.40%	0.259	5.32E-159
NDUFB7	FC	91.30%	81.20%	0.251	5.34E-159
MFAP4	FC	30.20%	14.10%	0.263	2.02E-158
DST	FC	90.30%	78.30%	0.259	2.59E-158
ATP5G1	FC	81.20%	67.10%	0.253	1.05E-153
FBN1	FC	45.90%	27.70%	0.298	1.34E-152
SERPINE2	FC	78.60%	57.30%	0.377	1.69E-152
COL5A2	FC	81.30%	61.50%	0.399	4.22E-150
TUBA1B	FC	93.90%	88.90%	0.355	6.01E-150
IGFBP6	FC	82.50%	66.90%	0.545	1.33E-146
LMNA	FC	97.50%	93.50%	0.344	1.45E-143
GRN	FC	81.10%	64.10%	0.276	5.43E-142
MYL9	FC	42.90%	26.70%	0.911	4.89E-139
ARPC2	FC	92.80%	86.40%	0.270	2.48E-136
LPP	FC	48.40%	30.30%	0.262	2.90E-133
COX7A1	FC	90.90%	84.10%	0.259	2.99E-132

MT-ND4	FC	95.00%	95.50%	0.285	1.62E-129
EFEMP1	FC	67.30%	54.10%	0.376	4.38E-129
PTRF	FC	82.80%	72.40%	0.361	2.86E-128
ADIRF	FC	95.40%	91.20%	0.449	3.00E-127
43350	FC	90.20%	82.80%	0.269	8.48E-126
MSMP	FC	15.90%	5.50%	0.960	2.59E-122
MT-ND1	FC	93.20%	94.10%	0.288	2.04E-116
S100A16	FC	48.90%	34.20%	0.283	5.48E-109
DDAH2	FC	57.90%	42.70%	0.257	7.26E-107
CAPN2	FC	56.90%	42.30%	0.259	7.57E-104
CCDC80	FC	90.00%	84.80%	0.504	1.13E-101
FNDC1	FC	24.70%	13.00%	0.275	3.20E-91
CDA	FC	46.30%	33.40%	0.269	1.43E-78
CD9	FC	96.20%	94.70%	0.496	2.11E-73
UAP1	FC	73.20%	66.20%	0.272	1.02E-65
PPAP2A	FC	35.00%	24.10%	0.276	4.48E-61
WISP2	FC	10.60%	4.40%	0.409	2.79E-59
MT1G	FC	82.60%	72.30%	0.295	2.88E-57
ENPP1	FC	76.60%	68.30%	0.299	2.51E-54
PTN	FC	30.30%	21.10%	0.323	2.39E-44
RGS2	FC	53.80%	44.40%	0.312	1.08E-39
AMTN	FC	15.80%	9.60%	0.474	1.70E-31
FABP3	FC	12.70%	7.30%	0.306	2.76E-28
INHBA	FC	75.10%	67.60%	0.251	4.42E-18
CYP1B1	FC	38.40%	35.80%	0.344	1.52E-09
TPM1	FC	34.60%	31.80%	0.301	9.67E-03
CD74	FC	16.20%	14.10%	0.494	1.00E+00

Supplementary Table S5A. List of cytokines predicted to regulate the chondrocyte cell phenotypes.

Upstream Regulator (cytokine)	Activation z-scores in HomC	Activation z-scores in HTC	Activation z-scores in preHTC	Activation z-scores in RepC	Activation z-scores in RegC	Activation z-scores in preFC	Activation z-scores in FC	SUM (z-score)	Expressed by chondrocytes (%)	Expressed by synoviocytes (%)	Expressed by HLA-DRA+ cells (%)
TNF	5.733	3.988	2.199		2.53	2.948	3.923	21.321	Not detected	16.16	60.26
IL6	4.952	3.256			3.425	2.706	3.929	18.268	Not detected	36.17	25.44
IL1B	5.738	2.338			2.667	2.488	3.778	17.009	Not detected	24.44	61.88
IL1A	3.513	2.756			2.406	2.626	3.514	14.815	Not detected	4.74	29.91
EDN1	3.279	2.6	2.19			2.6	4.058	14.727	Not detected	1.93	1.98
OSM	3.551	3.172	2.372		2.442	2.329		13.866	Not detected	3.17	17.38
PRL	2.423			2.216	2.131	2.034	4.504	13.308	Not detected	0.11	0.00
IFNG	5.684	2.597				2.585	2.31	13.176	Not detected	0.22	0.22
IL4	2.298	2.605				2.877	4.976	12.756	Not detected	0.20	0.15
IL5	2.619	2.8				2.985	4	12.404	Not detected	0.16	0.00
TNFSF11	2.522	2.214		2.219		2.95	2.395	12.3	9.87	2.83	0.59
CSF2	3.475	2.5			2.216		2.742	10.933	Not detected	0.73	1.98
CSF1	2.42	2.916	2.352				3.087	10.775	7.81	29.09	6.38
IFNA2	3.217	2.403			2.828		2.168	10.616	Not detected	Not detected	Not detected
IL3	2.271	2.391	2.188				2.433	9.283	Not detected	Not detected	Not detected
CXCL12	3.179	2.573					3.242	8.994	Not detected	47.13	14.74
IL17A	3.389				2.649		2.157	8.195	Not detected	Not detected	Not detected
IL2	3.502				2.189	2.449		8.14	Not detected	0.03	0.00
IL15					2.429	2.438	2.97	7.837	Not detected	4.72	8.43
WNT1				2.207		2.18	2.876	7.263	Not detected	Not detected	Not detected
CD40LG	2.243	2.593	2.2					7.036	Not detected	0.24	0.22
IL18	3.027	2.449						5.476	Not detected	5.34	35.56
EPO	2.784	2.401						5.185	Not detected	0.05	0.07
LIF	3.105	2.053						5.158	8.09	14.17	4.91
IL13		2.327					2.284	4.611	Not detected	0.09	0.00
C5	2.155	2						4.155	Not detected	1.45	1.17
IL27	2.313							2.313	Not detected	0.08	0.37
CCL5							2.236	2.236	Not detected	2.84	4.91
SPP1							2.225	2.225	35.34	9.70	12.98
TNFSF13B	2.219							2.219	Not detected	9.66	31.89
IL11			2					2	6.53	7.27	2.86

The activation z-score determined by IPA representing the likelihood of the predicted transcriptional regulator to activate the downstream gene expression

Supplementary Table S5B. List of growth factors predicted to regulate the chondrocyte cell phenotypes.

Upstream Regulator (cytokine)	Activation z-scores in HomC	Activation z-scores in HTC	Activation z-scores in preHTC	Activation z-scores in RepC	Activation z-scores in RegC	Activation z-scores in preFC	Activation z-scores in FC	SUM (z-score)	Expressed by chondrocytes (%)	Expressed by synoviocytes (%)	Expressed by HLA-DRA+ cells (%)
TGFB1	3.732	3.433	3.601	2.526		6.42	7.36	27.072	46.54	43.62	50.29
EGF	4.346	4.751	3.665		2.798	3.121	3.928	22.609	Not detected	0.45	0.15
AGT	3.154	3.585	2.584	2.188	2.231	2.974	4.228	20.944	26.54	4.46	0.59
IGF1	3.42	4.252	3.762		2.037	3.64	3.204	20.315	Not detected	42.34	18.77
TGFB3	2.771	3.092	2.611	2.366		3.381	3.398	17.619	14.87	18.10	2.49
GDF2	2.151	2.449	2.63	2.166		2.74	3.426	15.562	Not detected	Not detected	Not detected
FGF2	3.249	3.926	3.063			2.811	2.482	15.531	48.74	14.82	1.47
BMP2	3.073	3.791	3.398	2.386		2.288		14.936	33.34	17.03	3.96
VEGFA	3.206	2.507	2.777		2.563		3.548	14.601	43.71	22.58	27.79
BMP4	3.177	3.066	2.378	2.183		2.183		12.987	5.19	25.07	2.57
HGF	2.819	3.645	2.608				2.31	11.382	Not detected	6.54	5.13
NRG1	2.461	2.215	2.195				3.105	9.976	Not detected	0.07	0.15
ANGPT2	2.718					2.95	3.652	9.32	Not detected	6.20	1.17
CTGF				2.208		2.21	3.09	7.508	97.66	74.99	20.60
KITLG	2.61	2.414	2.201					7.225	8.93	14.23	4.18
LEP	3.095						3.243	6.338	Not detected	0.09	0.07
TGFB2						2.443	2.876	5.319	9.44	13.71	5.28
NGF	2.879	2.189						5.068	23.58	8.64	0.88
GH1	2.213	2.368						4.581	Not detected	0.23	0.00
PDGFC							2.78	2.78	19.52	18.01	13.42
PDGFB	2.602							2.602	Not detected	4.81	27.27
FGF10							2.425	2.425	Not detected	27.71	3.74
FGF7							2.236	2.236	5.42	17.64	2.13
INHBB						2.213		2.213	Not detected	2.06	0.51
BMP6						2.207		2.207	Not detected	6.95	0.95
FGF8							2.177	2.177	Not detected	0.08	0.00
IGF2						2.176		2.176	29.51	3.90	0.73
GDF9							2.117	2.117	Not detected	0.99	0.95
BDNF		2.103						2.103	Not detected	3.32	0.29
FGF1			2.088					2.088	46.03	1.42	0.15

The activation z-score determined by IPA representing the likelihood of the predicted transcriptional regulator to activate the downstream gene expression

Supplementary Table S6. List of OA-related regulators in osteoarthritic synoviocytes and chondrocytes.

	SY	SY	OLT	MT	CHONDRO
**Gene**	**avg expression**	**% cells expressing**	**avg expression**	**avg expression**	**% cells expressing**
**CYTOKINES**					
TNF	0.85755126	16.16	0.00158187	0.03766949	< 1%
IL6	1.77934731	36.17	0.01119925	0.07079241	< 1%
IL1B	7.22218732	24.44	0.0010367	0.02481688	< 1%
IL1A	0.20739783	4.74	Not detected	Not detected	< 1%
EDN1	0.08942337	1.93	0.00094584	0.05532706	< 1%
OSM	0.11960387	3.17	0.0003565	0.00888218	< 1%
PRL	0.00169112	< 1%	0.00109393	0.00138929	< 1%
IFNG	0.02400214	< 1%	0.00060181	0.00016810	< 1%
IL4	0.0010907	< 1%	0.00207364	0.00186238	< 1%
IL5	0.11254458	< 1%	Not detected	0.00046291	< 1%
TNFSF11	0.02624252	2.83	0.03435381	0.23667652	9.87
CSF2	0.12217304	< 1%	Not detected	Not detected	< 1%
CSF1	0.55304604	29.09	0.08907942	0.12565541	7.81
IFNA2	Not detected	< 1%	Not detected	Not detected	< 1%
IL3	Not detected	< 1%	Not detected	Not detected	< 1%
CXCL12	2.56053784	47.13	0.00396949	0.24792134	< 1%
IL17A	Not detected	< 1%	Not detected	Not detected	< 1%
IL2	0.00388308	< 1%	Not detected	Not detected	< 1%
IL15	0.06608152	4.72	0.00567904	0.01085935	< 1%
WNT1	Not detected	< 1%	Not detected	0.00035611	< 1%
CD40LG	0.01879786	< 1%	0.00032335	Not detected	< 1%
IL18	0.14333699	5.34	0.00162556	0.03775048	< 1%
EPO	0.00059691	< 1%	0.00358862	0.00232851	< 1%
LIF	0.48357187	14.17	0.20934401	0.15121719	8.09
IL13	0.01519629	< 1%	0.00054818	0.00020048	< 1%
C5	0.01577531	1.45	0.02840462	0.02542117	< 1%
IL27	0.00062502	< 1%	Not detected	Not detected	< 1%
CCL5	0.67710738	2.84	0.01202828	0.01702966	< 1%

SPP1	0.70833395	9.70	13.8545432	26.41631276	35.34
TNFSF13B	0.16009459	9.66	0.03461985	0.02889442	< 1%
IL11	0.21164188	7.27	0.00392056	2.60355190	6.53
**GROWTH FACTORS**					
TGFB1	0.85413248	43.62	0.64097183	0.76153085	46.54
EGF	0.0050613	< 1%	0.00124841	0.00121434	< 1%
AGT	0.14546452	4.46	0.28149366	0.22654131	26.54
IGF1	2.31677832	42.34	0.04392524	0.21918110	< 1%
TGFB3	0.17344237	18.10	0.12960505	0.16154326	14.87
GDF2	Not detected	< 1%	Not detected	Not detected	< 1%
FGF2	0.23692075	14.82	0.57439937	0.75613763	48.74
BMP2	0.47167904	17.03	0.45676802	0.53301685	33.34
VEGFA	0.40352567	22.58	0.58457432	0.51546246	43.71
BMP4	0.31115601	25.07	0.04071335	0.09364109	5.19
HGF	0.07877636	6.54	0.00077658	0.00950491	< 1%
NRG1	0.00086273	< 1%	0.00062108	0.00021699	< 1%
ANGPT2	0.30437288	6.20	0.02917789	0.14393222	< 1%
CTGF	4.91512698	74.99	18.684124	18.54246749	97.66
KITLG	0.16979471	14.23	0.09307919	0.07805551	8.93
LEP	0.00091981	< 1%	0.0003673	0.00021697	< 1%
TGFB2	0.12556261	13.71	0.07757924	0.11088658	9.44
NGF	0.1631364	8.64	0.05681832	0.71832369	23.58
GH1	0.00213602	< 1%	0.01320848	0.00505116	< 1%
PDGFC	0.20191683	18.01	0.13129045	0.25070483	19.52
PDGFB	0.1137311	4.81	Not detected	0.03299134	< 1%
FGF10	0.32961021	27.71	0.00214966	0.01758111	< 1%
FGF7	0.30732014	17.64	0.09392485	0.12079376	5.42
INHBB	0.02378681	2.06	0.00060804	0.00980327	< 1%
BMP6	0.07168878	6.95	0.01382435	0.06153321	< 1%
FGF8	0.00031855	< 1%	0.00031342	0.00007784	< 1%
IGF2	0.17133688	3.90	0.42811444	0.68274656	29.51
GDF9	0.01079508	< 1%	0.00358594	0.00155076	< 1%
BDNF	0.02433709	3.32	0.00827004	0.02062711	< 1%
FGF1	0.01640977	1.42	0.53699795	0.80027638	46.03
**PROTEASES and OTHER**					
ADAM8	0.08625243	2.49	0.00529755	0.01958402	< 1%
ADAM12	0.16411192	8.35	0.03152764	0.27939445	12.05
ADAM14	Not detected	< 1%	Not detected	Not detected	< 1%
ADAM15	0.52027929	31.09	0.1350985	0.31266840	18.37

ADAM16	Not detected	< 1%	Not detected	Not detected	< 1%
ADAM17	0.43070682	28.66	0.1910278	0.17827930	18.43
ADAMTS1	1.2399157	43.04	0.22650991	0.73930483	19.21
ADAMTS4	0.39328888	11.53	0.00193909	0.07353123	< 1%
ADAMTS5	0.41241595	21.58	0.08477879	0.37566779	13.92
ADAMTS8	0.00199743	< 1%	Not detected	Not detected	< 1%
ADAMTS9	0.23975834	5.78	0.01049355	0.09333423	< 1%
ADAMTS14	0.00547353	< 1%	0.00095825	0.01041145	< 1%
ADAMTS15	0.00820091	< 1%	0.00018986	0.00147547	< 1%
ADAMTS16	0.02716274	2.83	0.00030659	0.00242930	< 1%
ADAMTS17	0.01571554	2.20	0.00329598	0.00998285	< 1%
ADAMTS18	0.00330092	< 1%	0.00066559	0.00533063	< 1%
MMP1	0.114642	2.50	0.01584109	0.00481755	< 1%
MMP2	2.28396669	60.55	0.08560122	0.47811906	6.71
MMP3	4.00612688	24.80	9.9199593	1.54325759	54.17
MMP8	Not detected	< 1%	Not detected	Not detected	< 1%
MMP9	0.18145261	5.47	0.00125271	0.06618949	< 1%
MMP13	0.008894	< 1%	0.01022954	0.02066082	< 1%
MMP14	0.6032863	42.91	0.17960964	0.45239280	19.63
MMP15	0.00499547	<1%	0.00134058	0.00949421	<1%
MMP28	0.06100635	5.65	0.0004565	0.04523445	< 1%
ST14	0.02919843	2.23	0.00056512	0.00533437	< 1%
HTRA1	15.0445909	82.69	3.90133863	18.52409127	81.04
PLAU	0.84355472	34.95	0.00337354	0.12279485	< 1%
PLAT	0.26190368	14.99	0.0193858	0.09334085	5.39
CTSB	3.01391492	81.27	1.97312545	2.94204175	84.55
CTSD	2.80470779	80.41	3.21696629	3.47605726	91.54
CTSK	1.42171921	69.45	1.2512625	1.17190347	70.22
CTSS	1.16643153	32.34	0.06283827	0.35204916	6.53
APC	0.18076037	16.47	0.09655517	0.11971038	12.58
SERPINA1	0.38305545	13.84	35.8982598	19.91700017	96.16
TIMP1	16.6057286	96.52	19.6269541	26.46097394	99.47
TIMP2	3.27863411	85.84	1.16505821	2.70451978	70.93
TIMP3	9.06680252	79.71	1.47113236	2.66871183	54.33
TIMP4	0.28944674	24.83	1.48893885	1.00083381	64.11
IL1RN	0.35324165	5.21	Not detected	0.02356153	< 1%
